# Bridging Knowledge Systems and Perspectives to Inform Salmon Management and Research: A Kuskokwim River Case Study

**DOI:** 10.1002/ece3.72146

**Published:** 2025-10-13

**Authors:** Janessa Esquible, Avery Hoffman, Destiny Ropati, Brooke Woods, Jessica Black, Rachel Donkersloot, Mike Williams, Wilson Justin, Justin Leon, Carrie Stevens, Craig Chythlook, Tazia Wagner, Courtney Carothers

**Affiliations:** ^1^ Great Lakes Fishery Commission Ann Arbor Michigan USA; ^2^ Orutsararmiut Traditional Native Council Bethel Alaska USA; ^3^ University of Alaska Anchorage Anchorage Alaska USA; ^4^ Woodwell Climate Research Center Falmouth Massachusetts USA; ^5^ University of Alaska Fairbanks College of Indigenous Studies Fairbanks Alaska USA; ^6^ Coastal Cultures Research Aniak Alaska USA; ^7^ Kuskokwim River Inter‐Tribal Commission Bethel Alaska USA; ^8^ Althset'nay Dine Anchorage Alaska USA; ^9^ University of Alaska Fairbanks College of Fisheries & Ocean Sciences Fairbanks Alaska USA

**Keywords:** bridging knowledge systems, community‐engaged research, equitable knowledge sharing, relationality, salmon management, subsistence fisheries

## Abstract

In this research, we bridge knowledge systems and perspectives from Indigenous and rural fishers alongside state and federal managers and biologists regarding the state of salmon management and research processes in the Kuskokwim Region of southwestern Alaska. The key objectives were to identify strategies to improve salmon management, document perspectives on Alaska Native inclusion in current management and research processes, and illustrate ways to develop more inclusive management processes and organizations. We also identify key opportunities and barriers to relationship building between Tribes and management agencies. Lastly, we explore perceptions of equity and equality and how research and management account for these dimensions. This was a two‐component research project, with one component being primarily Indigenous‐led and community‐engaged, and the second component involving agency management and research staff. We carried out 28 semi‐directed interviews with 45 Indigenous and community knowledge holders across five different communities from June 2019 to May 2022, in addition to 12 interviews with state and federal managers and researchers in 2023. Our study revealed both key differences and shared understandings between state, federal, and community perspectives regarding salmon management and research and around agency inclusion of Indigenous Knowledge systems and Tribal governments. Shared visions and solutions for improving salmon management in southwestern Alaska and elsewhere reflect a greater need for community and Indigenous empowerment and inclusion in fisheries management and research, in addition to increased relationship building and agency time spent in communities. A key recommendation arising from this study is that trust and respect are precursors to meaningfully bridging knowledge systems. Our team encourages further investigation of current power and resource disparities that prohibit equitable knowledge sharing in fisheries management and research, while identifying broad solutions for improving the current salmon management system given diverse sharing across Indigenous, federal, and state experts.

## Graphical abstract

This article bridges knowledge systems and perspectives from Indigenous and community knowledge holders and federal and state staff regarding the state of salmon management along the Kuskokwim River. This research provides mechanisms for improving salmon management with a focus on relationality, equitable knowledge sharing, and through strengthened partnerships.

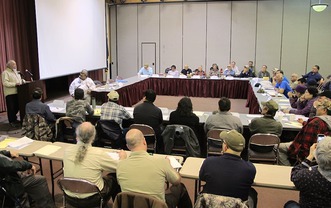

## 1 Introduction

Why are we fighting for subsistence? That's our right to do that, to get food like [we have] for millennia, from generation to generation. That's our cultural‐ and self‐identity. I believe we should be given our freedom to be doing what we have always been doing. **—**Bethel fisherman, March 2021

Since time immemorial, Alaska Natives throughout Alaska have stewarded the lands, waters, fish, wildlife, birds, marine mammals, and plants. These intimate relations and connectedness persist today despite colonial processes of Indigenous erasure and marginalization. While some may think of colonization as an issue of the past, colonial values, norms, policies, and practices of various forms persist. Laurie Richmond (2013) identifies colonial processes as “…dispossession of Indigenous lands and resources and the development of western management institutions to govern the use of culturally important fish resources” in ways that marginalize Indigenous interests. Along the Kuskokwim River, and elsewhere in the world, western management systems compounded with other issues such as climate change, have led to the inability of Indigenous communities to practice their Indigenous ways of life, and including the ability to harvest adequate amounts of fish and other wild foods necessary for the health and wellness of their families and communities (Richmond 2013; Smith et al. 2022).

The United States adopted the United Nations Declaration on the Rights of Indigenous Peoples (UNDRIP) in 2010, which states Indigenous Peoples have a right to ensure and be involved in sustainable management and stewardship of traditionally used fish resources (Richmond 2013; United Nations 2008). While the Kuskokwim River supports one of the largest wild Chinook salmon fisheries in the world, subsistence users have not been able to meet the amount necessary for subsistence^1^ for over a decade, posing significant threats to a way of life that has sustained Alaska Native Peoples in this region for thousands of years (Smith et al. 2022). In other parts of the state, such as the Yukon River, subsistence fishery closures create a disconnect between the people of the land and their fish relatives. The colonial legacy of fisheries management is perpetuated today through continuous attempts to marginalize and exclude Indigenous governance systems in present‐day management (Alfred and Corntassel 2005; Todd 2016; Whyte 2018; Stevens and Black 2019; Carothers et al. 2021). The current top‐down governance authority of conventional fisheries management and increasingly restricted access to salmon in Indigenous communities along the Kuskokwim and Yukon rivers pose social and environmental injustices that must be addressed. In response to these issues, this research evaluated current fisheries management systems through the dual lens of Tribal citizens and Alaska Native knowledge holders in the Kuskokwim Region and state and federal fishery managers, notably Alaska Department of Fish and Game (hereafter, ADF&G or state) and U.S. Fish and Wildlife Service (hereafter, USFWS or federal) agency staff. We conducted this research to inform strategies to improve current salmon management and to identify barriers to, and mechanisms for, strengthening Indigenous inclusion in salmon governance, whereby governance refers to “who makes what decisions, as well as to the set of regulatory processes, mechanisms, and organizations through which different actors influence and become responsible for specific outcomes” (Lemos and Agrawal 2006; Dawson et al. 2021). In this paper, we reimagine a novel fisheries management system that upholds Indigenous rights, sovereignty, self‐determination, and ways of life.

While this research project (Indigenizing Salmon Science and Management; National Science Foundation #1936378) is a statewide project, this paper draws on research carried out in the Kuskokwim Region of southwestern Alaska (see also Esquible et al. 2024). The Kuskokwim River flows from the western slope of the Alaska Range and into the Bering Sea through the Yukon‐Kuskokwim Delta. The Kuskokwim is the second largest river in Alaska, extending for 724 miles, and its watershed is composed of three distinct regions, including the lower, middle, and upper river. While Yup'ik, Cup'ik, Athabascan, Deg Xit'an, and Dena'ina Peoples have inhabited this region since time immemorial, Euro‐Americans and other cultures also reside in the Kuskokwim drainage. The Alaska Native Peoples of this region have deep cultural, physical, spiritual, and economic ties to the land, rivers, and ocean where harvests of fish, wildlife, plants, and other foods occur seasonally and year‐round (Brooks and Bartley 2016). All five Pacific salmon species can be harvested in various parts of the Kuskokwim drainage, including Chinook (*taryaqvak, Oncorhynchus tshawytscha*, king), chum (*iqalluk, Oncorhynchus keta*, chum), sockeye (*sayak, Oncorhynchus nerka*, red), coho (*qakiiyak, Oncorhynchus kisutch*, silver) and pink salmon (*amaqaayak, Oncorhynchus gorbuscha*, humpy; Yugtun, Latin, and colloquial names provided). The cultural importance of salmon cannot be overstated. Nearly every household in the region harvests, provides, or receives salmon for subsistence use (Clark et al. 2018).

Land ownership and management jurisdiction vary on the Kuskokwim River. The lower one‐third of the Kuskokwim River flows through federal lands within the Yukon Delta National Wildlife Refuge, while the middle and headwater sections of the Kuskokwim River primarily fall within state jurisdiction. As a result, salmon is dually managed by the Alaska Department of Fish and Game (ADF&G; state agency) and the Yukon Delta National Wildlife Refuge (YDNWR; federal agency with authority to manage as delegated). The Division of Commercial Fisheries is responsible for managing commercial, subsistence, and personal use fisheries within the jurisdiction of the State of Alaska (ADF&G 2025). In years where a salmon conservation concern exists and when subsistence needs are unable to be met, harvest of that salmon species is limited to federally qualified users, which has resulted in federal management occurring within the YDNWR. The prioritization of harvest by federally qualified subsistence users^2^ is supported under the Alaska National Interest Lands Conservation Act (ANILCA 1979; Coastal Cultures Research 2023). Under state management, there is a subsistence priority, however, the state's equal access clause ensures access of the state's natural resources to all Alaskan citizens (White 1994), thus prohibiting limiting harvest to federally qualified subsistence users. Different legal relations exist between the Tribal, state and federal governments. The YDNWR, as a federal entity, recognizes Tribes as sovereign governments with a Government‐to‐Government relationship. As a result, the YDNWR must fulfill the Trust Responsibility to Tribes and engage in Tribal Consultation regarding conservation of fish, wildlife, and their habitats, as well as protections of cultural, trust and treaty resources where Tribes have subsistence and other rights or interests (USFWS 2022b). Tribal Consultation is defined as “a formal, two‐way, Government‐to‐Government dialogue between official representatives of Tribes and Federal Government agencies to discuss federal proposals before the federal agency makes decisions on those proposals (BIA 2025).” This Government‐to‐Government consultation is unique to Tribes and not more broadly applicable to the general public. In 2022, the Alaska state government formally recognized Alaska's 229 federally recognized Tribes through House Bill 123 (State of Alaska 2022). Despite the existence of a Government‐to‐Government Tribal Consultation policy between ADF&G and the Alaska Board of Fisheries and Game, there is no documentation of ADF&G staff implementing this policy (ADF&G 2022).

**FIGURE 1:**
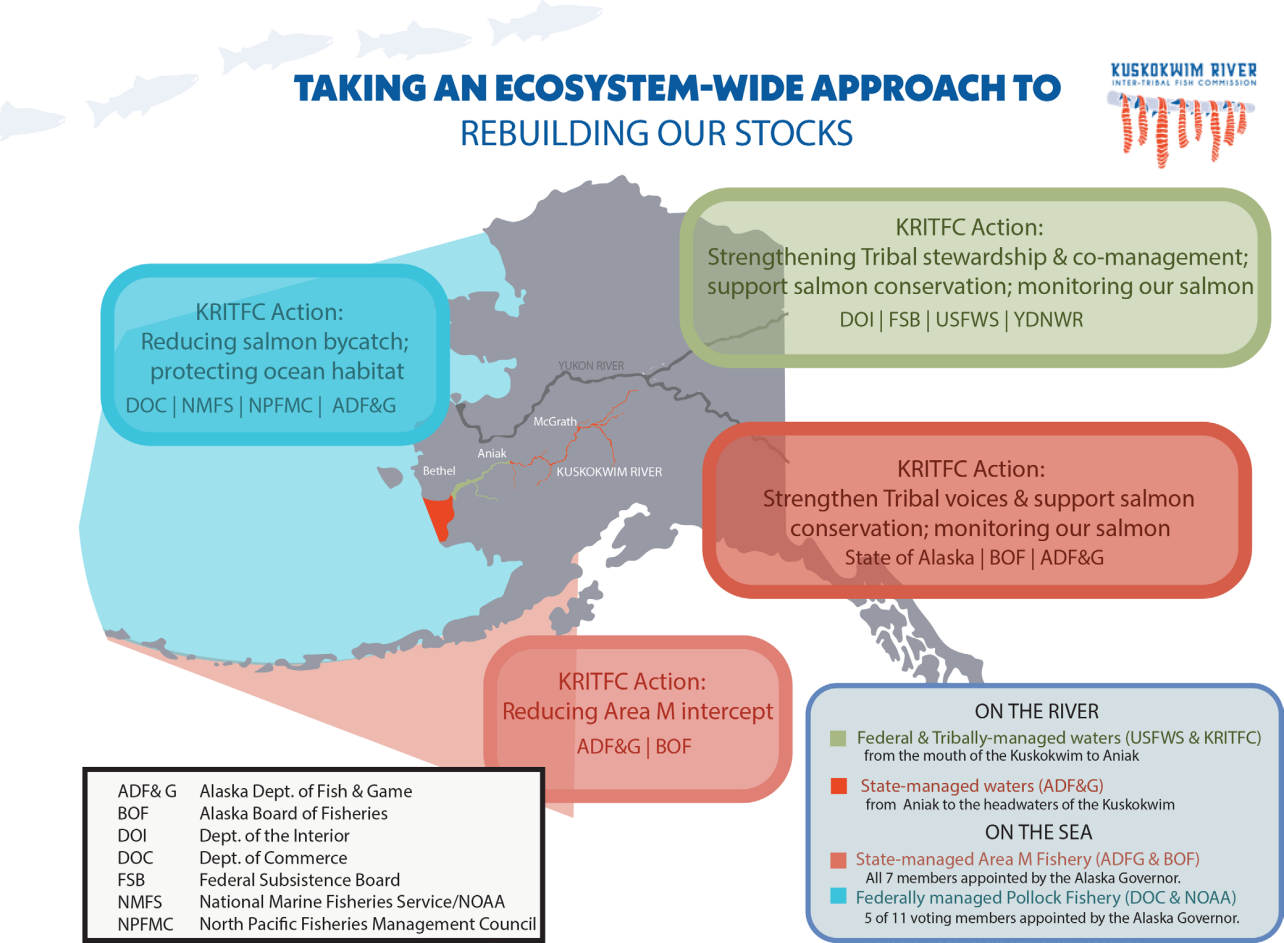
In their anadromous life cycle, Kuskokwim River salmon cross many jurisdictional boundaries, each with its own distinct management agency, management plans, and fisheries. Key management areas/agencies for freshwater (ADF&G, USFWS, KRITFC) and marine (NOAA Fisheries, NPFMC, ADF&G, BOF) life stages are shown here. KRITFC, guided by Kuskokwim Tribes, Knowledge systems, and values, works to take an ecosystem‐wide, gravel‐to‐gravel approach to salmon stewardship. Graphic credit: KRITFC 2025.

In 2015, the Kuskokwim River Inter‐Tribal Fish Commission (KRITFC), representing 33 federally recognized Tribes from throughout the watershed, was established and has since initiated formal consultation with the Department of Interior's USFWS regarding federal in‐season fishery management decisions. KRITFC's primary goal is “to rebuild the salmon resources to support and preserve a way of life that is vital for people's nutritional, economic, cultural, and spiritual needs” (KRITFC 2024). In 2016, a memorandum of understanding was signed allowing for “cooperative management” of federal fisheries on the Kuskokwim River drainage to occur between KRITFC and USFWS (KRITFC 2016). In this agreement, the Federal Subsistence Board (FSB) remains “vested with authority delegated by the Secretaries of Interior and Agriculture to manage subsistence uses and resources on the federal public lands in Alaska” (KRITFC 2016). Since 2016, the FSB delegated regulatory authority related to inseason management of fisheries in the federal public water in the Kuskokwim Area to the Yukon Delta National Wildlife Refuge (YDNWR) manager. As it currently stands, along the Kuskokwim River, the YDNWR and ADF&G remain the primary entities with legal authority to impose or rescind fishing regulations, while Tribal governments and citizens serve primarily in an advisory capacity, with the exception of KRITFC, which has shared authority on fishery management decisions within the Refuge boundaries (see Figure 1).

In the Kuskokwim watershed and across Alaska, there are multiple ways to engage in natural resource management decisions, including: the federal Regional Advisory Councils (RACs), state Advisory Committees (ACs), FSB, state Board of Fish (BOF) and Board of Game (BOG), Subsistence Resource Commissions, the North Pacific Fisheries Management Council (NPFMC) and others. Along the Kuskokwim River specifically, the Kuskokwim River Salmon Management Working Group (Working Group) exists and is composed of Indigenous and non‐Indigenous subsistence fishers, seafood processors (previously), commercial fishers, sports fishers, Elders, RAC members, KRITFC representation, and ADF&G staff (ADF&G 2024). The Working Group was established in 1988 by the BOF in direct response to requests from fishers in the Kuskokwim Area who wanted to have a more active role in the management of salmon (ADF&G 2024). Recently, Indigenous and Traditional Knowledge at the NPFMC and BOF has been increasingly and formally recognized as information to consider in decision‐making processes (NPFMC 2023; ADF&G 2024). Despite the many platforms that exist for public input in fisheries management, it is critical to recognize the sovereignty of Tribes and their correlating role in management. This, in part, catalyzed the formation of the KRITFC, as a co‐management governing body nearly 30 years later.

While many opportunities exist for Tribes and others to engage in fishery management decisions, the majority (75%) of Indigenous and community knowledge holders^3^ engaged in this larger research project do not feel their values are reflected in western management systems (Esquible et al. 2024). Further, in our exploration of the deep knowledge and wisdom shared by Alaska Native fishing families, the most common interview theme was western management. To better understand these findings, we conducted research with federal and state scientists and managers. The key objectives of this research were to identify strategies to improve salmon management, document perspectives on Alaska Native inclusion in current management, identify ways to develop more inclusive management processes and organizations, identify key opportunities and barriers to relationship building between Tribes and management agencies, and explore perceptions of equity and equality and how research and management includes these dimensions. This last objective was included in response to community interviews and previous research that suggested tensions between Indigenous governance mechanisms that center equity, or fairness, compared to western governance systems that tend to center on equality, or equalness (Donkersloot et al. 2020).

## 2 Methods

### 2.1 Community Engagement

**FIGURE 2:**
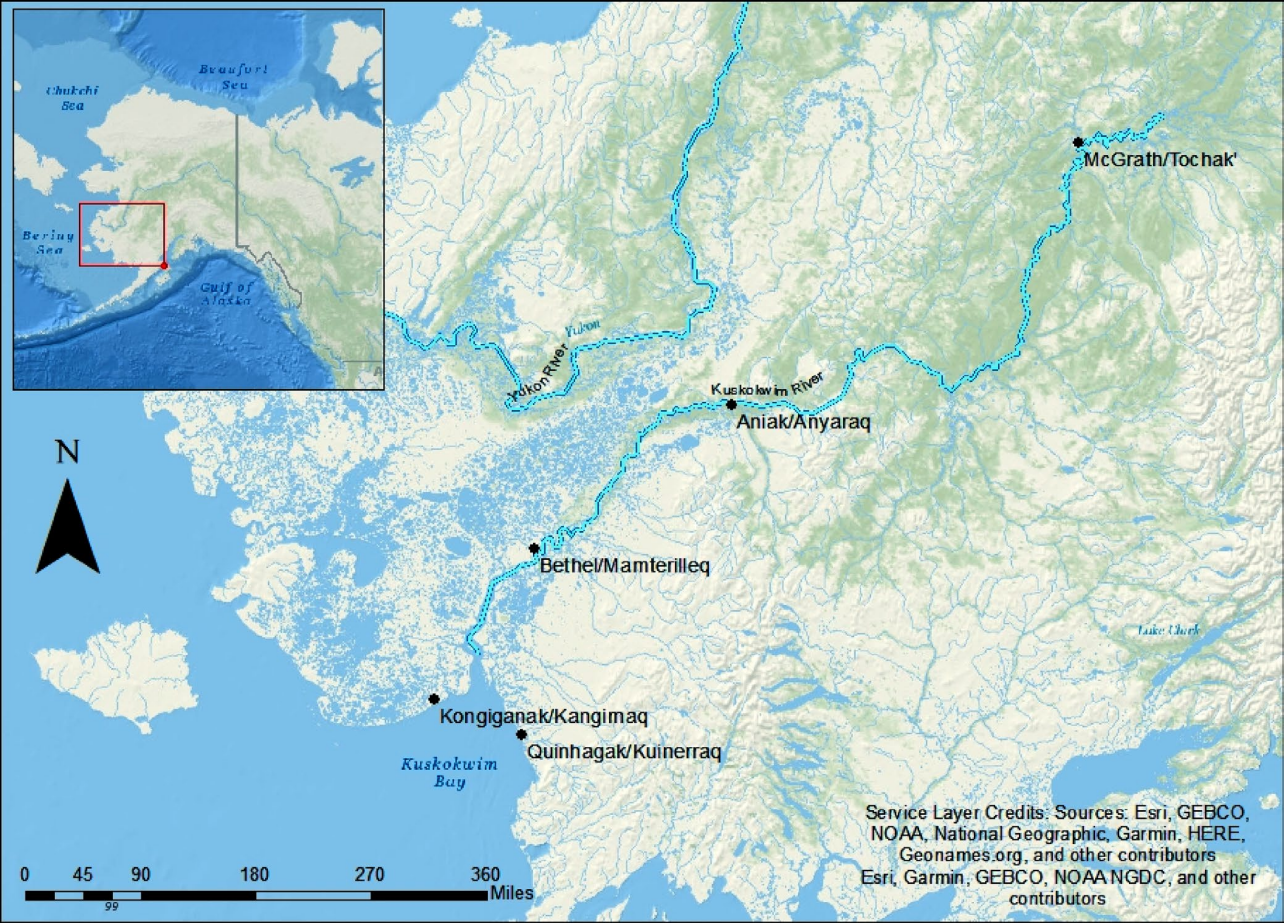
Map from Esquible et al. (2024), showing participating Kuskokwim communities in the Kuskokwim Region.

The first component of this project was Indigenous‐led and guided by six key principles including respect, relevance, reciprocity, responsibility (Kirkness and Barnhardt 1991), relationship, and redistribution (Harris and Wasilewski 2004). This work was grounded in relationality, where relationship building and spending time in communities were centered. We focused on uplifting Indigenous voices, knowledge systems, values, stewardship practices, and governance mechanisms pertaining to salmon. The community‐engaged approach to this research ensured our team respected Tribal sovereignty and deferred to Tribal leadership regarding involvement in this project. Our team utilized circle dialogs and garnered trust through relations (Wilson 2024) as a primary mechanism to catalyze initial relationships and ensure this research was in alignment with local Tribal research priorities (Esquible et al. 2024). We conducted semi‐directed interviews with 45 knowledge holders across five different communities from June 2019 to May 2022. These five communities included the coastal communities of Kongiganak (Kangirnaq) (and Quinhagak (Kuinerraq)) 10, the lower river communities of Bethel (Mamterilleq) and Oscarville (Kuiggayagaq), the middle river community of Aniak (Anyaraq) and the headwater community of McGrath (Tochak; English and Yugtun community names provided; Figure 2). The interview questions were open‐ended (see 3.8 Appendix 1 in Supporting Information. Community Semi‐directed Interview Questions).

Interviews took place with individuals and groups such as multigenerational members of fishing families. Each of these communities has a sovereign Tribal government. Community members include Tribal citizens and other non‐Native community residents. Interviewees across communities were selected utilizing snowball sampling, in which Tribal Council and community members directly identified individual experts and expert families with whom to engage (Huntington 2000). While most interviews took place in person, we conducted some interviews virtually due to COVID‐19 and participant preference. Interviewees included balanced gender representation of women (*n* = 22) and men (*n* = 23), and ranged in age from 19 to 91 years. Interviewees self‐identified as Yup'ik (*n* = 38), Athabascan (*n* = 4), Iñupiaq (*n* = 1), and non‐Native (*n* = 2). The length of interviews varied in length from 0.15 h to 1.75 h, with an average duration of 53 min. While most interviews were carried out in English (*n* = 26), all knowledge holders were given the opportunity to participate in their first language, which for many in coastal and lower river communities was Yugtun. We worked with a local translator, Patrick Samson, who translated all community summary documents from English to Yugtun.

All knowledge holders were provided honoraria as a form of gratitude for sharing their time, knowledge, and wisdom with us. All participants consented to participate in this project (UAF IRB 1146180‐10); most agreed to be audio‐taped, photographed, to be named in the acknowledgments, and to have their interview archived for local and public access. All knowledge holders were given the opportunity to review their transcripts. Participants were also given the opportunity to review quotes and associated salient themes that emerged from their interview. Participants decided how and if they wanted to be attributed in research products developed through this project. A community and regional summary of results shared by all knowledge holders was mailed out to all participants and discussed in person for the communities we were able to revisit in 2023. In 2024, members from our team visited participating communities to determine preferred local options for archiving interviews and follow‐up action items or next steps that our research team can support (see Esquible et al. 2024 for more details on this portion of the research).

### 2.2 Agency Engagement

Results of the community portion of this project informed the second component of this study—interviews with state and federal fishery scientists and managers. We developed an interview guide in direct response to feedback from community knowledge holders, which was then reviewed by our research team and two state research leaders in the Kuskokwim Region (see 3.9 Appendix 2 in Supporting Information. Agency Semi‐directed Interview Questions). State and federal agency staff were contacted via email, phone, and in person at the Arctic‐Yukon‐Kuskokwim BOF meeting in January of 2023. I (lead author) have had the opportunity to work with all of the state agency staff and the majority of federal agency staff engaged in this research in some capacity through my previous work at Orutsararmiut Native Council (ONC) as a fisheries biologist. Therefore, the reconnection with agency staff was straightforward and interactions were grounded in trust and respect built from previous working relationships over my time spent living and working on the Kuskokwim.

At the time of carrying out the interviews, I was employed by the Alaska Department of Fish and Game Subsistence Division. All staff received approval to participate in the project and offered personal and professional perspectives as employees of the Alaska Department of Fish and Game. In total, seven state staff and five federal staff participated in semi‐directed interviews. Eleven interviews were conducted over Zoom and one was conducted in person. The majority of interview questions were asked to all respondents, but given time constraints, some questions were omitted. The length of interviews varied and ranged from 0.7 h to 1.8 h, with the average duration of the interview at 1.2 h. All agency staff were male. Age was not identified. None of the seven states’ staff were identified as Indigenous, whereas four of the five federal interviewees were identified as Alaska Native. All of the state staff were based in the Anchorage, Alaska area (agency's primary headquarters). State staff reported on average 2 months spent in the Kuskokwim region annually. All federal staff were based in the Kuskokwim region, reflecting their office headquarters location, and thus reside in the Kuskokwim region year‐round.

We retained anonymity for all agency staff given the small number of individuals interviewed. Agency respondents were not invited to publicly archive their interviews but were given the opportunity to review transcripts for accuracy. Honoraria were not provided as all interviewees participated during normal work hours. Interviewees were given the opportunity to review and comment on a summary document and key salient themes emerging from their interviews (see 3.10 Appendix 3 in Supporting Information. Grant Project Outline). The primary method of communication for agency staff was email, but phone and in‐person visits also took place.

### 2.3 Participant Observation

Beyond conducting interviews and having conversations with interviewees, I also engaged in participant observation (Bernard 2002), where I had the opportunity to spend time in communities along the Kuskokwim River, with most of my time spent in Bethel, Alaska. This participant observation at times was carried out in conjunction with my employment at ONC, and at other times solely as a student. This participant observation took place in the community, at people's homes, at fish camps, and at management meetings. Throughout my time working in the Kuskokwim Region, I participated in six seasons of summer salmon management meetings, in addition to five seasons of spring, fall, and winter federal fisheries management meetings. My time spent at various federal, state, Tribal, and inter‐agency management meetings all helped to inform and shape this research in different ways.

### 2.4 Data Analysis

All interviews were transcribed and reviewed for accuracy. Knowledge holders and respondents were offered the opportunity to review their transcripts as well. Reviewed and approved transcripts were then uploaded into Atlas.ti (2021), a qualitative data analysis software. All interviews were coded using a grounded theory approach (Bernard 2002). In vivo coding was conducted to identify and refine themes until theoretical saturation was reached (Strauss and Corbin 1990). Themes were ranked according to frequency, and this research elaborates on the more commonly occurring themes in the results sections. This process was iterative, and coding occurred as research continued throughout this project. Responses to questions were also uploaded into an Excel spreadsheet to categorize and to help with the final organization and analysis of all data. The community component of this project entailed spending additional time reviewing the full transcripts, re‐listening to the audio files and having discussions with interested knowledge holders to ensure major points and key themes were accurately represented.

## 3 Results

### 3.1 State of Salmon Management

There's no other people other than local people in this area that are gonna care about salmon more than they [local Native fishermen] do. **—**Federal Respondent 3/8/2023

Federal and state respondents were asked to share how well they thought salmon management was working for communities and salmon in the Kuskokwim Region. Most federal respondents (80%; *n* = 4) felt salmon management was working fairly well, whereas only 29% of state respondents (*n* = 2) expressed similar views. While we did not ask this same question to community and Indigenous knowledge holders, we did document general perceptions of the strengths and weaknesses of salmon management and research. Shared strengths by all interviewees included the different roles the KRITFC (inter‐tribal commission and co‐management entity) plays in fisheries management as well as the collaboration between Tribes and agencies on fisheries research projects including but not limited to the in‐season harvest monitoring project and the Bethel sonar project that enumerates returning salmon during upriver migration. The in‐season harvest monitoring project has a strong community‐engaged element with the Elder fish distribution program and community Chinook salmon sampling program, where local fishers participate in sampling their salmon to help biologists determine the age‐sex‐length trends of Chinook salmon. Additional strengths of the salmon management and research systems were also identified by all interviewees (Figure 3A). Notably, many of the strengths federal staff spoke to were centered around their partnership with KRITFC, the federal salmon management team (primarily composed of Alaska Native fishermen) and how this cooperative management structure has allowed for increased trust by local and Indigenous people and success in conservation efforts. State respondents highlighted successful partnerships with local communities and tribes, management policies, and the public engagement component of their regulatory meetings. Community knowledge holders emphasized ways in which local and Indigenous community members are involved in fisheries management and research, as well as conservation approaches to rebuild salmon populations.

**FIGURE 3A:**
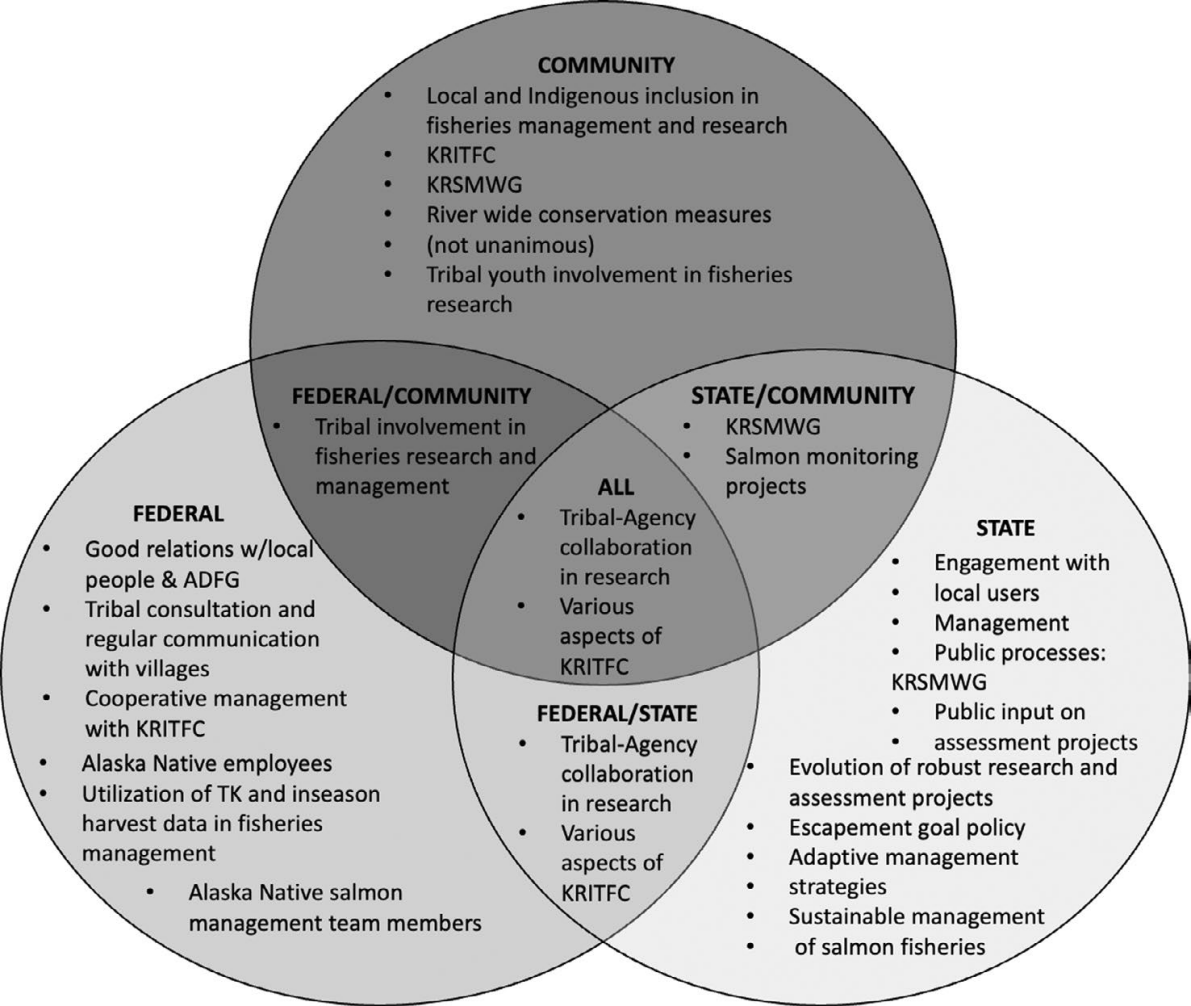
Salmon management and research strengths identified by community and Indigenous knowledge holders, federal, and state respondents.

Federal, state, Tribal, and other community knowledge holders were asked to share weaknesses of the salmon management system as well as opportunities for addressing these weaknesses and mechanisms for improving the management system. Confusion around management regulations regarding whether the river is open for harvest or not was a shared weakness of salmon management among all groups. This confusion was mostly attributed to miscommunication, the complexity of dual (state and federal) management, and the politics associated with salmon management, thus resulting in local subsistence users being in the middle of these issues. Poor relations between management agencies and communities were also shared weaknesses across all groups. Shared responses from agencies regarding the weaknesses of salmon management included: limited funding and capacity to build relationships in communities, inadequate managing and monitoring of salmon fisheries, and inability to carry out proactive research given that management and research are primarily reactive and rarely provide the opportunity to avoid future fisheries issues. Other weaknesses identified in interviews included lack of transparency (e.g., closed‐door management meetings) and missed subsistence fishing opportunities due to restrictions.

Whoever they have on the board or working there, they have no idea what it is like to sit on the bank and see your fish dry or your smokehouse and see your fish smoking or have a big, you know, eating a big fish head and eating your neighbor's food. They have no idea about that. How your whole life revolves around the fish. They don't know that. They just come in with their paper and they do this and this. They have no idea. **—**Aniak Fisherwoman 5/20/2021

Knowledge holders also mentioned federal management of commercial trawl fisheries in the Bering Sea and state management of the commercial salmon fishery in the western Alaska Peninsula and eastern Aleutians (called Area M). Most notably the political and financially driven component of fisheries management, inequitable treatment of subsistence users, and the prioritization of commercial fishing over subsistence fishing were identified as weaknesses by both federal respondents and community knowledge holders. Additional weaknesses were also identified by all interviewees (Figure 3B).

**FIGURE 3B:**
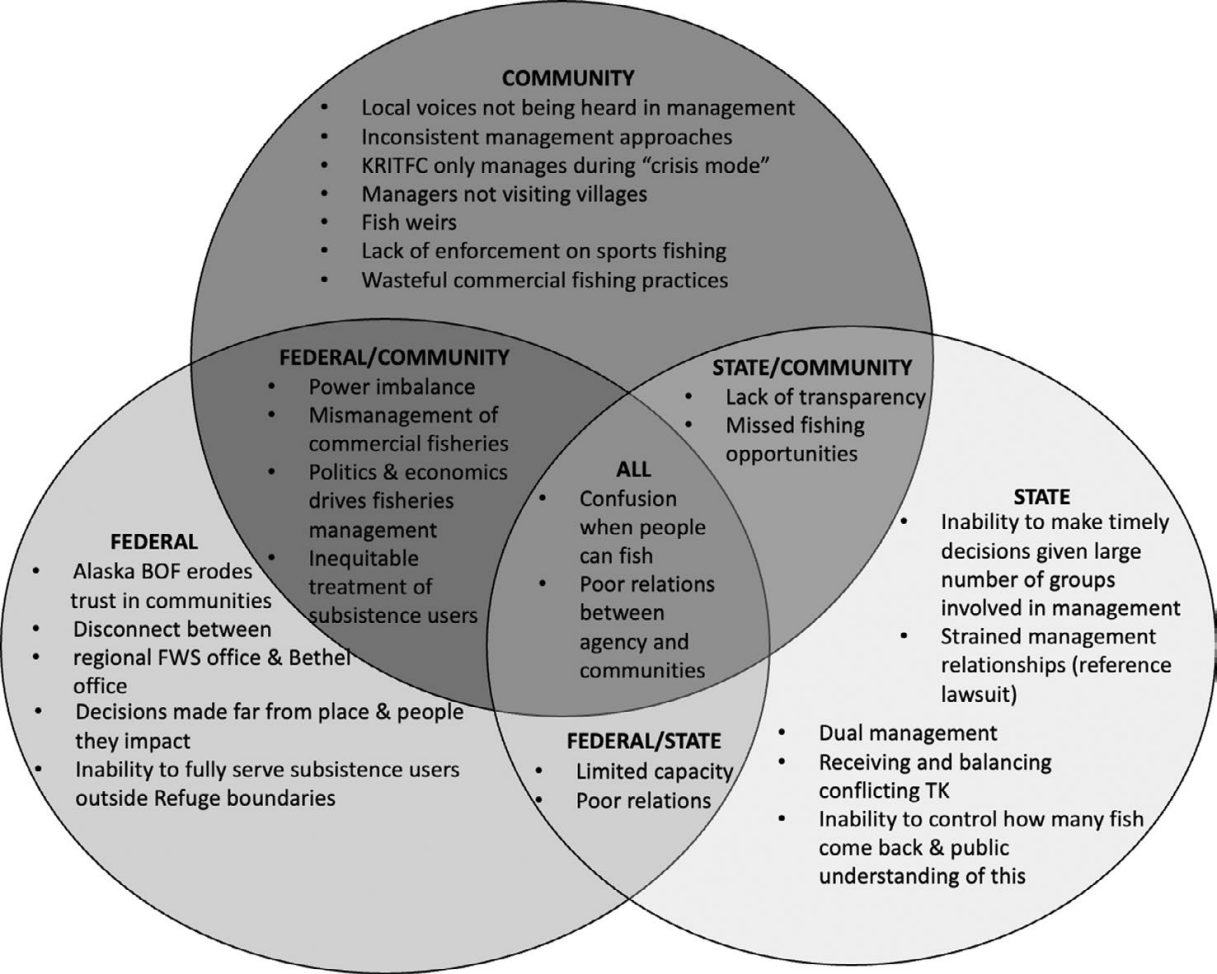
Salmon management and research weaknesses identified by community and Indigenous knowledge holders, federal, and state respondents.

## 4 Alaska Native Inclusion in Current Salmon Management and Research Processes

**FIGURE 4:**
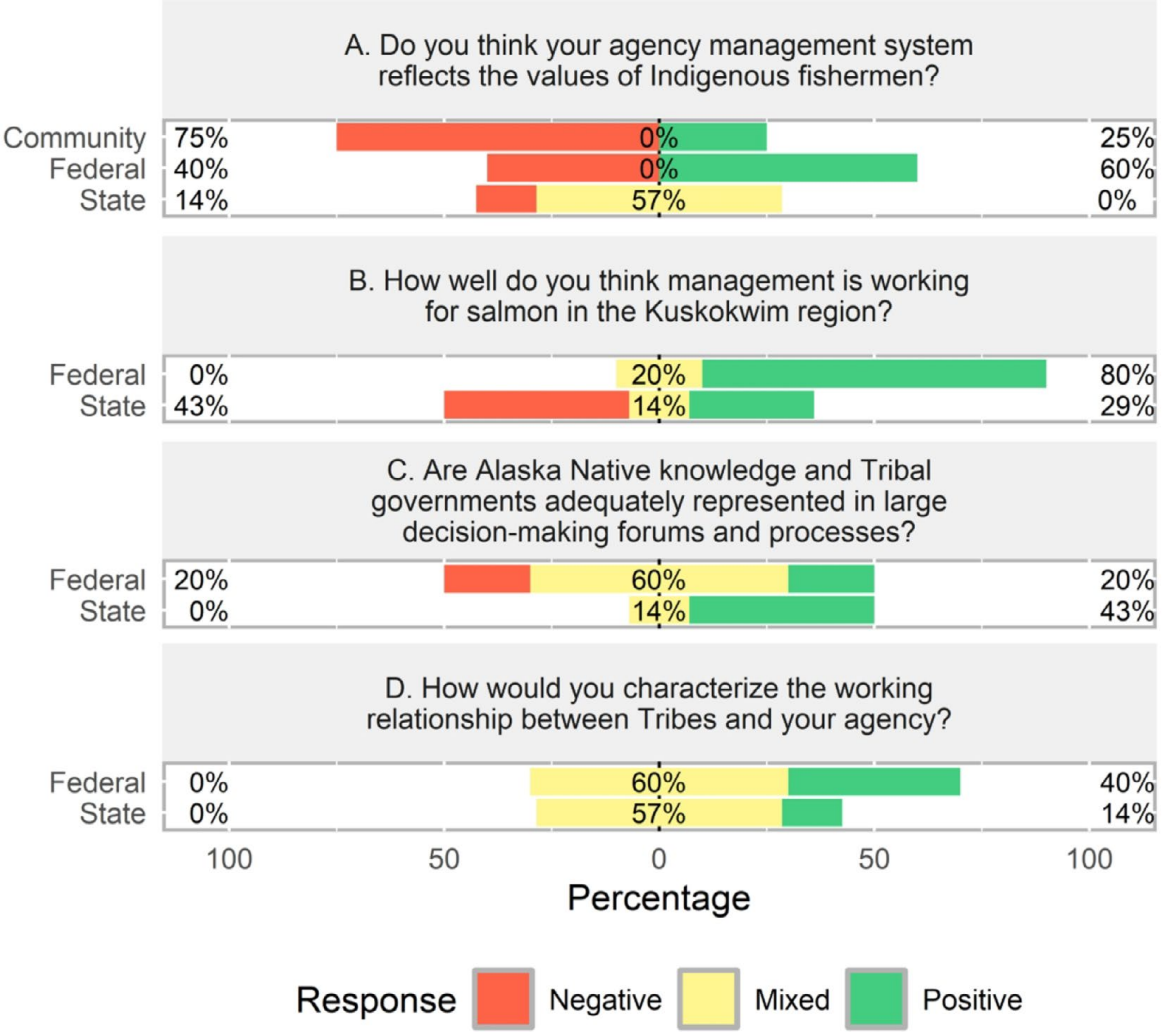
Scale characterizing the state of salmon management, Alaska Native inclusion in salmon management decision‐making forums, and the working relationships between Tribes and agencies, showing results from four questions (shown as A–D, above). Community (*n* = 45), Federal (*n* = 5), and State (*n* = 7) responses are illustrated. State responses do not sum to 100% due to the inability of state respondents to answer all questions for various reasons. Community interviews were conducted first, and this content informed the interview guide for agency interviews, which is why community responses are not included for all 4 questions.

As the majority of community knowledge holders felt their values and knowledge systems were not adequately included in salmon management (75%; *n* = 34), it was critical to follow up with managers and researchers directly to better understand the state of Alaska Native inclusion in current salmon management and research processes. We interviewed five federal and seven state respondents. They were asked the following question, “*Are Alaska Native values reflected in your agency's management and research system/processes*?” Interestingly, the majority (60%; *n* = 3) of federal respondents felt that Alaska Native values were included in federal management and research systems and processes. The remaining respondents (40%; *n* = 2) had mixed perspectives on this (Figure 4B).

The best way for this to happen is to have Alaska Native people actually working in these different positions at the Refuge. **—**Federal Respondent 3/8/2023

Study participants specified this shift in seeing Alaska Native values reflected in federal management and research systems as a rather recent occurrence, and through the formation of the salmon management team that is primarily composed of Alaska Native Refuge staff who “live and breathe fishing.” Mechanisms for Alaska Native inclusion entail hiring Alaska Native staff, working with Tribal governments, spending time in communities, and working with the KRITFC. In striking contrast, 0% of state respondents felt that Alaska Native values were included in state management and research systems and processes. State interviewees gave mixed responses (57%; *n* = 4), were unable to answer the question (29%; *n* = 2), or responded no (14%; *n* = 1; Figure 4B). Opportunities for including Alaska Native values in state management and research systems and processes were tied to the state's responsibility to ensure healthy, sustainable salmon runs for future generations and considering the needs of the whole river, which may allow for equity in the harvest of salmon across the Kuskokwim River watershed.

That's the foundation of what we do is providing for the future. **—**State Respondent 2/13/2023

These principles were also perceived by some as Alaska Native values which are embedded in the state management and research systems.

We then asked state and federal respondents if they felt Alaska Native knowledge and Tribal governments are adequately represented in large decision‐making forums like state BOF and federal FSB. The majority of federal responses (60%; *n* = 3) were mixed (Figure 4C). State respondents also had variable responses. 43% (*n* = 3) felt that Alaska Native knowledge systems and Tribal governments were adequately represented in larger decision‐making forums, while others (43%; *n* = 3) could not answer the question, and one response (14%; *n* = 1) was mixed (Figure 4C).

Federal and state respondents were also asked about whether Indigenous Knowledge (IK) systems were included in agency research and management processes and, if so, how.

Everything we do involves Indigenous knowledge. **—**Federal Respondent 3/6/2023

The majority (80; *n* = 4) of federal respondents felt IK and western science both guide salmon management decisions. In terms of inclusion of IK in salmon research and management, the guidance for doing so has been made very clear by the Biden Administration and secretarial orders given to the YDNWR. Currently, the cooperative management structure with KRITFC allows for local Alaska Native fishers to be directly engaged with and guiding salmon decision‐making processes. Ways in which IK is included at the Refuge include hiring local people through the local hiring authority (over 50% of Refuge staff are Alaska Native), the Refuge Informational Technician (RIT) program, regular Tribal Consultations, and their partnership with the KRITFC. The majority of respondents see both western science and IK as bodies of knowledge that directly guide research and management processes at the Refuge. However, this was perceived by some to be at risk of changing depending on who is in the lead management position.

All state respondents saw value in being able to listen to information from IK and local knowledge holders; however, the responses spanned a very large range in terms of how these knowledge systems guide research and management by their agency, which is why responses were not quantified.

As a manager you want to take in all available information. The more informed you are, the better decision you can make. **—**State Respondent 2/10/2023

While respondents want to allow for both bodies of knowledge to guide management, mechanisms for doing so were not clear or consistent. Respondents' perspectives elucidated western scientific quantitative data, what some respondents refer to as “hard data”, regularly guides management and research processes. However, examples of inclusion of IK in fisheries research and management were provided. These included: guiding new research efforts like weir placements, using IK to “ground truth” assessment project data, listening to knowledge holders at public meetings, learning how to fish, use boats and repair nets from locals, and leveraging IK with western scientific data to make decisions. One respondent shared that their decisions and actions must be “defensible” and spoke to “legal ramifications” of only relying on IK in salmon management. Another respondent claimed that most staff do not have the authority to incorporate IK into their work, and thus, this would likely need to be done in the form of a co‐management agreement, which does not currently exist. The ADF&G Subsistence Division (primarily social scientific division) was identified as the entity that carries out much of the work that documents IK, but this may not translate into actionable information for salmon managers and the Commercial Fisheries Division.

## 5 Community and Agency Relationships

The sooner we put more emphasis on relationship building and rebuilding, the sooner positive things will happen. **—**Federal Respondent 3/6/23

Some of the weaknesses identified by community knowledge holders included poor or no relations between managers and the villages. For this reason, we followed up with agency managers and researchers to characterize relationships between their agency and the villages and to identify opportunities and barriers for building relationships with the villages across the Kuskokwim watershed. All agency respondents emphasized the importance and value of building relationships and spending time in Kuskokwim communities. However, responses in terms of who and how this is carried out varied greatly across agencies. The majority of federal (60%; *n* = 3) and state (57%; *n* = 4) respondents characterized relationships between their agency and villages as mixed (some positive and some negative; Figure 4D). Agency staff revealed critical barriers for improving and building relationships and spending more time in communities. Federal respondent barriers included low pay for staff whose primary responsibility is community engagement, outreach, and education; time constraints and difficult travel; manager turnover; limited staff and capacity; language barriers; past exclusion of Alaska Native Peoples from management and other decision‐making processes; and the lead manager having too much power and control. “One person can make drastic changes.” Thus, if relationship building is not a priority for the lead manager, this individual can, and has, led to poor relations between their agency and communities.

State respondents identified additional barriers, including low capacity in terms of funding, staff, and flexibility in travel planning; closure of commercial fishing operations; timing conflicts; too many management meetings; a vast geography; hostility faced in villages; and needing to have an agenda to travel to a community. One respondent stated that the Commercial Fisheries Division does not emphasize these interactions much, while another respondent noted it was unfair for the average employee to have to carry the responsibility of all relationship building and stressed the importance of this needing to be a two‐way street between the agency and communities. Respondents noted limitations to biologist positions doing the relational aspect of this work in addition to lack of authority of staff, thus preventing engagement with Tribal Council leadership. The average fisheries biologist often does not have the capacity to spend in communities “just for the sake of developing relationships.” Despite the many barriers presented by federal and state respondents, all staff expressed hope in terms of building stronger relations in communities. These opportunities for community engagement and genuine relationship building were primarily centered around more physical time in the community and increased in‐person dialog with community members as well as increased engagement with communities at in‐person advisory body meetings and with youth through fisheries educational outreach programs at local schools.

## 6 Responsibility for Ensuring Equity or Equality in Fisheries Management and Research

Given the increasing attention on equity and environmental justice in fisheries (Burnsilver et al. 2022; Donkersloot et al. 2020; Esquible et al. 2024; NASEM 2024; NOAA 2022), agency respondents were asked to share their understanding of equity and equality in fisheries. Due to time constraints, only nine of the twelve respondents were asked this question. Responses regarding aspects of equity and equality differed among agencies. Federal responses focused more on their agency centering equity in the workplace, hiring process, and through partnerships with Tribal governments and KRITFC. Understandings around equity and equality varied among state respondents. One respondent viewed the two as “the same thing,” and referenced needing to distribute harvest in a way that everyone along the river is given time to catch what they need. Another respondent viewed equality as everybody getting the same and equity as the outcome. Three respondents shared similar examples of how equity is achieved in management actions, more specifically through allocation of harvest. This translated to allowing people more time to fish upriver because they harvest less fish than in the lower section of the river. This management action also prevents salmon stocks from being impacted differentially. One respondent stated that a focus on these concepts is outside of the realm of their work.

Federal respondents highlighted some of the same inequities reflected in community interviews. For example, differential treatment of commercial and subsistence user groups was brought up as an example in a February 2023 BOF meeting. “Those that don't have money don't get treated as fairly.” The respondent highlighted the financial hardship of rural travel to attend BOF meetings, language barriers, the intimidating process of formal meetings, and the disparity in resources that subsistence users have for management meeting engagement compared to commercial fishing industries. Another respondent referred to allocation and shared how they try to allow everyone to have equal harvest opportunity but explained the difficulty in doing so given that historically people living by the mouth of the Kuskokwim River and in lower river communities have always harvested greater numbers of salmon. The Alaska National Interest Lands Claim Act (ANILCA) was referenced in the context of subsistence priority, and how as a result of ANILCA, KRITFC and the YDNWR can collaboratively manage the fishery and restrict fishing to federally qualified users only, addressing issues of equity in accessing the resource. Lastly, issues of communication were brought up given language barriers. The federal agency has a position whose role helps to bridge the gap between the villages and the YDNWR by ensuring management and research‐related communication is clearly conveyed and exchanged with communities and the YDNWR. In a way, the RIT program addresses issues of equity by hiring staff who speak the local language and can effectively communicate with people in the villages who are also first language Yugtun speakers.

Roughly half of the respondents were asked whether their agency has a responsibility to provide for equity or equality in research and management processes. Unanimously, federal interviewees responded yes. For example, at the YDNWR, they have an internal hiring process and have opportunities to implement and improve processes addressing equity and inclusion in salmon management. This includes trying to be a better partner to KRITFC, visiting neighboring villages, and having informal conversations beyond formal consultation with villages. The pillar documents created by the USFWS also reference equity, inclusion, and fairness in the workplace. State staff responses varied, and some did not think ADF&G managerial duties have anything to do with equity “…That's the Board [of Fisheries] process, and we just implement the plan as best we can.” State management is bound to the State of Alaska constitution which includes the equal access clauses, which may clash with aspects of equity in fisheries. From a research perspective, one respondent identified issues of equity in coordinating with remote communities given their lack of reliable Internet, phone service, staff turnover, and navigating bureaucracy.

### 6.1 Potential Strategies and Solutions for Change

During the community research component of this project, we summarized key weaknesses of salmon management systems to share with federal and state agency staff in hopes of identifying potential solutions. The major weaknesses shared with agency staff included lack of transparency, poor communication, and inconsistent approaches between different management entities. We note that we did not specify a management entity when we asked community knowledge holders to share their perspectives on what is working well and what is not working well in salmon management. Thus, the weaknesses identified in the community component of this research reflect the general weaknesses of salmon management. Community and Indigenous knowledge holders identified several strategies for addressing primary weaknesses (see Table 1). Increased local and Indigenous inclusion in fisheries management and research was a key solution to improving salmon management.

**TABLE 1:** Recommendations for improving aspects of salmon management and research.

Category of change	Recommendation	Target group(s)	Target group(s)	Recommendation
Lack of transparency	Open‐door management meetings; easily accessible and interpretable fisheries data; managers and biologists spend more time in community beyond	Management agencies and co‐management organizations; local fisheries research organizations	Management agencies and co‐management organizations	Open‐door management meetings; easily accessible and interpretable fisheries data; managers and biologists spend more time in community beyond
Poor communication	Language translators present at management meetings; identify main mechanisms for information exchange in communities; creation of Tribal and community liaison positions; increased coordination with ADFG subsistence division staff	Management agencies and co‐management organizations; local fisheries research organizations	Management agencies and co‐management organizations	Language translators present at management meetings; identify main mechanisms for information exchange in communities; creation of Tribal and community liaison positions; increased coordination with ADFG subsistence division staff
Inconsistent management approaches	Increase collaboration between management entities; increase unity and communication regarding discussion of management actions; increase preseason and postseason communications with communities regarding management decisions	Management agencies and co‐management organizations	ADFG; USFWS; YDNWR; KRITFC	Increase collaboration between management entities; increase unity and communication regarding discussion of management actions; increase preseason and postseason communications with communities regarding management decisions
Local and Indigenous inclusion	Increase local and Indigenous hires on management and research teams; support Tribal and community‐based research; uplift and support Indigenous traditional and stewardship practices in western management practices; provide opportunities for local and Indigenous knowledge inclusion in fisheries management and research	Management agencies and co‐management organizations	ADFG; USFWS; YDNWR; KRITFC	Increase local and Indigenous hires on management and research teams; support Tribal and community‐based research; uplift and support Indigenous traditional and stewardship practices in western management practices; provide opportunities for local and Indigenous knowledge inclusion in fisheries management and research

## 7 Discussion

### 7.1 Salmon Management, Stewardship, and Sustainability

**FIGURE 5:**
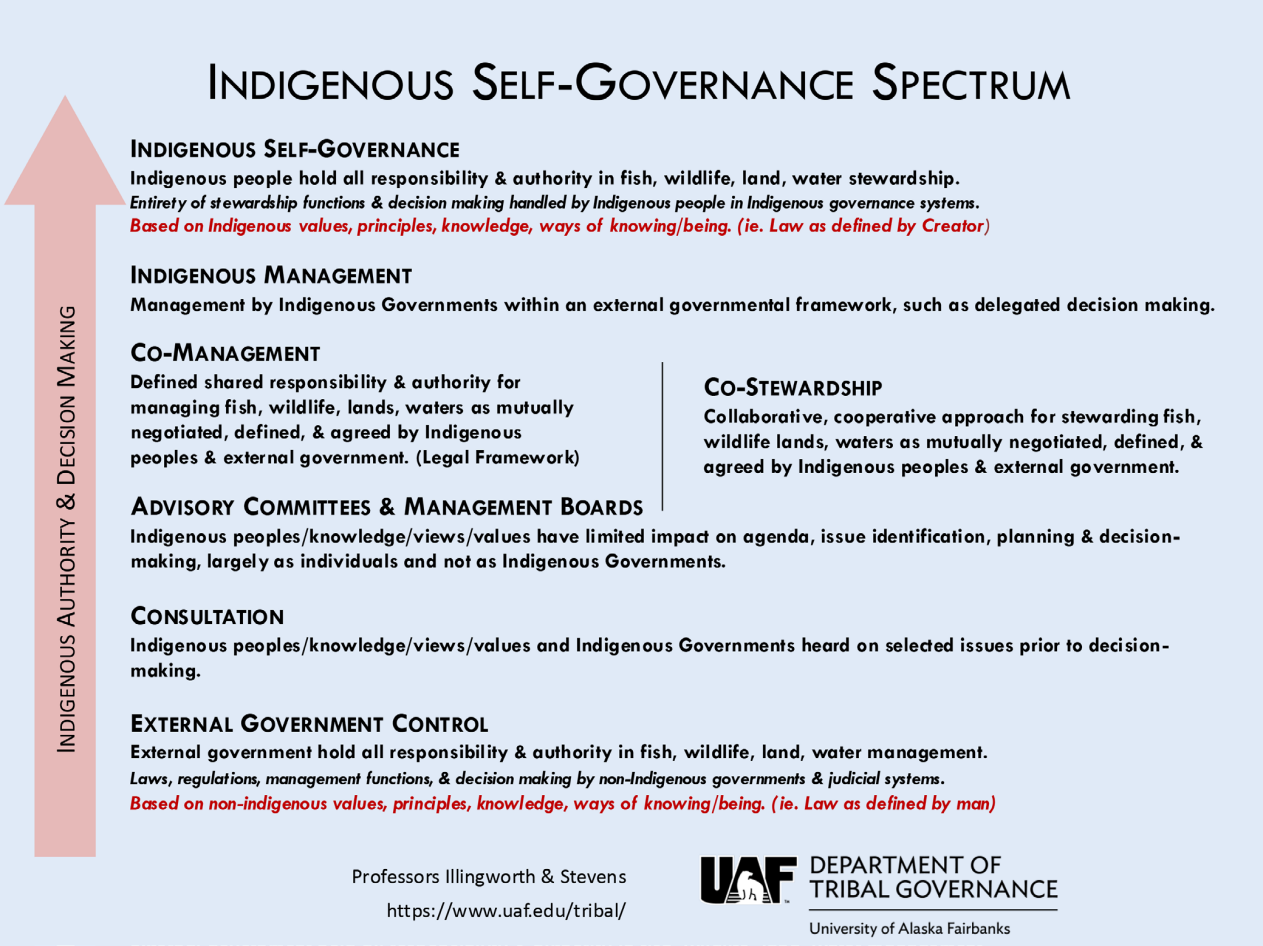
Indigenous self‐governance spectrum produced by University of Alaska Fairbanks, Department of Tribal Governance, Professors Kevin Illingworth and Carrie Stevens.

In this research, we compare perspectives of primarily Alaska Native community knowledge holders, federal, and state scientists and managers. We do so in efforts to find common ground, barriers to Alaska Native inclusion in fisheries management and research, and to develop a greater understanding between all groups. Additionally, our team conducted this research to produce more meaningful and sustained outcomes for salmon, Salmon Peoples, their health, and well‐being. Shared strengths of salmon management and research identified across all groups were centered around collaboration between Tribes and agencies on research projects and various aspects of KRITFC, including their role in co‐management and in management‐related communication efforts. These shared strengths build upon the work of many scholars who have identified the benefit of incorporating diverse knowledge systems in fishery science and governance (Alexander et al. 2019), the processes of building trust and resolving conflict in co‐management (Berkes 2009), and the involvement of local communities and Indigenous Peoples as it relates to effective and equitable conservation (Carothers et al. 2021; Dawson et al. 2021). Many of the Alaska Native knowledge holders and federal respondents associated salmon management strengths with more Indigenous responsibility and authority through forms of Tribal Consultation, and co‐management processes, whereas state responses regarding the strengths of salmon management reflected continued top‐down control with inclusion of the public more broadly in advisory committee and management boards. Figure 5 illustrates the movement from top‐down external government control to co‐management, and Indigenous self‐governance, which is supported by many Alaska Native knowledge holders who participated in this research.

The state BOF is a fisheries decision‐making body public highlighted in this research. Although many state biologists and managers highlight the open, transparent processes of the BOF, historical analysis revealed inadequacies and dominance of state agencies and certain stakeholders, and inefficacy of Tribal engagement, thus questioning the success of public engagement and participation at BOF (Krupa et al. 2019). Co‐author and Ahtna Elder Wilson Justin also recently shared that he was unable to find any record of his testimonies at BOF meetings, suggesting a need to improve accessibility and transparency of historical and contemporary BOF public testimony records (pers. Communication, 2/20/24). Krupa et al. (2019) suggest evaluating BOF utilizing a framework of efficiency, equity, and effectiveness, which could benefit many different regulatory bodies in Alaska and elsewhere.

All groups also identified common weaknesses in current salmon management and research processes, which included inconsistent approaches across management entities and confusion about fishing openings and locations. This confusion was rooted in poor communication and the complexity of dual management. Additional remarks regarding this situation were well articulated by a Bethel fisherman in a local news story published in summer 2021. “It's just crazy,” said Kuskokwim fisherman Tim Andrew, who called into the Fish Talk program, saying that the state is trying to exert its sovereignty over federally managed waters.

The only people that are going to get hurt are the subsistence fishers, because they're the ones that are going to get cited. They're the ones that are going to be confused. And, above all, it may hurt our resources. **—**Timothy Andrew, Bethel 6/4/2021 (Kim 2021)

This comment was in reference to ADF&G creating a fishing opportunity during the same time that federal co‐management entities chose to keep the river closed due to conservation concerns. When asking state respondents to share their perspectives on inconsistent management approaches as a weakness of salmon management, one individual shared very similar sentiments to Tim, “it's just crazy to me to be honest”, speaking to the confusion caused by inconsistent fisheries management approaches. This was in specific reference to the State of Alaska announcing a Kuskokwim River fishing opener that the federal managers stated was illegitimate (Kim 2021). There was great confusion and fear among subsistence fishermen unsure of whether they would get cited for fishing. This level of confusion, miscommunication, and dysfunction is in part rooted in poor relations and poses a significant cost and unnecessary burden to subsistence fishermen and has resulted in a lawsuit due to conflicting management actions simultaneously announced by both state and federal managers (State of Alaska 2023). This lawsuit is ongoing, despite the most recent win by the federal government (Turtle Talk 2024). Legal challenges continue to pose barriers to federal, state, and Tribal relations across Alaska, and are often deeply rooted in commodification of traditional resources.

Another shared weakness across all groups was poor relations between management agencies and communities. Shared weaknesses identified by agency respondents included limited funding and capacity, and subsequent inability to carry out research and some management actions in a proactive manner. Another weakness identified by community knowledge holders was transparency:

No they're [fisheries managers] going to do what they're going to do already. They're just having the meetings to make it seem legal that they're getting the community's advice or hearing the community but chances are their minds are already made up before they even ask us anything or we've talked about it. **—**Aniak Fisherwoman 5/20/2021

For this reason, it is vital that agencies work hard to build trust, be open to community feedback, be proactive and create transparent processes as ways to resolve conflict and address salmon management weaknesses (Christie et al. 2017). Transparency, in this context, can increase informed participation (Bennett et al. 2021) and accountability (Mason 2008) in fisheries management. Community and Indigenous knowledge holders emphasized the importance of open‐doored management meetings, increased management staff and Tribal council^4^ communication and expansion of management‐related communication methods across the Kuskokwim watershed as mechanisms for increasing transparency in management. Federal staff hope to engage in more frequent village visits with their management partner, KRITFC, allowing for more dialog and opportunities to build relations with communities. State staff identify the need to work with communities to learn more about what methods of communication work best for them, and hope to support communities interested in carrying out their own research projects. Ultimately, federal respondents viewed increasing Alaska Native representation in management as a primary mechanism to increase trust, understanding, and transparency in fisheries management. This is carried out in co‐author Tazia Wagner's home community of Metlakatla, Alaska. Metlakatla Indian Community, located in southern Southeast Alaska, is exemplary of complete local and Indigenous inclusion in fisheries management in accordance with Bureau of Indian Affair Northwest Region. Enhanced inclusion of Indigenous communities in fisheries management can lead to greater sustainability and resilience as well as more effective management (Lee et al. 2019; Jokiel et al. 2011).

I'm a firm believer in trying to get local people qualified, educated to get these jobs with fish and wildlife service, fish and game, fish commission, ONC [Orutsararmiut Native Council] and other folks that are involved with these fisheries that can bring not only something they learn from college, schools, the sciences, but also the cultural component, that understanding of the people, the true meaning of subsistence. **—**Federal Respondent 3/8/2024

Local Indigenous community members with academic training in western sciences and/or with scientific staff may help resolve misunderstandings between locals and the agency as well as bridge Indigenous and non‐Indigenous institutions (Reo et al. 2017). However, the onus shouldn't be on Indigenous Peoples alone; western scientists should also seek training and experiential learning in Indigenous Peoples, languages, places, peoples, and ways of being. In an Alaska context, agencies could explore partnerships and opportunities to support Tamamta graduate students and pathways, which may lead to increased Indigenous representation in state and federal agency staff (Tamamta 2024). Tamamta is a Yup'ik and Supiaq word meaning ‘all of us,’ that focuses on bridging Indigenous and Western sciences to transform graduate education and research in fisheries and marine sciences (Tamamta 2024). Similar programs nationally and internationally could result in transformation across academia and fisheries management as more Indigenous students and non‐Native students working with Indigenous communities center community‐led research initiatives and Indigenous knowledge systems in addition to ethically bridging Indigenous and western sciences in marine and fisheries management and research.

Other solutions for improving salmon management identified in state interviews included the creation of a new Tribal engagement role given the respondents' limited capacity and ability to travel to the villages and consult Tribes on various fisheries‐related issues. Such a role is also encouraged by Reo and co‐authors (2017) in improving multi‐actor environmental stewardship with Indigenous Peoples. Curiosity was expressed regarding how to improve communication efforts, and state respondents are looking to the community for solutions regarding this challenge. Perhaps through increasing the representation of in‐river residents within the state fisheries staff, we can achieve greater understanding and enhance communication with fishing communities. For this to occur, local hiring authority should be further explored, particularly among state leadership, given this is already prioritized in federal hiring. Another recommendation included improving the effectiveness and efficiency of the KRSMWG advisory body. Perhaps shifting away from limiting much of the agency engagement with communities in regulatory meeting venues and moving toward meetings in untraditional places like community centers, harvest locations, and fish camps could help transform relationships and encourage greater sharing, particularly by IK holders (Holtgren and Auer 2023; Esquible et al. 2024).

## 8 Alaska Native and Indigenous Knowledge Inclusion in Salmon Management and Research

Our results revealed differences in perceptions on whether and how Alaska Native values and IK are included in federal and state fisheries management and research. Federal respondents were fairly optimistic that their agency reflected Alaska Native values and included IK in management, primarily through Alaska Native employees and regular communication with Tribal governments in surrounding communities. However, interestingly, one respondent did feel western science guides much of their co‐management decision‐making processes since the composition of some of the Tribal inseason managers no longer included as many Elders and given the treatment of IK by western scientists as anecdotal “at best”, reflecting the continued dismissive treatment of IK as anecdotal, lacking objectivity, and reliability (Holtgren and Auer 2023; Simmson 1999). In response to this, it is important to analyze management frameworks to assess the structures and underlying assumptions of management (Nadasdy 2005) and whether they actually reflect Indigenous ways of knowing. Additional training and cultural awareness for western scientists on IK systems and working with Indigenous communities should be considered. The importance of including youth in fisheries management and research was also emphasized in this research. Involving youth is critical for “ensuring the long‐term viability” of Indigenous involvement in management and research (Reo et al. 2017), and also reflective of Indigenous values which are centered around forward thinking and sustainability. Lastly, one federal respondent hoped to see more Alaska Native Peoples in the “hot seat”, also referred to as higher level decision makers, reflective of the importance of shifting more of the decision‐making power to Indigenous Peoples (Holtgren and Auer 2023; Popp et al. 2018; Richmond 2013). Just recently, the Department of Interior and Department of Agriculture proposed a new rule to expand the FSB with three new public members nominated by Tribes, thus expanding Tribal involvement in resource governance (USFWS 2024a The Knowledge Network for Biocomplexity.).

State responses regarding the inclusion of Alaska Native values and IK inclusion in salmon management and research varied greatly, and suggested different understandings of Alaska Native values, with some unable to respond because they did not self‐identify as Alaska Native. This research revealed that federal respondents have more guidance by the Biden Administration and through secretarial orders to work with Indigenous Peoples, acknowledge IK as best available science and include IK in their research and management (Office of Science and Technology Policy Council on Environmental Quality 2022), whereas state employees did not. While state fisheries managers take into account best available information, they are unsure of how to include IK into their management decisions and research and whether they even have authority to do so. In fact, the State Tribal Consultation policy excludes Government‐to‐Government consultation with Tribes on matters related to, “emergency order (EO) announcements and management actions; preseason management strategies, outlooks, or forecasts” among many other things (ADF&G 2022). This reveals critical barriers to consulting with Tribal governments on a Government‐to‐Government basis regarding important management decisions, and limits Tribal engagement with DFG Commercial Fisheries Divisions to public spaces like the KRSMWG, where Tribes are treated as a stakeholders, as opposed to self‐determined Nations with governance systems rooted in deep time, pre‐existing any settler colonial structures (Brooks 2022; Reo et al. 2017). This research revealed that oftentimes, IK and LK are taken into consideration by state researchers and biologists and need to fit into their western scientific understanding of the fishery, thus perpetuating colonial power imbalances (Nadasdy 1999), and suggesting a need for a decolonial approach to better create processes that are truly inclusive of Indigenous worldviews (Pictou 2017). The key question still remains, what are the mechanisms for better including IK into decision‐making processes and research in a meaningful way?

Some examples provided to us in this research included Alaska Native Peoples serving as Tribal fisheries managers and including an agenda item for IK discussion at fisheries management meetings. Other forms of IK inclusion in research were utilizing IK to ground truth other scientific projects or to guide the placement of salmon weirs, again treating IK as a body of knowledge only intended to complement western science (Menzies and Butler 2006). We also heard IK referred to as “anecdotal” by one agency respondent, and attributing any IK‐related research and engagement to be carried out as separate from the ADF&G Commercial Fisheries Division. Treatment of IK as “anecdotal” perpetuates colonialism and delegitimization of IK systems. Aleut Elder Larry Merculieff, in 1990, shares this wisdom with us:

…the Native information is anything but anecdotal. It comes from an individual with an invaluable storehouse of information and knowledge about the environment which is irreplaceable and nonreplicable. This knowledge tells us the person, for example, that environments are in a constant state of flux. By the time the scientist formulate data with adequate time series to make information useful, the model probably will be outdated. **—**Elder Larry Merculieff 1990

At a recent BOF Committee meeting, the State Commissioner said Traditional Ecological Knowledge is best available science (BOF 2023). “As such, access to these knowledge systems should be an important part of informing Board decisions through their close proximity and intimate, often long‐standing, relationships with fish resources, the environment, and the ecological systems that are critical to fishery sustainability (ADF&G 2024).” Perhaps this will result in acknowledgment of value Indigenous Peoples and IK systems can bring to resource management, and better inclusion in future state management processes.

As we continue to see fisheries management struggle with incorporating place‐based observations and values (Silver et al. 2022), we suggest managers and scientists become better educated about the, “…social, cultural, and historical dynamics of the resources they manage and develop capacity to work with diverse communities” (Richmond 2013). The federal employees we interviewed were required to take the Alaska Native Relations Training class as new employees, and this may have resulted in increased trust, respect, understanding, and improved cross‐cultural communication between agency staff and managers (USFWS 2024a). Co‐author Ahtna Elder, Wilson Justin, who helps facilitate the course characterized the training as transformational (pers. communication 2/20/2024). All resource agencies working with Indigenous communities can be better prepared and equipped to carry out their work by becoming better educated on colonial histories and associated perpetuated traumas, in addition to understanding they are entering a space where their knowledge is not authoritative (Holtgren and Auer 2023). This trust, respect, and understanding must be built in efforts to successfully bridge knowledge systems in fisheries research and management, through processes including co‐production of knowledge (Ellam Yua et al. 2022), co‐governance, and the Two‐Eyed seeing approach (Reid et al. 2024). Additional guidance can be found in the White House government‐wide guidance document for federal agencies on recognizing and including IK in federal research, policy, and decision making (Office of Science and Technology Policy Council on Environmental Quality 2022) and in the onramps developed by the Local Knowledge, Traditional Knowledge, Subsistence Taskforce (NPFMC 2023). Managers and researchers should also be willing to address current power and resource disparities that prohibit equitable knowledge sharing and utilization in fisheries management and research.

## 9 Relationships

There is no replacement or shortcut for spending time with people and in place. Reid et al. (2024)

At a co‐stewardship symposium held in Fairbanks, Alaska in 2024, one of the panelists shared that we [researchers] must move at the speed of trust in communities (pers. communication 1/17/24). Federal respondents identified this trust being built through regular, and annual visits with villages where conversations and formal Tribal Consultations occur, in addition to encouraging co‐management and co‐stewardship, both processes grounded in trust and respect (Castleden et al. 2012). For the State of Alaska, the Working Group provides opportunities for increased relationship building, but only if mutual respect is given in this space. Advisory members should be given the autonomy to make recommendations without feeling coerced by ADF&G staff (pers. communication 10/23/23). Currently, state interactions with communities are limited to phone calls, face‐to‐face meetings when possible, and through collaborative research projects. In a co‐management relationship described by Kruse et al. (1998), the key to building relationships is for biologists to spend time in Indigenous communities as opposed to only in co‐management boards. Another barrier to building relationships identified by state respondents, was limitations for biologists and managers to spend time in communities given their other responsibilities and unrealistic expectations to spend time in communities that may not directly be related to a specific research project. There was also the perception that it isn't fair to place that responsibility on biologists who may not have the skills or capacity to spend time in communities for the sole purpose of building relationships. This barrier will be difficult to overcome, and will only hinder true transformation, and the development of meaningful relationships between agency staff and community members. Ultimately, in efforts to build better relations and increase the time agency staff spend in communities, staff capacity, retention and staff responsibilities need to be reevaluated.

## 10 Prioritizing Equity in Fisheries Management and Research

Salmon fisheries and communities in Alaska show increasing trends of inequities, a lack of fairness, in outcomes such as the erosion of rural and Alaska Native resource access, livelihoods, cultural practices, and self‐determination. Donkersloot et al. (2020)

Given the interconnectedness of salmon and well‐being (Donkersloot et al. 2020), and the importance of equity and justice for well‐being (Breslow et al. 2016), our research identified understandings of, and responsibilities to ensuring equity and responsibility by state and federal managers and researchers in salmon management and research. Equity is “the consistent and systematic fair, just, and impartial treatment of individuals, including individuals who belong to underserved communities that have been denied such treatment (NOAA 2022),” whereas equality allows everyone the same opportunities. The distinct differences in the YDNWR and ADF&G missions, in part, attribute to some of the observed differences in how state and federal agencies account for equality or equality in fisheries management and research. For example, for federal staff, there is direct guidance on considering issues of equity in USFWS Director's order number 226 (USFWS 2022a). In the State of Alaska, the state's “equal access clauses” (Sections 3, 15, and 17 of Article 8) “…guarantee equal access to the state's natural resources to all of Alaska's citizens (White 1994),” although equity/fairness is still considered by managers. For example, some state respondents claim that equity is the outcome, and they must consider how to manage equitable distribution of the harvest when making management decisions. Crosman and others (2022) recommend incorporating equity early on in governance to achieve a more just outcome. Another respondent elucidated challenges of centering equity given the inequitable access to technology and data (Mason 2008). While the YDNWR has a unique Government‐to‐Government relationship with Tribes, the ADF&G must allow everyone the opportunity to equally engage in research and management processes, which was clearly highlighted in this research. A potential mechanism for achieving more equity in the state process could be to properly use their Tribal Consultation policy and remove current specified “eliminations”, to allow for more meaningful engagement on management related matters with Tribal governments. An implementation plan for this Tribal Consultation policy in addition to an annual report highlighting key outcomes of the policy implementation are essential for ensuring state accountability to Alaska Native communities in fisheries management.

One example of equity in fisheries management observed along the Kuskokwim River and in the cooperative management structure between KRITFC and the YDNWR is the creation of an additional, fifth inseason manager by KRITFC (2016). They witnessed inequity in their management structure and created a fifth upriver inseason manager seat to ensure adequate representation and a comprehensive understanding of the salmon fishery is reflected in their decision‐making process. This is one example of an adaptive and equitable approach to salmon management. This approach has also in part addressed the issues of downriver–upriver allocation because of the increased representation in salmon inseason management, and a key takeaway for other fisheries governance systems. Our research team also recognizes Tribal leaders and allies as continuing to advocate for equitable salmon management in state and federal management systems (e.g., Board of Fisheries, North Pacific Fishery Management Council) to ensure Tribal voices and knowledges are represented in these systems and advocate for equitable and sustainable fisheries management that prioritizes environmental justice and Indigenous rights (Lavoie et al. 2025). Unfortunately, our research was not able to identify examples beyond what has been highlighted here in terms of mechanisms for inclusion of equity in salmon management and research. For management and research processes to improve, researchers and managers must address questions and issues of equity, power, and justice (Zanotti et al. 2020). Would it be possible to measure the success of salmon management and research systems in terms of social justice, ecological sustainability, economic equity, and cultural diversity (Howitt 2001, 5, 10)? Failure to do so may continue to perpetuate inequities, environmental injustices, traumas, and harms to fisheries systems and Indigenous Peoples across the globe.

## 11 Power Transformations and Restoring Relations

…Ultimately I can just make a decision, justify it, like it or not, take this action or not take action with salmon, and that I think is way too much power for one person when it comes to the cultural importance of something like salmon. It's not just managing a fish and fishery, it's like their whole culture along the Kuskokwim. Federal Respondent 3/6/2023

In efforts to truly see transformation and equity in fisheries management and research, Tribes must not only be treated as sovereign governments with the power to exercise self‐determination, but also carry decision‐making authority. In many cases, final decision‐making authority in resource management is retained by federal, state, provincial, or territorial governments (Mulrennan and Scott 2005; Goetze 2005; White 2006). It was made clear to us in this research that federal and state managers carry the sole delegation (federal) of authority and institutional authority (state) to manage salmon fisheries on the Kuskokwim. Tribal co‐operative management as described in this research suggests Tribes have the opportunity to center Indigenous values in fisheries governance, while also engaging in a Government‐to‐Government relationship where Indigenous Peoples are empowered and guide how their knowledge will be used in a management and research context. McGregor (2009, 75) claims this is the only true way for Indigenous and traditional knowledge to be respectfully “utilized in environmental management.” A unified Tribal approach to fisheries management that enables effective full watershed governance is essential as we move away from colonial regimes rooted in multi‐jurisdictional complexity and inequity. Active community research participation and ownership in management processes and TK studies (Kendrick and Manseau 2008), is an important shift that is needed in transforming the conventional approaches to fisheries management and research.

A state respondent shared, “I would love to empower people in communities…to be able to run their own projects.” This reflects the willingness and desire by agency staff to build Tribal capacity and reflects some of the strengths shared by community knowledge holders regarding local involvement in fisheries research. Some of the positive relationships highlighted in this research reflected positive, long‐standing Tribal‐agency partnerships of various fisheries research projects. We envision the continuation and expansion of these partnerships for the sustainability of fisheries and fisheries‐dependent communities in the future. Enhancing partnerships and expanding networks will help facilitate uplifting and supporting current Tribal capacity. Furthermore, the increased focus of relationships and time spent in community is important as relationships among communities and agencies are restored, and trust is built. Moving beyond relations as transactional and solely associated with salmon productivity should occur in efforts to strengthen relations (Reid et al. 2024). Given the multiple stressors facing Kuskokwim River communities and other fisheries communities around the world, including but not limited to climate change, politics and economics of resource extraction, the rising cost of fuel, difficulty accessing subsistence foods and other challenges, it is critical resource managers, Tribal governments and communities work closely together to achieve shared goals and visions. Through our shared visions of healthy salmon populations, increased fishing opportunities and the continuation of a way of life that have sustained Indigenous Peoples in the region, institutional changes and enhanced Tribal capacity will help achieve a more just, resilient, and sustainable future for salmon and Salmon Peoples.

## Author Contributions

**Janessa Esquible:** conceptualization (equal), funding acquisition (equal), investigation (equal), methodology (equal), project administration (equal), supervision (equal), writing – original draft (lead), writing – review and editing (lead). **Avery Hoffman:** investigation (equal), methodology (supporting), project administration (equal), writing – original draft (supporting), writing – review and editing (supporting). **Destiny Ropati:** investigation (equal), methodology (supporting), writing – original draft (supporting), writing – review and editing (supporting). **Brooke Woods:** funding acquisition (equal), investigation (supporting), methodology (supporting), project administration (supporting), resources (equal), writing – review and editing (supporting). **Jessica Black:** conceptualization (equal), funding acquisition (lead), investigation (lead), methodology (lead), project administration (lead), supervision (equal), writing – original draft (supporting), writing – review and editing (supporting). **Rachel Donkersloot:** conceptualization (equal), funding acquisition (lead), investigation (lead), methodology (lead), project administration (supporting), resources (equal), supervision (equal), writing – original draft (supporting), writing – review and editing (supporting). **Mike Williams:** conceptualization (equal), investigation (equal), methodology (equal), supervision (lead), writing – review and editing (equal). **Wilson Justin:** conceptualization (equal), investigation (equal), methodology (equal), supervision (equal), writing – review and editing (supporting). **Justin Leon:** methodology (supporting), resources (equal), visualization (equal), writing – review and editing (supporting). **Carrie Stevens:** conceptualization (equal), funding acquisition (equal), investigation (equal), methodology (equal), supervision (equal), writing – review and editing (equal). **Craig Chythlook:** writing – review and editing (supporting). **Tazia Wagner:** writing – review and editing (supporting). **Courtney Carothers:** conceptualization (equal), formal analysis (supporting), funding acquisition (lead), investigation (lead), methodology (lead), project administration (lead), supervision (lead), writing – original draft (supporting), writing – review and editing (supporting).

## Conflicts of Interest

The authors declare no conflicts of interest.

## Supporting information


**Appendix 1.** Community Semi‐Directed Interview Questions.

**Data S1:** 4_All Transcripts_Kusko_wTOC.

## Data Availability

The archived community data is available in select participating communities of Bethel, Kongiganak, Aniak, Quinhagak, and McGrath. All anonymous data has been made available internally and only to our team members. All the required data are uploaded as Supporting Information.

## Endnotes

^1^
The amounts reasonably necessary for subsistence, referred to as the “ANS”, is informed by historical harvest data and determined by the Board of Fisheries or Board of Game, whereas subsistence refers to the “harvest and processing of wild resources for food, raw materials, and other traditional uses…”.

^2^
Federally qualified subsistence users are permanent residents of a rural area or community that has a Federally recognized customary and traditional use determination for that resource.

^3^
Indigenous and community knowledge holders refer to all project participants residing in Kuskokwim Region communities; whereas, Indigenous knowledge holders refer to those who self‐identified as Indigenous and/or a Tribal citizen, and community knowledge holders refer to all knowledge holders who reside in community.

^4^
The Tribal Council refers to the governing body of a Tribe and/or a Tribe's legislative body.
