# Supplementary material for: Bridging Knowledge Systems and Perspectives to Inform Salmon Management and Research: A Kuskokwim River Case Study
Source: Ecol Evol. 2025 Oct 13;15(10):e72146. doi: 10.1002/ece3.72146 (PMC12516086; doi:10.1002/ece3.72146)
Supplement: Supplementary file 1 — Appendix 1. Community Semi‐Directed Interview Questions. [file ECE3-15-e72146-s001.docx]

**Appendix 1. Community Semi-Directed Interview Questions**

| **Semi-Directed Interview Questions** |
| --- |
| Please tell us a bit about your background – where are you from, how did you grow up, what connections do you have to salmon? |
| Could you tell us about salmon fishing when you were young. How was it different than today? |
| Were there rules about harvesting? Sharing? How were the rules enforced? |
| Are these teachings/rules still being taught and followed today? Why/why not? |
| How did your ancestors manage/steward/care for salmon? |
| What are the words for caring for land and animals in your language? |
| Do you feel like your knowledge/values are reflected in fisheries management? |
| What worries/concerns do you have about our salmon fisheries today? |
| What are some concerns you have with fisheries management today? In the past? What works well? What does not work well? How would you improve [ ] that is not working well? |
| What do you want to see for salmon and people in your region in 40 years? |

**Appendix 2. Agency Semi-Directed Interview Questions**

| **Category** | **Question** |
| --- | --- |
| Introduction | Can you please tell us a bit about yourself, where and how did you grow up and what are your connections to salmon? |
| Introduction | Can you briefly describe your position at (list agency/professional organization)? |
| Perspectives on Fisheries Management | Generally, how well do you think management is working for salmon in the region you work? For people of this region? |
| Perspectives on Fisheries Management | What do you think are the primary strengths and weaknesses of salmon management in Alaska today? |
| Knowledge Systems & Values | What do you want to see for salmon and communities in the next 40 years where you work? |
| Knowledge Systems & Values | What are your understandings of the values Indigenous peoples place on salmon in the region that you work? |
| Knowledge Systems & Values | Do you think your agency management system reflects the values of Indigenous fishermen? If so, can you please provide examples? If not, why not? |
| Knowledge Systems & Values | What is your understanding of Indigenous knowledge? How does it compare to Western Science? |
| Knowledge Systems & Values | What role do these different knowledge systems play in fisheries research and management in the _________ region from your perspective? |
| Inclusion | How does your agency currently include Alaska Native peoples, their knowledge, values and governance systems in salmon management and research? |
| Inclusion | Are Alaska Native knowledge and Tribal governments adequately represented in large decision-making forums and processes? |
| Inclusion | Do you see a need for more inclusion? If so, how might your agency build more inclusivity for the future? |
| Inclusion | What ways can Alaska Native peoples, their knowledge, values, and governance systems be better included in the current management and research processes? |
| Inclusion | What is your understanding of equity and equality in fisheries? |

**Appendix 2. Agency Semi-directed Interview Questions**

| **Category** | **Question** |
| --- | --- |
| Inclusion | What is your agency’s responsibility to provide for equity vs. equality or both? |
| Relationships | How would you characterize the working relationship between tribes and your agency? |
| Relationships | What value do you or your agency place in building relationships with the communities you serve? |
| Relationships | Is there support (time/staff/budget) from your agency to build relationships with fishing communities? If so, what does this look like? |
| Relationships | Can you please describe any barriers and opportunities to spending time in communities and building relationships with communities you serve? |
| Relationships | The ISM project has identified a lack of transparency, poor communication, and inconsistent approaches between different management entities as weaknesses of current salmon management systems. Do you also see these as problems? Do you identify other issues? Do you have any solutions for addressing these issues? |
| Closing | Is there anything else you would like to share with me that hasn’t been covered yet? |

**Appendix 3. Agency Results Summary**

**Indigenizing Salmon Science & Management Project: Kuskokwim Region**NSF # 1936378; UAF IRB Approval Number: 1146180-2


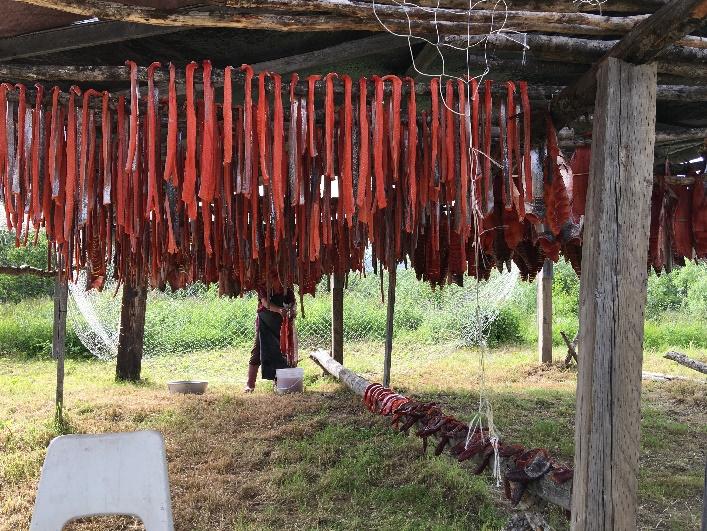


Bethel Area Fish Camp Drying Rack

**Guiding Project Goals and Objectives**

The overall project goal is to document the breadth and depth of Indigenous knowledge, governance, and value systems pertaining to salmon across Alaska. The first component of this project in the Kuskokwim Region involved working with Tribal governments and Indigenous organizations to refine project scope and conduct semi-directed interviews in Kongiganak, Quinhagak, Bethel, Aniak, and McGrath with 45 knowledge holders. These results will soon be published in the *Arctic Science* journal and will be freely accessible at (https://cdnsciencepub.com/journal/as). Based on the responses we received from knowledge holders, we created a semi-directed interview guide to administer to Alaska Department of Fish and Game Commercial Fisheries Division and Yukon Delta National Wildlife Refuge staff. Staff engaged in this research include researchers, managers, and other staff who have or are currently working with and serving the Kuskokwim Region salmon fisheries and communities.

Project objectives for this research project include the following:

**Objective 1.** Identity strategies to improve the current salmon management system from State, Federal, and Tribal (not reflected in this summary) perspectives.

**Objective 2a.** Document State, Federal, and Tribal (not reflected in this summary) perspectives of Alaska Native inclusion in current management systems.

**Objective 2b.** Determine how Alaska Native values, knowledge, management, and governance mechanisms can be better included into current management systems.

**Objective 3.** Identify key opportunities and barriers to relationship building between Alaska Native communities and State and Federal agencies.

**Objective 4.** Identify mechanisms for how equity or equality are included in agency research and management systems.

**Executive Summary**

All respondents were or are currently employed as researchers, biologists, coordinators, managers, or refuge informational technicians in the Kuskokwim Region. The majority of respondents from the Refuge self-identified as Alaska Native and as living in the Kuskokwim Region year-round. None of the state respondents self-identified as Indigenous and all live outside of the Kuskokwim the majority of the year. Perceptions of how well salmon management is working for the salmon and communities within the Kuskokwim Region varied. The majority of federal respondents reflected that management was working well, while state respondents were fairly mixed. The positive reflections regarding salmon management shared by federal respondents were centered around their cooperative management partnership with the Kuskokwim River Inter-Tribal Fish Commission (KRITFC). The majority of federal respondents stated that Alaska Native values were included in federal management and research systems and processes, which was primarily attributed to the large percentage (over 50%) of Alaska Native staff employed at the Refuge, the extensive formal and informal Tribal Consultations facilitated with communities year-round, their working relationship with the KRITFC, in addition to time staff spend in communities. The majority of state respondents regard the inclusion of Alaska Native values in state management and research systems and processes as mixed, with some respondents declining to answer the question.

Federal and state responses regarding Alaska Native inclusion in broader decision-making forums such as the Alaska Board of Fisheries (BOF) and Regional Advisory Councils (ACs) were fairly mixed. Distinct differences were observed regarding the roles of Indigenous knowledge systems in state and federal research and management processes. While all respondents saw value in being able to listen to information from Indigenous, Traditional and Traditional Ecological, and local knowledge holders alongside western science, federal respondents provided more clarity and specific examples on how this is done, whereas state responses showed ambiguity and conflicting assessments about how and whether this can legally be carried out by biologists and managers.

All respondents stated that spending time in Kuskokwim Region communities and building relationships was important for their job. However, the scale of community engagement and relationship building varied among federal and state agencies. Many of the federal respondents regularly spent time in communities and built relationships, whereas state respondents engaged more so through public processes, management meetings, and during project-specific community visits. There appeared to be more support, flexibility in job description, and emphasis for employees to engage in this type of relationship building at the Refuge. However, more Tribal and community collaboration for research projects were identified by state respondents. Lastly, all federal respondents were aware of and carried out formal and informal Tribal Consultation with Kuskokwim Region communities regularly. None of the state respondents were aware of Tribal Consultation policies at the Department of Fish and Game. Lastly, understandings of equity and equality varied across both agencies. Some respondents viewed the two concepts as the same, whereas others were not sure how these concepts related to their daily work. Respondents also provided some examples of how equity or equality are considered in research and management.

***Personal Information and Time in the Kuskokwim Region***

In total, 12 respondents were interviewed for this component of the study. Four of these self-identified as Indigenous (*all Refuge employees)*.

Home communities of respondents included Anchorage, Bethel, and nearby communities (*not included for anonymity purposes).* Professions ranged from researchers, research and management coordinators, managers, biologists, and refuge informational technicians.

All federal respondents reported living in the Kuskokwim Region year-round, whereas state respondents reported to have spent on average about 2 months in the Kuskokwim Region annually.


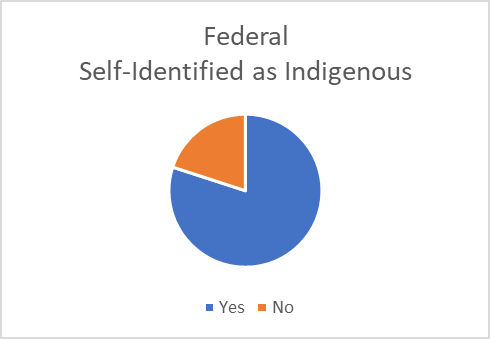

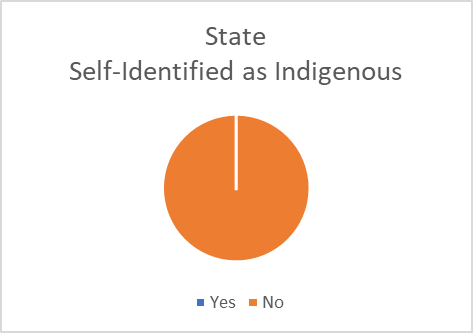


***Salmon Management***

Six respondents (*80% of federal employees, 29% of state employees)* stated that management was working fairly well for the salmon and the communities in the Kuskokwim Region. Three respondents stated management is not working well (state respondents), two respondents (state and federal) had mixed responses, and one respondent’s (state) comments on this question were ambiguous; this respondent didn’t want to place a value judgment on how well the salmon management system was or was not working.

*Yukon Delta National Wildlife Refuge (Refuge)*

*“There’s no other people other than local people in this area that are gonna care about salmon more than they do.” –* Refuge Respondent

Strengths identified by Refuge respondents included: good relations with local people and the State of Alaska, regular communication and Tribal consultation with villages, cooperative management structure with the Kuskokwim River Inter-Tribal Fish Commission (KRITFC), improved relations between the inseason manager, Alaska Native employees, and local communities, utilization of traditional knowledge and inseason harvest data in fisheries management. The strengths identified by respondents centered around their partnership with KRITFC, the federal salmon management team (primarily composed of Alaska Native fishermen) and how this cooperative management structure has allowed for increased trust by local people and success in conservation efforts.

Weaknesses and challenges identified by Refuge respondents included: the politics associated with salmon management and end result of local subsistence users being in the middle of these issues, the Alaska BOF system causing trust to erode (for example, perceptions of different treatment of user groups and regions as evident at the Alaska Peninsula/Aleutian Islands/Chignik finfish meeting in February 2023), the mismanagement of the Area M and trawl fishery, inadequate staff and capacity to carry out research and management activities, disconnect between the regional Fish and Wildlife Service office and the Bethel Refuge office, important decisions impacting federal subsistence users are made by individuals who do not live in the region or rarely or never visit the region, inadequate science and management conducted outside of the Refuge boundaries, and inability to fully serve subsistence users outside of the Refuge boundaries.

*Alaska Department of Fish and Game (ADF&G)*

*“There’s no salmon research that has occurred within the Kuskokwim that hasn’t been collaborative in some way, shape, or form.” –* ADF&G Respondent

Strengths identified by ADF&G respondents included: engagement with local users, the public process (e.g., Working Group), public input on how to run assessment projects, development and evolution of robust research and assessment programs, establishment and building of local partnerships to carry out research projects, escapement goal policy, adaptive management strategies, sustainable fisheries, and improved communication and information dissemination with the establishment of the KRITFC.

Weaknesses and challenges identified by state respondents included the following: inability to make timely decisions given the large number of groups involved in management, strained management relationships given the current lawsuit between the State of Alaska and the federal government, dual management, push for federal management given the limited resources they have and resulting misinformation, confusion, and missed subsistence opportunities, receiving and balancing the conflicting traditional knowledge and desires of local fishers, inability for management to fully control how many fish come back (e.g., survival of salmon in the ocean is beyond managers control), erosion of working relationships, and funding constraints which “limits our ability to be proactive”, and avoid potential future fisheries issues.

**Alaska Native Inclusion in State and Federal Research and Management Processes**

*Federal*

*“The best way for this to happen is to have Alaska Native people actually working in these different positions at the Refuge.” –* Federal Respondent

The majority (60%) of respondents felt that Alaska Native values were included in federal management and research systems and processes, and the remaining respondents (40%) had mixed responses. It was made clear that this shift in seeing Alaska Native values reflected in federal management and research systems occurred recently, and through the formation of the salmon management team that is primarily composed of Alaska Native Refuge staff who “live and breathe fishing.” The ways in which Alaska Native values are included in federal management and research processes is through hiring Alaska Native staff, working with Tribal governments, spending time in communities, and working with the KRITFC. Most respondents felt that there could be more ways to reflect Alaska Native values in their research and management processes, including improving relationships with Indigenous Peoples along the river and seeing more Alaska Native Peoples in the “hot seat” (i.e., having decision-making authority).

*State*

*“That's the foundation of what we do is providing for the future." –* State Respondent

The majority of responses regarding the inclusion of Alaska Native values in state management and research systems and processes were mixed (57%). Others (29%) were unable to answer the question or responded no (14%). Much of how Alaska Native values are reflected in state management and research systems and processes were tied to the State’s responsibility to ensure healthy, sustainable salmon runs for future generations to come, and considering the needs of the whole river, not just one community. Sustainability and considering the needs of others along the River were both viewed as Alaska Native values which are embedded in the state management and research systems. Respondents who were not comfortable answering the questions were largely due to the fact that they did not want to speak for Alaska Native Peoples, and thus it’s not their information to share. One respondent felt that the state has their own guiding Western principles centered around sustainability that are not in alignment with Alaska Native values.

**Adequate Inclusion of Alaska Native knowledges’ and Tribal Governments in Decision-Making Forums (eg., BOF, Regional AC, etc.)**

*Federal*

The majority of responses (60%) were mixed regarding inclusion of Alaska Native knowledges’ and Tribal governments in larger decision-making forums. They felt there was fairly adequate inclusion in the cooperative management structure between the Refuge and the KRITFC and in the Yukon-Delta Regional Advisory Council, but not at the BOF or FSB. Despite the cooperative management structure between the Refuge and KRITFC, respondents identified ample opportunities to improve communication and relationships with more fishers from the Kuskokwim Region.

*State*

Respondents (43%) felt that Alaska Native knowledges’ and Tribal governments were adequately represented in larger decision-making forums, while others (43%) could not answer the question, and one response (14%) was mixed. While the state doesn’t seek out individual community involvement in the process, communities along the Kuskokwim River are notified of when public meetings like the BOF and state Advisory Committees (ACs) are occurring. Both processes present many opportunities to interact and engage in decision-making. The BOF process was mentioned as a process that can be intimidating, and require initiative, drive, and motivation to participate in the process. Of the respondents who could not answer the question, it was largely due to the fact that they did not know whether Alaska Native’s were satisfied with the current level of engagement in these decision-making forums and questioned whether the level of engagement was adequate, thus unsure of how to answer the question.

**Roles of Indigenous Knowledge Systems and Western Science in Research and Management Processes**

*Federal*

*“Everything we do involves Indigenous knowledge.” –* Federal Respondent

The majority (80%) of respondents felt Indigenous knowledge and Western science both guide salmon management decisions, while one respondent felt Western science still dominates salmon management decisions, and claimed Traditional Knowledge (TK) is viewed as “anecdotal at best'' by higher level administrative staff and outside entities, and feels numbers mean more than words, especially in court. In terms of inclusion of Indigenous Knowledge in salmon research and management, the guidance for doing so has been made very clear by the Biden Administration and secretarial orders given to the Refuge. Currently, the cooperative management structure with KRITFC allows for local Alaska Native fishers to be directly engaged with and guiding salmon decision-making processes. The cooperative management agenda has an item specifically for Traditional Knowledge. Ways in which Indigenous knowledge is included at the Refuge includes hiring local people through the local hiring authority (over 50% of Refuge staff are Alaska Native), the RIT program, regular Tribal consultations and their partnership with the KRITFC. The majority of respondents see both Western science and TK as bodies of knowledge that directly guide research and management processes at the Refuge. However, this was perceived by some to be at risk of changing depending on who is in the lead management position.

*State*

*“As a manager you want to take in all available information. The more informed you are, the better decision you can make.” –* State Respondent

All respondents saw value in being able to listen to information from Indigenous, Traditional, Traditional Ecological and local knowledge holders, however, the responses spanned a very large range in terms of how these knowledge systems actually guide research and management by their agency. While respondents want to allow for both bodies of knowledge to guide management, several respondents weren’t sure exactly how to do this and are looking for guidance. It was very clear that “hard data”, or western scientific quantitative data, regularly guides management and research processes. Examples of inclusion of Indigenous and Traditional knowledge included guiding new research efforts like weir placements, using these bodies of knowledge to “ground truth” assessment project data, through listening to knowledge holders at public meetings, learning how to fish, use boats and repair nets from locals, and leveraging IK with Western scientific data to make a decision. One respondent did share that their decisions and actions have to be “defensible” and spoke to “legal ramifications” of only relying on Indigenous knowledge in salmon management. Another respondent claimed that most staff do not have the authority to incorporate TK into their work, and thus, this would likely need to be done in the form of a co-management agreement, which does not currently exist. ADF&G’s Subsistence Division was identified as the entity which carries out much of the work that documents Indigenous and Traditional knowledge and other “anecdotes”, but may not translate to actionable information for the Commercial Fisheries Division. Another respondent claimed other bodies of knowledge can help them when thinking about how this information may fit into their western scientific understanding of what’s going on with the fishery.

**Opportunities and Barriers for Relationship Building in the Kuskokwim Region**

All respondents felt their agency places great value on building relationships and spending time in community in some capacity.

*Federal*

*“The sooner we put more emphasis on relationships building and rebuilding, the sooner positive things will happen.” –* Federal Respondent

**Opportunities**

Opportunities for improving and building relationships and spending time in the community include the following: continuing to visit, be present, hang and talk with communities, Elders, youth, Tribal Councils, hiring more Refuge Information Technicians (RITs), encouraging more co-management and co-stewardship of resources, providing more funding for Tribal entities like the KRITFC, and having staff continuity and retention needed to continue building trust and relationships with communities.

**Barriers**

The barriers for improving and building relationships and spending time in the community include the following: the low pay for RITs, time limitation due to duration and difficulties in visiting all villages, manager turnover, limited staff, language barriers, past exclusion of Alaska Native Peoples from management and other decision-making processes, and the fact that the lead manager has too much power and control. “One person can make drastic changes.” Thus, if relationship building is not a priority to the lead manager, this individual can and has led to poor relations between the Refuge and communities as a result of time and energy devoted or not devoted to relationships.

*State*

*"If we want to be successful and build those relationships I think keeping those lines of communication open and working to build them is an important component." – State Respondent*

**Opportunities**

One respondent claimed good working relationships are central to the State of Alaska’s mission. Opportunities for improving and building relationships and spending time in the community include the following: pre-season and post-season Kuskokwim River Salmon Management Working Group meetings, phone calls between management and community members, salmon in the classroom activities, attending other community events (e.g., 4th of July fairs), collaborative research projects, future community visits, and spending time and building friendships with Alaska Native staff on assessment projects and when in local hubs like Bethel. Opportunities for improving and building relationships and spending time with communities were also understood as being carried out by the Alaska Department of Fish and Game Subsistence Division historically, currently, and in the future.

**Barriers**

The barriers for improving and building relationships and spending time in the community include the following: less capacity in terms of funding, staff, and flexibility in travel planning, closure of commercial fishing operations because time spent in community often equates to a particular project or fishery operation, timing conflicts of when community members and managers are available, too many management meetings and a vast area to cover in terms of traveling to communities, hostility faced in communities, and needing to have an agenda to travel to a community. One respondent didn’t feel that the Commercial Fisheries Division emphasizes these interactions much, while another respondent didn’t think it was fair for the average employee to have to carry the responsibility of all of the relationship building and emphasized the importance of this needing to be a two-way street between the agency and community. There are limitations to Biologist positions doing this type of work in addition to lack of authority that particular staff have to be able to engage with and build relationships with Tribal Council leadership. The average fisheries biologist often doesn’t have the capacity or time to spend in communities, “just for the sake of developing relationships.”

**Tribal Consultation Policies**

We asked many of the respondents whether their agency had Tribal consultation policies. All Federal respondents regularly engage in formal and informal Tribal consultation year-round, and sometimes several times a week with villages within the Yukon Delta National Wildlife Refuge. None of the state respondents who answered this question were aware of any Tribal consultation policies the state has in place. One respondent thought that someone working in the Commissioner’s office would know this information.

**Challenges, Solutions and Further Questions**

The community portion of this project identified several weaknesses of salmon management. Some of these weaknesses included lack of transparency, poor communication, and inconsistent approaches between different management entities. Responses to these weaknesses identified by agency staff are described below.

*Federal*

The primary reason for closed door salmon management meetings between the Refuge and KRITFC was due to the fact that this was the request of KRITFC, and it is their decision to determine how Tribal consultation will be carried out, and thus the Refuge is respecting this request. While several respondents said it is important to be transparent, one individual has viewed the benefits of closed-door meetings, one of which allows people who particularly don’t speak up to do so in smaller meeting spaces. Interested community members are encouraged to reach out to the Refuge or KRITFC if they’d like to be involved in future meetings, and they have accommodated such requests in the past. Regarding communication, while this is perceived as a challenge for some, their solutions for addressing this was to more frequently visit villages with KRITFC and explain their management decision-making process while also gathering feedback on how this process can be improved. Regarding communication, the Refuge alongside KRITFC is trying to disseminate information regarding fishing opportunities as soon as possible. One other comment that was made was the fact that the head inseason manager has far too much power, and is wondering why the delegation of authority letter couldn’t delegate responsibility or authority of the river to the Refuge, KRITFC, and the State of Alaska, which could potentially help avoid conflicting management strategies and mixed messaging on fishing opportunities.

*State*

Regarding lack of transparency, respondents felt fairly confident that their agency is fully transparent in all that they do. Several respondents were open to discussing ways to increase transparency and do a better job. One respondent felt the transparency issues were a “narrative” or “conspiracy theory” created to undercut the state and create tension between the state and communities. In response to poor communication, a respondent shared, “we’re always thinking about how we can get information out to the public.” Mass communication can be difficult to overcome in terms of making improvements, but respondents are open to mechanisms for improving communication and question how communities currently receive management and research related information. Regarding inconsistent approaches between state and federal management entities, the majority of respondents also viewed this as an issue. One respondent claimed “That was one of the things that was really troubling.” This issue causes great confusion and is unfortunate for everyone on the river. Another state respondent claimed much of their direction comes from the BOF regarding what they can and cannot do, and felt their team also listens to the Kuskokwim River Salmon Management Working Group 95% of the time. One solution for inconsistent management approaches is to have a more unified approach among management entities as opposed to dual management. *For More Information or Questions Please Contact, Janessa Esquible. Phone: 907-406-0040 Email: jaesquible@alaska.edu*
