# Supplementary material for: Bridging Knowledge Systems and Perspectives to Inform Salmon Management and Research: A Kuskokwim River Case Study
Source: Ecol Evol. 2025 Oct 13;15(10):e72146. doi: 10.1002/ece3.72146 (PMC12516086; doi:10.1002/ece3.72146)
Supplement: Supplementary file 2 — Data S1: 4_All Transcripts_Kusko_wTOC. [file ECE3-15-e72146-s002.pdf]

TABLE OF CONTENTS

Interview participant: Alexie Nicholai 2
Interview participant: Megan Leary 13
Interview Participant: Nancy Simeon-Morgan and Betty Simeon 27
Interview Participant: Ana Hoffman, Avery Hoffman, Stanley Hoffman Jr. and Stanley Hoffman Sr. 39
Interview Participant: Robert Lekander 60
Interview Participant: Mary Peltola, Matthew Kapsner 73
Interview Participant: Elias Venes, Elizabeth Hoffman, Connie Sankwich, Darrell and Ruth Garrison 92
Interview Participant: Henry Kohl 114
Interview Participant: Evon Waska 140
Interview Participant: Theodore ‘Tad’ Lindley, Elizabeth ‘Mik’aq’ Lindley, Elijah ‘Pinchboy’ Lindley 151
Interview Participant: David David, Rhonda Kanuk 166
Interview Participant: Nicholas David 173
Interview Participant: Pauline and Jonathan Egrass 181
Interview Participant: Nestor and Naomi Norback 193
Interview Participant: Helen Evan 207
Interview Participant: Arnold Andrews, Dara and Kevin Whitworth 212
Interview Participant: Nicholas Snow 232
Interview Participant: Matilda Evans 249
Interview Participant: Joshua Cleveland 255
Interview Participant: Dorothy Mark 262
Interview Participant: Lucille Mark 268
Interview Participant: Stanley Berlin 273

INTERVIEW PARTICIPANT: ALEXIE NICHOLAI

Interview Date: 2/6/20
Interviewer: Janessa Esquible, Janelle Carl
Location: Oscarville
Home community: Oscarville

Shorthand key:
( ) Note about what is happening during the interview, unclear of word, or spelling question
[ ] Words not said but added for clarity
{ } Action
Mmm-hmm, uh-huh are affirmative
Hmm-mmm, uh-uh are negative

Janessa Esquible (JE): Today is feb 6, and we are in Oscarville along the Kuskokwim. I'm Janessa Esquible and this is Janelle Carl. We are with Jimmy Larson Jr. and Alexie Nicholai. Jimmy Larson will be is the grandson of fAlexie and he will also be helping to interpret, and Janell carl will be doing most of the interview.

Well its not for sure the goal of this project is to get indigenous (inaudible) Yupik perspective and knowledge, wisdom, values pertaining to salmon and relaying that information over to, you know, the local organizations like fish and wildlife, fish and game, other tribes and trying to improve salmon management with, be it like, with the help of locals who really understand the Kuskokwim and the fish better than anyone else.

Jimmy Larson (JL): you probably understand,

Alexie Nicholai (AN): These fish are taken care of, by you know, by people because it is for their food. They try not to let the houseflies lay eggs and prepare it the best way for consumption. But when they are not properly taken care of, the houseflies lay their eggs and hatch into larvae and are not fit for consumption. There are many different facets of working on the fish to prepare for consumption.

And these trees are all not fit for use to cure the fish with, but we specifically use cottonwood. Now the cottonwood are the best in deterring the houseflies from approaching the fish.

When they first hang the slabs onto the drying racks, they place a fire with cottonwood as the wood source to keep burning to keep the flies from landing on the fish. Now that's the best way to dry your salmon.

Once they are dry, they are brought into the smokehouse and cold smoked to cure. The fish need to be cold smoked because if you burn them with hot smoke; that also destroys the fish quality. But light your smoke away from the fish and use the cottonwood to cure the fish and don't hold back on the smoke.

JL: You have fished the Kuskokwim River for a long time now, and being stewards of the Kuskokwim is also an important aspect and also to keep an eye on the seasons. When it is favorable, the wind…ahh what is it…what is…what is the word for cottonwood again? They prefer cottonwood than any other tree because they're repellant for the bugs that they don't make them bad.

Now considering that, the management purpose; can you say a few words regarding that?

AN: Of what?

JL: You know, now they are opening the fishing season late in the season, can you address that for a bit?

AN: Yea, the restrictions that were put in place are not conducive for the proper drying of the fish. Here on the Kuskokwim, they only fish when the tide is coming in. When it is low tide, there are many snags in the river that your net gets hung up on and tears it apart, therefore making low tide not a suitable for fishing. When the tide comes in, they fish and it is a good period. Those that are trying to procure salmon, they should not be subjected to time periods. These fish and wildlife announce specific times for fishing. They are not commercial fisherman; they are subsistence fishers for themselves.

JL: Can you stop this for a second. Can you stop this. So we will go over this. This is the first subject she wants you to cover first. This,

JE: Oh yea this has to be recorded to…there are no risks of this study, sometimes the topic gets emotional, but you don't have to say anything that you are uncomfortable with. The compensation that you would receive 75 and he would receive 100 in a check and its confidential, it is voluntary if you have any questions let us know, our contact information is all here and then this is, its later when we are summarizing the results from the communities up and down the Kuskokwim, if you want us to use your name to to thank you ,ehh you said we can record you, you want us to be archived so people can access it at the library and then if you want you can (inaudible).

JL: This first statement…they can use your name when they do a report or summary using your name.

AN: Even if you don't use my name, the people know the way of procuring and processing salmon. All of it.

JL: ok

JL: Yes spell it out and then like an Englishman, like when you sign a check, sign your

name.

(skipping the mailing address, and such)

JL: I would like you to share everything that you know, you know you do remark on some seasons, wondering why the fish or such are not showing up, some of the other fish, and some species do not show as they used too..and give the time and date which you already mentioned that the allotted time for fishing is not conducive for drying salmon.

Also, please address the timing of the fish that enter the kuskokwim, the first pulse and then the second pulse, and also include what you mentioned regarding the fish size restriction, what your thoughts are on that, that would be good. I am just telling you those, and furthermore, the subsistence fisherman, specifically if they do not upkeep care for the land and river, and please share that. Those are the three areas that I have mentioned. Also you mentioned trawler , you know you keep addressing the trawlers in the high seas, and how that has affected the fish. Please also mentioned that. Can you start by pretending to boat up the kuskokwim and then get to oscarville, and how do we care for the fish using our ways and also mention the dog teams of the past. The dog team is not popular anymore, and we eat the fish, the king salmon, we need to care for them and the specific number of salmon we used to catch and what is the cease point of fishing for salmon. Please mention that if you want as well, and then also the year-round effort of trying to catch fish, not by dipnets. And putting nets under the ice throughout the winter and summer, please also mention that.

AN: The salmon sometimes do not come all at once even if the first pulse is here. They are dependent on the weather. Sometimes even if the weather is favorable then there is a great number of fish that come in as a pulse.

The first fish that come in are the smelts. Those are caught with dipnets. There was talk of using dipnets to catch salmon here, but that method is not used to catch salmon with. If the rivers are narrow, then you can deploy the dip nets, or with narrow rivers you can erect a fence and use dipnets. Yhe suitable rivers to use dip nets have only one direction in which it flows, that are not affected by the incoming tides.

Janelle Carl (JC): I am going to ask you some questions. Where are you from?
How did you grow up? How do you connect to salmon?

AN: because it is our food, we work on them, because as people, we vigilantly work on them, and when they are properly cured, we store them for future use

JL: What is your name?

AN: Alexie Nicholai

JL: In Yupik?

AN: Uqsuungaq

JL: How does the salmon affect your way of life, how intimately is the salmon connected to your life?

AN: Well, all the people go and see the one who catches the most fish and check out his gear, and imitates his gear and ways and go out and fish for themselves.

Only some people, if a net is not properly hung, they do not catch as much. But others have nets that attract fish, and so they go to the successful fisher's gear and copies what it is like and they are able to catch more fish for themselves afterwards.

JL: You have grown up, gotten married, raised children and fishing is very important to you, how was it when there was store, you went and got food, but this salmon needs to be vigilantly cared for, in your opinion, what are your thoughts on that?

AN: This is the way of the store. When the weather and the climate is bad and you cannot harvest fish and that is why the store is very important for the people, it is a necessity. The processed food does not disappear when the weather is bad. But if the weather is bad, then the store runs low on its goods to sell.

JL: That is why the people were vigilant about procuring and processing fish for consumption. How was the food processed? Begin from tip to head.

AN: Many of the buckets and barrels were not readily available but the grass was abundant, and they braided the grass into bags and pouches and the finished product (salmon) would be placed into these bags.
JL: and now the use of the salmon, the stomach, the roe, the head and what else. Please mention the use of those.

AN: These food parts, such as the head, with some of the guts added, but discard the white parts of the guts and others and place the heads into a pit with the guts into the earth.

But the roe, they would place them on wood to dry and would be placed into the ground. It was said that dried products placed into the ground do not form mold or mildew and do not spoil and the shelf life of such is five years.

The people would take the skin of the king salmon and make them into sacks for the roe to be placed in, and they would place them into underground cellars. These would never spoil and even in five years the food is still edible.

And when they are going to prepare red berries, they would take this cached roe and add them to the berries.

JL: How come they like the salmon roe? Explain winter roe.

AN: The people would eat those as well, they are edible just by themselves

JL: Also in the cold winter they are eaten and warms the body.

AN: They satisfy your hunger and keeps your strength for a long time. And in the springtime, when the pike arrive, the pike deposit their eggs and when person used a bow and arrow to catch a pike, they would take the pike and squeeze the roe into their mouth as food. And that also fills your belly and staves of hunger for long time.

JL: What fish, in your opinion, is paramount in its use for or by people for sustenance?

AN: The white fish is paramount, it can be eaten raw, the innards can also be eaten raw as well as the pikes.

JL: King Salmon?

AN: The King Salmon has to be dried to be eaten.

JL: Could you expound on the uses of the King Salmon, how it is utilized, of all the fish you know, would you please address that first, the blackfish, no what is the name for it? what are the names of the smelts? Could you address, by season starting from the blackfish and other fish that migrate through the waters, those fish the come in the river, starting from smelts, what are steps/process of those?

AN: These blackfish stay in the lakes, and they stay put in the warmer temperatures, and when the weather gets cold, then they start swimming out of the lakes and traps then can start catching them.

JL: Michael, am i doing the right fish? what are smelts called in yupik again?

AN: Also in the upper areas, when there is no fish, when the weather gets cold, that is when the whitefish starts migrating out of the lakes, and can be caught with dipnets. including the pike, lush and the sheefish. Those are the fish that migrate out of lakes when the weather gets cold.

JL: After that, would you pretend you are a fish coming into the river, why do they follow those smelts?

AN: The fish have their seasons and routines that they follow.

JL: Why are those important for you?

AN: It is because it is a food source of people. Remember I told you earlier that the grocery stores, when there is no delivery, the food selection dwindles, but these fish that are dried do not dwindle.

JL: So after the smelts come up the river, what follows the smelts?

AN: The pike are the last ones that go up the river.

JL: what else? what is the procession? King Salmon, Silver Salmon?

A: King Salmon, Dog Salmon, Silver Salmon, those three come in tandem.

JL: Those species that you just mentioned, can you address their importance and use? How they benefit the people, that we prize for eating.

AN: The King salmon are big, so they provide a good amount of food, and they have a high oil content so one should not eat too much of it, so that is why the taking of king salmon are limited. On the other hand, the chum salmon are caught in abundance, because they were also used to feed dogs. The silvers are also not taken in abundance, because it is hard to dry them when the weather gets cool.

JL: Depending on season, and this one, these two are least, least favorable for drying and this is, and this fish is mostly for chums.

AN: I don't have anymore to say regarding those.

JC: When you were a small kid, how did you fish for salmon with nets? and as you grew up?

AN: The young boys follow in the fishing endeavors to observe and learn, and once they learn they can fish by themselves, all these things that need to be worked on, they learn from others.

JC: How has it changed, since a long time ago?

AN: I'm not really sure, because it is hardly cold out there anymore and the level of the water has dropped. The water comes down from the mountain range when there used to be a lot of snow in the mountains. Now there is not that much snow, and it is affecting the salmon which has forced them to change the timing of their runs. The water is used as a medium for their transport.

JL: That could be under rules

JC: ok

AN: Furthermore, when there used to be high water, the salmon used to migrate further up the rivers and creeks, and the brown bear would have food available to them, and

the black bears as well. But now, because of low water, the salmon do not go up to their spawning grounds, that is why we are seeing more brown and black bears in this area.

JL: That is a definite change from how it used to be. Now there are black bears mostly in this area getting fish, rather than where they would normally hunt their fish up closer to the mountains.

An: Even though the fish get here, they no longer go high up to the mountains because the water is too low.

JC: Before Fish and Wildlife and Fish and Game, how were the seasons or fish managed?

AN: They knew the season of when a specific species would be available. The people are intelligent.

JL: Calendar, seasonally so they, the people relied on most of those things.
JC: How did they know when the fish run was going to be good?

AN: I don't know about that, they probably looked at the water, the weather and knew what was coming. When there is a south wind, the fish run is quite thick.

JL: Did your parents stay at one place, or were they nomadic?

AN: They would move around and hunt. In the spring, they would hunt muskrats, and in the fall, they would also hunt and fish for blackfish. The blackfish were plentiful in the tundra. The lakes used to fill with water when there was a lot of snow in this area.

JE: He feels like his knowledge and wisdom is reflected in management, the way they manage the fish today?

AN: The Fish and Wildlife opens opportunity at the most inopportune time. The people use the tides to fish. At low tide, there are a lot of snags that tears up the nets.

JE: Does he think things are going right, the way the fish are being managed or what's going wrong, and how can he improve things.

AN: Well I just said it earlier, here on the Kuskokwim, we fish when the tide is coming in. The snags are not as prominent when it is high tide. At low tide, when you hit a snag, it tears up the nets. They are given specific times to fish. The subsistence fisherman should not be given a specific time to fish. The subsistence fisherman goes out when the tide is coming in for their food. The timing put on the fisherman is detrimental to the nets. Snags tear apart the nets, but when they fish with the incoming tide, they avoid the snags and furthermore, when you are trying to deliver your fish to fish camp at low tide, you get stuck in the mud. It is far easier to deliver fish at high tide.

JL: Can you add to how the management is going, how it is not conducive to proper care and drying, and include how the fishing is open during when the weather is not optimum for drying fish? How one has to work harder not to waste fish.

AN: especially when the weather is wet and humid, the fish cannot dry.
JL: You also mentioned dip netting how you have never utilized it.

AN Dipnets are not useful on a river like this, only when the current is strong, especially right after the ice has gone out, that is why the smelts run right along the river bank out of the swift current.

JL: Can you also mention the state of the river now, how the river is changing?

AN: Because there has not been any major snowfall, the turbidity of the water has changed. When there has been high water in the meadows, here and up along the river, the water runs off from the meadows and changes the turbidity of the water. When you put a net out to catch fish, the net is not visible to the fish. But when there has not been any runoffs from the meadows, the level of water drops and the water becomes clear. That is why the fish swim, avoiding the nets when the water is clear.

JL: Would you also mention the use and size of the mesh on nets, on how the restriction of mesh sizes affect fishing, in terms of the positive and negative aspects of it?

AN: The people look at their needs and work to accomplish their work to address those needs. When the weather is going to be bad, they limit the catch of fish, but when the weather is going to be good, they gather what they need to adequately dry the fish.

JL: You know now at this time and age, what is mesh in Yup’ik, what about the mesh size mandated now on fishing gear?

AN: The mesh sizes are different. When the fish are too big for the mesh sizes they do not get entangled. But for the king salmon, the 8 inch and 8 a quarter mesh sizes catch the bigger kings.

JL: Please mention the size differences of the kings that are now running.

AN: The small mesh sizes just catch the smaller fish and cannot catch the bigger ones, and the small fish escape the fish nets.

JL: When the fish reach the spawning grounds they have good growth, perhaps with the smaller mesh sizes, this is affecting the size growth of the salmon.

AN: They cannot gain weight and put on the necessary fat content, because I keep saying, that the factory trawlers are decimating the sea floor, damaging the food source of the fish. The seals are also affected with food poisoning and contributing to their deaths, because they are eating food that they have not typically consumed. Their traditional food has been decimated and because of their hunger, they are eating anything and everything to try to satisfy their hunger.

JL: This question to you, on what your thoughts are. What are the thoughts and concerns towards the fish, what are the status of today?

AN: Currently the status is good. The ice has frozen thick and the water level has not dropped low which has permitted fish to be moving under the ice this winter.

JL: This question asks according to the state of affairs currently of the salmon, what do you see in forty years?

These fish, especially the fish net areas, where nets are set, and when their owners of these gill nets do not check their nets daily, the fish rot in the nets, and the fish smell the rotten fish and do not want to swim through that area at all. That is why I have set myself as the traditional watch dog of who checks and who does not check their nets, and I tell them accordingly. The fishing grounds have to be cleaned daily and kept pristine.

JL: If you were fish and game, how would you regulate the fisheries?

AN: Remember I have said that the fishing is better during the incoming tide both here on the Kuskokwim and the Yukon. The first wardens used to come and ask the residents first about the fish, what needs to be done, etc. They do not do that anymore. They cannot get anything done anymore because it is not their homeland. But these new workers, they start flaunt their authority, changing courses, changing regulations, that is not good at all.

JL: Would you also discuss the fish weirs?

AN: These fish, when they go up to their spawning grounds, and when they get to the fish weirs, they do not want to enter the enclosures. That is why the fish do not go further up which affect their numbers in the headwaters, which in turn affects the brown bears, with a low number of foods for them. The salmon do not want to go up the weirs.

When there were no fish weirs, there was a lot of salmon going up river. The brown bears and black bears were not hungry and they did not come down to the villages.

JL: I am going to demonstrate, (rod and reeling) could you address that?

AN: That system also scares the salmon from going up river. That is why the fish are spawning in the lower regions.

JL: Mention the ocean. The trawlers, and the food that they are decimating.

AN: The fish spawn in the headwaters and when they migrate out, they go out to the ocean. The fry swim in between the bottom weeds, because it offers warmth and protection for them. And they eat from those areas. And now they are hungry because the ocean trawlers have decimated the bottom floor.

JL: In forty years, how is that going to be good to manage fish

AN: Those things have curtailed the growing of kelp and reeds on the ocean floor. It is also not affecting the fish only. Remember back in the day the birds would arrive nice and plump, the factory trawlers are destroying their food source, that is why they are arriving without any fat.

JL: The whole ecosystem is endangering birds salmon, everything from what they're doing in the ocean, meaning the food that they have to get for…what other one would you want more.

JE: No, thats good. Does he want to see to see no weirs and no trawlers in the future and having like having a healthy ecosystem?

JL: The ecosystem how it's affecting and he did also state the weather if favorable this year because of high water, so that's another one so they look at all these different things so they will know when it's going to be plentiful. Do you want to add anything?

AN: As mentioned they mince up the clams, and make clam chowder soup. They breaking it down there.

JC: do you have anything else to say?

AN: I have no more things to say
JC: You have any questions?

AN: What will happen to the things I have said, are they going to send them to the archives across the country? A lot of the young people will know these things if they see them in print.

JE: After all this interview, someone at ONC will probably translate this into English and we will go through the file, and we will look for themes, like trawlers, weirs, environment, so we will look for those things it concerns. We'll write them down, later, so we are doing this project this year and next year and use will compare…concerns and themes and bring it back to the communities and we will share this information with you and then we will have to figure out a way to effectively communicate this to the managers of fish and game and fish and wildlife and try to find ways that we can make positive changes to the salmon management, …it would be guided by all the information was shared in interviews.

JL: (explains above)

AN: I haven't said what is important yet. You know those sport fisherman in the headwaters, and in the tributaries, and from that, the salmon do not want to go to their spawning grounds. The bears are hungry because the fish do not go up there anymore.

JL: You are good.

AN: In the past before the sportfishing, and there was commercial fisheries here, and there was plenty of fish. So after the sport fisherman came, everything declined even the price of the salmon declined.

Jl: I did my best. He shares this, he asks people who cut fish, he asks why this is different, etc. so the other theme could be climate change.

JE: You guys are both Yup'ik, how old are you?

Alexie: 91

JL: 53, I'll be 54 in October.

JC: I'm 21.

JE: I'm 29 and I'm Ojibwe and Mexican.

JC: I am Yup'ik from Kipnuk

INTERVIEW PARTICIPANT: MEGAN LEARY

Interview Date: 5/21/21
Interviewer: Janessa Esquible, Danielle Lowrey
Location: Telephone
Home community: Aniak

Shorthand key:
( ) Note about what is happening during the interview, unclear of word, or spelling question
[ ] Words not said but added for clarity
{ } Action
Mmm-hmm, uh-huh are affirmative
Hmm-mmm, uh-uh are negative

Danielle Lowrey [DL]: Yeah so, the first question, please tell us a bit about your background, where you are from, how did you grow up, and what connections do you have to salmon and first what is your cultural affiliation and home community?

DL: Yeah sorry that was a lot, so your home community is Aniak?

Megan Leary [ML]: Yes

DL: And cultural affiliation?

ML: I'm Yup'ik and Athabascan. I'm also Tsimshian but I never practiced that part. We've never been around it so I don't know if you want to include that or not.

DL: And your age?

ML: 30

DL: Okay awesome thank you. And now the first question, so please tell us a bit about your background, where you are from, how did you grow up, and what connections do you have to salmon?

ML: Okay, so I live in Aniak right now. I've been here for 7 years now. But I grew up in Kalskag or a majority of my childhood we moved to Bethel in 2000. So lived in Kalskag first and then Bethel but were also seasonally living in Napaimute where we'd do all of our fishing and moose hunting, berry picking. All of our subsistence took place in Napaimute. Some of it in Kalskag and in Bethel but mainly in Napaimute. Does that answer part of it?

DL: Yeah, um

ML: Can you send me a copy of these really quick?

DL: Yeah we can.

ML: And my connection to salmon. I guess when I think about salmon and how I connect to it or how it connects to me I think about salmon and my family and also the land uh how. This is a hard question sorry let me think about it.
Janessa Esquible [JE]: Thats okay. It is a hard question. We heard that from others who were interviewed too. Just because their connection to salmon is so deep. If you want, we can come back to that one later and I just sent you the questions too.

ML: It might be, cause like I think about and like I'm connected to them like I use them you know for food, but also work for a while with like being involved with like the management of the fisheries. Okay where are you from and how did you grow up? Okay how did I grow up? Well, growing up in Kalskag it was just like any other kid in the village I guess. Went to school…a lot of our time was spent outdoors. I was the oldest child and I did everything with my dad. I traveled a lot with him in the winter time to other villages by snow machine. In the summertime, I traveled with him by boat and by barge because he was the barge captain. He would travel all over and he knew people everywhere. I think back to my childhood and a lot of my memories were when I went with my dad places. He was always around elders, older Native men. My dad is white but he grew up here on the Kuskokwim and grew up in Kalskag and Bethel. He went to BRHS and he knew people everywhere. I traveled with him. We would go to a house in Tuluksak and I would sit and be quiet. It was different for me to be a woman and be included in these things. Most of the time it is boys who go with their dad and sit and learn. It was very different, but I was the oldest, so that's why I got to go. We would go to a house in Akiak or Tuluksak and I just sat and I was quiet and I'd listen to them while they would tell stories. A long time ago, traditional stories, their experiences and things they saw hunting or fishing. At the time, it was just what I did. Now that I think about it, it was interesting, and I was very lucky. Sometimes I would want to go play but my dad would say no, sit down and listen. I really respected my dad growing up. I tried to do as much as I could with him. Growing up, traveling around, getting to know people, I would think about eating everything I had to eat. Especially going to an older person's house and eating, you ate whatever they gave you. I think that helped me to have more of an open mind in trying new things. Traveling around back and forth to Napaimute…right when school would get out, we'd pack up our boats and go up there. We were there from when school got out until a week before school started. Most of the time, it was just my family, my parents, siblings, my grandma, my grandma's sister and her husband would come from Aniak and stay there too. My cousins would come and go, but they never stayed all summer like we had to. That was interesting growing up. At the time I hated it. I wanted to be like everyone else who just got to hang out in Bethel or Aniak or Kalskag and run around all summer and bike ride with their friends and do all that kind of stuff, but we had to stay in Napaimute and get ready for fishing. I learned at a very early age to cut fish with my mom. I was 11 or 12. I started to cut fish, white fish, then chums mostly, she let me practice and then strips because those are really easy. My mom would head, gut and slab and I would just make strips and tie them. Then as I got older, I started making fancy fish, like the two

filets still connected to the tail and dry them that way. Dry and cut bellies for saluunaq (salt fish), kiarneq (excess meat/salmon jerky), as I got older. Still work on blanket fish. That's always hard for me, making sure I get it perfect and making it even so it dries even. I fished a lot with my dad. We fished at night before there were closures. We would try to fish during the day and wouldn't catch very much of anything. My dad said, we'll try fishing at night. We would start at 11 and all night until 5 or 6 in the morning. We would do a bit better. That's how we always fished in Napaimute when we weren't on closures or windows. I would go home and get up and help my mom cut fish, learn how to brine and hang and take care of fish that way. I would get wood and smoke it and do a lot of hiking because we didn't have electricity or phones or anything in the beginning. We had propane gas. We would turn the generator on at night and watch a couple movies and then go to bed. A lot of our time spent in Napaimute was just playing outside, hiking, doing anything to keep ourselves busy. Alot of hands-on stuff, learning how to do crafts, playing board games. It was a lot of fun. I really wish I could get out with my kids more, but with my job, I can't do that. I always wish I could, it taught me a lot about work ethic and being busy and that there's always something to do. Taking care of things because we weren't able to go to the store and buy a new rope or get whatever. Coming to Aniak once a week to do laundry was the greatest thing ever. That's kind of how we grew up and then we would go back down to Bethel to start school and then go back up during moose hunting. That's why I never played sports and my grades were always bad because we'd always be going back up there right after school started and go moose hunting, come back down, wait for it to freeze up and as soon as we were able to, we'd make the trail to Napaimute. My dad and I did trapping up there which was a lot of work to go back and forth. If he was able to, he'd spend more time up there, but I had to go home, then he was trapping martin and some other wolverines. Most of the time we would trap martin because it was easier. I learned how to do that from my dad. I always think about something my dad said when I was growing up. He always told me to watch and pay attention to what was going on along the river. We were always traveling up and down. He would say it's always changing and you got to pay attention. In the spring right after the ice goes out pay attention to where the land has changed along the beach. That affects the channels and eddies the way the current flows and how deep and shallow it will be. In winter time traveling, paying attention to the ice and when the water drops underneath the ice and the sand bars, somewhere along the river, gravel and sand bars, need to pay attention to them. I didn't realize that I do that. One time, I brought people out boating and I said look at that, and they said how do you even know this stuff, and I said I don't know, I just notice it. The river and land is always changing, so paying attention to that because it affects how we travel and the animals we harvest. Did that answer how I grew up?

DL: Wow, thank you for sharing that. The next question is, could you tell us about salmon fishing when you were young. You talked about fishing at night. Is there anything else about salmon fishing when you were young that you want to share?

ML: I am still pretty young compared to some of the other people you'll interview. I was able to fish before restrictions. I'm pretty young and I didn't understand what was going on. My first memory, I remembered my parents talking about the fishing windows when they would and wouldn't be able to fish. This was early 2000s. I didn't realize that was a

scary time. I didn’t understand what it was for or why it was that way. I don’t think that lasted very long. We were able to go out and fish however we wanted to. I think about that and when it was open all of the time, it was a lot harder for us fishing out of Napaimute to catch fish. We got like 12 fish and that was good to work on. That would be a mixed catch. I had a little set net too. My dad gave me a net and I found a little eddy and I would check it every day because I thought I need to help my family. I was 12-14 and had my own boat and everything. I would go and set this net. I would get chums, reds and once in a while I would get a little king. It was a little fifty-foot, five-inch mesh. I don’t know where the net is now, it was old. It was a lot harder for us to fish. We’d have to work really hard fishing all night and when we got 20 that was like super good. We just worked on whatever and whenever we could. Across Napaimute there was a family who stayed at their fish camp all summer. Soon as ice went out, they would come down and wouldn’t go up to Chuathbaluk until moose hunting. There was a boy who lived over there and go back down to school. It was the same for them. We took turns drifting in the same spot right in front of Napaimute. They were older and would drift in the day. Now I think with a lot more advocating for the front-end closure that’s been happening and more closures in the lower river, in hopes to get more fish upriver, it’s a little bit easier. In Napaimute we wouldn’t start fishing until the later half of June and then we would get done by the week after fourth of July. We would do the last fishing end of June and into early July and be smoking and drying after that. Now, I get done a little bit earlier. By the end of June and beginning of July we are done fishing [for Kings] and able to just smoke. We worry about jarring later with silvers and reds. The difference now is that it is more restricted. It’s more confusing to me because I am paying more attention to what’s going on and the difference between federal and state and trying to keep those clear. I’m in Aniak right on the boundary. It’s a little bit easier to catch. Being able to use fish wheels made a big difference because those can operate 24/7 with a live box and would have to return Kings. I think that more fish are making it up the river, especially Kings, with the front-end closure. Talking to people during the summertime….last year (2020), it was harder for people way up river but they were getting more. They are seeing fish in Takotna that they haven’t seen in a long time, but I don’t know. I’m still young. I’m paying attention more especially now that I do my own fishing. I know when enough is enough for my household.

JE: Thanks Megan. You were saying that using a fish wheel has been helpful. Were you all not allowed to use fish wheels when you were younger?

ML: I don’t know if that was a regulation or not. We didn’t have a fish wheel. I know Stony has used them for a long time and Georgetown had one for a long time, Sleetmute may have had one off and on, but it wasn’t really familiar down here I don’t think. There weren’t really any here. I would have to familiarize myself with Dan Gilikan and them and how this fish wheel concept came to be so popular. Napaimute got some funding and hired Nathan Underwood to build the fish wheels. Napaimute got these grants and it became something in the management that if there was a fish wheel to be operable 24/7 with a live box. Napaimute was granted the funds. Napaimute, Aniak, upper and lower have fish wheels and they became popular. People would catch whitefish, and it helped us get them a lot quicker and less effort. We would just have to drive up there 2-3 times a day and check it and get whatever. We shared it with the fish camp across from us. They would check it

2-3 times too. It wasn't back breaking work, catching snags and all that stuff. I don't know if they were allowed back then, it's something we didn't use. I don't know if it was because people were so used to using nets and being able to catch what they needed with drifting and set nets or if people just didn't know how to build them or didn't think there was a need for them. I don't know. Those were all changes in management and before I was getting really involved in the technical part of the fishery management.

DL: How did you learn to manage and care for salmon when you were growing up?

ML: I learned a lot of it from my dad. When we were younger, my mom and dad would take us fishing and do cutting. My mom was the manager of the family and would keep track of whatever was caught. She was the manager of this. She would say okay we have this much in the smokehouse and then we need this much fish for freezing and jarring. It took years of saying okay, we put this much away last year and ran out of dry house in February. It took time to figure out what would sustain our family and to be able to say when to stop. I remember one summer we were open up there and the run wasn't that good and we had to make that conscious decision as a family…especially when my dad was on the working group…before the fish commission was formed and had any traction. We were aware of the decline in kings and it being just open up there and people above us still need to be fish. We were just one little family fishing out of Napaimute fishing for 4-5 households. We just had to make that decision and think about when we need to stop fishing for Kings. Even though they are here, and we have this much, but we need to do our part and also accept that we can catch reds and chums still and need to stop for a little to let some of the fish pass. We were supporting multiple households. We probably didn't make a difference, but just having to make that conscious, fair decision…we wouldn't have been able to do this if we weren't involved in the conversation with the working group and knowing the numbers and escapement, all of the western data and conversation that happened. Talking to other people along the river, that was what my dad was really good at. From knowing people all over and once we had cell phone service in Napaimute, being able to text and see how fishing is for other people and sharing our fishing reports. Having this conversation to find out how people are doing in different places and having to make that conscious decision. Even though we are just one family, we need to do our part to help conserve. We can't sit at the table and preach conservation if we aren't doing the same thing. Being involved and knowing the background on the fisheries has helped us making the decisions to manage ourselves. I know this isn't always the case with other people who aren't involved or aren't aware of all of these conversations and the meetings that happen in the winter, the harvest data, what gets to the weir, inseason harvest data, Aniak and Bethel test fishery…It's easier for people who don't know about this info to say let us catch what we need, we don't waste anything, and let us fish. I can see that if they are not aware of all of these different things and information…We don't ever 100% know where the fish are and what they are doing. It's all a guess, the best educated guess. It's easier for people who aren't aware the decline in chum and decline in kings to just say let us fish, let everybody fish and asking why can't we fish? It's been our right forever. I would like to do that too but we just can't. We hope that the management decisions we make will help. Learning to care and be a steward of the salmon…a lot of that learning is from my dad who he learned from elders in Tuluksak

and Kalskag. Being respectful. Whenever we would catch our first fish in the summer, usually we would give it away to an elder across the river. We would cut it up and share. Elders need the first taste of fish. They have been around forever and we need to share this with them. It was always a really good feeling. They were always so happy because they wouldn't usually start trying until the ice went out. If we caught a whitefish, we would share that with them. We would always take really good care of our fish. When we were cutting them, we would be respectful. If we ever caught a really big king, which is really rare, my mom would be so thankful for the fish and thanking the fish for giving itself to us, and coming to our net and talking to the fish and being very grateful. I always thought that was a respectful thing to do. Anytime we ate fish we would keep all of our bones and burn them. Usually, we bring everything back out into the woods and not throw anything in the dump. With fish, my dad would tell us, when it's fishing season, you don't through them back into the water, you have to burn them because if you throw your bones back in you disrespect salmon. I don't know the difference if your cutting fish and throwing stuff back into the water, whereas the bones we would burn. In the smoke house, my mom was very diligent about taking care of her fish in the smokehouse. I don't ever remember there being spoilage. It didn't matter what the weather was, rain, shine, wind, cold, hot days, it didn't matter. She knew that everything we got we had to be respectful and take so good care of it because those animals, those fish gave themselves to us to provide for us. All of our hard work going into catching and processing the fish and hanging them. She would find lots of different ways to get creative to make sure there was no spoilage, like fans, hotter heat, fire, or no heat on hot days, just a little smoke. She'd hang them outside only overnight. Our fish have to travel so far. Even when we did hang them outside, we'd light smudge underneath to keep the bugs away. One thing I learned from my mom is she went through her smokehouse and fish every single day. Every piece of fish hanging in her smokehouse she would go through and look for eggs, make sure there were no eggs, she would make sure there was no spoilage on anything. If something wasn't drying correctly or getting enough smoke in a certain part of the smokehouse, she would move fish around. It was very, very rare that we ever had to get rid of a piece of fish from the smokehouse. That's something that I always admired about my mom. It takes a lot of time if you work extra hard, it can be done. That's what I do too now, even after work, I go through my smokehouse and make sure nothing smells, nothing has any flies or anything on it. Use a fan if I need to for extra air. I know some people don't have that advantage or aren't able to do that without a generator or at their fish camp. This is also something different that a lot of us do now. Especially up here, there's hardly any fish camps. Everybody moved to doing stuff at their house because alot of us who have jobs can't take that whole month to go be at fish camp or even in Napaimute and fish and process fish. Even if we didn't work, the gas to get back and forth and then the different fishing opportunities. Maybe it's a change in our generation wanting to be more in town. Alot of us up here, we do everything right outside of our house. This is something that has changed. With this, you lose a lot of your traditions and teachings. Down there, they talk about we are losing a lot of our learnings and the work that goes into being at fish camp, the family dynamics, and the responsibilities, getting wood, getting water, and watching the kids cleaning the house, fishing, mending nets, chopping smokehouse wood, cutting fish, the different roles and learning those things. Those things are getting lost because we aren't at fish camp or in Napaimute. That was just life every day. This is really sad. I

see it in some of these other kids where their families were very about fish camp all summer long, all of the kids, cousins, everybody…learning and working. Now some of these younger kids from these families don't get the same experience. It's sad. It's different when you are in town and there are so many distractions.

JE: Thanks for sharing that, Megan, so you are saying with the fish camps being moved to home, that it's harder to pass on all of those values because there are distractions and people have to go to work and they're not able to fully focus on time at fish camp with family and tending to all of the other responsibilities?

ML: Yeah. It's like a part of your identity you know. Along with just the work, the fishing and taking care of the fish and learning how to do those things, there is also your connection to your family fish camp. Some of those fish camps have been in people's families for generations. My kids would be 6th generation to use Napaimute and living there and fishing and doing everything around there, but I am doing it out of Aniak. Both RJ and I have to work. 2 years ago, I was going back and forth from Napaimute to cut fish and help my mom right after work, get up there, do fish, come home to Aniak late (midnight/1AM), get up and go to work, but it was very tiring. It was 2 hours a day of travel and then having to work on fish, get your kid to bed and get to work. I had to make that decision to stay in Aniak which was easier for me and my little family. I'm sad but maybe one day I'll bring my kids up there. Having your connection to your family, your history and place, your identity, stories, hearing stories. When you are working together, my dad was always telling stories when he was growing up and fishing even though he is white, his family lived in Kalskag and they subsisted and did everything just like a Native local person. They fished, trapped, got wood, hunted, dried meat, learned how to skin sew and everything and commercial fishing. There were times when I was 12. When I was 8 or 9 my dad built me, a wood built. He put oar locks on it on purpose and one day we went out and he said we aren't going to use the motor. I said okay what are we going to do? He said you're going to row. When I was growing up your appa used to make me row because gas was expensive or they didn't have money to waste on gas. It was hard. I was like holy cow, it is a lot of work. It was easier to throw your net out and you row your boat and so I just sat there with the oar lock and rowed backwards and kept the net just tight enough and move it around. We did that for a couple nights. It was nice and quiet, we just went with the current, no motors or noise and controlled our net by paddle or rowing. There would be stories about commercial fishing and using wooden boats, really small motors, just learning different things like that. It gets lost a lot of the time too without being at fish camp. Being in town, more distractions, even when you're out fishing, there's a lot more boats around you.

DL & JE: Thank you for sharing.

DL: This was touched on just a bit. Are these teachings/rules still being taught and follow today? Do you still burn the bones of the fish you eat? Do you see families paying attention to the runs and conserving the king salmon when needed? Just what you've talked about in the last question, do you still see that today.

ML: I do, and I do them within my little household. I'm not sure if other people do that. There may be less. Definitely not as much as when I was younger. Even simple things like taking care of your nets, my uncles would always tell us, don't step on your net, don't ever step on it because it's so disrespectful to your net. You walk around your net, you put it away at the end of the day nicely. That provides for you and feeds you. Sometimes I have to remind RJ, which he grew up with traditional grandparents, so I always ask him how come you don't do these things or teach our kids, and I think maybe it's not as important or real in this world today compared to when we were growing up with our grandparents and parents, making sure that we were still following the rules and traditions closely because they were like unwritten law growing up in their world. I feel like a lot more of that today is not being practiced or shared. I was thinking about this last night, there was something I thought about what my dad had us do growing up. I don't even do that with my kids right now. I think the world we are living in right now is so busy and so modern and technology, we are forgetting that these things, even though these unwritten rules and traditions, they are still very important to our identity. I just don't think we are remembering the importance of that and not passing them on as much. I am guilty of it too. You're first period, that summer, you aren't supposed to touch fish, and if you were going to, there was little things you had to do that would make it okay. No swimming and things like that. Those are definitely not practiced today. If they are it's very rare. I was told to do this when I was younger 20-25 years ago [chuckles].

DL: So next question, how did your ancestors manage, steward slash care for salmon?

ML: I think that they would manage kind of how I talked about earlier. Mom and dad would keep track of the fish that they needed, know how much dry fish to get, how much saluunaq, for canning and freezing. I think it was like that. I haven't read much on what the fish numbers were back in the 50s/60s, 40s/30s, but it could be managed internally. They also had dog teams and they made sure they had enough and knowing when to stop so that they weren't taking too much, but a long time ago, they didn't have stores. They had to make sure they had enough, but maybe they had tough winter, but the next year they would have to catch and put away more for their dogs. In caring for their fish, I think they were more respectful in the way they took care of their fish. The traditions, the unwritten rules, respecting every animal, which has a spirit, every animal can hear you when you talk about them kinds of things. To see you're a respectful person, to take care of the animals you catch, the plant and berries you pick, harvest, and then they want to come and give themselves to you if they see you respect them and the life they are giving you. It was definitely a lot more care, be careful and respectful like the way they took care of and harvested and processed the animals.

JE: You mentioned that now days, they are so busy, modern technology, are there other reasons why some of these rules are not being taught. Is it that they are not being passed down or what's causing this?

ML: I think a number of things. Up here, we have very little elders. We don't have any true, true elders anymore, maybe one. Just like a handful from Kalskag up to Stony. There are so very few. Those people know the real old ways and the language. There was that

disconnect. Someone referred to them as the lost generation. When times were changing, they are the people who are in their 60s or early 70s, there was changes and starting to be more modern world influences and so people were starting to go, they were still fishing, going to fish camp, hunting, moving, speaking their language. There was that modern influence and it got to be less and less traditional. There is that generation that is coming into the elders that were influenced a lot by more modern world things. Alcohol…It went from less traditional to more modern, still having some of those things, but then they weren't teaching them to their kids, which would be my mom's generation and a little bit younger. My great-grandma could speak Yup'ik, tan a moose hide, mend nets, she could do everything, know all the plants to gather for medicine, when to gather them, how to put them all away. She could birch bark baskets, and know when to harvest and take care of all of that. She could do so much, and there was a lot that she didn't pass on to my grandma. My grandma can't speak Yup'ik, got sent away down states and went to a boarding school, came back, but she had been gone so long, she didn't even know much about her culture. By the time she had come, Bethel was alarming and more modern. Then my mom's generation, so it was less and less. This is just my family, not for everybody. My dad knew a lot and was able to teach. He knew a lot and was able to teach. He knew a lot because he was with Sam Savage, Willie Pitka and these really old people. They all passed away. He was learning things to make sure that he would know. Change in time, technology coming in and now too, like bear has ears on the ground, I can't talk about them. This does not make sense to anybody in seventh grade right now, a bear doesn't have ears on the ground, that's not true, that does not make any sense, why should I listen to something like that or a moose can't hear me talking disrespectfully about them, so I'm just going to ride around in September until I shoot it. That's the way moose hunting goes, but they don't see, these things don't make sense to them. Their parents may know these things, but it's coming out of their mouth and it sounds silly, but it's not, it's all a part of our connections to our land, our fish, who we are. Even though it sounds silly or doesn't sound real, it is. It teaches you to be a respectful person and more careful. It keeps you grounded and your history, so I am making a mental note to myself, like Megan you need to do this for your boys because it's important. Did I touch on that question?

JE: Thanks so much for sharing, really powerful you've shared so much, and were on question 6. We have maybe like 4 more questions. Really good to hear what you have to share with us. I hope you're able to pass on those traditions to your younger kids. It's sad to hear that some of the youth there aren't aware that animals can hear them anymore.

DL: So, you briefly mentioned that in Aniak, there are very few elders. Is there any reason for this or are more elders leaving the community?

ML: Alot of them have passed on. There are some that have moved away. If they need special assistance, they couldn't get in Anchorage like a direct care provider or to live in a home or require more attention. In Kalskag, they mostly just passed away. In Aniak, a lot of them have passed away and now their kids that are in their 60s and 70s. There is this new generation of elders. They are new elders. I was thinking of elders, the people

that I used to sit in their houses with my dad. They are all gone. Sorry, I should have been clearer on that. We do have some, they are just the newer generation of elders.

JE: Thanks for clarifying that. Did you have any other questions for her Danielle?

DL: No, no questions.

JE: Ok, the next question is do you feel that your Yup'ik and Athabascan knowledge and values are reflected in fisheries management?

ML: No, not so much. I think now there's more influence working with the Fish Commission. I think that some of that is being taken into consideration now. There was a call on KYUK a couple weeks ago and people were calling in and one man from Bethel called in and was very passionate. He was very right about managers not thinking about perfect drying weather, lack of opportunities and at fish camps, lot of the traditions and teachings are getting lost. I guess, part of that is we don't think about that part. They are trying to balance mostly the western science in keeping things closed. We understand why it's best to keep things closed in the beginning even though it's not the ultimate/best way. For us, spiritually, traditionally and culturally, people just want to fish, let us fish, it's what we've been doing forever. I can understand that because you're always getting ready for each season. Right now, people will be getting ready around Bethel, out setting nets. The fish are down there earlier. No, but I don't know how to answer it right or the best way to explain it. No, I don't feel like they are, but don't know how to elaborate on this more.

JE: You talked about western science and numbers and then a lot of the information is used to guide management, so do you think western science dominates or plays a more important role or larger role in management versus elders and considering what the drying weather is like, things like that.

ML: Definitely. Even in the fish commission, when the inseason managers or executive council makes decisions, we use western science. We use all of those numbers and all of that data to make a decision. There are elders on there who give us their more traditional observations and we listen and we acknowledge them, but at the end of the day, inseason managers and the executive council looks at the western data because that's all we have. We don't have a traditional way of gauging this information or this data. It definitely influences the decisions that we make even though we're subsistence users and lived on this river our whole life and our families have been fishing forever, all their lives, for generations and generations, we still use western science to make management decisions. The state totally uses western science.

JE: If an Elder were to tell you based on the number of swan eggs or geese eggs he may see out there that the salmon run is looking good, it could be strong where there's going to be more fish coming back this year, can you use that information to guide your decisions that you make?

ML: I would have to look at it. Okay, where do they live? Is that the same case like up here? There are a few big lakes where migratory birds go and lay eggs. I guess I would also have to also ask other people locally, have you been out to big lake or whitefish lake and how were the eggs up there? I would ask around here to see if that was the same case or if it meant the same thing. Different parts of the river have different dialects, words for things, meanings, the way the grass grows and stuff like that. I would take it into consideration but would also be curious about other indicators along other sections.

JE: I see ok, awesome thanks. Do you have any concerns or worries about the salmon fishery today?

ML: Yes, I think that maybe this is how it's going to be forever. That is one of my concerns is that this is how it is going to be with these scheduled openers that we won't go back to free for all fishing days and living at fish camp. How do we even get back the numbers? It seems that every year no matter what we are doing, and I know we won't see results right away, because the fish go out to the ocean for x amount of years and come back. Is what we are doing right now the only way we are able to fix this? I know that we can really harvest if it's open to whoever and open all of the time. We can harvest thousands of kings (80 thousand). What else do we need to do further? How can we do that to help our numbers? What else is there that can be done besides putting it on the subsistence harvest users? Also, how are we going to be affecting other species? The less we will harvest on one, the more we may harvest on another. That'll be interesting. What year was it when all of the fishing happened down there, when people were fishing in Akiak and other villages when it was closed and they finally opened it and said to go fish. That was the only day that we fished. We fished one day so hard and the chums were running so hard and my mom and I we cut fish, like 80 chums in a day. We had a few reds and maybe a few kings and we did a lot of whitefish. We dried so much whitefish for dry fish that year. I think about those times where we are supplementing the kings we aren't getting, we are using a lot of other species. Should we be looking at those numbers as well like whitefish?

JE: You kind of touched on this question already regarding concerns you have with fisheries management and research. You said are we doing everything we can by restricting people on the river, is that really going to help the fish come back and how are those management regulations impacting other species? Are there other concerns you have about the way the fishery is managed today or the way the research is being done?

ML: Not much to say about the way the research is being done. I can understand Bethel test fish, Aniak test fish, and their purposes in assessing where things are. I know the channels change, we always hear about Bethel test fish because the fish aren't going that way anymore but it has to be consistent with science because if you change your location, it's a new set of data, you wouldn't be able to use bethel test fish numbers from previous years. Aniak test fish, I understand that one. I worked on that the first year it was in operation and was pretty cool and fun. We got our first king on June 1 and we were all like this is so amazing. We cut it up and gave it away to elders and we tried to share it with as many people as we could. It was such a cool experience and thing and project.

Sonar is kind of iffy because there are so many other fish swimming. They claim they can tell what kind of fish it is by size and number. I commend them for the different type of inseason harvest monitoring they do. There's no other way I can think of. When I was younger, I wish we could lift up the water really quick and see what's underneath. If only we could do that in a perfect world. In terms of management, federal and state differences and working relationships that those two agencies have with the advisory groups and also with each other. I really wish, in being on the fish commission, this is really…I am being more biased…it's opened my eyes a little. I really wish we could all sit down together and decide things together, like the working group, the fish commission, fish and wildlife, the state. I know those two have different responsibilities based on state statutes and ANILCA and ANCSA, but in reality, if we are all trying to work the same river, the same people, the same thing, it would be really helpful if we could all just sit down together all throughout the year and do these management decisions together. I was talking with somebody last week who called me very distraught about the emergency order sent by fish and wildlife saying when it was closing and what it could do. They were very worked up about the language of it. They said this is going to contradict itself, the state should have used this and that. I just said ok, you know what, an 82 year old elder man in Aniak who has been fishing and living here his entire life, never fished anywhere else in his life, never fished out of state, they don't care who the announcement comes from. They don't care the wording, the language, you are allowed to use, this supersedes whatever, they don't care. All they want to know is when they can fish, where they can fish, how long they can fish and whether they can use their 6- or 8-inch net. It really doesn't matter. If we could just work together, we are working for the same people. People really don't care about the little tiny things and details. They just want to know when, where and how they can fish because that's all they care about. That is one of my other concerns is everybody getting at the table together and working together.

JE: You think that one of the ways we can improve the management is by building relations?

ML: Yes.

JE: Is there something that…you mentioned different laws they have to abide by? Is there anything preventing that from happening already?

ML: I don't know.

JE: Is there anything else you want to share on improvements or on what's not working and what's working?

ML: No, I think I touched on most of it.

JE: Okay, thank you.

ML: I'm kind of nervous and distracted because my son is at work with me.

JE: We’re almost done and you can get back to work and your baby. One of the last questions is what does Indigenizing our ways of knowing salmon management look like to you? What does bringing your Yup’ik and Athabascan values into management look like?

ML: I don’t know.

JE: Do you feel like right now, you don’t know that there is space to do this or how to do that?

ML: Yeah, can you give me examples of what other people may have said?

JE: One example we heard was that families used to listen to the head of the household and the elder in the family and how many fish are coming back and listen to their relatives or that individual who was telling them like hey the chums are here but not looking good, I don’t think they’ll come back strong, let’s lay off the chum. Another example is just having more Yup’ik and Athabascan people, Alaska Native people from the river being a part of the management system.

ML: Like with the fish commission, having local people at the table. The decisions the commission makes is based on western science, but when we meet, we bring in our values, we listen and are observant and respectful. I don’t know if that's a hard question.

JE: That’s ok, we can move onto the next question. If you think of something else later you can always call us or let us know.

JE: What do you want to see for salmon and your people on the river in another 40 years?

ML: More fish and people were able to get out and not be told when and where and how. That would be amazing and the best scenario. I don’t know if that will ever really happen. I would like to see more younger people getting involved locally and on management boards. Like you said earlier, I think we can best advocate for our people and our salmon. We have that connection. We also need to bring that back. Remind people, maybe we aren’t following the old rules good enough and maybe that’s a reason. Again, to some people it may sound silly but I think having that connection, sparking it and bringing it back, the culture, the traditional culture that comes with the first fish of the summer and how you take care of it. Having more opportunities, I guess and like something that comes up a lot when I talk with people in Aniak when we are doing our inseason harvest monitoring, some people totally give up and like why? They are sick of being told when I can and can't do something. We have the opportunity now to fish, how come you're not out fishing? It’s too much of a hassle to keep up with the regulations. I see the frustration with some people up here. I am wondering how we can make that better for you so that you aren’t feeling so frustrated and mad that you can’t just go out and fish? Trying to somehow get more fish in the river and then we don’t have to be to the point where it’s restricted.

JE: I see. Have people in your community stopped fishing? They are no longer fishing at all because of all the restrictions?

ML: Yeah, some people in Aniak. Whenever there will be an opener I text or call them. I think it’s because there’s no job, gas is expensive, motors break down, feeling defeated. There are all these economic factors, high cost factors, new motors working when it’s not time to fish, not working when it’s time to fish. If you really wanted to do it you could do it. If you want to find or partner with somebody…or help out to try and get a little fish. There are some people who have just stopped and it’s unhealthy, mentally, spiritually, culturally, physically…it’s sad.

JE: In the next 40 years, you’d like to see people reclaiming Yup’ik and Athabascan cultural values, beliefs and practices?

ML: That’s like a whole another issue in itself. People up here don’t speak Yup’ik..the people who do haven’t taught anybody. There are very few people that speak Athabascan way upriver and they haven’t taught anybody. There’s just very little cultural identity up here and I haven’t figured out why. It’s pretty strong in the lower Kuskokwim and on the coast…maybe it’s because we’ve had contact with the Russians for longer? I don’t know. There is definitely a loss of culture and cultural identity, traditions, language and something that we can only try to work on. We are trying. There are some programs that we have and we are trying.

JE: I’m really glad that you are a part of that work and in reclaiming your culture, language and identity. Thank you so much for sharing. I’m not sure if Danielle has any other questions.

DL: No follow up questions. Thank you, Megan, for sharing.

ML: Thanks, I hope I was clear enough.

JE: Oh no, not at all. We’ll record and transcribe and take some really good notes.

INTERVIEW PARTICIPANT: NANCY SIMEON-MORGAN AND BETTY SIMEON
Interview Date: 5/20/21
Interviewer: Janessa Esquible, Danielle Lowrey
Location: Telephone
Home Community: Aniak

Shorthand key:
( ) Note about what is happening during the interview, unclear of word, or spelling question
[ ] Words not said but added for clarity
{ } Action
Mmm-hmm, uh-huh are affirmative
Hmm-mmm, uh-uh are negative

Introduction to project, review of forms, benefits/risks, & compensation.

Start at 8:50

Janessa Esquible [JE]: Where are you both calling in from today? Aniak?

Nancy Simeon-Morgan [NSM]: Yup were both from Aniak.

JE: Is Aniak both of your home communities?

NSM: Yup

JE: And what is your cultural affiliation? Yup'ik, Athabaskan?

NSM: Yup'ik.

JE: Yup'ik. Okay. And old are you both?

NSM: I am 50 and mom is 79.

JE: Okay great. And to get started you both are welcome and encouraged to answer all of the questions. We only have 10. And we'll get started. Can you tell us a bit about your background, where you're from, how you grew up, and what connections you have to salmon?

NSM: Okay my name is Nancy Simeon Morgan. I grew up in Aniak all 50 of my years. We live subsistence lifestyle, was trained and taught by my mother to cut fish and continue the Yup'ik culture of that native woman in Alaska. And tell them your name mom.
Betty Simeon [BS]: My name is Betty Simeon. I was born in Kalskag, 15 miles below Aniak. I moved to Aniak when I was 3 years old. I lived here ever since. I live subsistence lifestyle. That was my way of life.

JE: Quyana. And could you tell us about how salmon fishing was when you both were younger? And it is different than today.

NSM: Well I'm not that old but the big difference from today and from years ago is we all gathered at fish camp and did our business for however long it took at fish camp. And I think that is timing of people telling us when were going to fish, how much were going to fish, and how often were going fish. Fish regulations has really hindered the teaching of Yup'ik people at fish camp. I bet all my (inaudible) from my mom at fish camp. Every thing I know today I've learned from my mother and that's our cultural standard. Is we learn from our mother right or our grandmother. I was never privileged to have a grandmother just my mom. So my daughter is 22 years old and though in her younger life she spent at fish camp. She doesn't know the work that it entails of fish camp to this day because of the mandates, and who and what we're doing and how often were doing it. When I first started cutting fish, when my kids were little, I know maybe 30 years ago we had the

abundance to stop and teach and train throughout that whole time I don't feel being as getting older and a mother I don't feel I have that time for one, the abundance to train my daughter.

JE: Wow. That sounds really (inaudible) to hear. Thank you for sharing though. It's really powerful.

NSM: And I'll mom talk here. What was the question again it? Repeat it, would like to repeat the question.

JE: Oh sure thing Betty. I was just wondering if you could tell us about how salmon fishing was when you were younger and how is that different from then today?

BS: Its totally, totally different. When I was younger, you know everybody didn't have a boat and engine. Or you know mostly an engine. We drifted by rowing and just by drifting we got plenty of fish. We would get kings and dogs. We put everything up. We would get 300 fish a day. 300 fish a day and that was for humans and for the dogs. And everybody worked at fish. You know we would work all day into the night and next day nobody (eats?) There was no telling, you know, going to stop and when to go and there was so much, you know, everybody had plenty. Plenty to eat. All our dried fish are half dry, our salted fish, you know. And it was just totally, totally different now. It's just not long time ago and there's no, there no, I mean there's no (stealing ?). You know, long time ago (inaudible) at fish camp when you were putting up fish everybody worked. (Inaudible) gather wood, and everybody worked for that food. But right now it's not (inaudible). How we just scramble around, get what we can, go as fast as we can, to get the little fish we can. And to put that up and it's really, I don't know,

NSM: It's not a family anymore. Its hurry up and get done when you can get done. It's no gathering for the whole family to spend their time doing what we have to do. Making sure there's enough to make dog fish to dry, to jarring fish, to whatever we need to do. It's not a family thing anymore. We just got to hurry up and do it because you don't know when you're going to close again.

BM: Yeah, you know everybody would work real hard and one of us would be on the bank, and in the smokehouse cooking, and then we'd all sit and have a tea, a meal or boil fish or whatever up on the bank, take a break and then all go back fishing. It's not like that anymore it's rush, rush, rush and it's totally different but at least we get some fish versus none.

NSM: Next question.

JE: Thank you for sharing. The next question is how did you learn how to care for salmon when you were growing up?

NSM: This is Nancy. I learned to care for salmon strictly from my mother. As long as I could remember a little walking down to the raft to um, everybody had a job. Either it be

put melek or the roe or whatever you call it into the coffee cans. Somebody was watching fish, somebody was putting fish on the rack, somebody was packing fish up the bank, somebody was coming in with fish. Mom was cutting fish. Apa was doing whatever he needed to be doing. Making sticks for the flat fish. Dad was off coming in with the older boys with the fish. There was uncles and boys in the smokehouse moving the fish around. But I'm 50 and I am probably the last age group that can do everything from beginning to end. Our culture has probably from neon down age is just gone. I taught a lot of girls how to cut fish and jar fish and take care of things but that's just me. My mom wasn't, we, you know, were village people. We grew up, a lot of times there's people that is intoxicated quite often. Parents, my mom and dad, had many people, other people's kids, I was always proud to know that I always come home to a sober mom and she was able to teach me from beginning to end, every single thing I know from fish to birds, to moose, to you name it. Sewing, anything you can think of I'm privileged to know that my mom has taught me.

JE: Wow thank you.

NSM: Now that's not what happens. You don't have time to teach nobody. You don't have time to be on this (inaudible) and learn how to cut a blanket fish which entails fileting a whole, the whole entire fish off the backbone. Theres no time for that. I haven't even taught my daughter and she's getting old, she's 22 and to not know anything. But here is my mom. Can you repeat the question?

JE: Oh yeah. I was wondering how did you learn to care for salmon when you were growing up?

BS: That was just part of our life. As soon as you could start gathering. You know gathering (stakes) our world, that's what you did. Thats just.. You just grew up doing that. By the time you were 11 or 12, you know you can hang fish and you can wash fish and you start cutting fish and it was just part of your life. You were taught, you know, as you went on. Like when were dog fish. You know, chum. The good chum were cut for eating fish. That chum that are kind of bad we cut for dog fish. Mama would just say 'this is for dogs' you know. And there was (inaudible). No canned. … put here for the dogs. And everything was saved. And you just grew up doing that knowing but now they, it's not there. If there was general girl came by, (inaudible) would start cutting fish. She probably wouldn't even know a uluaq is or if I told her, it is for dog fish. I mean it's not there. But I'm sure somewhere down the line some of what we know these are lost and well be helping. I still help my baby she's starting to do the big blanket fish. And she does all the cutting of the strips, the fancy fish. To the half-dried heads and tails. You know we still save everything and use everything how we were taught. You don't waste anything.

NSM: Okay next question.

JE: Thanks yeah I actually had a follow up on what you all were saying that for Nancy that you might be able the last generation to know how to do everything from beginning to end that you, your daughter you haven't been able to teach her everything. She's 22

and that's getting a little bit older to be teaching the things compared to when you were taught when you were younger and I'm wondering so is that happening because, are these ways being lost because there's the fish, there's not as much fish anymore. Theres too many restrictions and the style of fishing you have to rush rush rush. And you didn't have time for that?

NSM: Yeah you somebody says you're opening at 6 o clock in the morning, you know that boys, my kids go out fishing and might come back with 5 fish and there going back out fishing you know if they're going to come back with 15 fish or 20 fish. There's not a whole lot of time to be teaching anything you're numbered. Everything's numbered. Theres no, okay we have 20 fish on the bank and were going to show you, for say, maybe mom would say were going to learn how to cut fancy fish today because the boys brought in 20 dog fish last night and 5 reds or something. that's not, we usually don't do that anymore. Theres no, it's not like my daughters not willing to learn it's just you don't have the time.

JE: Wow. Okay thank you for sharing. I'm going to let Danielle finish up with the next question here.

Danielle Lowrey [DL]: Yeah thank you guys for sharing. This next question was kinda touched on and so are these teachings/rules still being taught or followed today? Nancy you talked about how your daughter doesn't have the opportunity to learn as much now with more restrictions on fishing. Is there any rules that are being followed today that you have learned when you were younger?

NSM: Will I do, I follow. I'm 50 years old and if my mom said that three big king salmon that came in is going to be made into a blanket fish you better believe this girls going to be making into a blanket fish. I still to this day follow what she tells me to do. Regarding fishing. The tradition is not there like I said. Not being able to be at fish camp and getting up in the morning and mom is down there getting the sourdough pancakes going. And were going to go down and first were going to cut strings and then were do this and then were going to you how to do this then were going to do saluunaq. It's not there. You don't have the opportunity to do anything from beginning to end anymore. My daughter helps me throughout fishing all the time. But we don't have the opportunity to say go under the bank and or the fish to screw up you know. We work on limited amount of eating fish and if I gave her a huge king salmon to make a blanket fish and she screwed it up then what?

JE: Wow. I had actually wanted to follow up on Danielle. You mention earlier that I think it was Betty that there's no waste. Do you still feel that that's rule still being followed today?

NSM: In my family its being followed. Yes. We get clean all the backbones, we scrap off and we jar. You know for making like sandwiches and stuff. And the backbones itself we hang for the dogs for the winter. And when you're taking down your fish, the fins and whatever we save for our animals for the winter time. Like I said earlier were still subsistence livers. We still live on what we bring in. And my daughter knows the aspect

of fishing I mean she knows what we're going to do she knows how to make the brine. She knows how long we need to brine. But the art of cutting fish is not, its lost somewhere. And I can contribute that a 110% to (kicking). I feel like fishcamp is my life. I love that (inaudible). I would give Christmas, I'd give slaaviq, I'd give up Thanksgiving, I'd give up any holiday to be in my fish camp for the time I can be off work. And we can be down there as a family and that is not happened in years.

DL: Wow. Thank you for sharing. Betty would you like to respond to this question as well?

NSM: Can you repeat the question please? And mom you need to speak up a little bit.

DL: So the question are the teaching/rules that you were taught still being followed and why or why not?

BS: Yeah. I've taught my baby everything and my grandkids. When they're around like they used always come down to fish camp and we'd all gather there and sometimes with all the grandkids. And they would all pitch in but now the grandkids are grown now. And I teach my one granddaughter who's always with me, that's Nancys baby, I teach her not only fish but like to cut up moose or dry, and sewing and beading. Now she's a professional beader. She shows me well and our tradition. And now were learning, she's learning and Nancy is learning to make dolls. It's just in you. Our native way of life, I hope the people never, never, never lose that our native way of life. Our traditions. How we depend on our fish. If you don't have your dry fish, you're hungry. If you eat dried fish, you feel for and you're full, you're content. Thats how I feel. How we grew up. And always had a piece of fish. Every meal. And um, you know he (inaudible), but I'm sure my baby will teach her baby and her baby will teach hers. When the kids come by and want to learn, Nancy is always willing to help them. Teach how to do native foods and (desserts?) and gather (laavaqs) and (Briana) is good at that.

NSM: Okay next question.

DL: So the next question or actually yeah, the next question, how did your ancestors care for salmon?

BS: At fish camp.
NSM: Um, yeah that would be the same answer. In fish camp from beginning to end with no waste. All the family working until its done. That's the same answer. Except for the like, like mom said, the rowing of the boat and nobody had an engine then. Nobody had an engine. Row the boat to (inaudible). Or fish wheel.

DL: And then, the next question. Do you feel like your knowledge or values are reflected in fisheries management?

NSM: No.

DL: Why?

NSM: Mom, do you think fisheries management that come around and ask you how many fish you catch or whatever knows what they've been.. gets what we're talking about?

BS: No.

DL: So uh why do you feel that way?

NSM: If they did listen to us, we'd be having, we wouldn't be losing our cultural values. They know we understand that the fish is limited. We understand that. You know like last year we didn't have any dog fish the year before it was the king salmon. But our native people have a way of already working on that. I remember, I don't know if it was my mom telling me or my dad telling us, a one year years ago, his grandfather would tell him to lay easy on the dog fish or lay easy on the king salmon they are not going to be lots this year. Harvest more of this or that. So native people kind of took care of that and realize but now somebody else is coming in telling you what to do.

JE: Dang so just to follow up on that, so traditionally that's how management was done then by your ancestors? So, it was maybe head of the family or a relative would tell you based on their observation. Would they (inaudible) coming in?

NSM: Yeah that's what mom agrees to that too. Some day you know, the older your grandfather or the head of the family would tell us, if it goes for the same thing as my dad telling my boys don't be catching, try not to catch any martens this year their count is going to be really low or you only get the moose that you absolutely need and share throughout the family there's not enough cow moose. We still listen to what the head of our whole family says. But that's completely taking away from us for the fishing aspect of it cause somebody else is telling us what to do and how often to do it and where to do it.

JE: Thank you.

DL: Okay so, what worries or concerns do you have about our salmon fisheries today?

NSM: Well, I'm just worried they're going to continue to take away the days we can subsist and the people down there in trawlers or fishermen or the whoever is making all the money off this salmon still seem to be doing what they're doing. And up here, upper Kuskokwim were kind of just hoping. And every single year that were able to do our subsistence and we work really hard to try get what we need on the little days it's given to us. I mean moms not a young girl anymore. If they give us a 7-hour opening, when the boys come home at midnight or 7 o clock or whatever it may be, chances are she's not going to be able to participate until the next opening if it's not too late you know. But I'm up doing, taking care of what needs to be taken care but we don't get to have her and she's the reason that this happens every year.

DL: Wow thank you for sharing. Betty, would you like to respond to the question as well?

NSM: Can you ask it again?

DL: Yeah, so what worries or concerns do you have about our salmon fisheries today?

BS: Just about the same as Nancy. You know, we're quite up (2 miles?) up the Kuskokwim from you know the bay down there by Bethel. And when they open at Bethel for subsistence, if they open it in Bethel and then they close us, and then they close Bethel and they open us but it is time when the fish are going to reach us, what is left? They open Bethel again, you know. Down river to subsistence. We hardly get any fish and then we have to fish above 10 miles above Aniak.

NSM: Thats the other thing that change, while thinking about it, is my children aren't in their fishing grounds. They're telling them for drifting all my life, my family has drifted in one area and my kids know that. They were taught by my dad and their dad but now we got to go out of our comfort zone and into somebody's else drifting area.

JE: Wow.

NSM: We don't know.

JE: Is that because of the restrictions the restrictions on where you are allowed to fish?

NSM: Yup.

JE: And um, I had a follow up question actually. Are there any other concerns that you all have about the fish, like anything about the health of the salmon, the size, how they look when you all are catching them. Thinking about what they look like when you cut up them when you a lot younger?

NSM: Well my mom, first thing she said, was they are a whole lot smaller but that's the truth. When I was younger, you take two hands to lift the king salmon up on the table. You can sling those things around with one hand now. They way, way smaller and used to be (inaudible). The red salmon only had the worms in the stomach and now all of them have.

BS: Yeah.

NSM: Long time ago, the red salmon were usually the only ones with the worms in the stomach and nowadays it's pretty much all the fish and there's like an area on the fish in the stomach that, last year, I don't know how many stomachs I had to ruin and cut out, there's like something, like worm eating into it that you can chance. You got to take it out and there's also puss pockets in the fish. Anything you could know mom, same thing?

BS: yeah same. With those worms and the fish are so, the king salmon are so small. Compared to what they were. They were really big. One king salmon head could feed 3 people. Now only 1 can feed 1 person.

NSM: Yeah. And the next question?

DL: Yeah, the next question what are some concerns you have with fisheries management and research today?

NSM: Well, you know everybody has a job to do and there paid to do a job. I know they have the meetings and I know they, there's a reason for everything. And I get it. We try to do what we can with what we have. If somebody paid attention, many more years ago, maybe we wouldn't be in this situation today. How about you mom, how you think about management of our fish?

BS: About the same. I think really commercial fishermen way out in the Bay or whatever should slack off with it and let our fish go be able to come up to their spawning areas. So the subsistence people can have fish. Nobody buys salmon here. Or jarred salmon or you know we don't buy salmon in Anchorage or anywhere. I think they should slack off a bit and let em' go and come up to their spawning areas. Thats all.

NSM: Okay that's it.

DL: Yeah I have a follow up question, so do you believe that these meetings, fisheries meetings, are beneficial to the community? Do you think that they allow subsistence users a voice in management?

NSM: No they're going to do what they're going to do already. They're just having the meetings to make it seem legal that they're getting the communities advice or hearing the community but chances are their minds are already made up before they even ask us anything or how we talked about it. How about you mom?

BS: Yeah.

NSM: Okay same thing.

DL: Thank you. Next question, oh well, yeah, how would you improve fisheries management?

NSM: I don't think there's any, we can say what we're going to say but they're going to do what they're going to do. We're not, they pretend that were involved. But were not involved. there's no way to try help them improve it because they're going to do what they're going to do before we even, we don't have a say. How about you mom?

BS: Yeah that's how I feel. I say things and what they're going to do but you know they do what they want to do or think is best.

JE: Yeah, I actually have a couple questions based on what you just shared. Theres a lot of things that you mentioned that just aren't working well. One of them being that you all

listen to that head of your family and that's how it's been done and now you have to listen to other people who may not even understand your subsistence way of life sounds like. And I'm wondering what do you wish fish managers knew, is there anything that they can do to make improvements to how they've been doing things?

NSM: I don't know it's been so long that we've following what the hell they have to say. I don't think the whole chain of learning for subsistence lifestyle and the way we treat our salmon is broken. I mean, I haven't been to fish camp in, how long did they start this crap?

BS: (in background answering) maybe 10 years?

NSM: Probably 10 years ago. You know I can take my 14-year-old boy and we go to fish camp but we're not cutting fish there and working hard there all day. And he wouldn't even know the get around about working hard all day or he wouldn't even know how to, that we would expect him to get up in the morning and get his fishing clothes on and get down there and get the net ready and this or that. That's already broken.

JE: Wow. Have you heard of the Kuskokwim River Intertribal Fish Commission or do know or you heard anything about the different management agencies, like fish and game, Intertribal group, or fish and wildlife service? Or do they seem most of the same to you all?

NSM: I don't think anybody even knows the difference between them, for as much as the villagers know they're all one entity doing one thing. Do you know anything between the difference?

BS: All I know is my brother is a retired fish and game officer and my daughter in law is involved every Thursday meeting and talking about fish but I don't know, it's all one thing. The people's minds are already made up and they're going to come back with the answer that they were meeting about the beginning before they even started the meeting. The answer, they knew the answer there. Just moving it in a different way so they believe they have the community's input.

JE: Wow. Yeah that's really helpful to know, just taking some notes here. I'll let Danielle follow up on anything if she needs to or she can move to the next question.

DL: Yeah, we can move to the next question. So what does indigenizing fisheries management look like for you?

NSM: What's indigenizing to you mean?

DL: Incorporating any native values or knowledge into management of salmon.

NSM: Like I just said. Their minds are already made up before they even come to the village or people, what they're going do and how often they're going to do it. How about you mom?

BS: Whoever they have on the board or working there, they have no idea what it is to sit on the bank and see your fish dry or what your smokehouse and see your fish smoking or have a big, you know, eating a big fish head and eating your neighbor's food. They have no idea about that. How your whole life revolves around the fish. They don't know that. They just come in with their paper and they (inaudible) do this and this. They have no idea. I think if they knew more, or worked from the village. Nowadays now kids don't know anything about that either. I don't know, if it would be helpful or what.

NSM: Probably too far gone.

JE: Thank you, so do you think indigenizing knowing about or managing salmon, would one of those, would that look like maybe as you mentioned earlier, allowing families to listen to, the head of household regarding like how the run, like 'hey with this salmon, like the kings aren't going to be coming in large numbers this year, lay off them' Do you think if management allowed people to just to follow their traditional ways that that would be better?

NSM: I don't know. They've been in for 10+ years telling us what to do. I don't know if it can go back, you can't break something,

BS: There's no older people anymore to be telling what to do.

NSM: My mom and dad are the oldest married couple in Aniak.

BS: Theres no older people anymore to be telling. To be telling the… I still think it's, the commercial fishing down in the bay, somewhere down in those. If they slack off and if they let the fish be for a bit to grow and come back a bit and come up the Kuskokwim/Yukon to their spawning, I think that would be more helpful. That's it.

NSM: Okay next. How many questions are we on?

DL: We are on the last question.

NSM: Okay.

DL: What do you want to see for salmon and people in your region in 40 years?

NSM: I'm sure moms not going to be here in 40 years so I wouldn't have my leader. I guess in 40 years I wish that I would've taught my children everything they need to know about fishing, I mean taught my daughter in laws how to cut fish while at fish camp, all my daughters in laws were with us but my girls she wasn't, she didn't get to experience the whole life, from when your young to when your an adult moving into your own smokehouse. It's a big deal to move into your own smokehouse and do your own fish. I know my daughter in laws got it. I really, I'm going to work harder to teach my daughter

how to teaching (inaudible) if I’m not here. But I would my family to keep our fish camp alive but I don’t know about that no more. Do you want them to repeat the question?

BS: Yes.

NSM: Can you repeat the question for mom?

DL: Yeah of course, so what do you want to see for salmon and people in your region in the next 40 years?

BS: Oh my. I would love for them to be sitting on the bank in their fish camp with you know getting enough fish for their needs for that year without wasting or overdoing. You know there's, I would love for them to have that, to having their babies running on the beach and fishing on the (weekend), you know. “I got a whitefish gram” and they would run it up the bank and show me and then I would have to go down the bank and scale it and cut it up and hang it in the smokehouse. I would love for them to get that experience and the closeness of being together, gathering their food for the winter as a family.

NSM: And that's it.

DL: Wow, thank you for sharing.

JE: Yeah, thank you so much.

NSM: Okay well, we got to move on to our next part of the day, thank you for talking..

JE: Yeah thank you so much. I really appreciate your time…

INTERVIEW PARTICIPANT: ANA HOFFMAN, AVERY HOFFMAN, STANLEY HOFFMAN JR. AND STANLEY HOFFMAN SR.

Interview Date: 6/4/19
Interviewer: Courtney Carothers, Destiny Ropati
Location: Bethel
Home community: Bethel

Shorthand key:
( ) Note about what is happening during the interview, unclear of word, or spelling question
[ ] Words not said but added for clarity
{ } Action
Mmm-hmm, uh-huh are affirmative
Hmm-mmm, uh-uh are negative

Courtney Carothers [CC]: Okay, so we'll get started...We're visiting here with Stosh Hoffman, Ana Hoffman and Avery Hoffman and Stanley Senior will be joining us shortly. It's June 4, 2019 and we're at the ONC building in Bethel, Alaska. Thank you guys so much for visiting with us on the Indigenizing Salmon Management project. Umm, Destiny's gonna lead us off with our first question.

Destiny Ropati [DR]: Each of you are welcome to answer these questions. So the first question is please tell us a bit about your background, where you are from and how you grew up and what connections you guys have with salmon.

Stanley Hoffman Jr [SHj]: I'll go first. So, I was born in Bethel, and my dad Stanley, who's late, works for the FAA for 44 years and he was stationed in Bethel and, in the early 70's, he transferred to McGrath, which is 500 miles upriver. And because my family's a commercial fishing family and a subsistence family, I came back to Bethel every single summer for my whole life mainly, primarily for salmon in one shape or form. So, of course commercial fishing has kind of gone away, maybe 5,6 or 7 years ago and so I came back to do subsistence salmon and we did it as a family group with Eddie Hoffman and his family, the family did it as a whole and then we shared all the that and then we commercial fished on the side. And um, and then I was gone for about 20 years and moved back to Bethel to work. And that's when I met my wife Ana and I live back in Bethel now.

Courtney Carothers [CC]: Thank you.

Ana Hoffman [AH]: My name is Ana Hoffman, I was born here in Bethel and grew up here in Bethel. Every summer my family would move to fish camp when I was growing up. Our fish camp is at the Nick O' Nick fish camp, umm, downriver from Bethel before Oscarville and umm, my grandmother and her two sisters would camp there so our family, the Chase family and the Nicholai's umm and our relatives from Nunap[itchuk], Napaskiak and Bethel would all gather there. It was a pretty large fish camp. My grandfather had a store in the village of Nunapitchuk and in the summer he would actually move his store to fish camp so we had a store, cash register and everything at the fish camp. It was, that's why it's a pretty well known location on the river. Villagers would come and stop and go to the

store, it was like a social place too. I always find it funny now looking back because Bethel was just maybe 10 more minutes up river but they would stop and go to his store {laughing}. Umm so he passed away when I was very young in 76' or 77', so after his passing, we didn't continue with the store at that location but we kept the fish camp there and initially at that camp location, him and his brothers extended families would be there, but after his passing, it really became my grandmother and her two sibling's extended families occupied the camp. So every summer we would leave here at the end of May when school got out and we would move to fish camp and reside there til' August. My dad was a superior court judge here and of course there were no cell phones or anything then. He was the only judicial officer, so every day he would have to boat to Bethel to see if there were any inmates to arraign. He'd just stop at the river and the troopers would say yes or no and then he would come back to fish camp. He commuted from camp. So when Stosh talked about coming to Bethel every summer, even though we're both from Bethel we didn't meet until like I was in college. They would come to Bethel and we would move to fish camp. So, we would {Stanley Hoffman Sr. entered room}. We would harvest um all the salmon species and we had three different fish racks, the Nick family which is my mom's family, had a fish rack, and my umm, the Chase family had their own fish rack and smoke house, so we kind of processed them in that way even though we're at the same location, we had our own sort of operating happening. And then umm, in the late 80's, early 90s, that area started eroding really bad and the erosion ate away at the camp site and that was the end of our use of that campsite, and we all went different directions. My mom started her own camp a little upriver from Bethel, and it's just our family that uses that. The Chase family has it's own location, not far from the original site, and then the Nicholai has their location near Napaskiak, so it was, the river kind of ended that tradition, but we all still harvest salmon in the summer, it's just not in that location with that extended family.

CC: Do you still go to camp for a long period in the summer?

AH: I don't, not the way that we used to back then. I, with the boys, we would go for several days in a row, but um, being as close as we are to Bethel, it's like 5 minutes from the boat harbor, so it's easy to run into town and check work and all that stuff, so we do spend alot of time there, but it's not consecutive as it used to be.

DR: With having the fish camps sprouted all over the Kuskokwim River, would you say it changed the whole dynamics?

AH: Mmm-hmm, yeah, yeah. So we grew up very close to our second cousins, you know because we were all at camp together, but that didn't resonate as much with my kids, because they're not as immediately interacting with those extended relatives from the villages, but they interact alot with our Bethel relatives, but if we had that camp site, they'd know they would know their Napaskiak and Nunapitchuk relatives closer, so it did affect that dynamic.

CC: Hi Stanley, thank you for joining us. I'm Courtney, my name is Courtney

DD: And I’m Destiny

SH: Where you from?

DD: I’m from Anchorage

SH: Oh

CC: Anchorage, yeah

SH: I guess you’re all from Anchorage

Clarissa Zeller [CZ]: Red Devil

SH: Oh, okay. Mmm-hmm.

Janessa Esquible [JE]: Detroit

SH: Where’s that at?

JE: Michigan

SH: Oh okay.

Jessica Black [JB]: Fort Yukon

SH: Mmm-hmm.

DR: So, a question that we asked them, um we’re gonna ask for you too. So please tell us a bit of your background information, where you are from, how you grew up and what connections you have with salmon.

SH: What you mean by that?

DR: You wanna tell us where you’re from?

SH: Where I’m from?

DR: Yeah

SH: 1944 I was born in this scum of the earth right here called Bethel, that’s what everybody called it. Now, nobody says that nomore. Cuz things are better. Cuz we got a real nice river out there {chuckles}

DR: And umm, what was it like growing up as a child here in Bethel, Alaska?

SH: Not too good, okay. Bureau of Indian Affairs took over. They thought they were...we had some Native people, young guys, that couldn't speak the Eskimo language. If they heard them say anything in school, there were four whacks, you know. Bureau of Indian Affairs, like rotten eggs, they're no good but they sent all us young guys to uh, New York city to become, to learn something about airplanes and stuff like that, and there were suddenly 6 of us and that's how I graduated from high school. I went one year in Fairbanks.

SHj: Tell em' how Bethel was when you were growin' up here.

SH: Alright. Bethel...right out here, to the far side where there was a bridge, okay, my dad is the one that built that road going over there. You know?

CC: Mmm-hmm.

SH: There wasn't hardly anybody here, in school, but…

SHj: Bethel was about 800 people back then, right?

SH: What's that?

SHj: When you were growing up and going to school, Bethel was under a thousand people.

SH: Yeah, it was. What I remember, there was 500 people.

CC: Wow.

SHj: In the 40's or 50s.

DR: And what connections do you have with salmon?

SH: I'm sorry, I forgot my hearing aid.

DR: No, it's okay. What connections do you have with salmon?

SH: What brought everything to go, I think started with the airport site out here. All white...you know, there was alot of them that took whatever, one at a time, Native women, and when they left, they left. Most of them got married.

AH: She said, what connection do you have with fish, salmon?

SH: Fish?

AH: Yeah.

SH: Fish was then, plentiful. We used to get lots of fish. My dad said we put up 300 King salmon and heck we could get that in 3 days if we wanted to. But it's cutting the fish that took a lot of time, so but, everybody had alot of fish then. All the smoke houses and everything were full because there was hardly any people here, but now, with all the villages surrounded, theres 56 villages around Bethel, and Bethel is the main money place for 56 villages, but...all the people now is hard fishing, too many people.

Avery Hoffman [AvH]: Who's your dad?

SH: Huh?

AvH: Who's your dad?

SH: What's that?

AvH: Who was your dad?

SH: Where was my dad?

AvH: Who is he?

SH: He's out there.

AvH: Tell them.

SH: Edward Hoffman

DR: And it seems as if Edward Hoffman is known here in Bethel Alaska. He is named after the main highway, and…Do you feel as if he made a huge impact, on or in Bethel? Originating the city…

SH: Okay, how it started...this place (England), uhh, his last name was Hoffman. He came to get out of there, but when he finally got here and settled in Napaimute up there and that's when he had a whole bunch of kids. You know, they were, my dad, was what? There was lots of Hoffman's….that's where they all came from up there...most of them all came to Bethel, you know. That Kuskokwim River, you go up there, there was lots of (unclear word) all the time up there. None of these places are gonna outbeat Bethel now because Bethel is where the money is on this river. But anyways…

DR: And if you don't mind me asking, do you feel as...since you stated they all came from England...do you want them interested in coming into Bethel? Was there a main reason or?

SH: Why is the main reason for what? Why did he move here to Bethel?

DR: All them guys ended up working for a place called Nyac, the gold area, not far from here and they had alot of people getting gold from Nyac, is what it's called and uhh, boy, they hired alot of people, my dad was one of them. Almost all of those guys had all kinds of heavy duty equipment and all experienced coming to Bethel...but you know Bethel added alot of time being the mudhole of the whole state of Alaska. Bethel...but you know like as kids, across the slough we'd go walking in the mud, and then the snow was so high you couldn't even go some places it snowed so hard. It used to fill the big house we had across the slough. Later on there was no snow hardly, but...and uhh, let's see...what we had with uh, Bureau of Indian Affairs (BIA)… what's the name of that village, took over all the teaching….Uhh...but anyways, the teachers were really strict, you know, but it stopped soon as you got there, and there was no more putting your hands or getting whacked, never did it again. But then with BIA, teachers were mean...but we did what he had to do with them, so…

CC: What was fishing like back when you were young? Did the state or did your ancestors care for salmon in certain ways? Or take care of salmon…

SH: Everybody had salmon, you know...salmon was plentiful for everybody because there wasn't that many people here.

CC: Were there any rules about harvesting salmon in your family?

SH: You'd go down from Bethel and there's a place called Nunapitchuk, that's where her mom is from. They had what four villages, they'd come from there and they'd get all the fish they could get from there, and most of…

AH: Johnson River

SH: Johnson river, old age can't remember things sometimes, but anyway...everybody had lots of fish, good fish. You know? Now, we're lucky if we get a bunch of good kings.

SHj: And another thing I remember is they were much much bigger in the 80s and 90s...There were a hundred pound kings. They were way bigger. You'd see them every now and then. Now you're lucky if you get a 20 pound king.

SH: We set a lot of fish here and I'd go down there with Stosh, he was young. We'd go down for two hours and catch 100 big king salmon, and they'd open it for 4 hours, so me I loaded up my boat so I'd have to come back out. The white man did right when he bought me a jet boat because it was a jet boat, I think...gosh knows...if you don't understand a jet boat, it sucks underneath the boat and it goes {makes noise}, you know? And we're way down the river fishing, and I always follow the low water like a sand pit...and I always follow that, but I go there so I get to slow making a noise {makes noise}, and I'd load up every time. Someone wanted to know my secret but I didn't say nothing.

SHj: My dad and I had a theory, he one of the first jet boats in Bethel and we used it to commercial fish and uhh...we came up with a theory that when the boat was idling the

way it handled the water it made a noise that attracted the fish somehow...We can't confirm that, but, we think that's why he caught alot of salmon.

CC: Yeah, yeah...cool

SH: But then it was $3-$4 a fish, go by the pound.

CC: Wow.

SH: We'd make a couple thousand

CC: Mmm-hmm. Did you commercial fish for a long time? A long period of your life was commercial fishing?

SH: Let's see...there was...at the time I think there was 3 and then 4 fish buyers. And they always got alot of fish but the people that got most of the fish is down below on the...umm, what's that place we used to go and shower water long on the other side…

SHj: Fowler?

SH: No further than that.

SHj: Gailak?

SH: That first village, I thought that water thing that goes up and they made a cabin up there for, what's the heck the name of that?

SHj: Government cabin?

AH: It's okay…

SH: It's old age, I can't remember it. They got lots of fish, you know that place down there past Eek.

SHj: Quinhagak

SH: Yeah, Quinhagak. Yeah, they had lots of fish, them guys…And they still got that. In fact, they invite Bethel to go down there to fish if they have an opening...which is nice...because they get lots of fish.

CC: Wow, mmm-hmm. When you guys were growing up do you remember rules about salmon?

AH: Wait, was Avery gonna answer the questions too? Or just us…

CC: Oh sure. Sorry we've been visiting so much with Avery, yeah yeah, no no, of course.

AvH: Which one?

CC: Yeah, so wondering about when you were growing up with salmon do you remember learning about how like Yup'ik ways of caring for salmon or rules about harvesting or how you should treat the salmon or share it?

AvH: Umm, when I was smaller we'd always stay at fish and umm...I'd always go fishing with my dad but when we'd come back if they were fresh enough, uhh, the kids didn't really do much, just played around and like kept the fish camp occupied and we'd all watch my mom cut fish and we'd stand around cutting table watch the hearts still beat as they're outside of the fish...Ummm, she made us, we did a lot of string tying for the strips...Umm, but, every now and then when there was still commercial fishing, every once in a while Stosh would let me go, skip school a little bit, to, we'd go way down to Fowler and go fishing in his working boat he calls it. We didn't have a jet boat, we never used one for commercial fishing, maybe that's why we didn't do too good. That's about it...There wasn't that many buyers then, it was just kind of the tail end of the commercial fishing era. Us too sometimes, and my uncle…

SHj: It was a big deal in the 80's. When I was in high school fishing was full blown and Bethel was a really big deal. Uhh, lot of people had cash cuz it was mainly cash buyers and Bethel was kind of a different place back then. It was really vibrant and it was commercial fishing and then subsistence fishing that was really prevalent in the summers. It was kind of a different place back then.

DR: Any of you guys are welcome to answer this question...Umm, so what values did your ancestors use to take care of salmon?

AH: Umm, I don't think there was any rule about the gender roles but it was just...it was just the case, that the boys and men were primarily fishing and women and girls were processing the fish...Umm, but women, like I've gone fishing and as a young girl I would go fishing when I would want to, so it wasn't, there was no rule against women and girls fishing, it just wasn't as common or expected, but the fish cutting was very much the women's umm, activity. But the men, what I remember, the men would hang around the cutting...and bring up water, pack water and supplies, sometimes like when they talked about the big heavy kings, so the men would help to you know get them up on the cutting table and Stosh umm, would help me with heading and gutting, because I'd be doing it by myself. But at our old camp, there was always lots of siblings, my aunts and uncles around so, but we would umm, for the girls, we would um, like when you have a can and you open the top of the can, we would take that top piece and fold it down and it looks like shape of uluq, so I remember when we were growing up we'd make those pretend uluqs and take grass, I mean tree leaves and if you fold a tree leaf, it can, it'll be sort of the shape of the salmon and we'd cut of the stem to open it and make little pretend fish with the leaves and have little pretend drying racks

CC: Wow

AH: So we would kind of have this little play cutting table next to the women's cutting table, and so...and then we were always trained with chums {chuckling}, so they weren't as...you never trained on a king salmon. So the chums and in the winter time, you know pike...we learned to cut those, and then heading and gutting was the introductory people and also cutting the backbones cuz that's not as critical and they would watch, the ladies would watch to see you know who got a knack for it and who's not as good of a cutter and then you'd sort of get assigned, you'd do the salting and hanging, basically you're not a very good cutter...so there was that, that came along with is. The umm, the thing I've observed about cutting fish in this area. The traditional way of cutting the King salmon is very skillful, and you don't see King salmon cut that way in any other part of the state...Umm, it's a slab, where you cut it from the belly side into the backbone and the belly side can be very thin, separating the back bone from the skin, and it takes extreme skill to do that...and I think that's why you don't see it elsewhere, it's particular to this part of the state. Further up north they do like they call them earrings...umm, where they're hung like this on the drying rack, and it's cut from the backbone side, you see that in the south from us, but I have not seen another part of the state where they cut the slabs the way we do it here for the kings and the chums are also cut in a kite, and that's also unique to this area. So I have this...I think there's something to that, where there's some history here with the type of cutting here that isn't present elsewhere.

JE: For the kite, is that teggmaarluk?

AH: No kites are chums, but they can bet eggmaarluk or dry fish, but they have the stick, you've probably seen them when you go to the camp, chums are cut as kites, no other species...The reds are cut as earrings (Aqlitnguaqs). So when you see them hanging without the backbones...the kites have the backbones still attached to it on the other side of the pole.

JE: Wow, okay. I've seen the kites.

AH: The slabs are the King salmon (neqerrluaq) and then you also make strips with the King salmon.

DR: Is the process of taking care of the salmon your way still present today?

AH: Yeah, but it's not at every camp. It's becoming less prevalent type of cutting cuz it's hard. I mean I'm glad I know how to do it, but it's umm, alot of people have moved away from it, because cutting from the backbone is easier, so it is still present but it's not umm, everywhere. I don't think they do it that way upriver, at Red Devil...I have not seen the slabs past Kalskag. So I'm thinking, Aniak, they just do earrings. Aniak and up?

JE: Is there a benefit to cutting it that way?

AH: Yes, it's thinner. That's why you can make it dry fish, with the king salmon, it's big and it's fatty, but they way you cut it like this, you come out with a thinner slab and then use the backbone to make the kiarneq just the meats without the skin.

DR: And do you guys have any reasoning or theory behind why that is for the cutting geographically? Why there's certain ways in certain villages?

AH: I think it's because ummm, I think it's because it's an older tradition here and so the other parts of the state. Maybe I don't know if Kotzebue always had salmon or if it's a newer thing and if they're cutting it from the backbone because it's easier. I think it speaks to the age or umm, the oldness of our processing of fish in this part of the state.

CC: That's really incredible. Thanks for sharing that.

SHj: Yeah, just to add a little bit to what she said. Kotzebue, they always had caribou and other sources...but salmon was the primary source here so I think they had to make sure that they did it right cuz if it failed it could have been catastrophic. Because you know we didn't get...caribou weren't here for a while and there was no moose back then. Salmon was it, there was a lot of them, but that was the main reliable source, so they had to make sure it was processed flawlessly.

AH: And I think the scarcity of that type of cutting from the belly in is gonna become even more scarce as we are not fishing in June. Like I don't even target Kings anymore, so like the few fish I get I just make strips out of them. I won't make that kind, but sometimes I'll take a chum and cut it that way just to keep that skill going. It would be interesting to see if any other part cuts that way or even if in Russia or you know other arctic communities do that type of community.

CC: Yeah, yeah it's really interesting. Jessica and I were just in Russia, in Kamchatka and I don't recall hearing about belly side cutting.

JB: Well actually umm, our colleague, her name is Victoria, she's Native. She showed us pictures of salmon cut like that on her drying rack in her Portland presentation. She has all these pictures where it's like the entire fillet and belly all together on the drying rack that we could probably send to you just for your...we're hoping to bring them all here. Like we're trying to raise money to have a salmon conference with all our Native people and them.

CC: Is that slab, what is the Yup'ik word for that?

AH: Neqeerrluaq. Mmm-hmm.

JB: I can show you pictures later.

DR: And looking at the gender role aspect, do men get the chance of learning the cutting style?

AvH: Umm no…some

AH: There's some that are starting to, like James does all the cutting at Napaskiak. Umm, James cuts all his fish, so it's not...there's not rule against it but it's just not common. But now Stosh is starting to head and gut them, so I filet them.

AvH: Just to speed up the process.

AH: Yeah.

SHj: And even when I was smaller I'd go to my grandma's fish camp across the river and we'd do quite a bit at once...some nights, those ladies would cut up 100 and the next day my grandma would be very happy cuz that's quite a bit to handle. Like she'd get home at 3 in the morning. But I would be there as a little kid and as I got older she would need me to help her cut the bigger King heads cuz she didn't have the strength.

SH: Me, my older brother and me we'd go up and get the fish for my mom and it better be fifty, no more than fifty, so if we did end up fishing a short term, we got one 50, we'd hurry up and go load everything up over at the fish camp and get the heck out of there. {everyone laughing} Boy, she'd get so mad, that was it. But then, in no time, she'd fill up the big smokehouse. You go in there, man, best strips. But anyway, that's how it is. Everybody had lots of fish in the smokehouses, but you'll never see it again.

AvH: Growing up watching my mom cut fish you'd never see her leave while there were still fish there. She'd always stay up or not leave the table until everything's cut until everything is ready for the next step or the process. Umm, well we have sun like all night, we would be outside sitting around the fire. My mom would cut all the fish by herself and she did not complain at all. She just loved it.

DR: So any of you guys are also welcome to answer this question. What are words or concepts for caring for the land and animals in your Yup'ik language.

AH: Umm, so back at the old camp that I described, um, there was a system of if it came from the water, we would discard the parts that we didn't use on land and if the animal came from land, you'd do the opposite. I believe that was what we were supposed to do. And umm, but when we moved to the new camp, umm, my mom didn't and maybe it's because we didn't have the Elders, like it was her camp and we didn't have an older presence, umm, we would start dumping the fish guts and things back into the river. I remember my uncle, he's younger than my mom, one time he was criticizing her for not following that traditional way for caring for the remains of the fish that we didn't use, but she was a little bit progressive minded {laughing}. She's just like ahhh, but you know, that's an old way that she decided not to observe.

AvH: The first time my dad and I went seal hunting down at the coast, we came back, so this was our first time going hunting and my mom would get her seals from her sisters,

they used to live there and my dad and I came back with seals and we got yelled at because the seals weren't headed and gutted already. And we didn't know that, but we also got yelled at because the heads were sitting in our yard and there was a coastie that came to help my mom cut the fish, and we got yelled at for not putting the heads back in the water.

AH: Yeah, so I'm not sure if I have that quite exact, but there's some rule about where you get it from and where you dispose of them. For seals, like we never process them for ourselves. That was the first time we did that and we were scolded from a lady from the coast. {laughing}

AvH: My grandpa said his dad when he'd shoot a bear, what would he do to the head?

SH: What's that?

AvH: When he shot a bear, what would he do with the head?

SH: {laughing} he never told me, I never knew what he did.

SHj: Yeah, that was an upriver deal. I don't remember very well, but they did have procedures for the bear. They had the bear pointed in a certain direction.

AH: But for your question about words, it's like how to take care of, aulukiiyaraq, but you said something about land?

DR: Do you guys have concepts or vocabulary in your Yup'ik language that can relate to taking care of the land and environment?

AH: Besides aulukiiyaraq, there's not something I can think of related to that.

DR: And, what are some of the concerns you guys have about salmon fisheries today?

AH: Did you want to say something Stanley?

SH: We had moved up to McGrath up there, later on when you guys were small, and uhh, but before they got here, I was all alone in McGrath standing there and then I see a boat coming and it was pretty heavy, it came back down there and there's wow...he had forty kings on board, but I said oh, really good, looks like your dogs are gonna eat good today. The fish was so red, the big nose kings, and a big hook that goes down. Anyway, that guy came here right to me and said get your clothes ready in suitcase and get the heck out of here, this is our fish, we eat our fish. I said alright, I'm sorry. I'm not used to seeing any King salmon like that and you guys are eating it. You know the meat's kinda rotten, but the smell aint really there, it's just soft, but once they tasted special fish, they didn't want to eat that anymore.

CC: Right, right.

SH: They'd eat it for years. That's a 450 mile run from Bethel to there by the river.

SHj: See part of that is my dad is used to big shiny kings and when he first got to McGrath he never saw a fish like that and he accidentally offended the guy.

CC: Yeah, not used to seeing that.

SH: There was a place further up past Nikolai

SHj: The Salmon River.

SH: Yeah. I took this guy out we went there and we were catching fish with rod and reel, and we'd just catch and let em' go, but big ones. And then here comes a bunch of people coming down you know and they had fish camps and they were smoking that fish camp, and I said man, so okay, come back in about a couple hours and let's see what we can get. So we gave 30 King salmon, and they all smoked that fish and it's pretty good smoked.

SHj: I gotta get going here. I gotta go to a meeting.

CC: Thank you so much, umm, let me know just give you one more form before you leave.

JE: Yeah and some of these, I know you're on the board of game, and some of the questions later on like talk about how satisfied you are with fisheries management and so maybe we can talk more later if you have some more time.

CC: And this is honorarium for you and your wife thanking you. If you are okay with this recording being archived for the future, you just have to sign this form as well. And you can just print there and sign and I can fill in the rest. Thank you so much.

SHj: Yeah, thank you.

CC: Yeah, good to visit.

{Thank you from everyone}

SHj: Ana will be right back. Avery will cover for me, he won't fabricate any of it.

DR: What are some of the concerns you guys have with fisheries management today here in Bethel Alaska?

SH: Like if they're fishing out there right now today?

DR: Mmm-hmm.

SH: Uhh, they give you a shorter net, a smaller one, no more 6”.

AvH: There is 6”, no more 8”

SH: No more 8” inch. Right now it’s 6” inch. Some big ones.

AH: Ummm, for the management, it feels like there’s no consistency to the approach. So, at first when the restrictions first started, it was really frustrating because you didn’t know if there was going to be, what the schedule was going to be. If there was going to be restrictions or not, or what was gonna happen in the summer. So the first couple of years, I used to feel real frustrated through the month of June, but I decided to just, the way for me to deal with it was to not even consider processing fish until July. I didn’t want to do 3 or 4 fish at one opening and then have a closure and get three or four again later, another time because the timing for smoking and drying and umm, I like to do all of the fish consistently together, fill up your drying rack and then move the fish together into the smokehouse so that it can cure within that time frame that I can manage, not managed by other people’s schedules.

AvH: And you don’t need twice the wood to smoke half the fish. Like if it’s in these block years, if you have to get more wood than you need to do all of them at once. Yeah, so the way I dealt with it was to just eliminate my June processing and plan to do it in July, and then it’s not as emotional and I think this year with this preassigned dates for June is better than having intermittent meetings and then announcing openers all of a sudden as they’ve done in other years. I think that’s an improvement, but part of me feels like if we’re gonna try to conserve, then let’s all just do what I’m doing and stop in June, but people are...I understand that people still want to go out and fish in June. It also feels frustrating if we umm, since we don’t know what impact the other things are having on the fish...so we’re being managed because it’s a river system that lends itself to this type of oversight, but other systems aren’t being managed because they’re in different situations. So that part is a bit annoying.

CC: So you’re saying, you’re sort of bearing the burden for conservation and other groups aren’t really, yeah.

AH: Mmm-hmm. Yeah.

DR: Do you guys feel as if the restrictions and regulations off of the management of the fisheries change your values and perspectives on taking care of the salmon?

AH: Umm, I value chums more than I did before {chuckling}, so that’s one thing.

SH: You know, now the way it is today people are getting a taste for dog salmon and the red salmon make good smoked salmon. They’re really good, but not as thick as king salmon. The red salmon smoked is really good, it’s just thinner, you know. One thing we didn’t have anything to do with dog salmon. We never put any of it up. But now, lots of people are putting it up. The dog salmon is good, it tastes good.

AvH: In the early system with the block openers, or with the set net and when you're able to fish, the people and people still now, if they want Kings, they're gonna go only for Kings and nothing else. Like there's still ways to rig the system that they have now, like with the set nets, we had quite a few out there, and right now they said per boat, and whoever has the most boats, they're gonna put the most nets out there. Umm, even fishing in the non-tributaries it's a lot of work, but they still get their fish and alot of other people feel like that's not an okay system, because they don't have a lot of time and effort to go get those fish in the same area as those other people do.

AH: Umm, but when I said you know, if more people would just wait...and I think alot of people are starting to wait until July, but those people that do harvest...

AvH: They adapt quickly.

AH: Umm, but yeah, they do adapt, but it's like work and reward. It's alot more work for...I have alot of relatives that live in Nunapitchuk and they do fish in the tributaries and get their salmon, King salmon. I mean non-tributaries, and go to the coast and fish, but they also rely...I can't compare my consumption to theirs, cuz they rely...I consume way more salmon than my husband and the boys, but they consume even more than I do, so I'm not critical of their efforts to go and get the fish, because I can't compare what I eat to what they eat and how they rely on it, so they're very resourceful.

AvH: And some of them don't have access to other animals that we go to, like we go to, moose, muskox, caribou, seal and they only stick to what they know, and fish is all they know, it's all they're gonna do.

JE: So you were saying that you now fish in July. We often hear complaints about the weather in July. Has that affected you?

AH: This past year it did.

JE: Okay.

AH: Prior years, the fish has been good. This past year, it was very rainy and so it was...the first batch of fish was good, the second batch of reds have a taint to them from the weather, but they're still edible but they're not braggable. {laughing}

AvH: And like the other people that focus on fish in that month of July and they rotate their subsistence harvest to berries and all their focus goes into that. It goes from one to another and they can't split the process, one go get fish, one go get berries. It's a combined unit that everyone works together to get the process complete faster.

CC: Mmm-hmm.

DR: And you guys have originated from Bethel for a while now and you've mentioned that there are no management systems in certain villages. Do you feel as if your knowledge is reflected or being heard upon the fisheries management? Do the fisheries management recognize that certain issue?

AH: Umm, issue of?

DR: Of, how there's no management of salmon in certain areas?

AvH: Like how Bethel manages the whole river, not their own communities.

AH: Oh. I think they all feel like there's oversight. But I think they all feel critical about it too, so it's not oversight that they like. As far as their ability to participate, I think that the Inter-tribal fish commission is providing an opportunity for involvement. Umm, but, ultimately they just wanna know what the rules are so they'll know how to work around them, I think is what's happening on the ground.

DR: How can umm, your Yup'ik values or knowledge be better included in the management systems?

AvH: Thomas Thomas had a good way of uh knowing when the run was gonna come strong or slow. So at that fish fry, at that panel, he said that uhh, when the geese lay eggs, when they have more than six than that means it's gonna be a strong run, a good run going through, but when it's like, when they have less than six, like four. Like last year when we went egg hunting, there was five or four in each nest, and that run was barely strong enough. It wasn't as big as normal, so when Thomas said that he went egg hunting and he went to goose nests and there was nine and then a few days later he went fishing and he got twelve and he distributed to the Elders in the community. So with local knowledge and it's strong down there, but not as knowledgeable up here. Listening to the lower Kuskokwim knowledge is more beneficial to the run size and how to describe how the runs gonna be.

DR: Is there a reason why it's not as knowledgeable here as down as the lower Kuskokwim.

AvH: We get a whole lot of other people coming through Bethel and staying and not spreading out to the other villages of the region, so other people's knowledge doesn't stick, and it's only local knowledge, and that local knowledge has been around for generations.

AH: And we don't...it is more traditional out in the smaller communities. Um, so we're more not as umm, obligated to and not as exposed umm in Bethel. Some families are, but it depends on kinda the connection to different villages. Like for example, my niece is uh, she's...there's a lot of..she's from Tuntutuliak, this little girl, 12 years old, but she knows alot about the customs for umm maturity, for girls and I was picking her brain and she could spew off you know all these different rules shes supposed to follow. I mean,

maybe 1 of the 10 she had mentioned I had been aware of. Tunt in particular, and some of the tundra villages are very traditional and it's not as present in Bethel.

CC: Interesting.

JB: Can I ask an obvious question, but I'll just ask it anyways. Would you say that including more Indigenous knowledge in management would be a good thing?

AH: Yes, and I think even just the presence, like having that ONC program, I'm really impressed with how Janessa has worked to bring in youth into umm, the natural resources management and their exposure to the working group meetings, umm, and their engagement with camp, fish camps and users and their surveying. Having them conduct the survey doesn't feel as threatening, it doesn't feel as foreign. It's more umm, it actually is more engaging and so it really feels like the umm seeds that ONC that Janessa's planting are gonna be, create the integration with management. And it'll feel real natural, and it does not...some places where there's consultation, it's, it feels obligatory, and not sincere.

CC: Yeah, that's really helpful, thank you.

DR: With having a light hearted question being asked, what is your guys favorite thing about fishing salmon wise?

AvH: Fishing...I feel like it's a way of just having the net out and waiting until fish hit to watch the reaction of how it effects the net of like when it bobs, or if its a smoker, like when it comes out of the water and flaps around. Umm, when I was younger, I would get really bored sometimes and my dad would make me count each cork all the way out to the end. I don't know, but like if I bring someone out and they start to get bored, I tell em' to count, I tell em' to do the same thing I did when I was growing up. The people's reaction when the first fish hits and the joy of feeling that fish hit and watching everyone's reaction around it saying "Oh we got one." You could actually tell how big one fish is gonna be by how many corks it brings down. Like a big King will bring down four or five easily and everyone's reaction is like your eyes are big and everyone's happy. Like if you see for the first time you're like what is this. It's a site that not alot of people get to see, and with these closures, alot of the fishing is more combat fishing. When I was growing up, there was noone around us, we were able to fish where we want whenever we want and what size we could use. But, I was also introduced to commercial fishing with those timesets of when you have to fish and either upriver or downriver areas that are open. You would go downriver and you would see these boats off sitting on the banks, waiting for the time to hit and as soon as it hit six o'clock or whenever it opened, people would go straight out and they'd just be lines. That's the first time I seen combat fishing...and there'd be alot of people out there and that was my first experience like with the barges and having to walk up on them and uhh going and navigating around and where to go and you can go and get paid and get hot dogs and water, but there was also a lot of danger and how to manage each person's boat coming next to you, how to tie them up and always watching your looms? Making sure they're not out of the boat to get smashed in the waves. And

the bigger boats kind of had a role since everyone was in a line, like with our boat it was one of the biggest boats out there, like we'd pull like 6 people...Other people wouldn't even have to turn on the engine cuz our boat could do all the work. Since the buyers, buyers or sellers?

AH: Buyers

AvH: Since the buyers are like having, like leaving there'd be only a few out there, like 2 and you'd see one line all the way up around the bend. It would be as long as you could see and every once and while we'd get caught in that long line and be in there for hours and having to come home late. Now you don't see that type of boat activity coming up and down the river. It was just really cool to see all the boats in line, but when we didn't have a good run or good catch, we would just take it home or just give it to someone. We weren't gonna wait in that line for too long.

AH: What do you like about fishing?

SH: I like to catch it. You do the fish cutting. That's the way it was. We'd catch a fish, she cuts em. The strips...when I first started fishing with my older brother, he'd just get out of there and (can't understand) cut the fish..And I think how the women did that. I never seen no men cutting fish at that time, do you?

AH: Not then, but now a few. Umm, I forgot what your question…

DR: What is your favorite part about fishing or about salmon?

AH: Umm, when the weather cooperates, it's really nice. Umm and…

AvH: So you know like the best time to fish is like when it's rainy and stormy and windy, especially with the south winds because it brings most of the salmon into the river and you're able to catch more. Fishing in that stormy weather, how did that affect you?

AH: It's very stressful when the weather's bad. You worry that you cut all that fish and its gonna not dry. When it's south windy and rainy, like we used to put tarps up to try to block the wetness from getting on the fish...People make fires under the fish when they're outside to try to let them dry. The weather can be a very stressful part. That's why when it's good weather it's like relief. Umm, and then the other stressful part is the flies.

CC: Mmm-hmm.

JB: Umm, you said when sometimes you build a fire under it. Where I'm from, we always build a fire under fish, but here you wind dry it sometimes?

AH: Mmm-hmm. So I know my dads partners, Georgianna from Rampart, and so I know they go directly into the smokehouse, so I've wondered about whether or not what that

would do to our processing and if it would work here, but we've always air dried them first and then move them in.

JB: Mmm-hmm. So just to tell you a little bit, umm about where I'm from, which is farther upriver from Georgianna but closer. The weather is so hot in the summer, it's like 90s. It's like a fire, if a match hit the trees, it would just torch the forest, so it's not really humid ever, so I think that's why they go right into the smokehouse. Usually how we do it is we filet cut what we're gonna make you know, say you dip it in the salt water and we have one rack to just let all that slime off and then we move it into the smokehouse, but we don't have the wind that you have, so that's the difference, so I think it's all the environment tells us what we're gonna be doing and sometimes if it's raining where we're at, like we just decide if we're just gonna put it away and filet, like if it's a big storm, we don't have the wind at all to help us, but we have alot of flies and hot weather.

AH: Mmm-hmm.

AvH: Did they have...so I know McGrath has not like very windy and has big trees. Would they dry their fish in McGrath or go straight in the smokehouse?

SH: I never seen them do anything.

AH: Just freezer?

SH: But you know what they like is what they got me in McGrath. The Sheefish, they love that Sheefish, and I said wow. All McGrath just loves Sheefish. And all I could think of was the rain, you know King salmon, I think the sheefish tasted better than the Kings and they even smoke em' outdoors in the willow house they make, but there's always sheefish.

JE: That's really interesting because we deliver sheefish here and it's hard to find people..alot of people are actually allergic I've noticed…

AH: They're saying that {laughing}

JE: We gotta find people, call people to see who wants sheefish. {laughing}

SH: I didn't eat no sheefish when there was king salmon. You know when I was growing up, never, I wanted king salmon.

CC: Yeah, well thank you guys so much. Are there any other things that you wanted to share on any of this with us that we haven't asked about?

AH: Umm, the last, not this past year, but the year before was the first year that Stosh actually didn't do any fishing at all. Gauge and Avery went out and got all fish for me, and that really felt like umm, man, earlier I think you were asking about kind of rituals or things, and that really felt umm, accomplishing as a parent and as a family that and I you know

talked about it to other people when they would eat fish...I'd say you know Stosh didn't even fish at all, it was all the boys and it felt like we umm, could mmm, sustain, that the transfer of knowledge had occurred.

CC: That's awesome.

AH: Mmm-hmm.

AvH: So my dad doesn't really like to stay at fish camp but my mom loves it and he is rarely there when we're all there, but when we're never there he's always there. {laughing}

AvH: So like the first thing he did was with the rainwater, he made a running water system made out of like a CVC pipe with gravity and levers so just to make it more comfortable for my mom to be there because she loves it there. And with him never there and with us being small, like carrying up the water from the bank, it was like a pretty raggidy bank, it was hard to walk up with it, but now there's stairs. It was just like for looks for Stosh. He actually had a water pump so he'd just turn on the generator and the water would go from the river into the bucket where my mom needed it and so sometimes my brother and I wouldn't go to fish camp with my mom. She was pretty independent and she'd go by herself and stay there by herself so when she, she didn't have to get her own water, the water pump would transfer from the river straight to her tub, less work. So I feel like the traditional way of having the way of kids go get the water, and now we just turn on the water pump and it just goes straight to the tub.

AH: Yeah, we incorporate modern technology…

AvH: We also have that water pump for like in the summer, so when Ana and us bring our little cousins, we also have a water spray for them to play with.

AH: But there's also people use strippers not for the filets. When you were asking about general rules, when I've seen men get more involved with the stripping part. Like the women will do the fileting and the men will strip them cuz it's like a machine.

AvH: If you didn't cut straight like you're getting booted out of there, cuz I know Bugs used to be the strip cutter cuz he was the straightest cutter out of all of them.

AH: And there's sometimes Jayden, there's one of the boys would always hang out with me at the table and he'd touch the fish. So I always thought I can teach Jayden because he has an interest in that part of it, so it's not that they were prohibited, but it just sort of who has interest in what.

AvH: And we get yelled at really hard with the fish, cuz if you cut too low or too deep or too shallow on strips or slabs or kites, we'd get yelled at. If you cut it through, that could actually have the umm, drying of the fish to fall off from the rest of the skin. And also I think me and my brother love to hunt so like we'd always have a bee bee gun or pellet gun around the fish camp and were also like the protector of the fish so if a camp robber

would come, we’d shoot it, cuz my mom would get mad at it cuz it’d pick at the fish and the meat and it also caused it to fall to the ground.

AH: Yeah, it does. The birds are another...umm, problem. Cuz they will pick at the neat.

JE: I wonder if you could try that netting that Aaron Moses...do you have some?

AH: No, I mentioned it last year. Yeah, I wanted to try that.

JE: We heard it worked against the birds that were picking at the fish.

AH: Good, thank you.

CC: Thank you so much for all of your sharing and visiting. It was really helpful and I’m honored to meet you. Thank you so much. Anyone else have anything to say?

SH: I was thinking of the Elder fish person over here. This fish is even better than King salmon, the (?). And our...my cousin, fourth grade, we’d run up to the lake and we’d just catch our fish. It’s not no whitefish.

INTERVIEW PARTICIPANT: ROBERT LEKANDER

Interview Date: 6/4/19
Interviewer: Courtney Carothers, Jessica Black, Avery Hoffman, ONC Interns
Location: Bethel
Home community: Bethel

Robert LeKander [RL]: When I grew up it was, I listened to the old folks, but then the science part come in too. And they, Fish and Game or who were educated would tell me "No, that's not right." Cause people used to tell us that the fish go up under the ice at times when the river don't break up early and Fish and Game guy said "No they can't. They wait for the temperature just right and then they'll go up." I- so, and a lot of other things then we get to me it's who do I believe then? Science or them days even everything, even in school I was told that Native people didn't go to science school or any type of college and so to me which is right and who do we believe? But over the years I've looked at things. I've seen guys pull out a net with a king in it from under the ice going up and it's in the spring. And what's that? Lot of other things (inaudible) that kinda. When I was growing up who do I believe? I remember my grandma used to go get something from the - in the brush that were growing fresh in the springtime to put on the end of the nets, but I wish I knew what it was. I didn't. What's the difference? Then come to find out our fish navigate in the ocean and it's through the north pole south pole I guess then when they hit the stream out there the flow of the rivers their sense of smell comes in and they smell where they been, where they were born. I'm assuming that's what they - that's what old folks used to tell us. Anyways these first ones that fat ones are always the ones that go up to the headwaters of the Kuskokwim and I believe them. Mostly it was my grandma because we grew up with the – the old men died off. If you were 40, 40-50 you were an old man, but them days everybody was dying off. TB and what not, but we pretty much were on our own. It's what we had. Now it's so modern with these outboards and fancy boats and nets longer than you can see, well but if I had my (inaudible) I would still cut that net in half in and fish. Maybe we'll save more fish going up. And another deal is the fish spend their time only a year here and then go out in the ocean and they spend seven, six years out there, and I ask why you know. They're doing something out there too. Their bycatch and what not they're supposed to have somebody watching them but seem like we're always - the people on the river are the ones they hit most to conserve, and it's – I don't know. When we went to school they said history, math, and English were the main subjects and I don't think nobody seems to be looking back at history. History always repeats itself. What happened on the Grand Banks of Newfoundland there and all the way down the east coast and up the west coast, and it didn't go west no more. It's go north, go north. The last frontier supposedly, and I've been over to Bristol Bay canneries and I talked to some of the fishermen. They were company fishermen. Most of them were from Astoria cannery. It was Columbia Ward Fisheries, which is Bumble Bee Seafoods now, and I good friends with the fishermen there. They were called company fishermen. They fished for the company and the company furnished them everything; the boats, the nets, and food, and place to bunk when there's no fish. This one guy said so I asked him what he do in the winter? "I fish." Said he goes down west fishes all the way up and by

springtime he's up by Astoria and then it's time to go to Alaska so they ship him on up to Alaska to fish for canneries there. Now it's even lately here from the guys on the coast I've been hearing there's more lights out on the ocean at nighttime than there ever was cause it's fishing boats are going further north and north. What happened on the east coast, happened the east coast, and the Indians on the – they can't even get fish I guess down there. Columbia River and them rivers down there. What's happening up here now? They say global warming. You know the other day I told you what happened with the whales. They're getting hungry and skinny and dying off. What's happening to them puffins. What's happening to the big trawlers out there. Those big factory ships getting all the fish. It's just like if you put a Walmart here what's gonna happen to the family stores? They disappear and they get a Walmart taking over. What's happening out in the ocean is those big factory ships are the ones taking overfishing now in this pretty much indiscriminate fishing – anything in the trawl's dragging down there. (Inaudible) a clean amount out there. The commercial fishing on these rivers is going here, it's gone here now. Yukon it will eventually go out too, but they still got some commercial fishing, but it's history repeating itself all the time. By the time I'm gone it'll be no more. I hope not. Maybe we caught it early enough for the kings, but eventually. You remember that time we were on strike for chum salmon that were $0.25 a pound. Now we're fishing for even $0.05 we're selling them, but at that time we made a big row and they were catching down there at False Pass and around that area and they did something and chums are back now too. Reds always – like out in the ocean I guess. Yeah that's all of it and what else?
Jessica Black [JB]: Well we have some questions and Avery is going to help me interview you today. So we could go through the questions but everything you're sharing is just really helpful. So we could start asking you a few questions.

RL: Mmm-hmm.

JB: So Avery is going to start if that's okay with you.

Avery Hoffman [AH]: So my name is Avery Hoffman and today is June fourth, 2019. We are in Bethel, Alaska at the ONC, and do I say my age too?

JB: You can if you want.

AH: I'm 19 and I am Yup'ik and the first question we have is please tell us a little about your background like where you're from, how did you grow up, and what connections do you have with salmon?

RL: Yeah I grew up here. I was born in Bethel. I grew up here in Bethel in '40s. I was born 1943 and I grew up on fish and wild game. We had while the steam ship, paddle whalers (not sure hearing this correctly) were disappearing then and boats from Seattle would come up. Big ship and park across the river. Across the river was the main port. They had army airfield across there and everything, and that was the main part they unloaded all the freight once a year that ship would come in and we'd get our produce. What they send. Everything was pretty much in salt barrels and canned stuff started to come. Otherwise we maybe plane once a year after the war – Japanese war. Then the main

town was right here on the riverbank all the way. (local place name?) area was this way and we were cross the slough. (local place name). It started you know by the sloughs, the rivers, villages would start along the river. They get fish and whatnot from the sloughs that come out. Fish that come out of there. And we went to old log cabin school down there and I forget what year, I think in the ‘50s a new school was built. Kilbuck School (check spelling of name) and it started growing. I remember when there was 800 some people here. That was a lot of people and everybody knew everybody else you know. Also they had I remember they had alcohol then too. It was controversial. They, Johnny (inaudible - owned? the store over there and where big Chris lives it was a store. Goodrich I believe or somebody owned the store and they had cellars down there. Guys go down and bring up bottles and sell ‘em and nobody said anything about that in the old days, but now it’s changed, but five horse and there horse- so five horse was a big motor them days when I was growing up. I still remember my grandma say if we run out of gas we’ll just put up a sale (inaudible – Yup’ik word?) just with doors and put the canvas up and go and they said, “We got (inaudible) back.” She thought nothing of it. That’s the way they did in the old days. Time was nothing. When we get there. Now it takes only a day with a outboard, and then I grew up I had my own dog team too. Just five was used to go hunting and fishing upriver a ways and take me about two hours, hour and a half. Now it’s a snow machine and take ten minutes to get to where we go. Modern. Modern times now. It’s same way with all this fancy equipment. We used to go hunting jack rabbits with dog teams and we’d see a lot of ‘em even up here in the river bank my uncle set snares. There’s nothing now. No more jack rabbits. Pretty much cleaned them out with the snow machines. It’s modern technology in effect. Now they’re doing fishing with these big outboards and nets you can span the river with. In the old days we had wooden boats and small engines and we’d oar all the time instead while we’re fishing. The old folks told us we’d scare the fish when our nets were little longer than the boat and that was a long net. They were – before they were making their own nets in the wintertime with string and whatnot but then when they started going over to the cannery the cannery people would get rid of them old nets the fishermen used on the Bay that had holes and whatnot, and people even my uncle they asked them and they’d give it to them free. Old webbing and they’d work on them here. Cut ‘em shorter and they’d have nets all cotton nets with wooden corks and lead, the leads they were in little bricks you’d pull them over and hammer them on the rope on the bottom. Then we’d have to every time we’re done with the nets we can’t leave ‘em in the boat we have to bring it up and dry it otherwise it will rot. Cotton. I remember when they holed up – Eddie Hoffman. They lived not far from us over in the slough part there. “Robert you get a net like I got.” Nylon I believe. “Yeah look at this I leave it out and do not take it up every time, even when it’s wet. Leave it in the boat.” Wow, but then the year after I asked him, “Where’s that new net you’ve been using?” “Oh it’s wicked. Wants to break now.” You know you can’t – that nylon and stuff you can’t leave out in the sun and he never covered it up and it just – but he eventually found out. Everything’s changed now. Everything going at a fast pace.

AH: You talked about that big barge that would come through once a year. How?

RL: It wasn’t a barge. It was a ship.

AH: A ship.
RL: We call ‘em tramp ships in the military they would tramp if I wanted to cross the land taking one of them.

AH: How was the river then? Was it skinnier or what did it look like?

RL: Oh it was - right in front Bethel a guy used to swim across the river it wasn’t that wide. Now it’s so wide and wider than it used to be, but it’s about the same, but we used to have lot more snow in springtime you know where Swanson’s store is from our house across the slough I used to boat right and go over and tie up right on the railing, deck railing, and walk into the store.

AH: With the flood?

RL: Yeah with the flood. Now there’s no more snow and ice is thin. We used to hear that ice break. Four to five feet more thick busting up. In the old days when the army crew they had one of them army plane two prop (inaudible) come and they’d sand all the corners. The corners of the river up here by Joe Pete’s around there they’d put sand on the ice so it’ll soften up and melt cause that’s where they jam and the flood would come up and they’d do it all over. They were doing dynamite out of them too that one year and everybody said, “Oh you guys are killing the (inaudible) and all the fish underneath.” So they quit that.

AH: And Joe Pete’s was the old channel?

RL: Yeah that was the main, main channel around. It’s got a stray in the hard bend there and that’s where all the jam would be and right down here jam there it flooded everything around here. This, yeah, and the old days were – they’d even put oil on the roads to keep the dust down, and they started putting oil on the lakes so they’d kill the mosquito larva and it’d put a sheen over and the mosquitos would die off.

AH: Do you know if it would affect the birds?

RL: I never thought of that. Them days we couldn’t in the springtime they closed off what Mexico, United States, Canada, and Russia had a treaty. They said we were taking the eggs here and killing off, I mean the birds were getting less and less. The geese and the ducks and whatnot. So they close it off in the spring. That was our first meat pretty much in the springtime. We’d wait for them birds. Eating dried fish all winter. Fish, fish and them birds come. Wow we gotta go get some, but then we were hiding from game wards. We’d go hunt two o’clock in the nighttime and come back at night every time we’d hear a plane we’d hide.

AH: And that was with lead bullets?

RL: Yeah everything was lead. The only kind I had in the old days, but now you know, after (inaudible), there ain’t that many people in Alaska them days if the villages along the

coast where they left and up here and how can we decimate them birds. What was happening as I see the people down south selling the birds to the restaurants. They'd have piles and piles of them and they were getting less so they had to blame somebody and they got Congress to close the hunting up here. That's what I believe happened. Same way with the reindeer. They had the (inaudible- group?) from Lapplanders (Norway & Scandavian countries) come on here in the old days and they had herds all around here even where that high school is up there. My grandma used to tell me there used to be corrals back in there, even with Kwik/Gweek now up there we'd walk on the tundra picking berries sometimes and I'd see these old posts on the tundra and there's no tress around. So guys what are these and that was where they held reindeer. Those were the old posts that they put in and put these corrals to hold the reindeer and they were still up there. They had whole herds then.

AH: Did you ever see the reindeers?

RL: No. They were little before me, but my grandma used to talk about. She'd say they'd give 'em some, dole 'em out. The old saying never say no to preacher, teacher, or BIA man. Cause in the old days the teacher and the preacher knew who all the good guys were and they'd tell the BIA man, who gave out food and stuff, and if you were bad – didn't listen to the teacher or the preacher you hardly got anything. So that's what my grandma always told me. Never, never say no to them when they tell you something. There was an old standing joke too that one guy said to you was watch white man talk to I guess preacher was talking to Native guy. Native he didn't understand, he didn't understand English so he'd shake his head and that guy, the white man would really get mad. Jump up and down and wail his arms around, but you watch him and said yes, that's nod his head, the guy would be good. So what they told 'em old saying never shake your head. Always nod even if you don't understand.

JB: I'm really enjoying listening to your stories Robert.

RL: But you know it's always – even that reindeer what happened they had Congress shut 'em down. They had no more Lapplanders and they couldn't own them. Only Natives. And they knew the Natives weren't no herders. They were from spring camp to summer camp to fall camp. They lived off the land. They knew that. The guys, what the story I got I understood was cattle ranchers in Texas were scared reindeer meat was gonna affect their sale on their beef so they talked to their Congressman and they put it out that only Natives can own the reindeer. Nobody else. The Laplanders (spelling?) basically no more. Old Laplanders (spelling) some of them are still – I mean their offspring are here. Like the Saras and Speens area, but there's always some blockage. Yeah what else?

JB: The next question is how did you learn to care for salmon when you were growing up?

RL: Well it's not a thing – we'll start, we get the smelts first, then we fill up a boat not the tub or anything, tarp, old tarp in there. Tie it off and fill the boat up with smelts and bring 'em to fish camp and all the women would collect 'em. We'd take 'em up in buckets and

dump in the area we'd make an area and get the sticks, willows, and the women would all get together and string 'em all up. Used to be fish racks would turn silver from all the smelts hanging. And in them days smokehouse you couldn't see the end of it and I have a bad habit. My wife says, "How come you like the big smokehouse?" I don't know it's just old habit. When I was growing up we had smokehouses long and fill the back and up the midway then back then we'd take 'em out and put 'em up on all top. They were already smoked pretty much. The old people, the old women didn't do much. They'd watch the fire smoke in the smokehouse they'd smoke – take care of it, cause that was our food. Even the dogs that was family and dog food.

AH: And what kind of smoke did you use in the smokehouse?

RL: We used cottonwood. I still – that's all we use. It was a milder smoke. That tundra people use that other alder and whatnot cause that's about what – there is no cottonwood up tundra way, and even when they come down you could taste that bitter taste from the fish a little too strong, but we use that mild cottonwood. Milder taste. That was good. There's an old saying too you know, what you eat what you are.
JB: Yeah. After the smelts is that when the salmon start?

RL: Yeah. King. Kings. We didn't get – when I was young we didn't seem to get too many kings. We had it seemed like we had mostly chum, chum that's you know Bristol Bay and later on the bigger webs fish, web come in and then we start eating the kings, catching the kings, and this river here is mainly a chum river. Like the Yukon is king, the Yukon king and the Bristol Bay is the red salmon, and Kenai and whatnot. Each river has its own species.

JB: Yeah I'm from the Yukon way way far up so we only get kings and then fall chum. That's it. So we gotta, we get some kings, but we also the first run of fall chum we also eat those too. There still pretty good. The second run is more for like our dog food.

RL: On chums?

JB: Yeah.

RL: They're fatter then. Yeah we – I wondered about that. Must be two runs I keep hearing about fall run and they commercial fall run I guess so one of the runs I guess.

JB: Where I'm from we have no commercial fishery.

RL: Yeah that's way up interior.

JB: But here's For Yukon. If you go down maybe toward Rampart and Tanana like they still get like silvers, but we're I'm at we just get the early fall chum and that's what we call silvers, but they're not really silvers. They're not – they're still good to eat though and that's kind of what we rely on because of the king run has been so low.

RL: Yeah. Yeah it was all over the state I'm wondering what you know the kings just drop drastically how many years ago and what did that tell you. I don't know. Some place they ain't in the rivers I believe.

JB: The way you grew up just telling us how you cared – how you first got the smelts and then the salmon came. Is it different now than when you were growing up or is it similar like you know?

RL: It's pretty much similar, but to preserve 'em we take and put 'em in our freezers. In the old days we pack some in drums and cut 'em up and try to keep 'em in a dry place, cause they mold real fast, and we'd leave 'em in the smokehouse. Lotta dog food. That was mainly dog food we left in the smokehouse and we'd go up and with sled dogs go get 'em. The ones we were gonna eat we'd have to pound 'em on the post to get the mold off. My uncle (name?) said, "Go on. That's penicillin. Eat it. Good for you." I never know yeah wonder drugs have come out over the years. Penicillin and all that. Yeah we – in this – we never, we didn't know what a padlock was. Everything just turn it and leave the door closed and them days, them years there was no black bear hardly any moose. You see a moose around here it was everybody gets food. They get it.

AH: Were there rules about harvesting?

RL: Yeah. Harvesting birds and muskrats and mink and what. When they're having their young that's it. We leave 'em alone. And then they'd know pretty much, the older people would tell us, "No more. No more." Cause their fur was getting bum anyway and they were having their young so save. They tell us, "Save for next year. Leave 'em. Leave 'em alone." And we'd harvest other. Like fish would be here too we'd go spring camping in the spring. The old guys used to tell us, "The loons are having their eggs so we better get down. The smelts should be here." So we'd come down. Couple times we stayed edge of the lake for a few days because we couldn't cross the lake was still frozen. The slough. There's sloughs right there where we'd go come down. Frozen and we'd have camp (inaudible) guys I remember and we'd have just fact little village. Ten or so tents waiting for that ice to melt enough so we go across, go on down.

JB: What about rules with salmon? Like harvesting salmon like were there certain rules that your elders told you to follow?

RL: Yeah. Take care of what you, you know what you catch. Don't – even the bones or things when you've cut 'em put 'em in certain place. Don't put 'em in the walkways or anything and try to keep 'em clean cause that's your food. Our food. Never waste either. You're not gonna use 'em don't try to catch 'em.

AH: Where would – was there rules about where you could fish?

RL: Yeah. The old guys would tell us, "Don't set there that's somebody else's area." Or the villages. If you gonna hunt around there ask somebody from that village because

that's their area, hunting area, and you stayed where you're area is. Hunt in your own area. We followed that pretty much. Respect other people's places.

AH: Do you still fish in the same place you fished today?

RL: Yeah. Right up there at Joe Pete's, even its disappearing. Oh I was gonna tell you you've been catching lots of hair up there. Sasquatch hair down in the net. I noticed that this last time. And I said guess I'll go out further, but still there was that black hair on the net. That river is dying. That's what happened down here too. I don't fish in front of Bethel. I used to when it was good and I started catching that black stuff down around the bend by Standard Oil down that way. Current was enough that they were growing (?) stuff down there. So it's dying out down here too. Now it's up Joe Pete's area it's dying out. Going through you know straight (inaudible), straight river now and then no more. That river changes all the time, but yeah it's pretty much same place I fish up there. No snags, but I always liken us to the old farmers. Let them first guys go clean the fields out, get the snags out and everything. You snagged before some that I've had to cut sometime half the net. Can't get 'em out. That's not cheap either.

JB: When you were growing up if you like broke a rule or you didn't do something like what happened? How did like how did your people enforce rules?

RL: Yeah well I know in the villages they'd tell (inaudible several words). Three times she (?) told him, "Don't do it. Don't do it." They tell them to get out. Get out of the village, but here I never – like I said older people, grandma was there and we never broke too many rules, but I've heard about villages they kick 'em out. Banish 'em. The older folk. And when we were young if you go in a house in villages you don't walk in - you stand by the door until the owner tells you to come on in and don't be talkin' unless you're talked to.

JB: Yeah that's even when I was growing up you waited till someone invited you in. Is it different now?

RL: {laughing} Yeah. I get a kick out of guys well it's – the guys that tell these young kids you know, you know don't play on the road or don't be coming around my yard and taking my stuff. Hey. Them kids will give them the finger and laugh and walk off. They know they can't be slapped or anything done to them, cause they call the cops or something. In the old days we respected everybody even if we found something on the trail like nice big shovel or something – if you didn't know whose it was you stuck it in the snow right by the trail. The owner eventually finds it or if you know whose it was you put it in the sled and give it back to them, and if you gonna, you always ask the owner if you gonna use something of his. Axe or tools or anything you ask before you touch. Now I have the same problem I don't know where half my tools are and then come the next day find out that the grandkid has it or the daughter took it or they used it, but frustrating looking around trying to find it figure out somebody stole it or what. Can't leave nothing laying around no more. Even the houses we never used to lock 'em. We'd go fish camp or hunting just latch it. Leave it.

AH: What are the words for caring for land and animals in your language?

RL: Nuna auluku/aulukllluku nuna and it's your ground you take care of it and I still remember my grandma every time we went picking berries or when we'd stop on the tundra she get some food cracker or something and dig in the ground put it to the, give it to the nature I guess. Give it to the ground. We take from you we'll take care and don't leave trash and stuff around.
[unknown female]: What's that word again Robert?

RL: (Yup'ik word needed here). Take care of it. It's your land. Our land. My – I still remember the people grandma and them they say this land is everybody's not no land claim and whatnot. Berry picking area it's everybody's. Hunting area you watch what but berries and whatnot you go pick berries. They didn't mind so much the villagers.

JB: When you were like you said when you were with your grandma and she was picking berries or plants what did she leave for? Did she leave it for?

RL: Even piece of dried fish or cracker or something buried all the time. Just I don't know she was appeasing the gods. I tell her old foxes can come and dig it up and eat it anyway, but she never, never mind. What she did the old people did all their lives. Tried to give back what they take. Respect the land, animals, and whatnot.

JB: That's kind of how I was raised too to leave something small even like just whatever you have. Something. The next question is how satisfied are you with fisheries management today? Do you feel like your knowledge and values are reflected in fisheries management?

RL: Yeah they seem to. Today they seem to listen now, but otherwise seem like when some disaster happens and everybody seem to go in but before they never cared too much they just did, but when something happens everybody's in there on it. Now our ways and means are disappearing everybody's writing about them and whatnot. Before like I say it seemed like this is generation behind. The guys were knowledgeable when I was growing up. And I got to the point there times I said who do we believe? Science or Indigenous – the old people. They used to, the old people never went to school. They don't know what they're talking about. You heard that quite a bit in the old days. And lotta people went to these Catholic schools they send out. St. Mary's and Holy Cross and down to Mawah (spelling needed) and going high school down states, and lotta of them lost their ways you know. Some of them even quit. Hardly understand Native language.

JB: You mentioned just before the interview that you are on the Fish Commission here. Can you tell us a little bit about your role on the Fish Commission?

RL: Yeah I've been here (inaudible) years since Greg passed away and I've been on it and it's my role, well, we get together with upriver guys and Kwethluk our area, and then upriver guys their area, and James Nikolai I told him that one year, last year that there'd be more fish and he was right because he didn't see the fish going up into

Kisaralik/Kasigluk and Kwethluk. It's another area that they were fishing over here catching these ones that are going up further and they'd said they never catch nothing over here yet. Said they still coming. Told him and he was right and (inaudible) three, four rivers, three rivers there right there in that Kuskokwak slough. There's three of them and I'm sure there's lotta fish going up in that way. He noticed that they weren't catching until later on and main run wasn't here yet. They listened to us and I told them that I usually start fishing second week of June used to, but now this global warming I don't know what's gonna happen. Maybe we'll fish them earlier. Second week of June I go out and we'd cut 'em up right away because weather is still nice. Usually end of June it starts getting muggy and the flies come out and harder to dry any fish certain times of the year we fish for them. We used a lot of reds too now. When I was growing up there was hardly any reds. If you caught a red there you were high boat or what you call it, wow. I still remember Teddy Samuelson he's gone now. Come wave me over. It was them wooden boat days. He asked me, "Robert what kind of fish is this? Looks so bright." Must be a female chum they're usually brighter than the males and then another old guy he come around he, "That's a sayaq (red salmon). Hey you guys caught a sayak." He told Teddy, and it's (inaudible) a red salmon maybe word for red salmon. "Wow you guys catch one." Now there's so much of that red I don't know what happened. This is a chum river but basically I'd rather have reds than chums. Dog salmon they call it. It's for dogs the chum. Used to go (inaudible name?) Samuelson's store and they'd have 'em in – they call 'em kites, kites (spelling needed?). They'd be stacked I forget maybe 25 or 30 all stacked and tied up. They'd sell 'em that way. I think $5 or $10 for dogs. They'd just throw 'em in the sled and they'd have 'em when they go some place hunting or sometime a lot of them old people, I mean the guys didn't catch many so he sold 'em.

JB: They were in a stack?

RL: Yeah they'd pile 'em up. They were the kite form, that's the way they kept them dog salmon anyway, and then they'd stack them and tie 'em up. Guys buy 'em for dog food if they were short, running short, or when they're gonna go on a trip they're easier to handle and they're not bulky.

JB: Lighter on your sled?

RL: Yeah. For the dogs to eat. They'd have the – yeah. It was when I was growing up I used to want to go with my uncles to beaver trap. They'd go up in the mountains and that was you could only get ten beaver per person in the old days. Go up in the mountains, get 'em. I always wanted to go with him. Uncle, "No you go school. School." Couple weeks or so come back.

AH: Do you know if they would get ground squirrels also?

RL: Not too much around Bethel. It was the village Kwethluk and them guys were always going up getting parkis squirrels, they call 'em parkis squirrels ground squirrels they make parkis out of them. They were nice and you dry their meat too and eat 'em. Dried squirrel. With relations up with Kwethluk go up there, Slavic Russian Christmas time stay with

them. First time I tried that squirrel made my huppa's guy that was (inaudible), "You better watch it cause if you not used to it you gonna have the runs." I didn't believe him. It tastes good dried squirrel meat. I found out that night my system wasn't agreeing with me.

JB: Your uncle was right.

AH: What are the Yup'ik values, knowledge, management, and governance mechanisms that will improve the long-term sustainability and equality for the Alaska salmon system to benefit all?

RL: Conserve, conserve, conserve. A lot of people are going to red salmon now and they say well we'll use a smaller mesh nets and see how it goes from there. They're changing over you know, but like I said in the old days we'd catch king salmon was main food we had. Dry fish. There was hardly any reds but now we got quite a few reds and we're kind of changing over. I haven't used my king gear since they started. It's eight and a quarter mesh, bigger mesh for kings and it hasn't touched water for how many years now, but I still hold onto it hoping eventually we use it, but it's slow, slow maybe they caught it in time. I don't think so. It's big factory trawler ships out there that are coming up north and they're going further and further north. I'm afraid they're gonna be more whales dying and more birds dying. No food. But we can try. Smaller, smaller mesh them reds are good but they're smaller.

JB: Well we don't have any more questions but is there anything else you'd like to add Robert?

RL: {pause} Yeah there's something but it's not worth talking about, but yeah everything's changing. Now it's, everything's getting mechanized you know the computers and whatnot. Maybe I'm biased because I don't even know how to work 'em, but it's getting to the point now I'm praying even my grandkids don't – where did the sun come up? "Oh I gotta look on my phone I'll find it." Where did the wind (inaudible). When we were young the old people used to tell us, "You go out first thing in the morning you can tell pretty much what the weathers going to do during the day so you plan that way." It was you know, but nowadays they go out I don't know. They depend on their phone I guess or mechanism. One time we were coming back when brother was alive we went moose hunting over the Yukon and we got late start coming over this way through the back trail, tundra way, and his goddarn GPS was going haywire. I kept asking you know we'd stop every so often and I know which way the wind is blowing cause we going down south it's blowing from the south. Take goggles off and glasses off that wet snow was too much in the wind and he kept veering off. Finally I stopped and "Eric how come you keep move – turning." He said, "I'm going with GPS." Something wrong cause the wind is from the south and that's where we're going you know from the Yukon over this way. I said that's where we're supposed to be going. And he checked his darn GPS the batteries were going out. So I said, "To hell with that. Face the goddarn wind in the south. We'll eventually hit something. We hit the trail up by Kwik and followed that rock trail that he had – and tail, license plate tails those reflect good in my lights and he didn't even through that snow see them, that reflection that was a trail. We ended up hours getting scared. My gas was

down on E all the time back. We stopped one time to look around and we heard a plane, big one, one of them big planes for scaring up, we were behind tundra ridge going north, I mean going east that way, and I said, “Oh hell.” We turned around and come back and made it there.

AH: You were on the wood cutting trail?

RL: Yeah. So it was snowing so much at nighttime, but (inaudible) sucks. Use your head sometimes and you’ll make it. Otherwise it’s I guess it’s good thing them computers and everything, but what does it do after a while you’re another 100 years your brain ain’t gonna be there no more. It’s gonna be on your fingertips. One time my grandkids come home, “My teacher told me to get a computer to calculate.” I said, “What’s wrong with that one on the end of your neck up there? Tell your teacher that.” I mean we didn’t have the (inaudible) we were using our heads all the time to add, and subtract, and whatnot. I mean it wasn’t fast but you using the noggin up there, and you get in situations now I notice you don’t know what to do. I said yeah, but it’s mostly through experience on my part too. I tell ‘em when you’re like this you think first. Think. Think.

JB: Yes.
Unknown female – ONC Intern?: Robert would you mind if I took a photo of our group here? Is that ok?

JB: Might have to stand up to get us.

AH: Smiling.
(photo being taken of the group)

RL: Should have fixed my eyebrows. {laughing}

Courtney Carothers [CC]: Yeah you guys want to get in. Thank you. Thank you for letting us have such a large team interview you. Awesome. Thank you.

JB: Thanks Robert.

RL: Yeah when I was getting my passport I said, “How come you want me to take my hat off?” And they had my glasses off too cause there was too much reflection and I looked at the pictures and it don’t look like me. My hair is standing up and if I knew then I’d put it up a little bit.

CC: So we’re gonna have him sign that one and he can keep -.

INTERVIEW PARTICIPANT: MARY PELTOLA, MATTHEW KAPSNER
Interview Date: 9/20/19
Interviewer: Janessa Esquible
Location: Bethel
Home community: Bethel

Shorthand key:
( ) Note about what is happening during the interview, unclear of word, or spelling question
[ ] Words not said but added for clarity
{ } Action
Mmm-hmm, uh-huh are affirmative
Hmm-mmm, uh-uh are negative

Janessa Esquible [JE]: Hello, my name is Janessa Esquible and I'm with Orutsararmiut Native Council and we're here in Bethel at the Kuskokwim campus.

Mary Peltola [MP]: I'm Mary Peltola, I am the interviewee and lifelong resident of the Kuskokwim River and this is Matthew...

Matthew Kapsner [MK]: Yeah, I got it {laughing} Matthew Kapsner yeah…

JE: Okay, today is September 20, 2019 and we're here to do an interview….

{Janessa goes over project overview and authorization forms}

JE: Just tell me a bit about your background, where you're from, how you grew up and what connections you have to salmon.

MK: {Whispering} These are hard questions {Laughing}

MP: Okay, so my name is Mary Peltola. My Yup'ik name is (Akasaq) It's.. It was my grandma's name and she died right before I was born. Um, my mom is from the village of Kwethluk but in working with the fish commission and getting to know Robert Lekander better, he is my mom's first cousin. Um, he told me that my mom's grandparents and his grandparents lived year-round at the mouth of the Kuik River. Which I didn't know until I was like 45 years old. And then it made sense because that's where we've always fish, that's where my dad uh commercial fished and we had a tender boat. My.. All my mom's brothers fish there subsistence and commercial. Um, everybody in our extended family fishes there. So you know that made sense. So I grew up commercial fishing. Um, I started commercial fishing in 1979 and then had a permit of my own, commercial permit, um, starting in 1985 or 86 and um, I fished commercially as captain as 5 years before we sold the permit. And then I also tendered and worked as processors here in Bethel. I worked for Fish and Game as a tech for 3 or 4 summers in college. And then now I'm just a subsistence person with my kids.

MK: Alright. Hi Mathew Kapsner. My Yupi'k name is (Mataq) after my gram's brother. Um I've lived in Bethel for the past 10 years. Yeah.. So past ten years previously were in Juneau but we've always stayed in Bethel right? And then what connection I have with the river. I kinda been fishing for a while now with my mom. We've.. I haven't commercialized fish because that's illegal. And uh {chuckles} in Bethel but I subsistence fish during the summer when I'm not working {chuckles}.

JE: Okay awesome. Thanks for sharing. This pencil ran out of lead. Can I use that pen? Thanks sorry about that. Alright and the next question is, oh thank you, how did you learn to manage steward or care for salmon when you were growing up?

MP: Okay so, the way I learned was really by being at my extended families fish camps. Um one summer we've lived at my mom's younger brother's Alexies fish camp with him and his family and just growing here there's always instructions about not wasting any parts and I grew up you know eating heads and bellies and collars and you know um eating the flesh around the fins. I still eat the skin whether it's baked or dried. Um so that's you know it was, um, in then a lot of belief that fish give themself to us and growing up as a child hearing the blackfish song about seeing a blackfish going into a blackfish trap and it had holes in it. So it raised its head above the water and saw in this camp or community um it was untidy and peoples, you know, this lady threw out dog scraps, and the dogs were all fighting over those scraps instead of the dogs being tied up in the right way and portioning out to each dog so they're not fighting over the food. And she um in this story she threw waste into the river and you're supposed to dig a pit and put the waste into the pit um. So the blackfish said to them, well I don't want to give myself to those people they don't take care of their fish right so he swam further and saw another blackfish trap and looked and the people were doing things with you know in the right protocol and they were neat and orderly. And weren't fighting amongst each other. So that fish gave itself to those people. So I, and, even though you know we didn't talk about it everyday, there were still things like um, just little admonitions. You know, if you didn't, weren't clean or tidy the fish might notice. ANd then I'd hear in meetings um elders saying that we aren't supposed to sell anything that God gives us from the land or river or the ocean. Were not supposed to sell it for money. Um, not that I internalize that then, or, you know, listen to that, because obviously we were a commercial fishing family. Um, but just you know hearing elders saying that as a rule and then also say if people fight over the fish and fight about the fish. They'll quit coming. So those were some of the things I grew up hearing.

JE: Thanks for sharing.

MK: It's like happening.

MP: Mhm

JE and MP: {laughs}

MK: Oh is it my turn? Okay. Um well I just kinda grew up fishing with my mom. I don't have that much on that. Like uh {chuckles}

MP: Well did you grow up fishing to sell your fish?

MK: Just subsistence because it's illegal to commercial fish.

MP: Well it's not illegal.

MK: Well it's illegal in Bethel though.

MP: Well you have to have a commercial permit and they don't have that many buyers. Thats..

MK: Wait, in Bethel you can still commercial fish?

MP: Yeah, like silvers and there's commercial processors or people with commercial permits who sell them to AC stores. That's how they were selling them in AC.

JE: Oh yeah..

MP: Yeah if you have a commercial permit you can go to Fish and Game and get a catcher seller license since there's no processor here.

MK: I did not know that..

MP: Let the record reflect the look of shock on Mathews face.

MP, JE, MK: {laughing}

MK: Ahhhh, okay…

JE: Your mom, like did she, or did you learn how to take care of salmon or.. What did you learn?

MK: Well I don't really cut them. I kinda just catch them.

MP: Well what do you, I mean do you waste any?

MK: Well I give them to you {laughs}

MP: Well in like when you were first fishing who did you give your catches to?
MK: Alex Kapner.

MP: Like Katherine (Seeders) because she was a widow?

MK: Yeah

JE: Okay.

MP: Yeah that first year I was really proud because when Van and I were out fishing, he was like, how old you were, 10 or 11 or 12. And I said who are you going to give your catch to? And he said right away said Catherine. Because her husband had died that April. So he wanted to fish for Catherine.

JE: So that yeah..

MK: I kinda like answered a lot of questions like these (AYA) so i'm like outed. {laughs}

JE: That’s okay.

MK: So it won't be as proficient. So they are misinformed about commercial fishing thing. I was like “its illegal”

JE, MP, MK: {laugh}

MP: Yeah well King's it is illegal, but chums and reds and silvers…

MK: Well yeah that's what I was talking about.

MP: Yeah. You’re right for Kings it is illegal.

JE: That's good. And anything to share is valuable.

MK: Yeah lets just hope that it doesn't make it big.

JE, MP, MK: {chuckles}

MK: She’s like “It wont”

JE: Okay what were the rules about harvesting and sharing and then how were the rules enforced.

MP: So the rules that we had about harvesting were never harvesting more than you can handle. Like it's really bad to let your set net get a ton of fish in it and let them get old in the net. Or you know get 80 in your set net and then not have the ability to put them away right. Um we also had strong rules about like the first catch of the year being distributed to old people and people who can't fish for themselves. You share, you know, like the first one you catch you don't put it away for your family. You cut it up and share it with as many people as you can. And people that can't harvest for themselves. Um, so that was one of the rules. Those were some of the rules and it seems like they were enforced by social

pressure. Um. Being scolded or corrected and not really in public but it was bad enough if it was in private.

JE: Was that from anyone or elders?

MP: Usually its people in your family, or elders, or just anybody actually. You know even if it's chum salmon or like um. If you catch some and put them away and let them go sour or let them get maggoty. Even if it's chums that's still really shunned. And you are told, kinda the punishment is bad luck. Bad luck in the future and it's interesting to me how many times I have bad luck that I thought back and wondered what I did wrong.

JE: Bad luck when you were out fishing?

MP: Well there was a few years or two summers were poaching from set net and we didn't know the second summer till the end until one of the people who has a fish camp across from where we set our net said whenever we drive away people would come from upriver and downriver to check our net. And then, my son here started camping by the net. He camped by the net for two or three summers and having 12, 13, 14 year old boys come up over the bluff when they heard a boat coming made the boat leave. But the two summers that we had poachers where we were not getting, you know, they'd leave the whitefish or the chums for us. But they even cut the net to get the fish out. I didn't really realize until after the fact. But during those summers, I was plagued with guilt about you know what.. What have I done wrong that is making it so that fish were not able to catch.

JE: I see. Anything you want to share Van? {chuckles}

MK: {chuckles} What was the question again?

JE: Um, what were rules, the rules for harvesting, did you have any while out fishing?

MK: The exact same she had.

JE: Okay.

MP: Well, and like one for kids, I mean, a friend of mine came from New Jersey and she's from here. So she knows, she understands our norms. But her children who had been entirely raised in New Jersey were on the river for the first time as kids. And when we were, when the fish came in the boat. They'd go "Ohh gross! So slimy!" Remember this?
MK: Yeah

MP: And I had to pull my friend aside and say "I'm really sorry but you know I since this is our camp, our net and everything. If you're not going to mention to them, is it okay if I tell them or mention it to them is it okay if I tell them that were not supposed to talk about the fish in a way that is disrespectful or would offend the fish" We don't, like if its birds, we don't talk about them smelling or having bugs on them. And with salmon, you never say "ew gross" um, that's a really disrespectful thing to do. And even if it's children you

need to tell them right away that's that not the way you behave towards or think about your catch.

JE: Okay

MK: I don't think that I've done that before. Also because I was around them a lot more. Like ahh there's fish. They must be slimy.

MP, JE: {Laughing}

MK: Like if the fish was dry I'd be like “Yo what? Like what's wrong with this fish?”

MP: Yeah

JE: Okay, thanks. Think that's it for that question. Unless you guys have anything else left to share?

MP: Uh-uh

JE: Okay. Are these teachings, rules still being taught and followed today? And why or why not?

MP: I think they are still being taught. And maybe in my 20s and 30s I didn't really think about them that much. It seems like since i've been in my 40s um I don't really know why it's like I'm much more aware of them and much more accepting of you know maybe in my 20s when I worked for Fish and Game and because my western side I think I may have kind of dismissed it as superstition. Or um old beliefs. But I really you know, I think on a deeper level, you know, there were the years or decades of dismissing it. And now I've come back and I see the wisdom in all of the, i mean, even if maybe on a conscious level I don't fully believe that those salmon are checking to see if Im worthy or have some kind of radar to know if my net is a you know a good person's net to go in to. On some level I do see the wisdom of not arguing about things, not having a filthy camp. Um, you know just one of the rules, um, is that you have to keep fishing for them, for them to come back. And you know I don't, now I think I'm at a place where I realize western science doesn't know everything. Maybe there are lessons within this traditional knowledge that we don't have an answer for but is still correct. Maybe we don't know why it's correct. But it is still correct. But I think I have more faith in traditional knowledge now.

JE: Okay.

MK: I guess, okay, um, yeah I definitely think its still, uh, it today because like I'd say there's a bunch of kids in the room and theres new kids like you can tell which ones are from around here and which ones aren't based on the fact how they respond to like seeing certain animals and how they respond fish, fish camps, and like how they talk and everything. And you like you never are supposed to ask for things and things are supposed to offer things to people and everything like that. And the people that ask for

things is like “yoo chill” {laughs}. And so you can kinda just tell if they're from here or not based off traditionals.

JE: And those traditions and stuff are they still being followed and enforced locally?

MK: Yeah. I wouldn't say necessarily enforced but taught.

JE: Okay

MP: Well one of the funny things I was fishing with my husband and his dad and they started arguing and I didnt, it wasn't like I put a lot of thought into it, kind of out of a reaction. I scolded them. I just kind of quietly said “Shhsk, the fish are going to hear you” and both of them immediately responded positively. Like they got into shape. Because on some level we all believe these things. So I kinda do think we still check each other.

MK: That's weird because like I don't know if it's like believing, but just being respectful to the culture. You know what I mean?

MP: Mhm.

MK: Because I know the fish don't hear me but it's like I'd still be quiet.

MP: Well and you know during the years of extreme conservation, my father in law and I were fishing and I pulled in a big king and I asked him right away because it was with a four inch net and it wasn't entirely gill and the gills were not damaged. And I asked him “should I release it?” and he immediately said “No!” You know as if to say you can't put it back if it already gave itself to you. So you know I do think, um, on some level there's broad based belief.

JE: How do you think about the relationship between salmon and people in your culture and you kinda gotten to this somewhat. So feel free to share whatever, it's cool.

MP: Well.. One of the things I think about is the Yupik goddess of the sea. And how she withheld resources because humans were being, um, messy and trashy and not respecting the land and water. And this is the downturn in Chinook productivity and the downturn in returns in the last ten years. To me it's simple, where, you know, as humans collectively we have done damage, we've been doing damage and we continue to do damage to the Earth. Plastics, um, and all kinds of other things, um, are damaging the fish. So, what was the question again?

JE: Um, what do you think about the relationship between salmon and people in your culture? So the Yup’ik people.

MP: Yeah, I mean, I don't, yeah I think salmon have a relationship with people and people have a relationship with salmon. And us harvesting them, I mean, we are part of the ecosystem so there wasn't a symbiotic relationship where we were part of that ecosystem.

And I do feel like human beings have made a balance, disturbed the balance, and maybe not Yup’iks, but human beings have definitely been polluting.

JE: Okay. How about you Van?

MK: Yeah no for sure. Um, humans play a big part in the reason why fish are disappearing and everything. With pollution, carbon emissions, everything like that. But I wouldn't say it's not necessarily, like I definitely know Yup’iks can litter and stuff you know. Um, huh?

MP: Yup’iks that what?

MK: Litter.

MP: Yeah..

MK: And like we drive cars, so like...

MP: Yeah.

MK: Yeah, it's kinda everybody has a part in this. And I feel like, um, wait what was…

JE: For you, what is the connection between salmon and your culture? So like Yup’ik people versus you travel all over, um, everyone has a different connection with salmon or may they not have many relationships with salmon.

MK: Or like a different connection with the environment.

JE: Yeah how do you feel about..

MK: In the biggest cities, I wouldn't say most people necessarily do but that's not always true. You can definitely find Yup’iks that still fish in the city. Uhh, and then, but, everybody is kinda, everybody has a culture with it whether they know it or not. Because you can have an urban based culture. Or you know, like, African culture. Like there's just so many but, um, and then what's my connection with the fish or the culture's connection with the fish?

JE: Either.

MK: Okay so. My connection with fishing is subsistence fishing and doing all the stuff. But culturally fishing has been a really big part in history. Mainly because we live on a river. Like what else we eat aside from fish. What like, wait did they even hunt those back then?

MP: Mhm.

MK: Dang with like a harpoon? Bows?

MP: I think the nets were made out of moose and caribou (sinu). Like their muscles.

MK: That's interesting. Kinda crazy. Yeah, um. Yeah I feel like we're definitely like a fishing culture

MP: Yeah I feel like we're really connected to salmon because it makes up such a big part of our diet

MK: Yeah, um,

MP: And I feel like you know the nutritional content is really high so it makes us healthy people with salmon.

MK: Yeah I feel like everyone in Alaska fishes for the most part unless they are like vegans. {laughs}

MP: But like, you know, even along this river there different communities that rely more heavily on salmon. Like Bethel relies more heavily on salmon. Like Bethel the Yup'ik name is the place of many fish caches. It's the name of our town, (Mamterrilirmiut). Place of many fish caches. And, so it's even in our name of our town and it was such a big part of our subsistence larder. Like, you know, I don't know 50% or something. We and Kwethluk between Bethel and Kwethluk. Or you know this part of our river, this middle part, because we don't have land mammals, and we don't have as many moose and caribou and bear as upriver.

MK: Yeah a lot farther.

MP: Yeah so we have, I mean, the biggest resource that we have is the fish resource so we consume a lot of fish. We have a very strong relationship with fish.

JE: Okay. And you said that was place of many fish catches or?

MP: Caches.

JE: Caches. Like a, like, you know like a…

MP: In the ground.

JE: Okay yeah yeah. {chuckles}

MP: Place of many fish fridges, you know?

MP, MK: {laughs}

JE: Yeah like geocache

MP: Yeah

JE: Got it.

MK: And people are with a bunch of fridges in the ground.

MP, JE: {laughs}

MK: Like fridges are really bad for the environment.

MP: No, but..

JE: You guys have a fridge though

MP: Like at fish camp.

MK: Everybody has fridges. Ohhhh thats what you..

MP: At fish camp. That's what I mean.

JE: Yeah

MK: Ohhhhh okay

MP: Like hole in the ground.

MK: Ohh okay that's what you guys are saying about.

MP: Yeah {laughs}

MK: Nah I'm saying about like fridge at the house.

JE: No that fridge at the sonar is really good

MK: Yeah natives sell a bunch of fridges too that's polluting. At some point.

MP: But the ones we dug in the ground in the olden days.Those were good.

MK: Yeah no those are cool.

JE: Yeah.

MK: It's weird because they actually work. I remember I was so amazed I didn't understand. I was just like it's just dirt. How does it work? {Laughs}

JE: That's incredible. Yeah

MK: Did you learn about that this year?

JE: Your mom just gave me a soda from the ground and it was so good. It was really refreshing and.. Yeah.

MK: Homemade. Im kidding {laughs}

JE: No it was root beer soda {laughs}

MK, MP: {laugh}

MK: We just reuse the bottles.

JE: Yeah. Okay, um, next question is so now we're going to get some into the concern about fisheries and more into the fishers management side of things. Do you feel that your knowledge and values are reflected in fisheries management?

MK: Is that like Fish and Game and Fish and Wildlife?

JE: Mhm

MP: I have not felt like Yup’ik values have been part of Fish and Game or Fish and Wildlife.

JE: Okay.

MK: Yeah I’m not 100% sure. Um, I feel like there are a lot of, uhh, like Yup’ik people in F&G and stuff they may not, I don't know. I feel like F&G are like for the most part F&W not so much. Um, I don't know its really just depends where they're from. Like if they are from here they're pretty cool but if they're not they're just okay. I honestly...

JE: You go to the meetings though, yeah?

MK: Yeah. I’m not. Yeah

JE: So like when they make decisions do you and like however the river might be managed, do you feel like they are incorporating Yup’ik values from decisions you've seen made?

MK: Um, not exactly but I feel like it's like the decisions should be made by like the tribe or something not exactly the management like F&G F&W

JE: They should be made by the tribe?

MK: Yeah it should be made by the people who were here first. And like understand the lands better.

MP: I don't think they have reflected, you know, in the last 20 years of all and often the people that manage from F&G and F&W. F&G there from other parts of the state. A lot of people were born and raised in Anchorage and you know when they, in 2012, when they had the fishing strikes. The manager at that time didn't know that Yup'iks had a different worldview until he sat through those court hearings that winter. And you know, in 2013, he said "yeah! I went to the court hearings and I was shocked. Yup'iks have a completely different worldview than we do" And I was surprised that he was surprised. Um in 2017, when the inner tribal fish commission started working with the refuge on Chinook management. They used a process called, um…

JE: Like the structured decision making?

MP: Structured decision making. So in the end of June, they asked the local elders to predict how many boats would be fishing in the next two openings. How many fish they thought each boat would catch. And so the in season managers predicted the number of boats and they predicted that each boat would harvest about 100 fish per opening. And then after those two periods happened on July 1st and 4th or something like that, 1st and 3rd, or 1st and 5th. We came back on the 6th and the managers said to me privately they said "we were shocked by how accurate they were", you know, these guys predicted there would be 100 fish per boat caught. And we thought why would anybody want 100 fish? And this was during a summer where there was two chances to fish in June which is when most people are done. Um they do all their catching and complete it by July 4th. And that summer there had been only 24 hours of fishing allowed. So everyone felt the need to catch up. And there was this desire, everyone had this anxiety about not getting the fish that they needed. So when they looked at, when we looked at the numbers, the actual harvest was about 96 fish per boat. And F&W could not believe that our prediction was as close to accurate. And they couldn't believe that anybody would want to harvest that many. And again, I'm surprised at their shock. Um, that these are the people managing us and our harvest and they are that out of touch with what we need.

MK: Our culture.

MP: Yeah.

JE: Thanks. What worries or concerns do you have about your salmon fisheries today in the Kuskokwim salmon fisheries?

MP: My concern is that, so the system that natives have to operate in is a cash driven system. So F&G one of their missions is you know a certain million dollar ex-vessel value statewide. So that means that the subsistence fishery has no value. Because they don't put a cash value either subsistence harvest or personal use harvest. And that's also negative to people on the road system catch 6 fish per household for their personal use. Those have no value to F&G either. So when they make decisions on commercial fishing like Cook Inlet or high seas fishing in the Bering Sea, the commercial harvest is always the most important. Or hatchery pinks, we've come to understand that pink salmon and

king salmon eat the same food in the ocean and F&G every year increases the amount of pinks for hatcheries and that's only a commercial harvest. Most Alaskans don't eat pinks. That's all for export. That's all ex-vessel value. And those pinks are eating the same food that should be going to subsistence king salmon. So that's my concern is that we are not a cash driven sector and we never will be and we don't want to be. And that right there makes us have no value.

MK: My concern is that in the future F&G or F&W are still the ones advising us and everything. I hope that one day it's the tribe, um, and stuff like that. I don't know, I feel like, um, natives in this country have been treated very poorly and not considered um, yeah. Like it's crazy like natives are pretty religious people. Like the most religious people I've met but like it's like Gods been the worst to them.

MP: It might balance out in the future. One of the concerns that I have about management is you know that western management is there's no value in spirituality. There's, like, in western science there can't be a spiritual component. And for us that's the biggest part of subsistence is that spiritual side of it.

JE: So just in the, like, huge difference in worldviews, values?

MP: Yeah I don't see why western science can't have a little bit of an open mind for spirituality. Um, you know, and I worry that will be so emotional about it that it will take an emotional toll that we won't be able to focus on solving the problem.

JE: And what will, what will, be so like emotional taxing?

MP: Everything. I mean you know when men come to the meeting, it's not, to me, it's not just the emotions we have about fishing, it's kinda the last straw. Because there's so, you know, and the other thing is that natives are not good at communicating with western scientists and western scientists aren't good at communicating with native people. So were not having real conversations from either side.

JE: Okay.

MP: And I feel like it's gotten to the point where neither feels that it's constructive to even try.

JE: And.. what so you think would be useful to this somewhat, what are concerns you have with fisheries management in the past. So this is what we can get into the strengths and weaknesses of the current salmon management system. Is there anything that you think that works well? Doesn't work well? How can we improve?

MP: I think what has worked well is the last 5 years on the Kuskokwim, 15, 16, 17, 18, and 19. The inner tribal fish commission has advised F&W on Chinook management. And what I've seen is that places where western science has gaps in their data and their information or things like run timing and run strength and from what I've seen traditional

and ecological knowledge can help fill some of those gaps. And not in the same way that western science does, it's not through a daily recording or you know, peer reviewed western science techniques. Like developing a hypothesis and this and that. But it is science because it is, um, observing and noticing patterns. And I think a lot of what science is, um, continuous observation and recognizing the patterns. So when it comes to run timing, there are places in the river where people who have lived in that place on the river they do have indexes so to speak on run timing. And you know one example is 2018 James Nicori said, you know, we the fishing life didn't want to have more openings for Chinook. And James Nicori said "I think that we are going to make escapement and I don't think that we're halfway through the run yet because at my fish camp they are catching on the left side of the river and not on the right side. And when all the fish run on the left side those are the ones that are going up to the headwaters and the ones that are on the right side are going up to lower river tributaries. And they come in later about halfway through the whole run." So we actually did have two more chances to fish and we still met escapement. Based on James traditional knowledge.

JE: Really?

MP: And this year, people in Tuntutuliak said we have so many mosquitos and this is an indicator run strength. We believe there will be many many more fish to follow. And sure enough now F&G they changed their forecast from 150 now their run reconstruction they are estimating 220. So to me, this proves that traditional ecological knowledge has something to offer. We actually have the last two seasons we've been incorporating it. And it hasn't been perfect, it hasn't been a perfect mash, it hasn't been, you know, they isn't like a prescription of how to integrate TK. It's been done. But in a more informal way.

JE: Okay. And speaking of the formality, is there, you know how that when they, you guys decide there's going to be an opener and then they put out a special action, or news release.

MP: a news release.

JE: When they, you know when they had to justify, is there an area where they include this information so that it's documented on paper and this is why because of the TEK we just find an opener.

MP: I don't know, I'd have to go back and look at those. I don't think so though. And Ray Born, the acting manager has said I need to have western science just to find why we're having openings or closures. I think they are also supposed to include traditional knowledge but he doesn't do that.

JE: Really? Okay

MP: That's what Kevin (Wetworth) was just telling me

JE: So that might be an area of improvement?

MP: Well and the funny thing is in 2018 when James Nicori had this traditional ecological knowledge information that he shared, and the refuge still didn't want to have an opening. We made it clear because even when they were closing it, and that was not what the fish commission recommended. Their message would say in consultation with the fish commission, we are closing it. Which made it sound like the fish commission recommended there be a closure. So when he was going against the advice of the in season managers, we said you know the fish commission said when you issue your press release, your news release, we want it made clear that you are F&W is taking this course after consultation with the fish commission and going against their advice, this is the course of action were taking. They refuse to do that and they went with their advice instead.

JE: Wow

MP: So that was good. He also, the refuge manager at the time said okay we're going to go with this but are you willing to take the blame, is the fish commission willing to take the blame if the escapement is not met? Immediately the fish commission responded yes we're more likely to take the blame because we're getting the blame now anyway. So..

JE: Yeah so just open it.

MP: But I thought that was a strange question. Blame or no blame, it's a resource that ultimately we have the bigger investment in, the bigger vested interest. So yeah. You know.

JE: Yeah.

MP: You know. If the run tanked via blemish on their career, maybe, but if the run tanked for us it would be devastating to us as a people and to our culture.

JE: Anything you want to share Van?

MK: No not really. I was kinda, that was good. I feel like I'm not educated on these topics and I don't want to like…

JE: Well it sounds like what you mentioned some concerns or that the tribes aren't managing and that's kinda what you hope to see in the future.

MK: Yeah for sure.

JE: That could be where we can improve.

MK: Yeah that be really cool. But I doubt it's ever going to happen any time soon.

JE: Well they are already in it.

MP: Mhm. Getting our foot through the door.

JE, MK: Yeah

JE: This is the last question here. What do you want to see for salmon and people in your region in 40 years?

MK: Oh I'll go first {laughs} Uhh, like raise my hand. Um, I'd like my kids to be able to fish but that's kinda like a, I feel like that's a really, at this point, and the rate the world is going, that's kinda a fantasy almost. Which is really sad to say. Um, just because of how much we are polluting into the world and like how little we care, basically like how little action has been taken to um like clean it up. And I feel like that's gonna be stuck on our generation or future generations to clean. Uhh, so, maybe in 40 years we'll be able to fish, i don't really know.

JE: But that's what you want.

MK: I'd love it. Yeah that would be sick. But I don't know well see what happens. Um, maybe they'll will. Uh, lets hope. Just like I'll wait till all you guys get out of office. No, I'm kidding. {all laugh}

JE: You are already making rules you just went to the

MK: Yeah and then they're having all those climate strikes and stuff. Which is like really cool. I really like that idea. I just wish more kids did it. I saw like maybe 20 kids in Fairbanks that did it. Well Fairbanks is a pretty conservative place. But uhh, yeah. I don't know.

JE:And then what, you might just want to clarify

MK: Oh yeah Alaska Youth and Environmental Action. It was like uh 30 kids from around Alaska went over there and they all had different backstories, but same push for a better environment. But um, yeah that's so weird I would expect more people to go to the climate strike just because Alaska is such a green place. Like we see the forest everywhere. Like you can't be in Alaska and not be able to take a 20 minute drive and go to the middle of nowhere.

JE: Mhm.

MK: Which is weird. Because people are like, I don't know. It's weird. Doesn't make no sense.

JE: That's good you are taking action.

MK: Yeah.

MP: So for me in 40 years I hope that we have all the traditional knowledge that elders possess now and are able to get it from them and share it going forward. Because I didn't know about a lot of the traditional knowledge in terms of other species that, um, are indicators of salmon presence and abundance. Um, and you know, I think we probably have 15 or 20% of what you know the body of traditional knowledge was before contact. Maybe 5 or 10%. I don't know what the percentage is, but it is definitely not what it should be. And my hope, you know, one of the things I've noticed is that the way that Yup'iks think about fish has been mocked by F&G. When I was a tech I'd hear biologist rollicking with laughter about things that elders said. Like one of the things that I heard an elder said at a meeting when I went to at college was that they were talking about swim speed. And this elder said that he was told that fish can rise above the water and fly 100 miles in one night. And you while I might not personally think that's what happens, I don't think that it's worthwhile to laugh at somebody for sharing that perspective or information he was taught. Um, and you know that, the F&G people didn't, they were not buckled in laughter and tears in front of that person but when they left he was. And it sure made me ashamed of our body of knowledge. {tearing up} Um, and like ive seen Robert Lekander be hesitant about sharing what he knows. What I'm hoping that somehow {long pause} that something changes so that we are proud of what we know and what we've been taught and what our ancestors knew and the observations they made because, well not all of them may have been correct. There's a lot more to them than we give them credit for or western science gives them credit for. I guess my hope is that there won't be shame and embarrassment for the things that we've been taught and its not hidden. I feel like traditional ecological knowledge and our spiritual side has been shunned to the point where it's driven underground and we only have these conversations in private. And elders don't even want to share it because they feel like somehow it will be detrimental. They don't want their kids being ridiculed or their grandkids being ridiculed. And in 40 years I hope that we have an abundance of fish because of tribal co-management. I hope that we can get a handle on the greed in terms of ocean resource management. I hope that unfeathered capitalism we can somehow get the reins on the way the ocean is you know, um, being overharvested. I hope we can get a handle on ocean acidification and global warming.

MK: Yeah also on top I think thats why it should be put to the tribes not exactly from the lower 48 or federally governed people. And I also hope in 40 years, or wait we are still on that question right? {phone call} I'll wait for that… Is that your phone? Okay. Uh I also hope that in 40 years were not really a capitalistic society more of a regenerative. Um, meaning everything goes around in circles you know. Like we only take a certain amount of this, like what we need basically. Like just take what we need, don't take it all. And like don't devastate the land that you took it from you know?

JE: Yeah.

MK: Um, we do a lot of things like this at AYEA and they made, like we got put into three groups and they made each group try to do routines, like uh, so you had to basically grab a marker. There's like somebody sitting in a chair, grab a marker, mark the paper, and go give the marker to the person in the chair right. And then do that and then all of us ran

out of markers instantly right. Like after a couple goes, you had to do that work with a couple push ups on the way there. And then so they told us to break it up and uh like try to see if you can't leave as much as possible. And then one of the teams would go over there and grab a marker and put a dot on their hand and go around and go back and then like i don't know it was the way they did it made it so like they basically didn't touch the environment. So what they do is there is no boss person so it was a co-op and then two people would go do the, depending let's say you have one person that not really physically active right it's going to be harder for them to do push-ups, so you get the person that's more physically active to do more of the push ups so you had to do three so that person would do two and the other person did one and then they'd go down and do a little more and put the marker down and go back. And then it was just like a very fair and good thing.

JE: So like low impact?

MK: Yeah it was like there was like they didnt even mark the paper once.

JE: Wow

MK: They just marked themselves. And like that was the resources they were bringing back.

JE: Hmm, thats cool.

MK: Yeah.

JE: Well hopefully you can share that with your other classmates.

MK: Yeah. Oh yeah we're going to start a AYEA chapter here.

JE: Good

MK: Yeah I was just talking to a lady from Unalaska.

JE: Thats awesome.

MK: Yeah

JE: You gonna be the advisor? {laughs}

MK: We might ask Ms. Hansen maybe. We'll figure it out.

MP: Good

JE: Thats cool. Well thanks so much for sharing. Lots that I wanted to say and share but I’m supposed to just listen {all laugh}. Well this was really emotional for me

MP: Well my goal was not to cry and I didnt make it {laughs}

JE: Well that's really messed up. I was angry and sad at some of the things that were shared and made me think about how my people were treated too and both sides, mexicans and indians.

MK: Yeah I know. It really like all the real Americans were treated horribly.

JE: Yeah but I really am grateful for guys time and these responses and I hope that yeah we can all contribute to positive change and um, not feel, and i feel like your guys culture is really beautiful but i can't help but compare it to my own and it just feels like a lot stronger here. We lost our language and we lost a lot of our resources that were contaminated by petrochemical plants and spills and it's just amazing to be here and see all the fish. Even though maybe so little compared to what you had or your grandparents had. Yeah its just really beautiful and the people so welcoming and language is strong. So yeah Im grateful to be here and thanks

MP: Thanks Janessa.

INTERVIEW PARTICIPANT: ELIAS VENES, ELIZABETH HOFFMAN, CONNIE SANKWICH, DARRELL AND RUTH GARRISON

Interview Date: 10/19/19
Interviewer: Janessa Esquible, Destiny Ropati
Location: Bethel
Home community: Bethel

Janessa Esquible [JE]: (Goes over project details and forms) Let's go around the room and say your name, your home community, cultural affiliation, and how old you are. And then your relationship to each other.

Elias Venes [EV]: Okay I'm Elias Venes. I was born in 1928. Uh, I lived here in Bethel since 1965. I was born in Akiak and my younger years were spent up there till I was almost 20 years old. Then I moved down here since then I've been all over the country. Working many many different jobs. What else do I have to say?

JE: That's good for now. Just introductions. If we can get that information.

Darrell Garrison [DG]: Uh, Darrell Garrison, uh, born and raised in Bethel. Uh, 40 years old. Uh, ONC…

EV: (It's always that)…

DG: Calista member, shareholder, and Bethel Native Corporation. And Elias Venes is my grandfather and Connie, Elizabeth, Ingie, Ruth are my mom and aunts. Elias is my grandpa.

Connie Sankwich [CS]: Uh, Connie Sankwich. I'm 55 years old and from Bethel. Born and raised here. Um, also an ONC tribal member. Calista. BNC. Um, and the daughter of Elias.

EV: Your turn Ingie.

Ruth Garrison [RG]: Ruth Garrison, uh, Ingie. I'm 62 years old. Lived in Bethel most of my life. And daughter of dad and the girls. Darrell's my son.

Elizabeth Hoffman [EH]: Elizabeth Hoffman. I'm 67 in a couple weeks. Um, Born and raised in Bethel - well born in Anchorage - raised in this area. BNC, Calista. I'm not (claiming) Calista right now but {laughs}. ONC member. And sisters and dad. And...

Destiny Ropati [DR]: Uh. This is Destiny Ropati. I was born in Bethel raised here in Anchorage. I mean raised in Anchorage. I am one of the interviewees? Interviewer? Friend of Janessa. And (pretty much (unknown)). {laughs}.

JE: Uh, I'm Janessa Esquible and I'm from Detroit Michigan. I'm a member of Walpole Island First Nation Band. In Southern Ontario. And I moved to Bethel, or I moved to Alaska

in 2014 and I came to Bethel in 2016. And I work for ONC as their biologist. And we're partnering with University of Alaska Fairbanks to do this project. Indigenizing Salmon Management Project. And Destiny's been interning uh, fisheries intern for two years. So she's helping out with this project also. (Goes over project forms and purpose).

DR: Um, so can you tell us about salmon fishing when you were young?

JE: Actually sorry we're just gonna back up some.

DR: Oh my bad. Okay. Nevermind. Take that back. This is our first question. So uh, can you tell a little bit of how you guys grew up and what connections do you guys have to salmon?

EV: You mind if I get closer to you so I can hear better.

(Switching seat options)

DG: I guess I'll start. Uh.

EV: I bet this will help a little.

DG: Uh, like you heard earlier. Uh, I started fishing when I was 4 years old with my grandpa and my dad. Uh, subsistence fishing and commercial fishing. And then when I was 16 my grandpa turned over the commercial fishing license over to me. Or my parents and it later came to me after I finished college. And I've been fishing ever since.

EV: A long time. (inaudible)

DG: And then uh, since then, I've been helping our sister... commercial fishing kinda stopped. No more buyers here in Bethel. I've just been fishing for everybody else. All in the family for subsistence fishing. Uh, my wife (inaudible).

EV: For the record, his dad is the best fish picker in the country next to me. {chuckles}.

DR: Do you want to state how you grew up and what connections you have to salmon?

EV: Pardon?

DR: (Repeats question).

EV: Oh! I grew up in Akiak. We didn't have an outboard motor. Almost all the years that I've lived in Akiak. We did our fishing with a rowboat. (Inaudible). (We sail up a boat). Mile and a half, two miles above Akiak. Drift down and try to take so.. Wouldn't have to go the (unknown) and that other king salmon were almost always caught. And uh, set net. We caught a few salmon fishing but most of the time was caught without a set net.

DG: And you used a fish wheel too.

EV: Well that's different long years later. Up in Aniak. I lived in Aniak for 10 years. After I left Nyac in 1956 I think. And I moved to Bethel in 1965.

JE: The Nyac mine?

EV: Just Aniak. Worked for the FAA.

(Cannot differentiate the voices of the sisters) Ruth, Connie, or Elizabeth: He did work at the Nyac mine though.

JE: Okay.

EV: Before that I worked at Nyac for 7 years I think.

DG: My grandpas tell me stories about that fish wheel when he grew there, there was one time it was placed in a bad spot and he didn't get very many and they told him to move it to a different area. And he moved it and the next day he went there and he had 300 and it..

EV: No I had 600

DG: Oh 600 {chuckles} And it was overflowing.

EV: I had the best, somehow wound up with one of the best fishing sites on the river. That was by accident I guess. I built my own fish wheel. When I got there the town people asked who's gonna build your fish wheel for you. I said I would probably (inaudible). I didn't see any difficulties building it. I do my own stuff all the time. And so I built one. 7 5 different times. And we only got 2 to 3 fish each time. And then the neighbor boys come along, two (unknown) came along said they take (unknown) how to set net in the right place. So we went out and we finally set ah, we went above Aniak and (unknown) Aniak, so (unknown) drifting and rowing. We wound up 4 and ½ miles from Aniak. And I thought these guys up here are just trying to get rid of me because I'm a downriver eskimo. And there, most of those up there are part Indian. So I, so get me at the right place. Every day I went, by the fish wheel early in the morning most of the time before people got up. And on my way home I have to give away a bunch of my fish. Different people that didn't have any fish wheels you know. No outboard motors to go that far. And I did that all the years and then yeah.

JE: Okay.

EV: 50 or 75 fish each place that I stopped. Because I couldn't handle. I had a full side job. And I was building our house and I was (unknown). Oh that's from Akiak long before, when I was just a young boy [talking about a picture being shown to interviewers]. Maybe 13.

JE: Wow.

EV: And the other guy, Butch (unknown last name) I think. Oh he’s not in this one.

JE: That's in Akiak?

EV: Yes.

JE: Okay.

EV: But these are, that's my dad's shop the white one. And the one of this side is Arthur (Rues?). That house you see in the background. With the white window, my dad built most of the house in Aniak - Akiak.

JE: Wow that's really nice. Do any of you ladies want to share um, where you grew up and your connections to salmon?

EH: Maybe the oldest one I grew up remember Aniak well. Moving up there and all the stories. Moved down here when I was early teen. Mostly living here ever since. We fished up there. I remember fishing up there with them and cutting fish on the (unknown). Which was logs and then a net underneath. That's the upriver style. So there always in freshwater you know. And then we get to recatch a lot of the (silver?) and put em’ up there.

EV: That's where I put my fish that I gave away in those, what we call (Sirviqs)

JE: What were they?

CS: (Sirviqs)

JE: Oh okay.

ES: It's made out of chicken wire between logs.

(two people talking (CS & EV), inaudible).

EV: Stay fresh, till you get em split you know.

JE: Huh can you spell that.

CS: S-I-R-R-

(trying to spell word, asking for help)

Lady speaker: S-I-R-V-I-Q

JE: Sirviq. Okay. And that's where you place the salmon in there.

CS: Yeah in the chicken wire under water because the logs were floating. And the wire- the logs were up here and the water and the nets under here. And they hold the fish fresh till we're ready to cut.

DG: The Leary's still use that up there.

EV: (unknown) most people have a smaller much more than what I gave them.

JE: Wow.

CS: My sister I remember her falling in to that {laughs}

EV: (unknown) built my own boat.

JE: Wow.

DG: He built the best fish boat there was. That fiber glass one.

EV: Uh-huh.

EH or CS: He built, that's one thing that went hand in hand with fishing is we uh, when I was really young, like 7, 8 we up until then we lived right over here on main street. Um, where that empty lot is that used to be a big house there but, he had a this (unknown) hut out here was over there. And he or one similar to it and he built boats in there. Wooden boats.

JE: Oh wow.

EH or CS: I don't know how many hundred. I don't know how many boats he built in this delta.

EV: Nowhere near uh, hundred but quite a few boats.

EH or CS: It was a lot. A lot of boats. He was always building a boat. And so we spent a lot of our time in there with him. Um, and those were mostly fishing boats.

EV: But then on the side I built speed boats just for the hell of it. To get a few dollars. {laughs in background} (unknown what's being said)

EH: There was 10 of us girls, so he had all girls, and so we were all um, we were always going out and fishing with the family and cutting it with family (affair, up here?) Especially when it came time to do smokehouse fish.

EV: We put up fishing every single way you could think of I guess.

JE: Wow.

EV: Dried and smoked em. Made salt fish out of (soles) lotta sold the salt fishing (unknown) or butter (unknown) that were emptied out you know. Wooden barrels.

JE: Mmm. When did you guys stop using those? Or do you still use them?

EV: I did, when's the last wooden boat I had?

CS: Probably 80s?

EH or RG: Yeah. Um.

EH: When we came back from Saint Marys…

CS: Early 80s maybe.

EV: I was building with plywood boats after Bernie and I were married. So we were married in 74.

CS?: Yeah.

DG: It was mid-80s

(multiple people talking, hard to differentiate voices between sisters)

EV: When I decided I was…

RG: Once Nina was born they were, they started making…

EV: This morning Connie was cleaning up in the.. Pictures and stuff and she found a better.. We met a couple. Oh maybe I better not.

CS: Yeah that's not fishing one.

EV: (unknown) interesting story. Fishing from Australia. We got to know them for 9 ½ years every port that they ever where in.

(Possible food being offered in background)

EV: Try to get those letters back but we found all of them today. The girls stashed away one of those things over there. It was

(talking in background)

EV: What was their name again?

EH: I’ve never met them. We were living in Saint Marys.

JE: Hmm.

EV: What was their names again?

EH: I don't know.

EV: Anyway they had a boat, they built from Australia and sailed up to Alaska and they went all the way around finally wound up in the Sea of Japan. They disappeared up there. (inaudible) What happened to them. I pray for them today still. At least I hope that they find someplace. Lots of outlaws up there. People losing their boats but they were determined to sail that part of the country. From Sydney to Kodiak down to (unknown) Black Friday Harbor. Next one was down in Portland. And then California and down Peru. On the way they had all kinds of stories. Every place they stopped they wrote us a letter.

JE: Thats’s cool

CS: But they didn’t fish for salmon on the Kuskokwim. (all laugh)

EV: But they were truly sailors. But now we're back to salmon.

RG: Going out fishing we used to fish all night. And then come home and cut fish at Uncle Joes fish camp. Fish (cache).

EV: Thats a big process, you know you gotta make it just right. You gotta treat it right, clean it right, hang it right, and smoke it right. Get the right kind of wood to make it taste always the same.

JE: Could you all tell us about salmon fishing when you were young and then how is it different than today?

EV: Well when I was fishing, when I was a fish, I'll have to… They weren't around then… None of these… Anyway we rowed all the time. We rowed up the river. Drifted back down. We generally used a 30 or 25 or 35 fathom net. And you didn't need a big net them days. Because fish were (unknown) and you drift down and you have a boat full of fish. You might spend a couple times drifting. And you have as many fish you can take care of. Most of those fish were caught for dogs which we used for making a living with hauling our wood. Hauling different people around. Teams being paid for (unknown) doctors, dentists, and all kinds of people. Long time to live.

CS: The biggest thing I remember about when we were young fishing was the size of the fish, were probably double. At least double. They were huge. We had, it wasn't unusual to have a 60 pound king.

EH: As these pictures will show you.

CS: Yeah and some of… There was plenty of fish to go around. Um, you didn't see much waste at all. People used almost every part of the fish.

EV: Even the backbones for..

CS: And we even used it, we even use the bones and the blood for our garden. Every year. So that as fertilizer. And so yeah. We still do that. The other thing is it wasn't as regulated as it is now. The subsistence fishing was not as regulated but it wasn't… It doesn't matter.. People fished for necessity or you know. Um. We used an 8 inch mesh for kings and..

EV: I made… Sold every, every square of net several times 50 fathoms long… (unknown).

JE: You made the nets?

EH: Yeah he made the nets.. There's a picture and this was a regular occurrence we were always repairing the net.

EV: Every piece of work on that was done by..

EH: We always did it with mandolin (unknown) (laughs)

JE: She's a ukulele player

EV: (unknown) hang the net after you know. Put (unknown)

JE: Oh really? Wow. So how long did it take you to make the net?

EV: Well most of the winter.

JE: Wow.

EV: Depends how much time you had. I had to do all kind of things with dogs, you know. Make a living with the dogs and go out with them and trap with them, hunt with them, cut wood. Anywhere from a mile to (unknown). We didn't have chainsaws or anything. We did it all by hand. I cut more wood than almost anybody in this town. River. I cut 700 cords of wood. That makes a hell of a pile of wood I'll tell ya. My brother and I together we cut over 14 hundred cords of wood.

JE: Wow.

DG: One thing that I noticed difference is uh, when I first started going commercial fishing, I was able to take naps between every set and drift and I wouldn't do very much and I get ah, nice check at the end of the day, at the end of the 12 hour opening and it started getting less and less and next thing I know I'm out there doing a ton of work making half as much as I did back when I used to just sleep on the boat. And so I really noticed. Thats what I noticed that the numbers were really dropping off and they weren't what they were and the prices fell out.

RG: When was that?

DG: This was in the late 80s, late 80s when it was doing really well and uh, mid to late 80s when it was doing well and then in the 90s it really started dropping off.

EV: I sold my fish generally to one guy, buyer, and he said that I sold him the biggest king salmon caught and (unknown) hundred dollars for (unknown).

JE: Wow. Hundred bucks for one fish. Wow.

EH: It was a dollar a quarter a pound so it had to be a pretty damn good fish.

CS: The last time we did really well was, Eli must of been a sophomore in ah, college, and he came, maybe a little bit earlier.

DG: 90…

CS: 88?

DG: 96.. Was his freshman year in college.

CS: He might've been a senior in just out of high school in 1985. Um, we were doing really well that (unknown) I said have you been drinking? And he said whatever we catch today Eli, it goes towards your college fund. And then like really? Cause I knew it was gonna be a good day. Man it was a great day. (unknown) He was dancing 'i made the money' But then after that it seemed to drop off really quick.

DG: And then when I bring my boys out commercial fishing I wouldn't even take a cut for myself I would just want them to enjoy going out. So after I would just pay for the gas and the oil and let the boys split the money. Um, I would take any cut.

EV: It's a real coordinated fishing thing. All my life. My family was pretty much, we had to use.. I had one brother and one sister that fished with us. Maybe up in Akiak. Before we moved here. Back when it was all rowing and no motors. Just a little sail.

CS: That's not one of these boats, is it?

EV: It's quite a little… Quite a little (unknown). Akiak downriver in a row boat. Go back home and groceries for the winter. Takes quite a while. A lot of hard work.

JE: You'd row to Bethel and then take it back to uh…

EV: Yes we'd sail when we could you know.

JE: Wow.

EV: I remember it took us two three days to get home.

EH: There's some good sites fishing in those pictures.

(talking in background)

DR: What were some values that were....

(EV adjusting seat)

EH: Some values?

DR: Some values that were important to you guys going up?

EV: (inaudible) When we'd go to the FAI I was trying to build boats differently then. (unknown) old boats. I buy em' old boat. I cut up (unknown) all the way around the side of the boat. Then I added four feet to the boat.. Make a fishing boat, used that for 7 years. The idea of how many fish you caught.

JE: What were some of the values.. Yeah of like.

(EV showing pictures)

EV: We got 26 fish in one drift. King salmon.

EH: So everything was based on the seasons and so our family worked together you know when fishing came around. That was the most important thing. Everyone had to be present and helping. And we still do that. I don't help process fish as much but it's still the same way but it seems like so much more.. It takes.. It's a longer process now isn't it?

CS: Well because we get less..

RG: We do less but we don't, we don't, we haven't fished for (unknown) for a couple years.

CS: Yeah, well last two years.

RG: And then we've been only doing silvers. Darrell, mostly Darrell.

EV: After I married my second wife, we had 41 years before she passed away and uh, she was my full time fishing partner. I told her how to run and bought a 100 horse outboard motor on a homemade (unknown) fiberglass boat. 24 foot 8 foot beam. And many times we filled that boat full of fish. I could remember we got so many fish one time that we, the wind was blowing so hard we had to jump in the water to keep the boat from sinking. And we threw the fish directly from in the water to into our boat.

Unidentified man: I was lucky I came from up north different type of fish that came down there all of those girls were busy. 'Need help I'll help you' so…

CS: But I think it was really important to that all the families and other families that were in need, or, um, provided for by fish too.

JE: You guys were helping them? And were any values that you learned at fish camps, or when you were commercial fishing and how did these values help you and how do you feel like they've helped your community?

DG: Take care of others I mean uh, this year alone my wife and I fished for 13 households. Umm people that couldn't get out so we'd get off work and but we'd get a call saying somebody needs fish and we'd jump in the boat right after work and go take care of it.

EH: You share.. Sharing and...

EV: A lot of times we'd do that for friends that come from Anchorage or different places where they didn't have fish. And we did, we'd give them whatever they needed to last through the winter. Half of the fish or something like that. Sometimes more than that too. I've never sold to my friends ever.

EH: With subsistence lifestyle you have to be, you know, the freezers need to be full. For the winter. Apparently winter so, moose, ring seal, but not so much anymore. But fish you know, that's a lot of mouths to feed throughout a long winter.

RG: Somebody wrote a book once called always getting ready and it's mostly a photo journal. A lot of it is of this area. Its an awesome book but that's basically our life out here. We're always getting ready for the next season. Like now we're getting ready for burning wood and cutting a lot of wood. Well be getting ready for the fishing season. So well start pulling out. Everything is always connected. Seasons are always connected.

EV: (unknown) what is his name, Parker, that wrote the book 'Always getting ready'

EH: Yeah James Parker.

EV: (unknown) Parker. His brother fished here. Commercial fisherman for a while. And I thought he was a little bit (unknown)

DG: One of the big values that I learned from my dad and my grandpa was protecting your investment. You always, at the end season you always got to (catch hook) the net before you put it away. And put it in a dry spot. When they get older they have to be taken care off because they'll start (unknown).

EV: Every drift we came home. I insisted we spread the net out on some place. Patch every hole because it might be too big of hole by the time you get to fixing it you know.

DR: Were there certain rules for harvesting salmon in your guys family? Or fishing…

EV: There was hardly any in the beginning. (inaudible) Before I started fishing, my dad used to tell me about there was a tremendous amount of red salmon in this river. And most of the years that we fished it was some red salmon. But slowly coming back. You guys have to take care of them. Counting fish and stuff in their streams are doing a tremendous job I think. So if its coming back its coming back. Lotta people don't agree with me about that but a lot of things… I disagree with it because I always wanted to be completely (unknown)

(Phone ringing in background, with someone answering it)

JE: Are these teachings you guys talked about, taking care of others, sharing, helping others, um, taking care of your net, investments. Do you feel like this is still being taught and followed today?

EV: I think it is to a certain amount, they're beginning to know that if you don't patch your holes in your net you know. But nets nowadays are very very expensive and so you try to make what lasts (unknown). In the early days the difference was the quality of the net they were originally made with salmon twine. 12 strand salmon twine. And depends what size fish you want you know. It's always the same webbing you use. And then uh, I was gonna say something and I lost my train of thought.

DG: I don't think its being uh, shown or um, shared with as much as it should. I do know we raised our kids learning how to do it. And uh, I work at the juvenile jail so we get a lot of kids that have never done it before. Mended nets or even gone out drifting. And each summer I bring the kids out and we catch salmon and teach them how to process it and share with elders.

CS: Even the art of cutting fish.

EV: One thing I see nowadays, what they didn't used to do, is plug streams with the net. Slide streams and they set the net all the way across. And that's normal. We let some go up the river in the early days.

JE: In the early days they were doing that?

CS: Even in the early 80s, 90s. I remember seeing a lot of set nets along the river. Like I don't see that as much as any more.

(Multiple people talking at once)

CS: The amount of set nets that they have now are nothing compared to what it used to be.

JE: Do you know how come that changed.

CS: Probably regulations you know. There's strict regulations.

EV: Well in the early days there was a lot more fish so you'd use a shorter net. And you, you uh, if you set the net it was always had to be in sort of eddy. Cause that's where the fish gather you know. To rest.

EH: Cold water.

EV: I was looking at these boats here. Both of these boats…

DR: Do you guys feel…

EV: The boat is how wide it.. Wide (unknown) ribs.. FA used to get a lot of their..

JE: Those are really good photos.

EV: There…

JE: They're awesome.

EV: Uh, up like that. In cases, in wooden cases. And I take the cases apart very carefully and save them. Cut em down and that's what most of the ribs are made out of. People said I couldnt do that but I did it. I saved a lot of money by using marine plywood. Because marine plywood is (unknown) cost you know. I used.. I take in here, plywood bolts (inaudible) Of powder… boat load. And I fixed it myself and fill the holes before I.. I took cutout of impurities in the wood before I put it in the water.

DR: Do you guys feel like your knowledge and or value are reflected in the fisheries management today?

CS: Less and less. Yeah. But on the other hand though I think that the management is very very necessary. Yeah. Today. Because the fish numbers are dwindling so rapidly. Um, I.. Listening to other members of the community that have you know, subsistence fished all their lives I don't think everyone really agrees, understands the cycle of the salmon, the life cycle of the salmon. And how necessary for them to be able to return to their spawning grounds. To rebuild those numbers. Um, I just don't think that's…

EV: I think its…

CS: People are interested enough to know that. The importance of that. So… You know we all saw what it was like when salmon were plentiful and everybody was getting their share of fish but um, steps have to be taken to be for them to, for those numbers to come back. If they're ever going to come back. I'm kinda doubtful that they will.

EV: People used to wonder about. I got off work generally the fish season that already opened before I got off work. So I got everything ready a day or two before that. Then I'd go out after everybody was already. One time I remember we were out on the river on the other side of that island over here that's disappearing. I went out everybody, there was maybe 600 boats between here and the mouth of the river fishing for salmon. And I took a look and my wife said to me where we gonna fish? I said I'm not gonna go below here cause there's too many. But I took a trip around and there were fishing either close to the shore or in the side of the river. And I set my net behind the last boat upriver and I got by far the most fish that first drift. Just by boat full of fish. And the guy ahead of me I knew very well from all my life. And when his wife 'how come you always catch more fish it was just ahead of us' but he was too far from the shore. I said you ever notice I take my cap off and I stick my head under water and I call the fish. I had to, (unknown) This is how I do it. And I stick my head under water. His wife got biggest kick out of that. She said 'I think he does that cause I always see you sticking his head over the side of the boat'.

Another elder: And Darrell grew up slapping the water with the ore. Scare the fish and make them come into the net.

JE: I seen an elder showed me that too here. With the paddles.

(multiple people talking)

EV: Silver salmon like to run in shallow water. And if there, you're close to the bank and your net is quite aways out. If you take it slap the water with the ore and all to your net.

Elder man: We tried it this summer and we broke the ore. (laughs)

JE: Uh. Do you guys want to share any more on or like.. How you feel that your knowledge and values can be better reflected in fisheries management?

DG: I agree with what Connie was saying about its necessary. We're still able to fill our freezers um, with the amount of restrictions that are being placed on so that that way its sustainable and uh, we even go above and beyond like my mom was mentioning the last couple years we've avoided going king salmon fishing and we started doing silver salmon. Smoke strips, um, just because… With it so I appreciate what managements being done and we're still getting our fish. (unknown)

Elder man: Salmon are coming back.

JE: Yeah okay.

EV: But you can (unknown) just up here shortly. This time of year there's gonna be nets set all the way across looking for.. They're selling whitefish and late run silvers. And they sell them locally I think that should be watched more closely cause your stopping you know, the majority of the fish. You could see it in several of the stream along the.. The airplane flies along.. You should be able to see them land and correct that stuff. Because you're stopping the fish from going up to spawn you know.

DG: Um, one thing that I would like the management to do is there's a lot of families that are less fortunate than us to all have jobs, um a lot of people don't have that.. I'm wondering if there's like some special openings for low income families that actually, that utilize uh, a lot more salmon than what we do. Um, if they could hold special openers where people that qualify for food stamps or something to get out… Or little less restrictions.

JE: Yeah.

EV: they don't have a boat…

DG: Yeah a lot of times the motor will be broke down and they can't get out for that special opener.

EH: I know ONC goes out and fishes for people too…

DG: Like the villages.

JE: Okay so..

DR: What um, what do guys wish fish managers knew about your community and your fishing practices?

CS: I wish that um, well I think there's mentality that sometimes that fish managers think that people are just overfishing and just keep fishing, fishing, fishing, fishing. But I wished that they would have that respect for knowing that families really do fish, I mean it is just a.. Its always been the mentality to just fish for what your family needs and when you have your quorum of what your you know, what you need to feed that family for the winter. Then you don't fish. Theres no, I have not known any… personally any families that do that waste. You know that overfish um, and I think that sometimes that's the mentality of some of the fish managers.

EV: I think that myself the fish they catch now is the end of the run. There's still quite a lot of… They're mostly.. Its whitefish and sheefish. Early caught late caught fish that they leave their nets set day and night all the way across. You get there.. Boat you have to lift your engine up to get across the stream. So they are very illegal. But in the early days

they said it was much much worse than that. When they used to have red salmon canneries down the river from here. I was down the river when I swear all the way across the part of the river. There was a net all the way across.

JE: Is that because people there was an incentive for people to get more fish to sell them?

EV: No that was.. Commercial fishery.

JE: Okay.

EV: Well that came to a stop because too many people kicked them out.

DG: So I know my dad told this, said this opinion just about every year we were commercial fishing. Was uh, ‘I wish the management would take a closer look at the migrating salmon um, coming from the deep sea trawlers’, that they are saying that they don't come up these tributaries but I don't believe that because we witness it were we’d have commercial fishing and the runs would be excellent. And then right as soon the there’d be an opener down in Bristol Bay. It would be like uh, curtain being dropped. And uh the salmon just be gone. Um and then Bristol Bay would catch record amounts of salmon every year even now. And were still continuing to have limitations and what limitations is Bristol Bay having? Because biologist says that um, those salmon are migrating up here but we've seen it while commercial fishing.

CS: One of the things that I have thought about a lot too though with our river is how much of the contamination affected the runs um, if there has ever been a study on that as far as the number of salmon that are coming our river as opposed to like the Yukon or Bristol Bay area. Because we had that seawall of cars but not so very long ago you know. 35 years ago. It was a seawall of cars and there has got to be contamination from that that has lingered over the years.

JE: Yeah contamination has been brought up by several families. Concerns about contamination. And that kinda ties in with the next question. What worries and concerns do each of you have about our salmon fishery today?

CS: My biggest concern is the mine that proposed Donlin mine. I’m extremely concerned about that. Um, not so much for in our generation maybe, but for our children and for our children's children. What is the world going to look like here 40 years from now? After that mine has gone in and you know. They can't guarantee it 100% containment. There's going to be seepage and they say 98% but that’s not good enough. To say that it should go away its affecting way too many people.

EH: And then you hear…

CS: This has been the lifestyle here for hundreds of hundreds of years. Um I think that money is just not important enough to risk lives.

EH: On NPR somewhere in the world where there's a big spill at a gold mine. Almost.. I heard another one yesterday morning, I was laying in bed listening to the radio. There it was on NPR another big spill somewhere down south. In South America I believe.

EV: Even the best years of fishing…

EH: Seeping into the streams…

EV: That fisherman gets a small percentage of the price of that fish. It’s the buyer and how they manage. It makes most of the money.

CS: There's um, there's a community out in (Libby) Montana. That um, we have a sister that lives down in that area. But I don't know the year that um, the contamination was first discovered. Um, people started coming up with cancers, different forms of cancers there. Families that it wasn't in their DNA, it was directly linked to that mine. And has now affected other people. Generations later they're still having people with birth defects. Our sister Lisa has known somebody that had um, lived in that area all his life. He was native american. Probably in his 50s or 60s at that time that his now since past from cancer. Um, but there have been recorded number of birth defects that were that affected that area and different families, generations born later. You know and that's terrifying.

EV: Weve just seen it. Fished the other up (unknown) fish deforms.

DG: The salmon that are dying off were doing as much what we can to sustain them...

EV: My son in law one of them was a high sea fisherman. And his hobby was taking pictures. Movie pictures. My daughter in Anchorage has all the pictures. She’s a widow now. He worked on a what you call it you know the big harvesting boat. But he done all the (unknown) great huge fish nets, God only knows how long their drift was. Truck full of fish. And he said they're throwing away.. You know their, (unknown)

DG: By catch?

EV: Throw the King salmon back in the river but he said just a very small portion that are being thrown back of the kings. But you get a mix of thousands and thousands of fishing them. In a net you can't tell what's in the middle of it. Or even on the side of it. Because there's so few in number compared to the rest of the fish.

(Looking at pictures in background)

DG: My concern is uh, is the environmental impacts. Where we have fish salmon that are dying because of the water temperatures were too high this year. And whatever work we do to sustain its uh. I mean we got a lot of environmental issues that are costly. And then I when not talking about salmon but um the grey whales that are passing away. My theory is that Fukushima nuclear discharge is affecting their migration paths now were starting

to see a lot of those environmental concerns. Putting them in the water and water temperatures warming.

EV: When I lived in Aniak I built a log house and we lived down slough. My son was down the river backpacking around and saw fish in the shallow water. And nobody, no Fish and Game was stationed in Aniak. They didn't know what kind of fish it was. It looked like kinda like a monster. But it was only about that long. I saw bottom fish that look worse. Bottom fish I've seen look better than he did. So God only knows where they come from. That was in the 50s.

DR: Do you guys think there could be any improvements with the concerns you guys have?

EV: What did you say?

DR: Do you guys think that there could be any improvements with our fisheries today?

CS: I think we could help prevent the decline I think that it's still declining and you know it may never recover but yes there's things that we can do to help slow that decline you know and regenerate and start some regeneration but it would be like in the way of more trying. Nobody ever wants to hear it but maybe fish for (unknown). Um, for certain period of time or really limiting the numbers even more. Stopping the mine from going in.

EH: What about the other (unknown) Um, like we get stocked and they're fishing up there and they're posting pictures of all the fish that they're putting up and everything and the people down get a little PO’d about that because… What about so and so? He caught all those hundred and some fish last night. They fish all night and they cut in the day and they show the smokehouse full. And we were limited to 5-10 pm or so.

CS: And did those.. And did those, you know I understand it’s near the end of their life cycle where they're going up and spawning. But have they reached their spawning grounds yet? Maybe they're not getting up there.

(Multiple people talking)

EV: One year in September in Aniak I was moose hunting and I had these kids mother with me. We were coming home and we there was a blind in the slough going into the timber cut. We never were in it before so we decided to go in there and see if there was anything and it was (unknown) pretty good side lake. (unknown) millions and millions of salmon that tall. And you can see em very very plain the water. Three days later we went down to the same lake brought the kids along and there was very few fish. Already migrated out to the sea. So they go out in batches.

JE: Out there?

EV: About the size of a barbie. That's just a few miles out of Aniak on the Aniak slough. I lived in the Aniak slough myself. And just a short ways down to that (slough) but there's out to be a lot of those places along the river.

JE: So before we get to the last question, one of the other things we wanted to ask was what you felt worked well and what does not work well with salmon management today.

CS: I think the counting, you do a very good job and I think it's a reliable way to do it. Um, the uh, closures are sufficient. There not what everybody wants to see you know everybody thinks there's not nearly enough openings now. But I think it's necessary.

EH: I think it's fair.

CS: Yeah. I think it's fair.

EH: I think everybody who wants to fish has the opportunity to. My concern is the people that, it's like you said, people with broken engines. They can't afford 400 dollars to go fix theirs. So they miss out on those kinds of things. So some of those openings are simply missed by people who aren't able to access the river.

(Phone ringing in background)

CS: Um, I think there are a lot of people that think that um, fish and game, and you know people regulating the fish are the people that are taking the fish off the tables and that's simply not true. It's really, it has to be done for to bring the salmon back you know. There's any chance of that. Um…

DG: A lot of people would get upset with me for saying this but I think there needs to be more restrictions and less openings because even though that we are being restricted we still haven't gotten into ever situation where we felt like we did not enough fish. Um, so even though they are restrictions we are staying out longer to get what we need. Or going out more often to catch what we want so. Even that, even though we've dwindled our counts of what we used to put away. We basically cut it in half. It's mainly because of the families that were providing for is smaller. And so were still going out and catching quite a bit of salmon. And were only catching what we need.

EV: But the countries about to change with the population you got. Populations growing fast. And everybody has a big engine and fast boat and they can get to the fishing grounds that they know are very good and they its overharvested some places and…

DG: Yeah I know I have a few friends that go down and fish by Quinhagak when there's limited king opening up here and they'll go down to Quinhagak and catch what they want and so it's not, the restrictions that they're imposing right now are not very, not strict enough. I think they should be stricter.

CS: I do like that um the rules that were if you're not, I don't know if it’s still um, in effect but if you're not living or residing in Bethel that, you can't fish. You can't subsistence. Is that, am I correct?

DR: Yeah.

JE: Federally qualified users?

CS: Yeah.

JE: When the feds are managing.

CS: And I’ve heard some people that come that are from here that live outside Bethel in Anchorage area. That complain about that. But that’s their choice you know. They choose not to live the subsistence lifestyle. Then they.. I think its right…

EV: They come from thousands of miles to harvest or fish.

CS: You know it's sports fishing.

EV: I don't think there's any restrictions in (Washington) fish that goes down to the airline out and goes home with them.

JE: Sport fishing.

EV: Fisherman. I’ve seen half a dozen fisherman put 5 boxes and probably at least 5 fish in each fish box. And they're… theres a lot of that goes on you see that all the time. When they get whats in that box. Nobody knows how many.

DR: What do you want to see for salmon and people in your region in 40 years?

EV: Pardon?

DR: What do you want to see for salmon and people in your region in 40 years?

EV: I’m sorry…

CS: Darrell can you repeat that to grandpa please?

DG: Uh, in 40 years what do you want to see how do you want the salmon…

EV: I would like to see the salmon (unknown) as they were before. I don't think.. I’d like to see it. Keep on. Uh, monitoring how many fishes goes up the river. As many as you can. I’ll tell you something that nobody mentioned yet. When the first big motors come out, outboard motors. You're going up the river. I don't care which river you go up. If the waters slow, and you have a big wake and ride like hell with the boat. This goes on all the

way up to McGrath and beyond. If big motors go up this small stream and have big wake, you go behind them and go up to the brush line there's all kinds of little fish up there. Never get back to the river again.

DG: Um I'd like to, just because I enjoyed growing up and being together with the family and processing the salmon um, and the stories that we share I really hope that my grandkids are gonna be able to have that same experience and being with family and working together as a team to get ready for winter. And I really hope my grandkids have that opportunity.

EV: To me there's nothing better than taking your family out and catching your first big king salmon. Prepared to take the guts out of it and put all kinds of stuff. And build a big fire on a sandbar. Doesn't get full of whatever you call it… Coals.. To cook your fish underneath it. Underneath the coals. I don't think there's a better way in the world to fix a fish. Depends what you put in it and how good it tastes. Even if you put nothing in it it still tastes good.

DR: How about you Ruth? What would you like to see?

RG: I'd like to see my great grandkids to fish…

EV: We don't do that like when my kids were little. We always went out on the weekend. Sunday especially. Wed go up the rivers aways and build a big fire. And have a lot of fun then one of us, two of us would go out and catch fish and dress it up just the way we wanted with being all kinds of stuff to put in it. Wrap up in tin foil and everybody wanted some. Everybody even that passed by you'd wave good friends they'd come and have some or some of it because there's was always more than what you could use.

CS: Um you know Alaska Native Medical Center, part of their vision is that I see it all the time on TV. Um, that Alaskan natives are the healthiest people in the world. And forty years from now if they, if thats going to part of their vision then we have to go back to the land I think and stop eating so much processed foods you know. Everything is here on the tundra and in the river and in the sky. And that if families were to go back and really truly become subsistence again. Not, not talking 100 percent today we have so much cancer going around. Its getting worse every year it seems. And if our river is not polluted and full of fish and the lands are you know not polluted with whatever chemicals are out there um then thats, it could be a reality.

EV: But you know every town along this river and every river that's in Alaska. People wanna live by the river. Guess where they slop goes in their toilet. Water goes, it goes in the Kuskokwim River. But you could, it's a proven fact you could take a dump to five gallon bucket of waste in the river. If the, you could go down there and eat the fish that's in there, there's no contamination. 90% of the time. So water clears everything up fast.

JE: Does anyone else have anything they'd like to share on that last question. Otherwise I think we'll wrap up here.

EV: You have to pay for all this… (everyone laughs in backgrounds) (end of interview)

INTERVIEW PARTICIPANT: HENRY KOHL

Interview Date: 04/05/21
Interviewer: Janessa Esquible (JE) & Danielle Lowrey (DL)
Location: Virtual
Home community: Bethel
Transcribed by Avery Hoffman

Henry Kohl [HK]
Danielle Lowrey [DL]
Janessa Esquible [JE]

DL: So to introduce myself I, Danielle Angilan Lowrey. I was born and raised here and I went to school at UAS in Juneau and I've been working with ONC for three years as a fish technician and I just recently started the Fisheries Biologist position in January.

JE: I am Janessa Ii've been, well I am from Detroit, Michigan and I moved to Bethel about five years ago and have been working for ONC as a Biologist and now more recently

HK: Has it been that long?

JE: {laughet}

HK: Time flies when you are having fun I guess.

JE:{Laughet} yeah it does, let's see and Henry can you state your name, your age, your home community, and your cultural affiliation.

HK: Henry Kohl born in November, 11, 1957 so that would be 63 years old born in Bethel and I am Alaskan Native, well half native I should say. But

JE: Half Yup'ik?

HK: Yeah

JE: Okay, thanks alright and Danielle if you can

DL: Please tell us a bit about your background, where you were from, how you grew up and what connections do you have to Salmon.

HK: My connections that I have to salmon? Oh boy, okay well I guess I was raised right here on the river in Bethel and first time I went fishing I was probably nine years old and that would put me right at about in the mid 60s somewhere in there. And well, what was the other part of that?

DL: A bit about your background, where you are from, which is Bethel and how you grew up.

HK: Well I guess I can say I grew up right here in Bethel, fishing was a big part of it. I commercial fished when I was younger and then a lot of subsistence later on for myself, my sister, my uncle, lot of family. And other few others along the way but well I guess salmon is a, it's a big part of subsistence here for the people on the river. That's, it's a the majority of the subsistence I would say.

DL: And how was salmon fishing like when you were young?

HK: The salmon fishery? Commercial fishing or the subsistence fishing?

DL: Could be both. How were they different then today?

HK: Well subsistence fishing was, see I come from the family that Bethel is named after well the old Bethel. The new Bethel is the Orutsararmiut is what is across the river and it means the sod for your mud house. The place where they gathered the sod for mud house. But the old Mamterilleq was the big smoke house that was across the river and that was my uncles smoke house. It was a three tiered smoke house that was huge and he had a trading post so they did a lot if fishing. They had somebody fishing all summer everyday, two women on the beach cutting fish and they smoked fish all summer. They'd funnel those mainly dog salmon was the biggest one. They'd catch a few king salmon for subsistence for themselves, Dog salmon for the dogs and dog Salmon to trade down on the coast down there. So what they did was they funnel thirty two dog salmon to a bundle and they'd, they barge those and put them on a barge my other uncle would Henry Madson Sr. would take, put those on a barge he worked for Tony who was my uncle who owned the smokehouse and they'd barge those, those salmon from right across the river above the old airport where the big smoke house was they'd barge those all the way down the river and trade for Seal Oil, Herring, and all the dried food along the Coast. And then come back up and trade those in the store all winter. And well I guess my mother subsistence and the families came out of there and was Molly {inaudible} my mother all lived with this Japanese guy. He was the one that did all the trading in the store and their subsistence came off of the king salmon early, some they ate some king salmon but mostly a lot of dog salmon.

JE: Really they mostly ate chums?

HK: The majority of the fish back then were the dog Salmon.

JE: What years were that in?

HK: Those were, that was geez probably 40's and 50's. Late 40's early 50's right in there.

JE: Okay

HK: And then I didn't start fishing myself until or with my other cousins, I should say from the same family until probably the mid 60’s and it was mainly king salmon and there was lots of them. You could walk across the river on them and then probably in the I started remembering commercial fishing well they were doing commercial fishing then. But it was real small scale and it was, not sure what size nets they had but it was pretty small scale. And it was five dollars a king salmon and KPC slabbed some and then later the Shagg brothers came in [and] got a little bigger because they were able to buy more and it was still five dollars a fish and were bringing hundreds big loaded boats of King Salmon bring’em in five bucks a piece. Well I shouldn't say hundreds I should say more near hundred you know. I remember standing on the barge looking in the boats and they are just filled with king salmon. Five bucks a piece they'd throw them up on the deck on the barge and the whole deck would be Just covered with them. Processing would take two or three days after an opening processing but. {interruption} Part of me?

JE: Were these people processing at fish camp or were these like actual like commercial fishing processors?

HK: Commercial fishing processors. Yeah, he had a barge right off the beach it was a pretty good size barge with a house and tin covering over it, and it was open on each end. And they boxed the fish in there and that was kind of the start of the bigger commercial fishing. And course they had openings, had to have a commercial license that was just the start of the commercial fishing here. And subsistence was still wide open, and what I remember about people eating king salmon was it was too rich for them. So they wouldn't eat as many they'd eat some and they mostly made dried fish out of it. Most strips very few strips, and then they would salt the bellies but it was too rich you know, most people complained about being too rich for them. So they preferred the dog salmon.

JE: WOW

HK: And they'd, they'd cut {inaudible} the king salmon they'd make the big blanket. With the, the cleats on it, you know. It was too rich for flat fish, but the dog salmon most people made flat fish. Kind of like kite shape with a stick a cross it. And there was no cleats, they would cut it like ⅜ to half and inch thick then when it dried it was like a quarter inch think but kind of flat no cleats. And they would literally do hundreds, everybody had a dog team and that was the dog food. Good ones they kept for themselves and the bum ones the dogs got you know. {laughter}

JE: Really, I was wondering what are the cleats? What does that look like?

HK: You mean, the I mean the slots. They normally cut.

{Hand gestures}

JE: Okay I see what you mean

HK: Nobody makes flat fish anymore. It's a different style of dry fish. You see them now and you know they all got the cleats in them like in the pictures.

JE: Where they cut them across and

HK: They start on the inside and go around the rib cage and just kind of follow the skin. And they never put the cleats in there. Put a piece of willow, you know. Ten twelve inches wide stuck it on each side by the fin and you left the backbone hanging off, off the table and the backbone would go over one side of the rail they put cleats in that but then it was flat on the flat part if it came out good that was for them. Dogs got the backbone.

JE: {laughter} and I had one more before we go on about when, so you were talking about how they used to do a lot of trading. Bringing the salmon to the coast and then they would bring up seal oil and other things, when did the or how long did that go on for? When did you start seeing it change or decline in the bartering?

HK: Probably right, about the mid 60’s late 60’s maybe right in there.

JE: Okay, gotcha

HK: Yeah that was, that was they bundled them up thirty to the flat sides you know, they'd put them meat to meat and skin to skin you know. And they'd bundle thirty two in a bundle and tie them up and that's what they traded.

JE: I got ya. Okay

HK: Smoked, all smoked.

JE: Okay thanks

DL: Next question, how did you learn to manage, steward, care for salmon when you were growing up?

HK: Pardon me?

DL: How did you learn to manage, or steward, or care for salmon when you were growing up?

HK: Mainly by watching I guess, and helping I spent my younger years every year go to fish camp. We never had a fish camp in our family but I'd go to my uncle's fish camps and stay there all summer. And then some of our other cousin had camps right close to him and we helped out with all of that. All kind of shared the same thing, you know. I didn't participate in getting any of the food, I just went across there and lived with him and helped him. It was kind of carefree like you know the.

JE:{Laughter}

HK: Go to fish camp and run around as a kid, you know. {laughter}

JE: Were there things that you remember your uncle or cousins or even your parents or grandparents at home or at fish camp about like maybe not wasting or how you, I guess how you went about fishing being aware of other people and their fishing spots things like that.

HK: You know there were they tried not to waste any food. It was you know of course if fish was bad you know they would throw it out, you know. And they see a few of those you know like the Ichthyophonus that we see now. I'm sure it existed then you know.

JE: Okay

HK: Cause they talked about it and they threw those out you know. But they tried real hard not to waste theirs because it was hard to come by, you know. They even took you know the scraps that we throw away now they dried all of that. And they even took the stomachs out of the king salmon and dried those. They turned them inside out, cleaned them up and dried them up.

JE: And actually to go back up to the first question you shared a lot about when you were younger, are there any big differences that you're seeing today cared to when you grew up and how things were commercial fishing and then you said that now you subsistence. What are some of the big difference from your younger days compared to how you see things now?

HK: They fish more king salmon.

JE: Now?

HK: It's more geared to subsistence is more geared towards king salmon and not so much dog salmon.

JE: Do you know why? Or do you have any idea why that might be?

HK: Well I guess it’s all people's balance {Inaudible} everybody is kind of maybe they are all hyped up about Omega-3 who knows, I don't know. {laughter}

JE: Okay so that is one of the big differences?

HK: I, I think it is.

JE: And then you mentioned earlier that it was wide open all the time, is that different today?

HK: Yeah you know what we never had any closures like this, the restrictions that we fish with where it's open and only reason, only only thing they'd have openers for was commercial fishing. Rest of the time it was open 24/7 for subsistence. Unregulated.

JE: Gotcha

HK: And that all started happening in I think that was 2014? 2015?

JE: the regulations?

HK: For subsisters before then the only ones that got regulated were commercial fishing.

JE: Gotcha. Okay

HK: Oh my god the commercial fishing back then was phenomenal.

JE: Really?

HK: Just fill the boats, fill 'em. If you couldn't get three hundred fish they were dog salmon or silver salmon first drift you weren't a very good fisherman you know. {Laughter}

JE: In one drift three hundred? Dang. WOW.

HK: Yeah

JE: Were most of the commercial fishers people from the region and most people that were benefiting from commercial fishing people from here?

HK: Yes. It's there's I don't know of any outsiders that lived outside you know that came back just for commercial fishing.

JE: Okay

HK: I would probably say like 98% all regional fisherman

JE: Okay, thanks

DL: So in my previous question you said that back then people were trying not to be wasteful and trying to be resourceful with their salmon, is that still being taught and followed today?

HK: Well it is kind of a hard question, cause while you see some people now they spend a lot of time fishing and putting up fish when there are openers and then you drive by later and the fish are still hanging there you know {laughter}. Makes you wonder you know what are they doing with those fish {laugher}. You know, back then people lived right there with the fish and they'd move to fish camp and stay there. You know they didn't run back

and forth if you had a fish camp you put up a tent or you had a little house. The richer ones had a little cabin but mostly tent, tent frame and they stayed there all summer. There was, there was not much fishing down river because the people from down there would come up and from Johnson River up this way the fishing was better. So the coastal people came up and they had their own camps and the tundra people Nunapitchuk and Kasigluk and well Atmauthak was kind of new. They came and they had their own place, there was Tunt fish camps and Nunap fish camps, Bethel fish camps, Akiak fish camps, you know, the Kwethluk ones everybody kind of had their own area, you know.

JE: Yeah I have a couple questions about that. I was wondering couple things so, you mentioned about people live right there at camp pretty much all summer. What do you think, why do you think less and less people are spending their summers at camp.

HK: Probably because of the motor transportation. Outboards have gotten bigger you could go farther faster, and they are more reliable and there's more money available for gasoline which was. Back in the day gasoline was cheap but Natives were poor they didn't have money for gasoline a lot of them were when they fish. They didn't run their motor like they do now, they had oar locks on the boat and they would oar. They were wooden boats.

JE: Wow.

HK: Instead they'd shut the motor off and it was manpower fisherman. Later on as commercial fishing frequently they used more motors. And as Yup'ik Eskimos got richer now you see them with all 50,000 dollars trucks you know. They were able to run their motors a little more {laughter} and I think for that reason they go back and forth to their fish camps.

JE: Okay

HK: You know and cause it's now you know even if it's twenty five miles away that's just around the corner. Before back then it was a couple hours to get there you know. 25 horse in a great big wooden boat you know. So…

JE: Easier to get there now. And then you mentioned there were a lot of camps that people would come up from all these different communities and you know we do our surveys in the summer. And I remember when I first got here we had more camps that were from people, people from different communities like Nunapitchuk, Kasigluk and now it seems like there's more and more people just from Bethel and were not seeing people form the coast or other communities. Do you know why that is?

HK: Probably from the availability of food. And the, and the hardships of subsistence, meaning there is less time to subsist don't get to fish like we used to. So we don't have as many fish. So why go through all the trouble and just stay there all summer instead of fishing and putting away your food, they are waiting for the next opener. See, see you have less time fishing. Less time working on fish.

JE: Okay,

HK: When they were at fish camp they put away hundreds of salmon. They would probably do. A common fish camp back then was probably two to four hundred dog salmon and probably anywhere from, I remember I kind of remember, about forty or fifty was a high number for king salmon.

JE: Really?

HK: Yeah, they dry those and make the big blankets {inaudible} but the main one they relied on was the dog salmon.

JE: Okay, thanks.

HK: Maybe it's changed too because of well, transportation you know.

JE: Yeah

HK: Back then they had to have dogs. To move you around and you had to feed the dogs so that was the dog food.

JE: They needed more chums. And then before Danielle goes on to the next question the one thing that I just remembered is you talking about sharing, when you were younger you fished and you fished for like a lot of your family and then other maybe non family members too. So it sounds like sharing is a value that you practiced all your life. Do you stil- do you feel like sharing is still a pretty like a pretty common practice or value that most people still share now?

HK: That's kind of a native thing they, they, they share a lot of their family yeah. They'll help you if you need enough. But you know if you are needy enough, but it's not like it used to be you know there was a lot of sharing back when I was a kid.

JE: A lot more sharing? Okay

HK: Well people were more needy, you know they were poor.

JE: Yeah, okay.

HK: But the houses you see now, you know they all drive big trucks and they all got big houses it's pretty common for this size of room, you know they would raise a huge family in this size of room. Cut cram people in the house you know {laughter}. So that seems like you know.

JE: Yeah

HK: They grew up in a home that was 24X40 and that was a big house

JE: Wow.

HK: You know. And today that's you know that's not a very big house at all. You know.

JE: Do you think the restrictions or is there anything else that is preventing as much sharing from going on or you think it's mostly just not as much as a need?

HK: Well I know there's an attitude change. You know I don't know what causes that. And well for instance if you would have broken down in 1968 on the river anybody, the first person that came along would have stopped and helped ya. But now you know same with sharing is same attitude as kind of like what I'm trying to explain here is, if you were to break down with an outboard on the river now you might be lucky if someone body stops by and in a couple hours you know, {laughter} and I don't know what that's from. I don't know whether it is you know that Yup'ik has become more westernized, you might say. Whether it has to do with money or they used to care for each other, they help each other a lot more than they do now.

JE: Thanks for sharing that.

HK: Maybe because the gene pool was smaller back in 1800 and everybody was kind of related still in 1960 you know {Laughter}.

JE: Yeah

HK: You know now the population's five times larger you know. You ask these younger kids nowadays you know who their grandpa is and they barely know them, you know. Everybody back then knew who they were related to, who their relations were, you know everybody. You know now its a, it's, it's not like that the younger generation they don't know.

JE: Really?

HK: They don't know who they are related to, their extended families you know.

JE: Wow, okay. Thanks for sharing.

DL: How did your ancestors manage, steward or care for salmon?

HK: Well they tried to catch as much as they needed, you know. Some of them early, early, early like my grandfather his day they had to make their own net so they spend a lot of their pair time in the sum- I mean in the winter time preparing for the summer time by making their net. They had to hand sew it, so the salmon they did catch. They took very, very good care of it because they didn't have much of it, you know. I mean you had to work for it. You had to go out and row, you had to make your nets so of course you

didn't want to waste it, you know. And the parts that we cut off like the collars now you know or even the tails you know and the heads, you know. Most people threw their heads away. They never threw any heads away, they either dried them or salted them or they buried them and ate them {inaudible} when they are half rotten what they call (pateq) stink heads you know. {Laughter} That they ate a lot of that but you know they and they'd take the tails you know the very tail and what little was left on it when you got done making dried fish they'd hang dry, and half dry them. And then they made those for egamaarrluk which is half, half cooked half dried half cooked.

JE: Oh the tails.

HK: Yeah so that with the collars the tails all the scrap parts you know. Like I was saying earlier you know. They'd even take the stomachs, heart, I don't know if they did the livers but my sister was telling me that they'd take it and they'd cook heart all the time. And they used as much as they could on that fish. I remember the part of the cuisine when they are kids we'd always, salmon soup, you know, you see who was going to get the eyeball cause we wanted the eyeball. {laughter}

JE: Eyeball is pretty good?

HK: They are good eating {inaudible} you have to squeeze the stuff out of them you know the ink {laughter} you {inaudible} but yeah they are good.

JE: They are pretty yummy and what do you know anything else about stewardship practices of ancestors like before we had all of this, this management. Even like before technically Alaska was a state before they were the Fish and Game or Fish and Wildlife how they managed or steward the water and made sure there was enough fish for everyone.

HK: Well they were kind of here just kind of here Fish and Wildlife was way in the background they, they had an office and they, they managed more towards birds.

JE: Okay

HK: And then the State of Alaska kind of was in the background and just of monitored as far as I knew of you know, there you know we knew they were there. There was no real you know I don't know they might of came around and did surveys for something but I don't remember. I think the first time I ever remembered the State of Alaska asking the Natives to kind of conserve the king salmon was I think it was right around the early in the late 70’s or the early 80’s I want to say. And that was kind of the first time I heard of any management on the State, they were asking all the users to kind of conserve the Kings because the numbers were kind of low. I kind of remember that. That was about it.

JE: Thanks

DL: Do you feel like your knowledge or values are reflected in Fisheries Management?

HK: Well I should hope so. {laugher} Well i try to share my knowledge and try to help manage through sitting on the ONC Committee here, chair the committee. Try to have good values where we help conserve and ensure that future generations get some of the king salmon and… Well I guess you might say that I don't think our, our views are really what was part of the question you're saying that.

DL: Reflected in management.

HK: Right, reflected in management I don't think our views are really considered to the Fish Commission. Cause they say that they have the elders and well I don't think so. They say they have the elders but when it comes right down to it we are here at ONC. If we were to speak our voice we'd have to go through our representative and our representative is one of fifty representatives. Which it has to be in a pool in order to get anything done. And they met once a year that's the only time that the only time that they ever listen to their elders. So other than that. I try to share with my experiences and my opinions with the current managers at Fish and Game, Fish and Wildlife and some of the major players that I know that are involved and try to get things done that way. I miss any part of that?

JE: No thanks so much. I was going to do one follow up just finishing taking some notes. So do you feel you mentioned, the Fish Commission specifically that its difficult to have your views aren't really reflected in their process. Do you feel that through the State and or Fis- like Fish and Wildlife service your values and knowledge are reflected in their management processes. Or do you feel like maybe not?

HK: Rephrase that, write that down.

JE: Okay, so you mentioned that your views are not reflected in the Fish Commission process. You stated couple reasons why which I really appreciate and so I am wondering is it any different with the State or Fish and Wildlife Service.

HK: Well Fish and Wildlife Service I know I'd have a better voice with Fish and Wildlife Service if we weren't part of the Fish Commission. Because the Fish Commission has three managers plus their CEO. And they have a lower river, middle river and upper river inseason manager and well if ONC was to pull out of the Fish Commission we would automatically have as much voice as the CEO of that Fish Commission. I know that, they've already told us that.

JE: Wow.

HK: They said we have to listen to everybody. You would have as much voice as they do. But Fish and Game, the Fish Commission says that they have the elders' voice but try to get it in try to get your opinion across to them without going through the whole process of having to get a pool of the representatives together to have the same opinion so that you'd have actual voice at the meeting. They say in that pool that they have the elders

well, you know how is it that, that they actually get that point across? So with State management they go to the Working Group under state management. The doors open Fish Commission doors closed every meeting doors closed. State meetings doors open anybody gets to speak anybody everybody they have the elders they truly have the elders.

JE: The working group

HK: The working group and they listen to people they make decisions based upon the testimonies that they do here. And I think it's a good process if the Fish Commission had something like that. It would be a good process it's a long process and you have to sit there and you know kind of something you have to tune out you know. {laughter} But it's a long process but it works. But the States problem is, is as the number recede on the king salmon so does their management they just keep lowering the numbers and you never know {Inaudible} five years if it continues under state management. With an objective of 40,000 king salmon you know. Which is, which is what we don't want.

JE: So there's a concern then with the state having constantly bringing the escapement goals down.

HK: Right

JE: As the population goes down there's kind of a shift in that bound and there might not be fish left.

HK: See where all that started was from was, in 2013 they messed up in their management by not closing it when they knew the numbers were down. So, what they did, they just had these openers, these random openers and it didn't work. They wound up with very low escapement, managers actually came out and apologized and as a result of that Greg Roczicka got together with ____ at AVCP right from ONC here and they came up with the 804 process. And they forced the federal government to come in and get those numbers back in line.

JE: 2013

HK: 2014 {inaudible}

JE: Okay

HK: 2014, 2015 right in there we started all that. And the Fish Commission later came in because of that process.

JE: Gotcha

HK: The Fish Commission came in later which Greg started from visiting the Yukon Fish Commission.

JE: Really? Okay.

HK: And they came in and helped put it all together and the dream Fish Commission that you see now, was not the dream that we had. {Laughter}

JE: Well thanks, that was all really, really awesome. Sharing got lots of good notes.

DL: What worries or concerns do you have about our salmon fisheries today?

HK: Worries?

DL: Yeah worries or concerns about the salmon today.

HK: Personally or as a chairman? {laughter}

JE: Whatever you want.

HK: Personally I'd like to my worry is, truly I think Donlin Creek is going to go open. And its, it's going to be another twenty years before they start dumping out, overflowing twenty, thirty years before they start overflowing or discharging from their from their holding pond. And while that's another fifty years from now twenty or thirty years they start dumping, twenty or thirty years of accumulation of heavy metals that are coming out along with all those metals that are going to come on our river and what's that going to do for our salmon? You know we got grandkids growing up you know. Who knows your kid might even be here you know. And are they going to be able to eat those salmon? You know, you got those fish going up the little fingerlings coming out and they're carrying all of that you know. What's going to happen to this river in fifty years? So that's kind of a personal thing but.

JE: Yeah

HK: But as a chairman my worries of what's going to happen with the fishing here is. Where is the Fish Commission going? Where they going with all of this? You know they say they are managing for 110 thousand as our objective here at ONC. They have kind of the same objective but they're going to be in control, Fish Commission is not going away. You know 804 processes is not going anywhere. So what kind of management are we stuck with? We got, we got, we got the three, the three managers. And we have one CEO and they all meet with the feds and it's all closed door. And nobody gets to have any input. Where is it going? The process they have is, it's not really based on people. People's needs, it's based on what they see you now, those four managers right there and truly I think it's based on the CEO's view of what is happening. And right now they use the state numbers they use ONC's numbers they'll have an idea of what's happening. And [leader; she/her] gets to put it out there and decide what is going to happen. She drags her managers with her and she calls, she calls BS on all the numbers and that's what she wants to do. You know she put it right in our face that numbers are no good. She's told the state that they are wrong and who is she, she don't bring anything to the

table. You know, all she does is come in with a big voice and pushes everybody around demands you know, where are we going with this?

JE: So that's one of your concerns then it's just the management with the current management state of management its not inclusive. Don't really know what they are basing their decisions off of.

HK: Right

JE: And there is no place, for tribal members, people living up and down the river, maybe just a few because the meeting are closed doored. So those are some of your concerns.

HK: Right {Inaudible} what's her view you know, you have to put yourself in her shoes and what's her view what's she looking at to based these opening off of and and closures you know. And trying to get people fish and you know I know that you know even theres low numbers they still have openings and you know it kind of backwards you know. Truthfully the true way to manage this fishing and to make it work is it needs to start off closed. And the model needs to produce itself, the state model needs to produce itself and then you should have openers. You see what I am saying?

JE: Yeah

HK: That's the conservative way but everybody is up in arms about it. It's a hard thing to live with you know even the twelfth that we have that came from ONC that we in this room put together from the closure from June first to June twelfth. You know that’s, you know that, that's stretch a closer everybody wants to fish, everybody. We based that on thirty-five percent of the run passing the majority of the fisherman here in Bethel. That’s what is was based on.

JE: So do you think that you mentioned like we should just start off closed? Do you think that announcing a few openers maybe like late may or early June is the way to go? Or do you think that they should wait until more and more come in, more and more data comes in and we hear like from fishers up and down the river before deciding we are going to have these openers?

HK: Well that would be the correct way to do it. You know but well I wouldn’t want to put my name on it though it needs to be {inaudible}. {laugher}

JE: Yeah

HK: That would be, you know that's I'm sure that they all kind of know thats that you know i've talked to people that are in the background and they all say that same thing that {interruption} but you know they say that, that's the way to make that model work and that's the way to get the correct numbers for escapement. Currently right now it looks like a good run, Bethel Test Fishing does there you know CPUE, Catch Per Unit Effort. You know as that number climbs then, looks like it's going to be a good run, and they hurry up

and have an opening and then it’s (shhhew} falls off then what? Then you just took a majority of your fish that could’ve been got escapement. You know that I'm sure that the running exists and to try to rebuild stock, but you just took the {inaudible} you know, so.

JE: I see, thank you. For this next question it says so the question is what are some concerns you have with fish management and research today, what's working well whats not working well, how would you improve it. I feel like you spoke the reason I'm chiming in I feel like you spoke a lot to this already. I guess maybe one of the things you could do if you wanted to speak more to what's working well. You mentioned the working group. That's a good inclusive process open door. Are there other aspects of management you feel that are working well? Or areas that you think really need to be improved.

HK: Well what I feel really working is the inseason number that ONC has. You know that, for the system that we do have now when there is an opener, how much fish are caught. Nobody knew before. And now we have real life data that, that is given to the managers even regardless of if they asked for it or not you know that was something that we started. Got a grant for it and started and now they use it because it's a real live number that works. Now they have an idea, a testament of per boat and all they do is an aerial survey and get the number of boats out there and they can tell how many fish are getting caught.

JE: Gotch ya okay.

HK: They manage for the, as run produces. But if it falls off the system that they have now it can be over fiction easily.

JE: Okay

HK: Almost what we did last year {Laughter} you’re expected to have a 90 thousand excess king salmon but we are what maybe 10 thousand. It's not even that.

JE: Doesn't seem like much.

HK: I think the escapement was what 86 or 68 or something like that.

JE: I think in the 80’s yeah that sounds about right.

HK: yeah

JE: The mid 80’s.

HK: Well according to the state in there objective is lower number 65 so according to them we met escapement.

JE: Well now that then so is that something that you would improve? You kind of spoke to this earlier with escaments you, you guys shoot for 110.

HK: Right.

JE: The states got a range 65K to 120 or whatever it is. Do you feel like that something you think could improve like maybe they should reevaluate that lower bound? Cause it might be too low or…

HK: Well in one of our last meeting when Boyd was here is that his name Boyd?

JE: Yeah.

HK: Boyd was here I remember I asked him to try to improve relationship with the state were and get higher numbers for their objective.

JE: Gotcha.

HK: Instead of lowering the numbers get higher numbers so.

JE: So that's another one?

HK: We know that the feds manage for about 110 somewhere in there.

JE: Okay.

HK: You know, they try to.

JE: Getting the state on board. Is there anything else that you want to share about management, anything else that you want to share about like strength, weaknesses. Improvements.

HK: Well I think we {inaudible} most of it you know, nice if the Fish Commission did use the elders. You know it's a hard thing for them, they don't want to do it. Because all the people the public over and in they have to listen to stories about how it was fifty years ago and you know it doesn't really pertain today you know so.

JE: But it is important to give them that time and space.

HK: Right

JE: Our elders okay, thanks for sharing.

HK: Yeah I think they can improve by having open door meetings and their mystery you know hopefully and if there is something wrong with their style of management. You know hopefully the representatives can get together and correct it.

DL: Okay, so what does indigenizing our ways of knowing about and managing salmon look for you. Look like for you? {Laughter}

HK: Indigenizing salmon management?

DL: Yeah, what does that look like for you?

HK: Well that's a mouthful. {Laughter} Okay. Well, well indigenizing it, well the people are more educated now. More of them pay more attention to managing management. We, I think our ultimate goal is to shoot for that through the Fish Commission. You know, so.

JE: To indigenize salmon science management through them?

HK: We got a long ways to go, you know there's ONC odes a little bit of what's involved in it you know we gather numbers. We currently got a grant to do a model and come up with our own model so that we could test it against the states I guess. You know that's basically what the Fish Commission wants to do. They were running, they were going to do that grant. But they had to have a tribe to do it. They came to ONC and ONC did well they are going to have numbers, course you have to have education, hopefully get more natives involved in biology. Biology just about fishes is not where it stops at, or starts at you have to have water shed behind them and the ecosystem that happens stains all of these you know the rearing and the return round. What about the feeding ground, got to manage all of that to have healthy salmon. There's a lot of stuff that goes on in that ocean that we don't know about. There's a lot of fishing, lot a lot of pollution, what about all of that biology you know. Then of course get the elders involved you know they're not going to be happy you know. Well, you got long ways to go for indigenizing salmon management. Long ways to go, were just touching it

JE: Can you think getting elders involved and you said more Alaska Natives involved with biology that that would also encompass these people bringing traditional and indigenous knowledge? Do you think, do you see that kind of being a part of Indigenizing Salmon science and management?

HK: Yeah I think, I think, the elder knowledge in there in the past, what they have seen in the past and what they've heard about the past, all of that being, being a part of management is. I think managers are willing to listen and learn and maybe use some of that towards the objective.

JE: Okay.

HK: The current managers. I'm sure that if it, if it went to Indigenous the 100% being managed by Indigenous people I'm sure they make that a lot more heavier then the current managers would.

JE: I see, okay, thanks.

DL: What do you want to see for salmon and people in your region in forty years?

HK: What do I want to see in four years for salmon and the people?

DL: Forty, forty years what do you want to see for salmon and the people in the region in forty years?

HK: Forty years, well I guess it would be nice if we had more indigenous involvement in the management and of course I'd like to see it sustain itself you know. Currently now you know we look at what happened two years ago with the, with the chum salmon you know. There being one of the main fish out here if the king salmon don't come in then everybody goes to dog salmon you know. And you remember what happened with them is the water was so warm they were floating down the river belly up, you know I mean a large number suddenly disappeared you know. And it was a real eye opener for people, you know it's, we take these salmon for granted you know and all of a sudden they're gone. Forty years I hope there's stil… here it'd be nice to see my grandson, I have seven, it's a tough battle right now. It's kind of like, the breaking ground where the indigenous people are getting involved in the management and it's going okay I think you know as far as the feds are going just trying to stuff down their throat but. You know that's part of the, part of the, the, the management skeem i guess you might say when they came in the country you know. That was forced upon them with ANILCA. And well as far as the State goes, they don't care about us. They don't want to hear from us {Laughter} actually that's not true.

JE: But not recognizing tribes.

HK: Right they dont recognize tribes but I feel they do listen to us.

JE: Okay.

HK: I think, I think ONC gets a lot more done, did get a lot more done earlier on and I think we have a pretty good open door with, with the state you know. They don't seem to brush us off, you know. The feds were welcome to walk in anytime over there. I think they have an open door every time indigenous management that's a long ways away. You know most people nowadays even of all the numbers that we have talked about in the last ten years, people are starting to really get involved and paying little more attention to what's happening with the king salmon. The numbers involved and they are kind of starting to understand when we talk about numbers. But the majority of them, they still don't know what that means. The salmon are still taken for granted the return out there, when you pull up to a fish camp, “How come we can't fish?” They still don't understand the whole concept of conservation and maybe there won't be any in twenty years. Tough battle though good battle what a tough battle it's worth a while.

JE: That's the last question that we had for you. This was a really amazing interview. Quyana for all your sharing.

HK: That's it?

JE: Yeah

HK: You're going to give me one hundred bucks for…

JE: You spent a lot of time with us {Laughter}

HK: Maybe an hour

JE: We really honor and value your time. Do you have anything else you want to share with us?

HK: What can I say, well, I hope that, I hope that through all the involvement that all the natives have with donlin you know the donlin process it's in the process of getting approved right now. But I hope that through all of that the native tribes start to understand what the long term outlook is going to look like. I hope that they come together enough to where they stop this project. So you know the earlier mining project like, like Nyac and Kalmakoff and Flat and all of those that tore the country up and contaminated it. There's a lot of, lot of a lot of people working, they learned a lot. And good people came out of it, because of the work ethics and you know the things they learned and the monetary values and you know all the involvement that local people had came out positive but you look at what's left now. You know, you know we are still dealing with Red Devil, {inaudible} high levels of Mercury forever you know and. But in the long run it's not going to be good for our country, you know. the, the short view of that is up river is going to get rich they'll probably be some entertainment here in Bethel, might see some of that you know. There will be a lot of people willing to work up there at first but then I think they are going to bring their own crew in {inaudible} what's happening. And the local people will get pushed out after it starts to go and who's going to be left with the clean up? Canadian owned outfit, who's going to be left? American taxpayers, they clean that up.

JE: Wow.

HK: I hope the native people come together a little bit, maybe they will stop it but I don't think so. I think Calista lets them go and up river wants to go real bad.

JE: Are you a Calista shareholder do you have friends and family who are?

HK: Yeah

JE: So how do you get your voice through to them if you don't want the mine to go forward. Is that even an option now.

HK: Maybe you could get ahold of Robert Hoffman that sits on our committee, he has some of their lessons.

JE: Oh really?

HK: Robert Beans, who's the other one, I think James Charles.

JE: So there's a couple then that can only there's only a couple that you have to go through. To get

HK: Lessons yeah.

JE: Wow so you can't go to Calista directly.

HK: Well if you know the back door ways maybe

JE: Wow.

HK: But they don't want to hear from you and you'd have to do a lot of lobbying to swing the board

JE: Wow.

HK: Yup, but if you know the you know the board members or you have a way in on the board members you could be influential. But if you think you are just going to walk up to the front door {Laughter} you won't get your voice heard, that's not going to happen.

JE: Okay, well I know that they are looking for subsistence users for some sort of subsistence committee for Donlin. I don't know if you would be interested, I could look it up when we stop recording TKC [the Kuskokwim Corporation] just put out a notice not that long ago. Might be good to have someone like yourself.

HK: TKC

JE: Yeah

HK: Really?

JE: Yeah.

HK: Middle river? Looking to shave their subsistence?

JE: They are looking to establish a committee yeah that would inform Donlin, so I'd have to find {inaudible} I can pull it up here

HK: You know the one that would probably have the most influence upon that whole thing is the Kuskokwim watershed.

JE: Oh really

HK: But it went bankrupt you know.

JE: Oh yeah.

HK: Don't have any money.

JE: The one Dave Cannon was with them.

HK: But it's based on the base studies of what's here now, see all the ecosystem that's happening and the water control and they would have a big big portion of what would have happened with the watershed. The water discharge that came out of that project. And the funding.

JE: Darn.

HK: So if you really wanted help with something this whole country, keep the watershed finding.

JE: Okay.

HK: Help them, that's the baseline and if the mine makes any changes they'll be the first ones to know.

JE: Okay well thanks for sharing.

HK: As far as fishing goes from when I was a kid to what it is now way different {laughter} it's way different. You know there was a lot of fish, a lot of lot of king salmon, there was a lot. I mean you could walk across the river on them. They bought them by that thousand and thousand and thousands of them. Five bucks a piece.

JE: Wow, and how they just don't see that many?

HK: I saw a document one time, Alyssa gave me a document one time that had Congress when Don Young he negotiated purchasing smaller fish out of this river. And it was with the Japanese and they, he made a deal with them that they fished off the Kuskokwim Bay offshore for fifty thousand king salmon. Maybe it was five thousand king salmon, some low number. And in return they got to fish down there in return they would come and buy fish along the Kusko. Kuskokwim put a processing plant in. And they came in and bought fish along with Campolucy and Bruce Crow and that was the 70's and 80's and holy cow they cleaned out and then they went down there at the Kuskokwim and checked those guys, they had half a million king salmon on board.

JE: They were only supposed to get five thousand?

HK: Some low number, maybe it was fifty thousand.

JE: Who was that?

HK: Some Japanese outfit I forget the name.

JE: Oh my gosh.

HK: Yeah huge number.

JE: And they caught them? What did they do with the fish?

HK: I don't know, they probably kept them and just didn't allow them to fish anymore.

JE: Oh my gosh.

HK: Yeah.

JE: Terrible, so they probably contributed also to the decline?

HK: Probably started right there you know that.

JE: Taking all those fish.

HK: You see that they call it decadal oscillation in the numbers I don't know if it's from decadal oscillation or maybe could’ve been from that number from that could've been the start of that. For the server i'm not sure. Alyssa still might have that document.

JE: And that's Don Young and who Japanese outfit?

HK: Japanese fish buyers on the Kuskokwim

JE: Okay cool great job, thanks so much Henry.

HK: But yeah a lot different there was a lot a lot of people fishing 800, 900 people fishing all loaded up but just piles of king salmon five bucks a piece.

JE: {laughter} WOW, different days

HK: Yup then when it was unmanaged you know nobody heard of any depth rest- restrictions and there was no restriction nobody knew of any net restrictions and we caught only using 8” but the depth restriction was actually 35 mesh but nobody heard of it and a lot of people fished 45 and I was one of those that ignorantly broke the law. You'd go out there to get sixty kings a drift

JE: Wow. One drift? When was that?

HK: early 90’s

JE: Early 90’s and you were using 8” 45 mesh? Now you can't use 45 mesh or no

HK: No its always been 35 mesh deep.

JE: 35

HK: Ya,

JE: You were using 45,

HK: Yup,

JE: Did they stop 8” that wasn't that long ago was it?

HK: That was right about that time 2013

JE: Shoot okay,

HK: and they went to 6”

JE: Okay.

HK: In the hopes of saving the large king salmon and that was Beveraly Hoffman did a big push on that

JE: Oh really okay.

HK: Cause they were seeing a decline in the large 40 50 pound kings salmon.

JE: Wow.

HK: and yeah these fish we catch now with the 8” they went through you'd see a little tug and they'd go though

JE: Really/

HK: That's the fish we are fishing now and there and there there like a jack salmon they have a different flavor to them.

JE: The fish you are catching now?

HK: yeah, they taste a little off.

JE: Really?

HK: Yeah

JE: You’re seeing more and more jacks.

HK: Part of me?

JE: More and more jacks?

HK: Yeah, we consider these, we consider these fish to be smaller king salmon, jack salmon.

JE: Gotcha.

HK: All the fish the king salmon were talking about are 30-35 pounders a king.

JE: Back then

HK: Normal king, yeah.

JE: How much are they now?

HK: You're lucky if you get a you know in these 6 inch you get a 25-27 pound king your king really good. And that was the jack salmon that went through the 8 inch net.

JE: Thanks,

HK: There was, and you know, they were all this big sixty or eighty of those in your boat you got some finish {Laughter}.

JE: Do you have any pictures of those?

HK: I might.

JE: If you have any let us know.

HK: Yup

JE: Be cool to see for our project, look at them compared to the ones we see now.

HK: Huge, they were all huge.

JE: Wow

HK: Some of them with the 8 inch gear some of them wouldn't even get caught they would just bounce off. And those are you know 55, 50-60 pounders you know. Once in a while you get a 55 pounder you pull them in you know they are keep on coming you know, and then great big bellies on them you know. They are pretty awesome to see. But truly I believe that that 35 pounder is still out there, lots of them. Go drop a 7 ½, 45 mesh 7 ½

in there and you will get a bunch of fish. And they typically swim 16 feet and deeper and that's where we stop fishing right at about what is it about 14-16 feet with a 6 inch?

JE: Okay.

HK: Somewhere in there, you know maybe maybe 18 you know were right at the top end of where they like to swim. But I believe they are out there, you know I don't know what the sonar test fish are catching but they fish at 45 mesh but I believe it's 8 ½ inch which is too big for this river.

JE: Too big?

HK: Yeah so it might use a i think they do, maybe they do you a 7 ½ 45 I don't know what their numbers are.

JE: I think they for sure use 8 or 8 ½ I don't remember.

HK: Yeah

JE: When I was there they had a bunch of like four or five different kinds.

HK: But they fish at a bum spot. It's kind of shallow, and well all they want is a snapshot. They don't want to go where the major fish portions are you know but they just want a snapshot of what's there. And if, and those, those king salmon, those big king salmon if they have their way they'll swim in that deeper water. And stay away from where those guys are fishing.

JE: The sonar wouldn't capture them then.

HK: Right.

JE: Okay.

DL: Thank you for sharing and taking some time to be with us.

JE: Thanks so much Henry.

INTERVIEW PARTICIPANT: EVON WASKA

Interview Date: 4/26/21
Interviewer: Janessa Esquible, Danielle Lowrey
Location: Virtual
Home community: Bethel

Evon Waska Sr. [EW]
Danielle Lowrey [DL]
Janessa Esquible [JE]

JE: Okay, looks like it's good. Uh-huh and do you want to introduce yourself first?

DL: Yeah, I am Danielle Angilan Lowrey. My parents are Jeff and Sophie Lowrey, and I was born and raised here in Bethel. I went to school in Juneau. I am the fisheries Biologist with ONC, and I started in January but I've worked with ONC for about three seasons now as the fish technician.

JE: And I am Janessa. I am originally from Detroit, the great lakes region. And I am with the [inaudible] band so a first nations tribe in Canada and I am Ojibwin, my fathers side they come from Mexico. And I moved to Alaska in 2014 and I've been in Bethel for over five years and I've been working here through the Natural Resources department. So I'm really grateful to be here and quyana for joining us. You want to introduce yourself too.

EW: Evon Waska Sr. I lived here all my life and I continue to do so. Living here.

JE: Awesome, Quyana. Can you

EW: I'm from Bethel. Mamterilleq.

JE: Yeah, okay awesome we are grateful that you are here. And Evon Waska Mamterilleq. And Evon, what is your age?

EW: 62

JE: 62 from Bethel and you are Yup'ik?

EW: Yup

JE: Okay so, Evon Waska, what was the day again Danielle?

DL: The 26th

EW: Okay.

JE: And you can feel free to share anything outside of what we ask, and we will just kind of take turns taking notes and if you are ready to get started we can start. Okay, so the

first question is just tell us a bit about your background, where you are from, how you grew up, and what are your connections to salmon.

EW: I was born in Anchorage but I have lived here in Bethel all my life. And I was born at the time where we're still using dog teams and in a one room house, we were using wood stoves back then there was a lot of snow. But uh-huh growing up I can still recall dinner was only fried king salmon and it was placed in the box in the middle of the floor by my mother in our winter time. It was part of our diet everyday, diet. Maybe all through the 365 days a year but I remember dried king salmon being our only dinner at that time. Morning through lunch all too even breakfast. Even those dried king salmon eggs were for keeping us warm in the winter time, right my mom would tell us to eat those foods. And I can still recall when we first got a freezer my dad, before they knew about freeze wrap and the ziplock freezer bags my dad would put the whole king salmon in the freezer. I don't know how many ways to get us through the winter months. I still recall seeing him do that but anyway every spring and summer we would harvest the Smelts came first and we would dry those up along with my sisters everybody would be, the whole family would be helping out. I had three sisters and two more brothers, but the Smelts came first and then the sheefish we smoked and dried and then the king salmon came. And my dad had a two story smokehouse and we'd make sure we filled up the top and the top part with king salmon, slabs, dried king salmon, and one quarter of the, quarter of the smokehouse with more dried king and the rest would be chum, reds, and silvers all dried.

JE: Hmmm yum. {laugher}

EW: And uhh when mom and dad said there was enough then then we would quit fishing. Wait for the chums and red salmon and the last would be Silver salmon.

JE: Okay

EW: And we would have uhh ice nets all through the winter, like my dad, when the ice would be about three-four inches we'd go out and set river four nets. And the best one we would leave in all winter. I still practice today. I took my net out though, I have a net every winter.

JE: You set your net out there?

EW: Yeah

JE: And

EW: But I took it out

JE: Ohh okay

EW: To the condition, springtime

JE: Okay, you get whitefish, pike.

EW: Yeah whitefish, burbot, pike, sheefish, and there's uhh I believe there's four kinds of whitefish, the ones with big eyes. I don't know what they call them but we call them pointy, pointy nose Whitefish. And there's the block heads and there's the ocean Whitefish.

JE: Ohh

EW: The ocean whitefish we will get in February, but one thing I noticed this, when I first started setting the net underneath the ice that we are catching there was an abundance of pike like predator fish and I hadn't caught that many before when I first set the net out. We usually sell them, catch them, we usually go hooking down Johnson River and catch those. We are catching them in the nets.

JE: Hmm

EW: Biggest ones were maybe about four feet. We don't hardly get those sizes but how big we are catching four feet and pike.

JE: Really

EW: Northern pike

JE: So when you were younger you were not catching as many?

EW: No we would go Johnson River and hook them, we would hook them at this time and dry them up.

JE: Uh-huh

EW: But one thing I notice now that uhh ice nets that we set in November when the ice the river froze up were catching more pike now.

JE: Hmm okay

EW: And I have a net right above we call it Joe Petes. We {inaudible} pike well already catch and then I wonder if they are eating up the salmon Smolts

JE: Huh, and you’ve been fishing in the same place ever since you’ve been young?

EW: Yup

JE: Go to the same place.

EW: And we don't usually catch the monster pike; they are usually up the river like the Holitna. The Holitna River is catching that size.

JE: Really. So they are a lot bigger?

EW: Yup. And there's an abundance of them, lower pike.

JE: Okay

DL: So next question, could you tell us about salmon fishing when you were young and how is it different than today?

EW: Salmon we had boats uhh wooden boats then and our, and our outboard weren't too modern there was hardly any reverse. We had to oar, the person that was oaring would oar, and the person in the back by the engine would set the net out when we were drifting. Then we would oar and drift and that's the way we drifted with eight and a half, eight and a quarter king nets, white king nets. And then my dad would mostly use set nets like four of them but when we needed more we would go out and drift and make it. His main goal was to get what we call fishing drying weather. Sunshine and wind cause king salmon slabs are harder to dry, he would go hell bent on catching lots of ling salmon to try to make sure we had enough for the winter months.

JE: Uhh okay

EW: Okay then we would buy out usually catch when I had my own my family of my own. Had about three kids then my goal was about eighty kings dried, dried king salmon strips, salted when I say salted king salmon we would put them in a five gallon bucket along with rock salt what we call rock salt. We call them saloon’aq those would be more food for the raw winter months and plus the freezer but today I don't eat that much now.

JE: Okay, is there anything different about any other thing different about salmon fishing like are the fish, do they look the same, are you able to go out and fish as much as you were when you were younger. Things like that.

EW: No since these restrictions we are always too much on restrictions. By the federal and state wildlife, here as these agents. They restrict us too much and the biggest one is April 2021 and we're on restriction and the biggest thing on my mind is the Donlin Gold mine.

JE: Hmmm

EW: Because why are they going to leave that tailings poison lake, it's not going to be a pond and they predict that twenty-thirty year mine life and they're going to leave it up there.

JE: Uhh

EW: And I highly worry about that because we still collect rainwater in our containers overflow and this tailings lake it wouldn't have no drainage. It's going to be man made and it's just going to sit there. I know it will overflow and if it does break everything will be gone here on the Kuskokwim I believe.

JE: Hmm

EW: I asked people older than me and they agree with me when I ask them uhh how you think, what if that dam breaks would there be any more fish? They would say no.

JE: Ohh

EW: And that's my biggest worry right there and what makes me mad is this State and Federal Fish and Game they don't even bring that up uhh State does but the Federal Fish and Game there highly focused on keeping us restricted they don't even bring that thought of it the gold mine. And {inaudible} I heard once that the manager, maybe the head Fish and Game he literally gave Donlin Gold to go ahead to mine I, I remember that. And me and my people we struggled were on restrictions for fifteen years and we are still struggling to put enough dry fish for the long winter months and when they, what makes me more mad they let us fish in increments in the rainy weather. We are always trying to beat that we are usually done by July fourth. king salmon, chums and reds. and we hardly dry silver salmon, it's mostly for the freezer waiting for my family to freeze them, silver salmon. Now they make that, they make it harder to make dry fish and otherwise they call us into hardship and that was not, that's not part of their I read in that one manual that they are not supposed to be doing that. That federal fish and game guy guide book, they are supposed to be helping us people.

JE: I’ve heard you say at meetings which I really appreciate and respect that you listen to your elders and you know what they have to say about fishing, and I was wondering if this next question kind of ties in with that is. How did you learn to care for salmon when you were growing up, you know not thinking about Fish and Game or Fish and Wildlife but just what did your parents or your grandparents or your elders teach you on how to take care of salmon and be a god (steward).

EW: The biggest one where we dried king salmon that was our main source of food, it wasn't chum salmon it was dried king salmon. Throughout the winter my dad and mom would, we all would be eating it and it would be making us full. It was our, our diest everyday by knowing that growing up its already part of your culture and self identity of who you are and I feel that is being lost due to the restriction that we are having and I that complain more than once that its affecting our young people too back then first king salmon we caught, my mom would cut it up in pieces and I would be the one that would hand to the elders, the elderly people and families around, there was a lot of sharing and that one fourth four or five feet king salmon it we wouldn't have it all we shared it with that first one. Or the first ones anyway through set netting. But now there are too many restrictions. Through all this hardship, hardship we are stressed more what I mean by

stress were the thought of not having enough for the winter, long winter months. Before our children were growing up when my dad was alive he would tell me, “Hey remember your kids stomach grows every year.” And just by him saying that I used to, we would go on through generation after generation to make sure we have enough for your growing family and for the long winter months coming. When the moms would teach their girls how to cut fish and take care of the fish, there was no wasting.

JE: And with hmm you mentioned sharing, no wasting, they taught the younger generations how to take care of the fish. Is that still being practiced today? Or you think people are still doing that even with the restrictions.

EW: Yup, yes.

JE: Good

EW: It’s part of our culture and self identity like I told you. It's our main source of food. They have their cattle and cattle a part of their farms while they are harassing us and restricting us and harvesting our food. And that's why our people used to be nomadic but our moms and dads choose this education. They settled down but the one on making sure the village was close by the river so they know every spring and summer the salmon are returning. They would go to fish camp for chinook, I set up mine by my house like my dad used to. We still make fish camps.

JE: One last question before Danielle asks you this, you said you’ve been fishing in the same place since you’ve been young are people, do people respect your traditional fishing areas where you set your net?

EW: Yup, now we have to go to tributaries when we are closed and I always fear for, even myself and my other people that we go down the river were the river below Napakiak starting from Johnson River the river gets wide and when it gets windy and stormy that's a dangerous place to be in. starting from the Johnson those tributaries (Non Spawning) Johnson River, Kialik River, and Pailiq River that's above Eek Island and used to be Quinagak but those are dangerous places when we have a little boat. It's harder to get fish with those restrictions.

DL: The next question is how did your ancestors care for salmon?

EW: We set nets first and we get our setters ready like before breakup and we would do necessary mending and our dad would teach us how to mend and get the set nets ready and then we would learn how eddies were through our dads and where to set them and we also got our drift king nets ready. We are always preparing for the next food source so they say that. But then we {inaudible} were raised.

JE: And today do you think that your Yup’ik values and your knowledge do you see reflected in management? You think management is considering that when they are making decisions.

EW: Yeah they are to me it's discriminatory to, Anchorage people are commercial fishing for king salmon and I see them giving them away to restaurants I see it on Anchorage Daily News channel 2 channel 5. Me, me and my people here on the Kuskokwim and Yukon River we are restricted and we have to beg for one. And that's not right to me, I think we are the only restricted rivers here in the whole of Alaska.

JE: Wow, okay.

DL: What concerns do you have about our salmon fisheries today.

JE: You mentioned Donlin so we have that one down here.

EW: Well back then we used to go moose hunting to the Holitna and the HoHo Rivers that were upriver and this elder Nickson Bill {Phone Rings} they used to warn us. I don't think the moose population can continue to support this status quo. And I believed it even I, I have five grandchildren. I believe those female king salmon are very important for going up the river and spawning and for it to go back to eight, eight inch and king fishing right now we are using half a net and six inch up from the Johnson River. Down below that we used a full fifty fathoms for it to go back to king fishing using the full fifty fathoms. I don't think the king salmon could support the status quo. Why our populations are growing here in the, not only in Bethel but in the surrounding villages I heard the elder James Charles mention that more than once and I am starting to believe that is true. But I'm okay now, six inches and half a half a net just to get enough to get by and supplement the rest with chums and reds and silver salmon. But I still long for king salmon. {Laughter} Man, they should give us one year without restrictions for all these fifteen years we have been on restrictions.

JE: Fifteen years. I'm sorry that is a long time. Is there anything you've talked about, you talked about concerns with fisheries management. You're concerned about the restrictions, are there other things you're concerned about regarding the fisheries managers, are there things you know with who…

EW: Awe

JE: Who they are, what they do

EW: they had one last year they opened up upriver twenty four hours twenty-four seven that's not right. They are going to do that. They should let us have an opportunity to fish too on the Lower Kuskokwim even on the Yukon.

JE: Hmm-hm

EW: And I call that miss management by Fish and Game and that's not right and we had a two week closure and that was not right for us to be closed it was called to under heart for me and my people.

JE: Is there anything that you think that's working with Fisheries Management anything positive about Fisheries Management or not really?

EW: {Inaudible} I've been here along with my people out on the river and I notice the big ones are not getting caught, some of them.

JE: Hmmm

EW: And I haven't {Laughter} caught a big female egg baring one in a six inch net {inaudible} that makes me happy that means they are going to places and spawn.

JE: Okay so you like the six inch is good then.

EW: Ya

JE: I see

EW: But at least they should maybe let us set nets for, give us, ten big kings or five big king quotas. We deserve that through our king steaks and big king salmon. Man that one big king salmon that four foot and five footers man that goes a long way.

JE: Wow

EW: That really takes the stress out of having food for the long winter months.

JE: Ya, so on the quota do you have recommendations for what managers need to do better? They need to have less restrictions. What other things do you think they should do?

EW: Less restrictions and more fishing opportunities and like to set the biggies to fish drying weather for the chum and red salmons. Those are, we need to have fish drying weather and the folks in, too much on me and my people and I find that discriminatory. Due to the fact why the only ones here in Alaska that are restricted. And what makes me mad there's commercial fishing in Anchorage and giving them to restaurants and now they are talking about low chum runs, back then we used to have advocates and that was for the {inaudible} and they would go to Anchorage and tell us about I heard them say it more than once, you guys need to advocate and let more chums and reds pass for the ones that are bound for the Kuskokwim and Yukon Rivers. There was more than one advocate, he'd always go to Anchorage and advocate for us on that and now they have these fish trolls and I remember that bycatch of fifty thousand kings they dump them in the sea.

JE: That's devastating. So we need to have more advocates for these areas, the trollers and protecting our fish out there.

EW: There doing no more advocates

JE: Wow

DL: So next question: what does indigenising our ways of knowing about and managing salmon look like for you?

EW: Back then the main purpose was getting them for the long winter months like I told you when they first started coming in the main folks was getting them and letting the fish. And the moms and the girls make dry fish and when mom and dad said there was enough, that's when we had enough for those species and we wait for the next salmon to come. And that is still the way I do it.

JE: So being able to put enough up enough for your families and listening to the women on how much they need

EW: Yeah

JE: When to stop fishing? Okay and what do you want to see for salmon and your people in this area in the next forty years?

EW: See just like I told you. I don't know how their focus is on me and my people. They should be focusing on the Donlin Gold mine and that big poison lake they are going to leave behind that's powerful enough to kill everything here on the Kuskokwim River anyway. But they focus on the natives here on the Kuskokwim and they do moralize us and that's not right. I believe they should give us the freedom to fish, why do I say that when those, then Junes are gone and then we open up again for fish you'll notice there's hardly any, other wise people fish when they want to go fishing. And what used to be about king fishing, those people that got done early, a lot of room on the river, no combat fishing, what we call combat fishing everybody is out on the river and there's hardly it's like commercial fishing. Back then it was open twenty four hours twenty-four-seven. Those families that needed it would go and they would fish on their own time as long as they are in the fish drying weather. And there was a lot of room then.

JE: And you've been calling in to the meetings a long time being an advocate for your people and the fish, are managers listening to you?

EW: No I believe they are not because I heard on the radio and all the same they, they predict no fish runs and already me and my native people were on restriction automatic here on the Kuskokwim and Yukon Rivers, that's not right and they predict low runs on king salmon and were to blame low runs on the chum salmon were to blame. We all know the fish and the salmon come from the ocean. I believe they should look on in the ocean instead of harassing and giving us a hard time giving us a hard time for putting up food for the winter. This ain't the first time this happened on the, I read on a book in a book once but I forgot who the author was on Yukon fishing, subsistence fishing look back then and they were Yukon people were not catching king salmon and then there was a catholic

priest he heard that they were in {inaudible} king salmon on the mouth on the mouth of the kusko- Yukon Rivers that were bound to the Yukon Rivers and that catholic priest helped them Yukon Natives on that one. But I try to recall who the author was. And I believe that today there is bycatch in every section being done at sea. We all know that the salmon come from the sea. And that's not right to {inaudible}.

JE: Is there anything else that you want to share with us before we close out.

EW: I want to say give us that freedom to fish again, we live in the United State we dont tell them how to farm and do what they want with their beef and cattle why they harassing us, why are we fighting for subsistence like I heard one of my people saying that, “Why are we fighting for subsistence?” That's our right to do that, to get food like for millennia from generation to generation that's cultural and self identity. I believe we should be giving our freedom to be doing what we have always been doing.

JE: Quyana for sharing, one of the other last things I wanted to ask is, you know these forecasts: the Fish and Game forecast or whatever they say the numbers are low or it's not looking good. What do you and your elders and ancestors know and understand like the salmon runs? Do you look at stuff like the wind or other things?

EW: Back then there was a lot of snow and growing up winter was winter and we didn't worry about that, mom and dad didn't worry about that, all the natives didn't worry about that we all knew the salmon was coming every year like they always do. These two half breeders overfished one day with their fifty fathoms and that's what started all of these restrictions but if you let me fish with a fifty fathom net when they are here, there'd still be kings around. Just enough I don't know all we need is eighty kings to support my family but I don't believe in these predictions what the Fish and Game and State, State and Federal Fish and Game making prediction already causing stress to me and my people for already they put the word out they are going to put restrictions on us. That's not right, you hear my people we don't make predictions we always know they are coming.

JE: Is it disrespectful then to make predictions about the fish? Do you think? For them to come out with those numbers like that.

EW: Yeah there, one thing the elders know, they know by the. They just know by the man they just know that every year they know they're coming back. That's all I could say there's enough for everybody but State and Federal Fish and Game they just make it harder to get it and that's not right.

JE: Yeah I don't have anything else quyanaq-vaa this was really, really powerful sharing thanks for sharing. Thanks for sharing all this with us.

EW: Quyana.

INTERVIEW PARTICIPANT: THEODORE ‘TAD’ LINDLEY, ELIZABETH ‘MIK’AQ’ LINDLEY, ELIJAH ‘PINCHBOY’ LINDLEY
Interview Date: 2/15/21
Interviewer: Janessa Esquible & Daniel Lowrey
Location: Virtual
Home community: Bethel
Transcribed by Avery Hoffman

Notes from interview [recording started late]:

Janessa Esquible [JE]: Please tell us a bit about your background – where are you from, how did you grow up, what connections do you have to salmon?

Tad Lindley [TL]: Tad Lindley, when I grew up, we thought we were doing pretty big if we had canned pink salmon and hardly ever had it because it was considered a luxury. When I was living in Fairbanks, we worked in Cordova and red salmon became big part of our diet. In Bethel, my diet transitioned more to King salmon because I married my wife who was from an active fishing family. Father-in-law was active member of the Kusko Fish Co-op. He had a Kuskokwim commercial permit I also have a Kuskokwim permit and have fished in Bristol Bay as well.

Recording of audio starts

TL: Process of selling my Bristol Bay permit right now but I still have my Kusko permit.

Elizabeth Mik’aq Lindley [ML]: I can go. Ummm, I grew up in Bethel and grew up fishing with my dad and going to fish camp. I have a really strong connections with salmon. Um, you don’t really think about it until you leave like, you can't just go out and go fishing whenever you want, but yeah I grew up fishing with my dad. I grew up on king salmon. I don't even really like chums and reds {chuckles}. Yeah we were one of the primary providers for my mom’s side of the family. We also commercial fished when me and my brother were little.

TL: Trying figure Pinchboy’s computer out but it looks like it’s not working.

Elijah Lindley [EL]: Okay. So, I’m Elijah I’m from Bethel. Grew up here my whole life. And I think I grew up around a heavy subsistence lifestyle. I grew up going to fish camp and fishing. Every year, every summer that I can remember. And what connections do I have to salmon? Um, I think I have a lot of connections but like my sister said it’s kind of hard to think about how to describe. What connections you have. I would go commercial fishing a lot. Um, yeah. I’m just. I don’t know how to say how I feel connected. I feel really connected to river and the salmon that we catch.

ML: Yeah, that's a weird. It's a hard question to address. I'm like.. I'm trying to answer this like. Find a way to form this question of connections to salmon and my own research and it's like really hard to think about.. And I don't think my apa ever thought about his

connection to salmon it just was, it's like Yup’ik peoples relationship with subsistence and the land it’s so relational and like you know you don't want to think about it until those relationships fade (inaudible) or have to transform and yeah it’s so much a part of you that. Sigh. that its that its hard to say it in words like what this connection is. And for a long time like, talking about when we couldn’t fish, like I would just start crying {little chuckle}. I don’t want to cry. {whisper} But umm yeah. I think like talking about it at school when I was an undergrad like that was hard to talk about it so. Truly deep connections where we are.

Danielle Lowrey [DL]: Thank you all for sharing.

JE: Yeah thanks so much.

TL: I think with my father-in-law, after he got dementia… He still always talked about fish {starts crying} Like he couldn't, wouldn’t even go to the window to look what time of year it was but there would be snow on the ground when he would be asking me if the reds were hitting. It was like, one of the things that he never lost touch with in his mind.

JE: Thank you so much Tad. Would offer you a tissue if we were in person.

TL: Thanks.

JE: Really powerful sharing. But thank you so much for all of you. One of the next questions that we have on here is, if you could tell us about salmon fishing when you were young and how that might be different today.

TL: When I first started out here we cut and we ate more fish than we do now. Probably because we had less money. So, we ate less store food but yeah, we'd eat fish, I’d have fish for breakfast lots of times and umm a lot of days I’d have fish twice and then we'd have not just salmon but always had like, there’d sometimes there’d be five species like pike, dried pike, dried smelt, kiarneq (excess meat – interior muscle), umm chums, dried kings. I don't eat dried reds. I think I ate too many dried reds when I was younger and I got sick. Umm I don't mind them baked or fried but I can't eat them dried but we probably ate more when my father-in-law was still robust. We had umm probably four households out of our fish camp so, we had umm a set quota, we’d usually put up like 80-100 kings for slabs, and then umm not to be critical of other families and stuff but I see people doing strips first but I don’t think that’s the old way of doing it, cause umm we'd always do slabs first and then we'd do umm strips after the slabs were ready to go in the smoke house because there wasn't enough room. So, we'd umm had always want us to cut about 50 or the ladies to cut about 50 kings for strips and then when strips were done, we’d umm fish for small fish for chums and reds and umm dry those after that so we’d put up way more cause there was, there was us and him, his house hold in Nunap and Toms house here and umm Margaret and umm Caroline lived here part of the time and those two are my wifes sisters. So in those times umm Larry had his own fish camp so lot, most of the time I was the only time who wasn't working so I got to fish umm until we were done, like every day. And sometimes if it was a slow year, I would fish every tide like twice a day

umm but so there was, there was no thought of restriction at all cause salmon management work and board was for it was for, commercial fishing they didn't have anything to do with subsistence. And umm course now we, it’s umm quite a bit different I guess were probably cause of having too umm fish at Johnson River it causes us to work harder for fewer fish. Umm but then we don't, we don't need to put up as much fish as we used to. Cause Tom and Minnie do their fish at Napakiak now and my Father-in-law is gone so that's umm the biggest change like having probably having the schedule dictated by who, umm we may get into it later I don't know I don't have a super high {chuckle} opinion of umm what's going on right now. I don't know did I answer the question? Yeah, and you'd see it was umm people would, you could tell people would just fish until they were done like there'd be, you could tell by the number of boats on the river. There’d still be fish, plenty of fish coming but people would be done so the pressure would, would drop off significantly. And we set netted too, my father-in-aw set netted umm I don’t like set netted fish cause sometimes they would come up dead. Unless I could sit on the net and watch it so but he always set netted early and then when there were running strong enough to drift then we would start drifting. But what he told me was when he was young, they didn't really target kings because their gear wasn't durable enough to target kings like we do now. So, and then some of those years we've eaten more chums which I really like dried chums umm but some of those early restricted years before we umm when we still maybe had more people to feed then we ate more chums. But chums are really good dried.

ML: I think what I remember most being different is just I don't know when I started fishing. I was probably like five when I started going fishing with atti and reversing the boat, I remember that was my job when we were fishing. I guess that the biggest difference is just the con.. I feel like it used to be more continuum of fishing like we would just fish throughout the summer and then, and then all the restrictions happened and, and then you just can't go out and fish and learn the river anymore. Like whether you have fish for the winter on depends on whether your schedule lines up with the management and to reschedule when they decide people can fish. Like a couple of years ago I had to fly home and fish for my family, dad was going to be out of town and I don't know where my brother was, I think he might have been working but I had to go home and I was kind of illegal because I wasn't technically a federally qualified subsistence user but umm and it was a federal opener. So, I went home and like drove all the way down to Johnson and umm got our ten fish and then came back to Anchorage afterwards and umm I am worried about my little cousins because like they can't just go out and fish. Like they’re never going to know what it's like, what it was like or like learn the river. Those were my notable changes for when I was young.

EL: Umm. I guess when I was young I kind of saw going out and fishing is like a chore and I would be like “umm” when my dad would let us know were going out fishing tomorrow and I would be kind of like can I just stay home you know and just like play out with my friends instead of going fishing. And then like whenever I would go out whenever we'd be out on the boat I kind of just like I'd want to do the least possible you know I’d like, when we are waiting and whole the net is out, I’d probably wanted to just like to just like lay back and take a nap until like we could go back. But now I guess today I kind of

see it as more like an opportunity to like get food and stuff like that, you know since a lot of it is restricted now umm you kind of get a little excited when you're going out. Umm I didn't get a chance to fish a lot last summer but when I did you know I would get excited ya know like “ohh yeah we are going out fishing” it's a good chance to get some food I guess that’s how its different today than it was when I was younger.

TL: And I think umm the girl [EL] had on umm that like we are not like learning anymore I mean I’m learning how to fish Johnson River but the opportunity is so limited that we really can't afford to experiment or try, I’d probably try, there's a new spot I want to try but umm I have to be way more precise about exploiting. Very limited opportunity in terms of where we fish and really have to be on top of the tide which is kind of an odd thing because when you’re not fishing every day you’re less on top of it, so fortunately the places that we used to fish are still productive that I want to think about the future I think about if the river shifts it will be difficult in our limited amount of opportunities to really learn where to fish unless my ideas are transferable I don't know if when I do well its cause I am smart about it but maybe not, maybe I am not. I guess I’ll find out if my ideas are transferable when the river changes.

JE: Thanks, umm I had a couple follow up questions based on your sharing. Do you think the recent restrictions are causing umm not being able to pass on that intergenerational knowledge of how to fish and where to fish and when to go out and stuff with the younger folks?

TL: Yeah, cause there's families that are, that are missing seasons if their or missing up you know significant parts of a season of their boat is down because they can't borrow a family members boat because family members are using it on the limited times. I think it's really hurtful to the passing on of traditions. I mean seems like there's two things going on. There's the fact that CVRF pulled out which they employed a lot of people fishing and a lot of people learned to fish that way as a helper on somebody's boat coupled with their restrictions for subsistence fishing so you’re not fishing every day and somebody stays home or misses an opener they’re missing what in the past would have just been just another day but it might be I don't know. We got all our fish in three openers I think cause, because Johnson River was wasn't productive this year. So, the kids each fished one opener and then front two so I must’ve fished four cause yeah we fished one with a friend of mine and all those fish, half of them we gave to his wife's family and the rest of them we gave away to people who couldn’t fish. So probably these young kids are there not learning how to hang gear or mend gear. So yeah, when Pinch boy was telling the truth when he was little, he didn't like fishing. But when we would go to sell fish and there were other boats around, he would work really hard {laughs} when he had an audience. {laugh} If you see my kids’ fish if you had that opportunity they are really well, a well oil machine they know exactly what to do and umm and that’s just because of a lot of repeated exposure to it.

ML: I think just one more thing on that because so much of what you know is that, that constant context with the with the constant exposure like going out on the river multiple times a year and I mean this part of the lower river is really dynamic so if there's not

continuous learning of changes knowledge can't be passed down and then be applied to different contexts.

JE: Thanks Mik, Can I add an odd question I have one other question for you before I move on to number three. (Of federally qualified users do you think that in recent years with the water we are living in umm being restricted to federally qualified users has posed any challenges for you when you are trying to come back and go fishing with your family.)

ML: It should I haven't really let it though. When it's a federal fishery I can't even touch the net but I've gone out every year, I think. I think there was one year that I didn't go out but nobody caught me. I do think that those qualifications could be problematic.

DL: Okay, well next question umm how did you learn to manage or care for salmon when you were growing up?

TL: Well, I got a degree in fisheries form UAF but I think as far as my perspective on management the fisheries program was pretty weak on salmon management so I learned that from a biologist named Sam Sharr at the Cordova office. As far as caring for salmon my father-in-law really went to great effort to explain to me and teach me how to, how to do everything umm {Emotional} he said the day would come when he wouldn't be there. So I got a lot of good instruction from him I didn't realize it at the time but I did after he got sick and couldn't go to fish camp he'd been preparing us. He did a good job, some of the things I do fishing I guess I bleed and slush my fish too. So, some of the things I like they never did that but umm I learned that from commercial fishing, I guess. So, but yeah when you, I'm pretty proud of how we take care of our fish. And of course my wife's not here right now but her slabs are better than anybody's I've ever had. The Indians strips on the Yukon there umm Athabaskan they're better than ours but my wife slabs are they're the best. No offence to your mom Dow. {laugh}

ML: I also have a degree in fisheries. Twenty years later they didn't really improve on salmon management I don't think. They have one management class that (inaudible) and umm there isn't a lot about Alaskan fisheries or how to manage salmon but I guess I learn through atti who learned from apa. I don't think I can speak to management yet, maybe someday. I need more experience but I think we've always cared for salmon really well I've recently like when you leave and really value things like be able to fish and do subsistence activities and then get exposed to like the idea of being Indigenous and like you know how our way of life is not normal. Put into words how we come Indigenous people generally you fish and things they harvest umm so I was telling atti this summer about how fish have agency and the fish we caught gives themselves to us. {laugh} Because we caught a lot of fish that were like napping. So that was just how I, how I thought about those napping fish giving themselves to us in our net.

EL: How I learned how to manage our care for fish, I did, I didn't really help with the cutting up or the hanging or anything like that. But one thing I always remember like, to do I guess or one thing we always did was help my dad when he would bleed it and store it in ice after we catch it and I don't know how that helps that. I guess it helps with the cutting up

with it after but that’s kind of one thing that I mainly remember and I guess I’d pay attention to my aunts and when they could kind of like store, make heads, and like store underground and that whole process but I wouldn’t really pay attention to, to close attention cause I don’t care for stink heads. Umm But other than that there’s not, I can’t it’s hard to explain or remember everything that I know and how to care for salmon.

TL: Yeah, there is a lot of tedious things like, because I being a teacher after I quit working in Bristol Bay in the summer time I was at fish camp all the time so there's a lot of tedious things that I didn't really care for like moving the fish into the smoke house and stuff. But I’m glad I learned it I’m glad I was there and not at a job or something so I could learn that. There's so many little things like, that maybe people don’t think about that are really critical to the operation like when to put in the smoke house and even I don’t know where your fish camp is Dow. But some people have really gross fish camps like their location so I think they {laugh} where's your fish camp? {laughing}

DL: It is umm on the Kuskokwak

TL: {laugh} Okay yeah those are good ones up their umm some people they don’t plan properly so they don’t their set up their like fly traps. {laugh} Yeah the Kuskokwak fish camps look good. I always get a good look at them when we are bringing log rafts down.

JE: Are there any other things umm thinking about like traditional or indigenous values for caring for salmon some of the stuff that were shared in other interviews was umm providing or sharing the first catch with elders or those in need who can't go out for themselves umm taking someone else's traditional fishing location when you’re out there. Things like that…

TL: Umm, my father-in-law wasn’t real in, he wasn't strong on the old traditions I mean we always had plain boil like with the early on with the plain boil with raw onion. Umm we always our first fish we always share it out with multiple people for that fish. We always had plain boil with that first fish at fish camp. Now that we commute, we can give out, we could, were more likely to bring that first fish to Bethel. So, then we can share it out but at fish camp it wasn't, you know, everybody was getting the first fish at the same time typically. But we try to get lots of fish out to people cause we have been blessed to harvest a lot. Even with the restrictions so we try to take care of people who can't fish or older ladies or widows or whatever. Even with white fish you know with I know this about salmon but pike we send into Anchorage and we usually bring fish into family in Anchorage. Not a huge amount but the... thing that I guess one thing that strikes me like watching my wife cut fish is that she cuts reds different than chums different than kings and just thinking that that like that’s been passed down for probably three, four, five thousand years from her from her mom from her mom’s mom and umm it’s pretty awesome to think about that that she’s like carrying that on and when my mother-in-law died I never she died before I met my wife but after she died my father-in-law could do everything. Although I never I never seen him cut a king salmon cause he had my wife to cut fish for him but umm. The other thing and I guess we can’t say what gets published and what doesn’t but my wife was umm fortunate to be the best trained of her sisters and I don’t know what that is but

you watch my wife cut she is really fast so when other ladies come to help often times they do the more meany, {laugh} can I say that? They do the more menial things hopefully they don’t read the transcript. {laugh} but yeah I don’t know if I spoke to what you were asking but.

JE: Yeah

ML: My apa wasn’t really super traditional he was Christian so like we didn’t I don’t he didn’t really share a lot of like those old traditions umm with us. I think cause a lot of them are (inaudible) umm he then or whatever like Christians.

TL: He just didn’t share a lot of stuff.

ML: Hopefully relates to that.

JE: Do you all think that some teachings and rules that you shared and the last question are still being followed today?

TL: Seems like gender roles are at least at our fish camp are pretty much the same. It’s what they were when I married into it. Which I assume it was the way it always was. Sharon’s mom they had ten kids I think. So, when they fished they put up more fish when the kids were younger then, you know when I started like they put up two or three hundred small fish. Her mom did almost all the cutting. But those things I think gender roles are still pretty much intact.

ML: I’m an exception to that because I finally just learned how to do a slab this past summer {Laugh} but I know how to fish really good.

JE: So in your family Mik, did the wo- you as a woman you fish and you process the fish, and the men mostly fish.

ML: Umm, yeah I mean I, I didn’t do any processing but I did, I did more the men tasks ever since I was small. But yeah, definitely my mom and aunts would be the fish cutters and umm me an atti and sometime my- sometimes my uncles umm would be the fishers.

DL: Next Questions, umm how did your ancestors manage or care for salmon.

TL: They umm, I’m speaking for my wife obviously but umm before they had refrigeration when she was little coming from what my father-in-law told me they would umm the chums they would cut the top off of a fifty-five gallon drum and then they would put chum slabs in there, put a piece of cardboard on top and jump up and down to compress them and they would fill up drums with chums like that. I think probably most people won't do anymore because we have more access to refrigeration. But umm then I think they made more suluunaq than they do now because that doesn't require a refrigeration either. Of course, because we have freezers we can eat fresh frozen all year long which wasn't something people had in the past.

ML: I don't know a lot about how ancestors managed, stored and cared for mainly cause apa was the only like elder in my life. Umm and I guess I haven't really learned these things, but I'd like too.

EL: I'm not too aware of how they did it back then. Like traditional ways and stuff like that but if they did some of the things that we do today are similar to the way they did it back then. Then I guess I know some of it but I don't know too many traditional ways that they cared for salmon.

TL: One of the big differences is the seasonal migration because like if you go to Nunap today lots of people hang their fish at the village they have umm dry racks and puyurciviit [smoke house] like right in the village because motors are faster and so it but when my, when my wife was little, they would, they would fill the boat up with everything they needed and go to fish camp and they would fish and then when the fish were getting ready to be done the men would go up river and they would make a log raft. And they would bring the log raft down to fish camp and then they would pit everything on the log raft and they would float it down to Johnson then go up the Johnson River back to Nunap for about the time they would pick salmon berries cause they didn't have motors just were not powerful enough to commute. But that's a hardship for the people on the tundra villages now because they transitioned away from Kuskokwim fish camps and so it's that trip down to Kuskokwim from Nunap or Kasigluk is too far for, to make it worth having a setnet down there and stuff like that. So I think those people have been hurt a little bit worse by regulations than people here then Bethel have cause they have to travel further.

JE: Thanks for sharing, may you all feel that your knowledge is reflected in fisheries management today?

TL: I'd say from passing on the heritage perspective that that definitely not because the opportunity is so restricted that the as we touched on earlier that people are younger people they don't have the opportunity to learn. Like they did before the regulations. So I feel frustrated at the current state of affairs when they first went to restrictions it was, for me it was like being unemployed because I was so used to doing nothing but fishing the first half of June or like fifth through the twentieth. Like every day and that umm I'd imagine there's other probably other guys that experienced that also like. Even though I had lots to do it was depressing, I had a hard time doing other things even though I knew I had time and I should do them. Now I have gotten more acclimated to it, I guess.

EL: I would say I would think so as far as trying to as them trying to keep the numbers up for like the younger people that trying to fish. But also, I think that also kind of clashes like you just said with opportunities there's not a lot of times were like were everybody can go out and fish at the same time. Even when there are openers it kind of clashes with people working and stuff like that, so yeah.

ML: I think (we are worried?) that management is designed today, there isn't a lot of room, there isn't a lot of room to include knowledges or other values its very much scientific.

Umm {laugh} its mostly numbers and umm I'd be curious to know what it looks like, what would management look like where there's in active exchange of decision making, capturing values that people want to maintain in fisheries or just ways of making decisions.

TL: I'm really concerned about Fish and Wildlife Service managing cause their model of their career [progression at the managerial level] model results in guys who really have no knowledge of the people or the region being the man, the umm top manger there cause that's how they climb and that job is moving around the country. So, they don't know and then they have enforcement officers who don't know like their people can't answer questions like, where {laugh} can I fish here or not, and they don't know their way around the sand bars even but you think about like tides are such a critical thing. The chances are really good that it, fish and wildlife manager has no idea about tides, and certainly may have never fished a gillnet before even. So, yeah, the, the way with the current system it really umm for a lot of people it is hit or miss and it's based on tides and location. So, I feel like I'm really fortunate to have fished as hard as I did as I was younger to umm to maybe be able to predicted where I'll do good but not everybody has that knowledge.

JE: Thanks Tad for sharing about umm fish and wildlife service and I guess on that note. Do any of you have anything like specifically that you want to share about how maybe one a day do you like fish and wildlife and or Kuskokwim intertribal fish commission versus the Alaska department of fish and game may better include your values and knowledge and the management decisions they make? If not how, I was just curious.

TL: Well, I think that the Salmon Management Working Group is far more inclusive of, of umm or far more umm thoughtful about values and umm you know one of the I guess one value that we didn't touch on earlier is that they say if you are argue about our resource, it will go away. Like you see how the moose in the Holitna just came down to about nothing cause umm people up there were getting stingy about them. And I mean it was such a tremendous resource like people from the coast were going all the way up to the Holitna I remember at Kipnuk seeing Lunds coming down coming from the Holitna with moose and caribou in them and it [the moose abundance] went away. So, the umm conventional wisdom here is that it is because those people up there got fussy about it. So, I worry a lot that piece here and I, I also think that I worry about the Kuskokwim intertribal fish commission although I'm very much in favor of indigenous management. They have a vested interest in keeping us in crisis mode which kicks it over to US Fish and Wildlife service which I think is a bad, I think it's a bad move. Thats just my opinion though, so but in all fairness having worked for both the US Fish and Wildlife Service many years ago in Dillingham. When I worked for them, I felt like they, in a lot of ways were more respectful of the Yup'ik people than Fish and Game maybe. Although I think umm like Doug Beaux is an outstanding individual, I think we have been fortunate here we've had some, had some really good people. I think at least in my experience with the Cordova office in particular it seems like there was less respected for. There was more of an attitude like this is the USA I've got constitutional right to do whatever I want to do versus umm there's a people who've been living here for thousands of years. This is actually their land and

we need to respect that so I think I got off topic there. Umm but I do I worry about this whole umm KRITFC because they don't have a voice without there being a crisis I would like to see their voice being heard even when we are not in crisis mode, in other words, removing the incentive to be in crisis mode. They (KRITFC) certainly provide a stronger voice for the people way upriver where many of the fish actually spawn. And I think it's going to come back to, come back to haunt us. Because I think their mission is that people would continue in the old ways but in crisis mode its real hard for that transition of knowledge and attitudes and thought process to be transferred. Course that's me I'm outsider's perspective.

EL: We are on question seven, right?

JE: Umm yeah, we are almost there. (inaudible) do you have anything you or Mik' that you want to add? (inaudible) to your dad

ML: (inaudible) All I'm going to say is that, I'm just going to say that I agree with that. Umm I think, I think that the crisis mode strategy is doing more harm than good and I guess back to agencies managing agencies. Umm I worked for fish and game for four years so. I've seen people that are like you know, the reason why a lot of people don't like fish and game. But I think they are more accessible than Fish and Wildlife Service. Umm as far as a managing entity.

TL: Yeah, I'd rather see…

MK: In Bethel at least

TL: I'd rather see fish and game managing it exclusively with the Salmon Management Working Group [SMWG] cause that's largely Indigenous people. In over my years here I've felt like fish and game has listened to the Salmon Management Working Group a lot of the time.

ML: Yeah, I think Nick's doing a great job.

JE: Thanks .

DL: Thanks for sharing. Okay now question seven umm, what are your worries/concerns you have about our salmon fisheries today?

EL: I think they're kind of worried about a little too much on trying to save the salmon and then with and not like giving the people a chance to fish. But umm what can you do when there is not a lot of fish to fish for umm. So I guess there's like, the worries you have kind of clash with the wants that, like what you want to do you know?

TL: Thats a good quote.

ML: Yeah I think…

TL: Go ahead, small

ML: I think we have kind of touched on the concerns main concerns. I think we are on upward- upward trajectory realistically but it's just these heralds of fighting for management who gets the say so. Umm yeah.

TL: I think I worry about bycatch and all of that but that's not something that's really in the score of Kuskokwim Management it’s a bigger issue. I think that Kuskokwim, Yukon, Unalakleet River thing is ridiculous {laughter} like I don't know if you know what I am referring to but I don’t- can’t put the official name on it but what {laugh} where it’s just, I’m very much pro commercial fishing, and I love Pollock. But I also, I also think that it's hard to deny that we- were a maybe we are in this situation because of Pollock fishery. Or umm other intercepts that might be happening that we don’t even know about.

ML: Also, Pink Salmon, like communities across the Arctic they are seeing that their streams are becoming choked with pink salmon and it’s hard to differentiate between years.

TL: Mm…

ML: And if they have never seen so many pinks before. So, are they coming from? Why are there so many?

TL: Because too many people are eating banquet chicken {laughter} I think umm lot of them used to, up there used to umm probably still do hit them pretty hard but boy I had another concern. Oh the size of the kings they seem to, they seem to be getting smaller, and I know they want to put it on mesh size. Thats another thing that's changed of course too is like I started out fishing eight and a quarter. In eight inch and now I just like out of an emotional attachment I've been going and fishing my eight inch the last day of May before they shut it down for eight inch cause it’s one of my favorite nets. {laughter} but umm I was not, I see the kings have their all, their all plugged with tapeworms and I don't know if that is related to the size or not. I sure it is in some way, but I don’t remember like twenty-five years ago were they that plugged with tapeworms? I don’t know.

JE: Thanks, any other last concerns out of any of you want to share before I move on to the next question? …. Okay I think you- you all spoke already somewhat to number eight so don’t (inaudible) you don’t need to repeat yourself on any of this.. Umm the next question is what are some of the concerns you have on fisheries management/ research. Do you want to talk about some of you concerns with fisheries research you could speak to that. Umm and this question is really trying to get at, what are the strengths and what are the weaknesses of our salmon management systems. Umm today. So what’s working and what’s really not working. Or recommendations for improvements.

TL: I think sonar is definitely a strength. I think they are using the wrong kind of web there but, they are not asking for my input so I think that the web that they are using is probably

giving them less accurate apportionments. I think the weir programs are great. I mean at least we are getting a pulse; I wish the radio tagging could have gone on longer. I think that's pretty fascinating be pretty fascinating data set that could have run a few more years. Umm and I want to complement ONC also under your reign there Janessa, you've really focused on data and I data collection and umm the mathematics of it which I think is important so it wasn't like that before you got there. Not to be critical of anybody else but I think you've done an outstanding job. So yeah those are the strengths I would speak too. I kind of wish that umm somehow we could get information on the fisheries at Kailiq and Tarriyaraq or that other stop. Then there's that one where that the guys from Eek fish and Johnson River but at the same time that's touchy and people don't like to talk about it cause. If you talk about it more boats show up, I guess. And those are umm, I don't know if you guys have fished at Kialiq but it's a small, even Johnson is small. So and that's something you could, you could umm throw back in the early question like this fall I saw a guys drifting in the Gweek. Which is just crazy and like thirteen to seventeen set nets in the Gweek. All targeting salmon that's like, you never would have seen that before cause its just low, low productivity. But umm because the guys that do it, some of them do really well but.

JE: Tad, so is that you think there's more fishing pressure in the non-spawning tributaries because of the restrictions?

TL: Umm yeah, nobody fished there, like at Kialiq. People fished on the cut bank side across the Kuskokwim from the mouth of Kialiq like it just doesn't, wouldn't make sense to fish there. Umm I don't know, James Charles would have a better pulse on that. I don't think people fished on the sand bar there. We didn't we fished on the channel side. And nobody ever fished Johnson like, like, for salmon. Just cause why would you?

EL: I don't have any major concerns with the, the management and research at umm yeah.

ML: I think I shared my concerns with current management and I think any research that goes on is so, beyond the scope of regular people, and regular people activities that. It doesn't really matter I mean, my advisor asked me if I knew this research, we did a lot of salmon work out on the Kuskokwim and I said no, like. I've never heard that name before in my life. {laughter} So I think that research doesn't have this big of a role in umm in fisheries where we are. Aside from fish and game research activities I guess. Like the tagging project.

TL: I guess one thing I would like to see is umm, if I had a blank check would be umm genetics on… Genetics works so that, followed up with genetics on bycatch. So, we could have a better pulse on umm Pollock bycatch and umm like that 50 thousand kings or whatever I don't know if Janessa you ever brought up at Salmon Management Working Group they got like 50 thousand kings one season at the Shumagin Islands. And they didn't run any genetics on them. So I'd like to see baseline genetics and then have some comparative work done with bycatch.

ML: That, I'm curious about all the reds in the river.

JE: Are you concerned about the red or do you feel like that is something positive?

ML: Umm I'm not concerned about it yet, I guess I'm just curious. Like where are they coming from are they, are they Kuskokwim reds? Because that's a lot of reds that we got and then also the chums everywhere was a poor chum run this year.

TL: Yeah, I think the reds, that's pretty cool the red numbers coming on like that.

JE: Thanks, and Mik' you were saying that um the local people maybe you know they might not be as connected to research that is happening. And so, I wonder if you or anyone else has any suggestions for improvement how we can better connect local research for management or things are just fine the way they are?

ML: I guess it depends on the researcher like, I don't want a really disrespectful researcher going out to Bethel and like imposing connections with the community. But not having any respect for the people if that makes sense. But I mean if it's a good researcher who wants to do good work and they would, they would interact with the tribe at least. Umm it's something that I'm trying to priorities in developing my thesis, is like I want to, I want, if I'm going to do research on Nome's land like (inaudible) does a lot of their scientific coordination I don't know if Native Village of Nome does a lot. But umm like previously in my pursuits I talk to Native Village of Kotzebue just to like in the very initial stages like I'm kind of interested in these questions. So, I think like if, if researchers want to be involved in communities and prioritize community questions as far as their research activities, they can make themselves known to people. Umm but I guess it just depends on the researcher. I don't want to be someone who does research and like no one knows who I am like I have this paper published in ten years about fish that were taken out of Nome and like they never heard my name before.

DL: Okay, thank you. Next question, umm what does indigenizing (inaudible) salmon management look like to you?

ML: Native managers {laughter} people in management positions that understand Yup'ik world view.

EL: I couldn't say it better myself.

TL: One thing Buzzy told me was, when he was the manager, it was really hard. Cause people didn't like him in that position. But I'd agree totally…I don't think there is any other way to have a level of trust between the two entities.

JE: Okay, thanks and then the last question is, what do you want to see for salmon and people in your region in the next 40 years?

ML: People to still be fishing and to be going to fish camp. Making slabs…

TL: There is erosion of heritage these- you see in some families where they all they do is strips anymore. {laughter} Which I think I, I mean maybe that's fine and stuff but to me I worry about that when I hear about families that, they say "well we just do strips". I don't know why I should care but, I would hope that my umm great, great grandchildren are still eating slabs.

EL: I would just want to see a lot of salmon and not a lot of limited opportunities for fishing and like full racks when you drive up and down the river. {Laughter in the background} Full racks.

TL: Thats another good quote. {laughter} Blue and orange from Tunt to Kalskag {laughter}

EL: You could see the orange from a mile away.

TL: I'd like to see commercial fishing come back. I know it just crushed me when CVRF decided to put that money into education grants cause there so many guys my age. They were making twenty-thirty thousand a year and that was their new snow machine every few years and it really was such a necessary validation of themselves as men. Who had the ability to be prolific harvesters and to see that yanked away for education kind of made me sick. I mean not that I'm against education but I'm the only one ever talking about it so maybe it doesn't matter but really heard me to see that. To see that money transferred from subsidizing commercial fishery to basically preparing people to leave the region and go what to white collared jobs in Anchorage or beyond. And I know that I don't know I'm not on the board of CVRF they didn't ask me so. {Laughter} But I'd like to see numbers that would support commercial fishing again cause our fish is really good. Think we have the second-best chums in the world and second best kings in the world after the Yukon.

JE: Thanks, do you all have anything else you want to share? Before we close out.

TL: HMMM, probably think of stuff as soon as we leave the meeting but. Yeah, thanks for umm thinking of us for interviewing.

EL: Yeah

JE: You could thank Mik for volunteering {laughter} in our class last semester.

ML: Yeah (inaudible) you guys thought {laughter}

JE: Well do you guys want to get a picture? Can I do a umm screenshot and if you want to share any pictures with us for project use when you are out subsisting? This isn't like the normal setting.

TL: Mmhmm

MK: Yeah I'll send you stuff that I have and atti you could send stuff if you want.

TL: Okay I'm not good at going through pictures.

JE: Thanks okay on the count of three well smile {laughter} okay, one, two, three {laughter}

EL: Nice

INTERVIEW PARTICIPANT: DAVID DAVID, RHONDA KANUK
Interview Date: 10/19/19
Interviewer: Janessa Esquible, Destiny Ropati
Location: Kongiganak
Home community: Kongiganak

Janessa Esquible [JE]: (Janessa goes through project overview). Okay Rhonda and David. Is your home community here? {David and Rhonda nod head} Kong? Okay. And cultural affiliation Yup'ik?

Rhonda Kanuk [RK]: Mhmm.

{In the background} And Cup'ik! Sike! [laughs].

Janessa Esquible [JE]: What are your guys ages?

Rhonda Kanuk [RK]: I'm, I think I'm 54.

JE: 54?

RK: He's 33.

David David [DD]: 33. I was about to say 19. [laughs].

RK: I don't want to turn 60, 70, 80 [laughs].

JE: Okay wanna get started.

Destiny Ropati [DR]: Yeah, am I asking?

JE: Yeah.

DR: Okay so can you tell us a little bit about your background. So where you're from, how you grew up, and what connections do you guys have with salmon.

RK: With what?

DR: With salmon. Do you want to go first?

RK: You can go first.

DD: Why me? It's ladies first right? [laughs]

RK: Rhonda M. Kanuk. I'm from Kongiganak, Alaska.

DR: And where did you grow up?

RK: [laughs].

DR: [clarifies] How did you grow up?

RK: My step mom and dad they took care of me (inaudible).

DR: And what connections do you have to salmon?

RK: Well we harvest, work on fish, handle and dry them, don't want them to rot or get rotten.

DR: And can you state where you're from?

DD: I’m from Kongiganak, Alaska.

DR: And how did you grow up?

DD: I basically was raised by my grandparents because of behalf my mother, she was out trying to work for our needs.

DR: And what connections do you have to salmon?

DD: Well basically (were winter store).

DR: So, could you tell us about salmon fishing when you were young?

RK: Well while I was growing up as a little girl, my step mom and dad, my brothers, they were older. They used to put up lots of salmon. So many salmon. You name it. When they had no fishing, um, closures. Man they used to bring home lots of fish and work on them even when it’s getting dark.

DR: And how is that different from today?

RK: Nowadays too many fishing restrictions we hardly get any more fish. We don't put up lots of fish like back, back then.

DR: And for you (boyaq) could you tell us about salmon fishing when you were young?

DD: Mmmm basically we (inaudible) as much as she said she use to got.

DR: And how is that different from then today?

DD: Well, restrictions. And now I never thought of that you know. Those restrictions, we rarely hit those first runners. First runners of the salmon. And. Well. It's because uh, well

for maggots you know before house flies start to turn into maggots on the fish. All that breaks loose. That's all part of the restrictions.

JE: And so the restrictions are wrecking the you guys, cause you're running into the maggot and fly season.

DD, RK: yeah

JE: Whereas before you, that wasn't really a problem.

RK: Cause when the first fish come in there's hardly any flies flying around and when they fish are really tight coming in. All of these flies are everywhere. Start putting lots of maggots. Start speaking on the fish. So we have to watch them very closely.

JE: Okay.

DR: And those are the fishing restrictions you guys were referring to?

RK: Yeah.

DR: Okay. And so what values were important to you growing up?

RK: My values… My mom taught me really young how to take care of food on hand right away. Like seal, furs, salmon, anything. She taught me a lot. But now I teach my grandkids even when they're young how to cut fish, work on seal, work on (unknown what was said), etcetera.

DR: And where you boyaq, I know they are values applied to hunting. Do you know what kind of hunting. Do you know what kind of values that you are correlated with? Or that you apply with?

DD: Fish?

DR: Mhmm.

DD: Well with fish there's uh, moose, seal, animals. Such as seal, walrus, um, and land mammals like um, moose, caribou.

DR: Are there any values in that with hunting? Or..

DD: Basically the same thing (inaudible).

DR: And do these values help you see everyday lifestyles?

RK: You know every year, the food they hunt for is getting less every year. And more restrictions. It's what I don't like about. When they, I'm trying to store as much as I can for

winter when they're around and available. And I try to take care of them right away. Before they rot.

DD: Because we ain’t get any (inaudible).

RK: Yeah. Besides the stores here prices are sky high like triple prices from the store here.

JE: So those values taking care of food, help your community, basically it sustains your.. Allows you to continue.

RK: Yeah we mostly eat Yup’ik food as our main source of food here.

DR: What values did your guys' elders used to take care of salmon?

RK: What?

DR: What values did your guys' elders use to take care of salmon?

RK: They were taught down from their mom and dad and they passed down to us. We listen they teach us. They teach us. Uh, which they were taught from their mom and dad.

JE: Do you guys feel like teachings and rules that you learned maybe at fish camp or fishing or hunting, those are still being followed today?

DD: Mhmm.

RK: Yeah.

JE: And the village is still pretty strong?

RK: Yeah they uh, that's why tribal teach my grandkids at young age.

DD: Generations after generations.

RK: Mhmm.

DR: How did you guys learn to care for salmon when you were growing up?

RK: My mom and dad, my brothers and sisters they taught at young age. When were we won't forget from a young age. They taught us how to (inaudible) on hand. With using our hands. With instructions.

JE: Do you know how your ancestors managed or cared for salmon? Like were stewards of salmon before all these other like Fish and Game or US Fish and Wildlife.

RK: Back then there was no F&G not that I know of. They used to, they would take good care of their dried salmon, put in barrels, watch them closely, try not to let them rot or anything. They even used to stir in salted water. Since they have, back then, they had no freezers or nothing. They used to stir them even under the ground and put the fermented, uh, the seal oil under the ground as refrigeration or like uh, freezer.

JE: How do you think that back then they maintained healthy populations of salmon and wildlife? Like maybe like, you have any rules that you know of, making sure everyone had enough, you know how like managers maybe restrict the mesh size or the length. Maybe like your net can only be so long like, do you know any traditional ways of kinda governing in this area?

DD: Maybe more restrictions.

RK: There's no, we have no restrictions down there down in the ocean or in hunting grounds.

DD: (inaudible)

RK: That and there was before lots of houses were built here. Maybe only 4, 5 houses were here and they used to be caribou around here.

JE: Wow.

RK: Back then.

JE: Really.

RK: Yes.

DR: Do you guys know when they stop fishing for salmon?

DD: Yeah, um. Certain time of the month. Like. (inaudible) but maggots. Um, we pretty much catch on the peak run and then you know fish on until we get more than enough that we store away before the maggots poop on them.

RK: Yeah.

DD: Hanging fish.

RK: You only get a couple hours of sleep and start working on fishing. So they, when the weathers good, when the weathers good and calm we try to have that dry fish dry right away before the fish start molding.

JE: Okay. And do you feel like your knowledge, indigenous knowledge and values are reflected in fisheries management today?

RK: No

DR: Why do you say, why do you not think so?

DD: (inaudible)

JE, DR: [laughs]

DR: Um, so what do you guys wish that fish managers knew about your community and your fishing practices?

RK: I wish they could quit doing stop restricting our fishing rounds. I wish they wouldn't have to close our fishing areas. So we can store lots of fish for winter.

DR: What worries or concerns do you have about salmon fisheries today?

RK: I'm afraid in the future we might not like put away more fish than we use to. So I'm wishing fish and game would stop and coming around and try to put more closures to fishing grounds.

DR: And with caring for salmon, are there different roles in yours guys family? When taking care of the salmon.

RK: Like I said, as soon as they bring the fish to our fishing table, we try to work on it before the flies come around and put maggots on our fish. And try to work on them right away. And hang em' to dry. When we spoke the weather. Cause when it gets raining, it's hard to keep them dry and spoil right there.

JE: Okay. Do you have any concerns for fisheries today?

DD: Mmmm no.

JE: No concerns?

DD: Mhmm.

JE: Okay.

DR: What do you guys want to see for salmon and people in your region?

RK: More openings. No more restrictions.

DD: Mhmm.

JE: Do you guys have, I think one of the other questions was, is there, if you were to give managers any suggestions for improving the system, um, how can they do better? Other than not restricting your area?

RK: I wish they could come to the village or have a special meeting with council and community members.

JE: And talk about management and whats going on? So coming here?

RK: Yes, mhmm.

JE: Do you have any suggestions? Is there anything else you guys would like to share with us?

RK: I wish F&G would stop managing our fishing grounds.

DD: We just caught a walrus.

JE: Yeah Boyaq just caught a walrus.

RK: Yeah.

JE: Hes ready to cut.

RK: He’s gonna go cut you guys gotta go watch.

JE: Wow. Quyana.

DR: Quyana caknek for your time.

INTERVIEW PARTICIPANT: NICHOLAS DAVID

Interview Date: 10/18/19
Interviewer: Janessa Esquible, Destiny Ropati
Location: Kongiganak
Home community: Kongiganak

Janessa Esquible [JE]: To get started we have Nicholas David?

Nicholas David [ND]: Yes.

JE: And Destiny Ropati and myself Janessa Esquible and it's 3:05, we're in Kongiganak, and it's Friday October 18th. The first thing that we have here is an overview of the project and so basically the project is called Indigenizing Salmon Management and the goal of the project is to use a participatory approach, so like through these interviews, to document Indigenous values, knowledge, management, and ways of governing that are connected to salmon in Alaska, but we're focusing on the Kuskokwim region. So some of the objectives that we're hoping to achieve here is to document Indigenous values, knowledge, management, and governance and then assess the strengths and weaknesses but from a local perspective, your perspective, of the salmon management system and then how can we better include Indigenous values and ways of knowing and governing the salmon into the current system. And so these are some of the objectives we're going to be addressing and as of now you know we don't – there aren't really any risks that we see by participants in the survey but there also may not be any benefits. Ideally we'd hope that this research will create positive change in salmon management but that's you know uncertain as of now, and so the compensation for the interview for you would be $100 for participating so we have some forms here and ONC, the Tribe that I work for and am representing, they'll cut you a check for participating in the interview. This is all confidential so any information we have will be stored in a locked office. It is voluntary as Destiny probably had already mentioned. These are the contacts for here and for the Kuskokwim it will be myself and we have Jessica Black, she's Gwich'in from the Yukon, and then Courtney Carothers who is based out of Anchorage. And this is just, yes you may use my name to thank me for being part of the project. So once we do all the interviews we summarize all the information that people have shared with us. If you'd like to include your name in thank you in whatever you know the final product is of the research you can put yes, if not you can put no. This is yes, you've already said yes we can record you. No, if you prefer not to, and then yes you may take my photograph. So maybe like a photograph of you and Destiny on your iPhone. And this is just your name, signature, and date. So if you could just review and sign there.
{Sounds of signing consent papers. Continuation of oral history archival and check details}

Destiny Ropati: Now we'll get started with the questions. This is Destiny asking the questions. So if you don't mind, could you tell us a little bit about your background, like where you're from, how you grew up, and what connections do you have with salmon?
ND: Okay. Back in December 1953 I was born at Kwigillingok, which is about eleven miles west of us, and that's basically where our family grew up until we moved back. My parents

originally moved here in ’66 so we’ve been living here in Kong since ’66. And I’ve gone to boarding schools for education beyond 8th grade, when I went to three years at Mount Edgecumbe Boarding School and then my senior year I transferred to Bethel High School and that’s where I graduated. And in the fall of ‘72 I attended University of Alaska majoring in music and business administration strictly to get into the music industry. Then during my junior year – for my senior year I didn’t return because I had, the previous semester I had taken too many credits and I eventually burned myself out. And then after that I had a job with KUC at the Community College in Bethel, and then after that based on what my parents wanted me to do was to return home and they were not getting younger so after ’75 or ’76 I started living here permanently and during the course of that year ‘til 2000 I was involved with construction involving the community’s water system and other community projects that came up. Along the way when I was young my parents were mostly gathering a lot of food. Mostly from the sea and the land. And one of ‘em is the salmon. It’s what we basically grew up on year after year and along the way my wife and I learned how to be self-reliant on what we can do to keep our traditions going and as of today that will never stop. So the last – we’ve been married for over forty years and strictly we’ve been living on the land. We don’t benefit from other entities that are involved with the food stamp program or any other sources. We basically rely on our own.

DR: And can you tell us about salmon fishing when you were young and how is that different than today if it’s any different at all?

ND: My dad and I when he took me out salmon fishing back – like I’m 65 now – I think I was 10 or 12 years old, eight years old could be, we used to go to the Kuskokwim river and then my dad he would have me use oar to keep the, keep our drift net straight. And most of the time my hands would blister up because of all that oaring, and here my concern was that if I complained to him verbally I was afraid he wouldn’t bring me along to the next fishing trip. So I kept my hands closed. Didn’t want to see him see my blisters so I kept up with work because I loved going out fishing so much I didn’t want to hurt my chances of him saying, “You’re not going.” So that’s just how I grew up in a fishing family. My wife and I both.

DR: Would you guys go out frequently back then too?

ND: Only during salmon runs and then when I mean salmon runs the first run isn’t only the salmon it’s other species like herring, there’s then salmon comes next, and then other fish eventually show up. So from the water that’s basically our main source of food other than seal, walrus, and other things that we harvest from the waters.

DR: Okay. Would you say it’s the same time running as of now compared to back then with fishing with your dad?

ND: What’s that?
DR: Would you say that the fish are coming back at the same time in modern times, like right now?

ND: No. No no. It’s completely different. Back when I was a small heading out it didn't take long for us to catch the amount of fish that we needed versus nowadays it’s much, it’s completely different the amount of fish that return. Is the way when I grew up we don’t see that salmon anymore come back.

JE: Really.

ND: The numbers I’m not good with, but back then I can remember recalling when we threw our net all we needed was eight-foot length and that would fill our boat. They would be like every web would be just so full of fish that’s basically all we did in certain times like the tide. But nowadays it’s much more not difficult to go out. We got better boats, better outboards, versus what we had back then, but the amount of fish yes it’s changing.

JE: What size nets do you guys use now?

ND: I use five and a half because of the salmon net restrictions.

JE: Five and a half. Is it a long net?

ND: Regular yeah, it’s a regular salmon net that I usually make myself.

JE: Okay. Yeah we use like 150 feet to 300. Depends. But that’s a big difference from eight.

ND: Yeah. I use a full net yeah, and then from our nets I basically learned from my brothers how to make nets, how to mend, how to hang nets. Because it's part of our life, our lifestyle is to keep our nets renewed and so forth.

DR: Were there other values that you learned? You mentioned these are things you learned like mending the nets, taking care of your nets, anything else that you learned whether it was at fish camp, when you were out fishing?

ND: You had to learn how to maneuver your way around where all the – ocean is different out there. Low tide it’s different and high tide you just go straight but then low tide there are certain channels you have to be aware of going back and forth. I don’t rely on technology. I just rely on my instincts where the sandbars are gonna be and where the channels are gonna be versus nowadays younger people use technology just to get themselves where they wanna go. So I’m still old school.

JE: Any other values that you learned? Did you guys grow up going to fish camp?

ND: Yes. She and I had our own fish camp. Matter of fact we still have our own fish camp house below Akiachak still. You go back there – we, since I started working for the school district since late ‘90s we haven’t gone back. We just process our own fish here. We have our own fish rack; we have our own smokehouse.

JE: Anything that you learned at fish camp growing up that has helped you in your personal life or has helped your community?

ND: Well it, the fish camp upriver, it's a much different environment. The climate it’s much warmer and dryer than down here where it’s much cooler and more humid so it makes a lot of difference when you learn how to maintain and dry your fish properly. And the first things we learned when we started doing our fishing here versus what we did was weather because of the climate change and then so we had to adapt with what we did, how we got our fish done upriver, versus what we can do to keep our fish processed. So that was what we basically learned was how to adjust for the climate.

JE: I see. Were there any values or how do you know how to take care of salmon?

ND: It’s basically the look and see. Well first, the first thing you do when they’re fresh, she and I have to always make sure once they’re picked up and brought here that they’re taken care of. Cut up while they’re fresh. You’re gonna wanna make sure they don’t sit there you know because once you pick the salmon out of the water you know how the next two or three days it’s not gonna be the same like you just pulled it out of the water. So basically you wanna make sure you take care of the salmon first. Other than after it’s all cut up we hang it based on the weather we make sure we know exactly when we go through the entire rack to say hey we need to bring it into the smokehouse. It’s not just about smoking the fish. It’s just about keeping the fish from going bad. So yeah we have to deal with the weather and the environment. And there’s a certain time when the maggots and the flies will start laying there – we try to take care of our salmon needs before that maggot season starts.

JE: Oh. When is that season here?

ND: It’s in July.

JE: July.

ND: Regardless if you like it or not it does come. So what we also learned in preserving the salmon was to add brine. Dip it in brine and salt and we store some in buckets with rock salt and other things. We jar salmon for the winter. So everything from the tail end to the head it’s not wasted. The head, salmon heads, we basically the king salmon mostly we store and make salted salmon heads.

JE: Yum.

DR: Do you feel as if the teaching rules are still – do you feel as if the teaching and the rules of caring for salmon are still taught and followed today?
ND: I don’t know if that’s – it all revolves around family structure. So like I said she and I basically grew up depending on salmon. If we don’t do any salmon for the winter then it’s different. I mean how other people that don’t rely on salmon are beyond me. She and I have to; it’s just how we live.

DR: How did your ancestors care for salmon? Was it the same as you?

ND: It's not the same. Back in the earlier days they didn't have the luxury of having electricity. They didn't have any freezers, and now these other things you know to store the salmon. They had their way of preserving the fish for the winter, but nowadays we have the electricity, we have freezers, and other luxuries that our parents didn't have.

DR: And how did your ancestors manage or steward the salmon? So before we had all this outside you know kind of like Fish and Wildlife or Fish and Game managing – how did they manage the salmon?

ND: Back then I never heard of any management. It was basically as needed. Or like the instinct for this is the salmon is here, we need to do our salmon thing. So back in those days I never heard of any restrictions at all.

DR: When would you guys determine or know when to stop fishing for salmon without the management? If there was no existence of the regulations.

ND: Well there's several ways. First is the amount of time given to us when the agencies tell us that the salmon is gonna be open. And it depends on how much is being caught and then when she and I think our quota for the winter is enough is when we stop. We usually meet our needs but with much more restrictions it's much more different than what we did twenty, thirty years ago because of salmon restrictions, especially the king salmon.

DR: And what do you wish fish managers knew about your community and your fishing practices?

ND: What do they need?

DR: What do you wish the fish managers knew about your community and your fishing practices?

ND: One of the things I'd like to see is the fish management, because geographically we're out there in the open and the Kuskokwim is wide and long and the salmon they're be times that there's salmon runs that might be better fishing for upriver people but for lower when all the fish go past us that's the part that is most difficult. Now the salmon runs are past us and basically when that happens when people, some people don't catch enough, it will hurt them. It will help the upriver people but downriver you know.
JE: Hmm. So you wish they knew more about how to find a balance to where they like?

ND: It has to do with the weather mostly.

JE: Does it? Okay.

DR: Do you feel like your knowledge or values are reflected in fisheries management?

ND: Yes.

DR: And how so?

ND: Yes. My knowledge goes back 40-50 years ago. Our environment is changing. Our environment wasn't the same as it used to be 40-50 years ago because of all this pollution from climate change. It's as if our world is going in reverse osmosis. It's not – I'm afraid of the next 20-30 years. That's when I look at our younger generation. What they're gonna be like if the climate changes, what this earth is doing, what the people are doing to pollute this earth with all the oil and any other pollutants. It's irreversible. It's as if the world nowadays is in reverse of how the earth is made. So it's gonna slowly – I think we're at a point where it's not gonna return like it used to be. That's what's troubling me.

DR: And what worries or concerns do you have about our salmon fisheries today?

ND: A lot. Like I mentioned what's gonna happen in the next 20 years? Will we see the same salmon? No.

DR: Do you have any concerns with the fisheries management too?

ND: I have a lot of concerns. One of them is I think they need to do more management of what happens to where the fish are in the wintertime. Right now they're out, most of them are out there. What happens in that water is what's gonna happen to the salmon? I hear of big trawlers. I hear stories of people working in those huge trawlers. I heard there was a lot of salmon bycatch, which concerned me. A lot was taken out of the water, especially the king salmon that I know of. So it is – that portion of the problem I think nobody has addressed really what the salmon nowadays is doing out in the open until it comes you know when it's their time to come up and spawn.

JE: Hmm. Do you have any opinion on what works well for salmon management or what's the positive or the negative?

ND: The positive I can know is their concern with the spawning areas and the amount of fish that come up. Those are some of the things that I like about our fishing management, but I know there could be improvements.

JE: And then improvements with the bycatch? Any other areas to improve on?

ND: The bycatch is basically what I mentioned before is what still concerns me. Because I've heard stories about a couple of residents who went out to these trawlers to work. They were telling me that they were throwing away a whole bunch of king salmon out with the water – the bycatch.

DR: What do you want to see for salmon and people in your region in 40 years?

ND: I want people to continue to rely on salmon like I do. Like we do. She and I don’t wanna stop. So I hope I’m saying this in a positive manner with other people. Not only us but for other people here, upriver, and whoever can rely on the salmon.

DR: And from fishing for this year’s season of 2019 were there any weird cases of your salmon catch or did it like- did some of them look weird or was there a lot of die off of the salmon in this area?

ND: No. We’ve seen some differences when we were processing and when she was cutting up the fish some of the fish had some spots in the flesh that didn’t look normal?

JE: White?

ND: They were like yellow-ish green. A lot of spots and we tried to avoid processing them, so we just threw them away. Even though we didn’t want to because she and I don't waste, nowadays there’s certain salmon that don’t look healthy at all.

DR: Do you have certain favorite memories with your dad from fishing?

ND: My favorite memory was not telling him I had blisters. He wouldn’t take me along if I complained, because I still love going out fishing. One time I had an injury on my leg. I had a big gash about that long and about that wide from a job injury and these guys were heading out fishing and you probably guess what happened to me when I saw them leave. I was crying.

DR: How long did those blisters last for?

ND: Almost a year. It was a bad injury. So in one season I couldn’t go out fishing. I tried telling her, but she said no you’re not going. I’m not gonna go back with you to Bethel. Your legs are gonna get infected so she said no you’re not going. That’s how much I love fishing.

JE: Wow. Is there anything else you want to share?

ND: The only thing I wanna share with you guys if this is helpful is to determine some of the things that can be helpful for you versus helping other people. That's my one concern.

JE: Helpful to us?

ND: Helpful to you and to pass on the message to other people you know. We can still have our salmon livelihood and keep this going. That would be awesome, that's why I care.

JE: Yeah. Hope so.

ND: The only thing that worries me is our environment. Like I said it's irreversible with all the damage that's been done to nature already. It's like this whole earth is restructuring itself as to how it's made and with all the damage that's been happening it's alarming. I'm done.

JE: Quyana.
(end of recording)

INTERVIEW PARTICIPANT: PAULINE AND JONATHAN EGRASS
Interview Date: 1/31/2019
Interviewer: Janessa Esquible
Location: McGrath
Home community: Aniak, McGrath

Shorthand key:
( ) Note about what is happening during the interview, unclear of word, or spelling question
[ ] Words not said but added for clarity
{ } Action
Mmm-hmm, uh-huh are affirmative
Hmm-mmm, uh-uh are negative

Janessa Esquible-Hussion [JEH]: Yeah you can start with your name, um, your home community, your cultural affiliation, and your age.

Jonathan Egrass [JE]: Um, my name is Jonathan Egrass. I'm 30. I'm, this is my hometown, McGrath. Um, I'm Athabaskan.

Pauline Egrass [PE]: I'm Pauline Egrass. Um, my maiden name was Lane. I'm 26 and I would call Aniak my hometown, but I've lived up and down the Kuskokwim. Um, and I'm Yup'ik.

JEH: Okay. And Janessa here. (States project overview). Okay first question is tell me a little about your background, where you're from, how you grew up, and what your connection is to salmon.

PE: Um I grew up around Aniak. My mom was originally from Kalskag. And her and her sisters would put salmon away. My aunt was a single mom so it was really important for her to make sure she had salmon put away and we'd go down and uh, kinda just be a little kid there. Run around and take fish hearts and eat them and stuff like that. But I got to see all of the salmon being put away and learned how to cut fish when I was really early, or really young, I mean. And um, I mean I always enjoyed all of it and then how it brought us together and so, I wanted to be able to do it. But my parents weren't together and we primarily lived with my dad and he didn't fish. And he didn't know how to put fish away. So once Johnny and I got married and had our own families, I wanted to learn how to do the nets and how to smoke fish and put it away for winter. So that we would be able to do that for our kids. Um, and it was always kinda harder up here in McGrath because by the time the salmon get here they're old. Um.

JE: And the river is so silty too. They're not, you know, we have to do set net or um, we have a community fish wheel. But it wasn't used last year.
PE: So we went and learned with Johnny's sister how to do a set net and how to put fish away. And how to smoke and how to jar and everything so that when we decided to do salmon further upriver here we were able to do it by ourselves.

JEH: And how come the community fish wheel wasn't ran this summer?

JE: I'm - I don't know. That's a good question but my thought on it is that there were having trouble in the years past with people not being able to take all the fish, you know. Like they would have to stop the wheel at times because people were getting overwhelmed with fish basically.

JEH: Oh okay. They didn't want to waste or anything?

JE: Yeah. So they would stop the wheel and instead of putting it in and letting it run for a little bit in, yeah. That's what I think.

JEH: Okay thank you.

PE: I think there was a large break in the community for years where not a lot of people fished. There was a while where there was only a couple people who even had set nets. And when the fish wheel was put in, I think that families were not used to getting fish like that.

JEH: I see.

JE: In larger quantities, in larger amounts.

PE: Yeah.

JEH: Okay. Was the fish wheel put in and was it not here all year, or most of your life? It was just more recent?

JE: Uhhh, yeah this was a community fish wheel that was paid for by different entities around.

JEH: Oh okay.

JE: Um, to get materials and pay for the labor to construct it and all that kind of stuff.

JEH: Okay. Did you want to share anything? On how you grew up and your connections to salmon?

JE: Um, my connection to salmon isn't nearly like hers. Um, I grew up here. I do remember my grandma putting fish away when I was little. She has a smokehouse too that she used. I remember we never smoked any fish at my house growing up. We did every now and then again but it's not something that was a regular every year occurrence. Um, but I remember my mom always getting salmon from the Yukon because that's where she's from. She's from Grayling.

JEH: Oh okay.

JE: Or Anvik but she grew up in Grayling. So they would always send her king salmon over.

JEH: Oh okay.

JE: And so my.. Growing up I guess mostly what I had for salmon was um, you know, king salmon fillets or stuff like that. Instead of having strips or stuff like that or jarred fish or whatever.

JEH: And mostly from the Yukon?

JE: Yeah. And then like she was saying, um, we went down to my sister who lives in Red Devil.

JEH: Oh okay.

JE: So Georgetown, Red Devil, Sleetmute area that's where they do all their fishing and uh, that was really fun to do. A good learning experience because there was so much more abundance it seems down there. I guess it's more of easier access to different streams but.

PE: And they're not as old.

JE: Yeah.

JEH: Gotcha. So you mean it's kinda harder to fish up here.

PE: Mmhmm.

JE: In a way, yeah. You have to know the areas to set and um, like, I know, probably smoked a little bit already but these are really red. That's how it is down there. And then when we're up here there's so much further upstream that the meat is more..

PE: White.

JE: Yeah

JEH: Oh really?

PE: Malluk

JEH: Okay. Gotcha. And…

JE: So it's harder to smoke the fish up here I guess because of that.

JEH: Okay

JE: Because, uh, you strip them, and sometimes the meat will want to try peel away from the skin.

JEH: Mmmm.

JE: And then, uh, down there it's somewhat more firm.

PE: Firm.

JE: That it holds together better.

PE: And it was such a fun good time with your sister down there. That when we came back the next summer we helped out with his sister here to help teach them. They built a smokehouse that next summer and we helped them smoked their fish.

JE: Yeah and it's, they knew how to do it, but they wanted to know how we learned to do it with my other sister.

JEH: Oh okay

JE: Because they would do strips and then do half dried jarring. And then we would do, we would make strips. Like dry fish. You know.

JEH: Okay. So you shared that with your other sister.

JE: Mhm.

JEH: Thats awesome.

JE: So Ruby is the one down in Red Devil and Renee is here.

JEH: Oh okay. That's really cool. That you guys got to experience it together. And for, how is the salmon fishing when you were younger compared to now? Are there any differences from the times that you do remember? Going out fishing?

PE: It seems like we don't see the big giants anymore.

JEH: Okay

PE: I remember my aunt bringing home giant salmon, just, that were enormous and it seems like we don't see those anymore. Um, our family fishes for silvers mostly, um, if we're having a good year of kings sometimes we'll take kings that are given to us but we don't do sets for kings. But definitely the kings don't come up like they used to.

JEH: Hmmm.

PE: Um, and it almost seems like in the last couple years there been more jacks.

JEH: More jacks?

PE: Mhmm.

JE: Yeah I've heard of people catching them in their nets in stuff.

JEH: Hmm. Are you seeing that with the silvers too?

PE: Mhmm.

JEH: With all the fish?

PE: Mhmm.

JEH: Okay. And were there any rules about harvesting or sharing when you guys were fishing together. And if so, how were they enforced? Or like even your parents or grandparents?

PE: I don't, I think I was just so young that I never paid attention to what, if there were regulations at the time but I don't. It seems like it was open all the time.

JEH: Open

PE: When I was growing up it wasn't regulated like how it is now. And her smokehouse was always full. Always full. It was always with, she did kings for herself. Kings and reds. And then she would put up dogs because she had a dog team.

JEH: Okay.

PE: And so I mean she was always working really hard in her smokehouse and was always full. But and I even been down there recently but my mom told me that they just, any chance there's an opening, they're on it.

JEH: Hmmm.

PE: Just so they can get what they could. Um, and before where they didn't get silvers as often. Now she has to get silvers.

JEH: Okay.

PE: Um. Back when she was rich and can pick her kings and her reds. But yeah. She gets silvers now.

JE: Uhhh I never really remember the regulations either for one because I didn't fish as much when I was little. But, um, more in the recent years, uh, I think the kings have been open past year maybe past couple years here but with no restrictions. You can set net them or fish wheel them or something but you can’t rod and reel. I don't know.

JEH: Okay

JE: I don't really know.

JEH: Yeah.

JE: Um, but part of the reason why we'd go down and go with my sister and just get silvers was because of all these regulations and the closures and stuff and um, it was just easier for us logistically. They're still good fish. So.

JEH: And are there any, so for rules about harvesting and sharing, like anything just in your family or Yup’ik or Athabaskan values that you learned about sharing your fish when you get it um, yeah any teachings?

PE: When we do our set net here, before we give to anyone or before we take we definitely go and ask the elders.

JEH: Okay

PE: Because there's a number of ladies here who just jump up and down when you give them any fish.

JEH: Yeah.

JE: Yeah. Definitely. And as the summer comes and you know, before fishing opens or before the fish are here, um, we’ll ask the elders to you know, like hey if we start getting fish or if we have the opportunity to start getting fish do you guys want fish. And yeah.

JEH: Okay

JE: We are always sharing. Yeah. And not just salmon, it’s you know, it’s whitefish, it’s sheefish, it’s any of it..

PE: Or if they go out first bring birds or…

JE: Yeah.

JEH: Okay so everything.

PE: Mhmm.

JEH: And do you, either of you, remember when you were younger were there any like just rules locally like even though there weren't any restrictions maybe by Fish and Game, or whoever, um, about like where you put your net or how you fished, keeping in mind of others.

PE: There's different families who have their areas where they set regularly kind of just, you don't set in another families area..

JE: Yeah.

PE: I mean it's like the same with like trapping or…

JE: Yeah and if we ever do try and set somewhere we know others have set in the past. We usually try and ask.

JEH: Okay.

JE: Um.

PE: And most of the time people are really willing to share and give tips.

JEH: Okay.

JE: There, I think a lot of it that people are excited for us as a young couple and young family to be doing this and…

JEH: Yeah.

JE: Probably want us to be able to go out and experience it and do it ourselves.

JEH: And, are these types of things you shared, are these still followed today. Do you feel like more or less people continue to share and provide for elders and respect each other's traditional fishing or set netting spots?

PE: Mhmm.

JE: Yes.

PE: Yes.

JEH: Can you, how do you think your ancestors managed, or were stewards or cared for salmon? Like before we had any of this Fish and Game coming around?

PE: I feel like they understood not to take more than you needed. I'm, just from having to put away fish and all of the hard work that went into taking care of it and making sure that

none went bad or was wasted. It was such hard work and I couldn't see anyone take on more than they can handle. In the past when we were first learning there was those times where pieces spoiled or things didn't smoke right or something where the meat wasn't edible and man it just felt bad. So I don't see anyone wanting to do that.

JEH: Mhmm.

PE: And especially..

JE: Yeah they relied on a lot more.

PE: Yeah

JEH: They did?

JE: I agree with that.

JEH: Okay. Do you feel that your knowledge, wisdom, and values are reflected in fisheries management? Some more like Yup'ik and Athabaskan values into this more western fisheries management?

PE: Yeah.

JEH: Okay.

PE: I can see that where people get mad about the regulated times before but it reflected because there were those years where the king opening wasn't very long but then in the, I mean last year we had a better year of the kings coming back. So that I feel like it reflected upon the actions that were taken.

JEH: Okay. Good. How about you?

JE: I agree. I mean I..

PE: I feel like a lot of things we're on the same page because we have to work together too. {laughs}

JEH: Yeah, yeah {laughs}

JE: Yeah we talk about all this.

JEH: In it together.

PE: Yeah

JE: We talk about all this stuff too.

JEH: You do?

JE: Yeah I mean when we're doing it when it's happening, when we're fishing and all that kind of stuff.

JEH: You feel pretty content then.

PE: Mhmm.

JEH: Okay. Do you have any concerns about your salmon fisheries other than the smaller size and stuff? More jacks?

JE: No

PE: Nothing that.. Like sometimes we'll get a whole group of fish that have lots of worms in them but that just like happens.

JE: Yeah.

JEH: But you’re not seeing like more worms than usual? Just about the average?

PE: Yeah.

JEH: Okay. And do you have any concerns with the management of the fishery? Now? In the past?

JE: No.

JEH: No? Anything you think works really well that managers are doing? Or things that like maybe don't work so well or?

PE: Um, I think it's good.

JE: I think the closures really worth, like she was just saying..

PE: Yeah. And..

JEH: Closures are working?

JE & PE: Yeah.

JE: I would agree I mean just to see the numbers of the fish come back up. Yeah.

PE: Like it's hard right now but in the long run it will have a good benefit.

JE: Yeah.

JEH: Okay.

JE: A lot of people don't like it. Lot of people don't want it to happen but..

PE: But those are people who rely mostly on kings and.. I feel like we probably have gone and then like the king fishing, but it's such a prize salmon that we just.. And silvers are good.

JEH: Yeah.

PE: So we're happy with that.

JEH: Okay. Is there anything then that you would improve or no? Sounds like you guys are pretty happy.

PE: Were happy {chuckles}

JEH: Cool. Okay. Anything that you want to see for salmon for your people in the region in the next 40 years?

PE: I'd like for more people here to learn how. I think there was the kinda gap were people our age and maybe the generation above didn't really fish and..

JE: Didn't appreciate it.

PE: Yeah. They..

JE: Like..

PE: I think that their parents fished and didn't really do the best job teaching the next generation and we had a large gap in our community where we had no one really fishing.

JEH: Wow.

JE: Well there was fishing but it wasn't like..

PE: It was just like certain individuals who..

JE: Yeah. The people that had dogs or people that put away for themselves instead of..

PE: They just didn't have a real interest in teaching.

JEH: Mmm. And did that be like your parents or like maybe people in their forties or so?

PE: I'm not gonna lie even like your parents.

JE: Well they didn't do so much fishing but yeah.

PE: Um, but yeah. Um. People feel like people maybe in the 50s, 60s where I don't know what..

JE: And there are groups of people where they teach their families but the other thing that is happening is that there's a lot of people leaving. So they might have taught their kids but then those kids have since moved away and there, and maybe they fish where they are now but.. you know.

JEH: Yeah. Do you guys have any thoughts on that? Since you've been tied to McGrath for so long? Like the land and you know it so well on the resources. With the population declining, do you have any concerns about that?

PE: I mean of course. {chuckles} But um, I mean there's little things we've done to step up. And we have two young girls here who we want the community to grow and to prosper but the hard part about it is that we're not going to sacrifice our kids to stick around for here. Um. But while we're here like we said we came back and helped his sister's family, which made their kids really enjoy it. I mean our girls so we boat down to Red Devil, just like a 190-mile boat ride.

JEH: Wow.

PE: But our kids always enjoyed it. And then the best part about it is our kids being little kids who talk to all their buddies. And so when we’re doing fish and cutting stuff like that usually our kids are running around with their friends. And then they eat good fish and they tell their parents. I feel like slowly we're trying to get more people to do it, and it’s worked but it's been slow.

JE: Yeah. I feel like us doing our own fish has definitely encouraged more people to try, at least try.

JEH: Especially like younger folks.

PE: Mhmm.

JEH: That's great.

JE: Last year we did uh, I did a set net at Black River down by (F. Healy) like an hour and a half boat ride from here. I think it's like a 50 mile boat ride. Um, silvers run up that river. And I went down. I couldn't get anybody our age to go with me, to go and set the net. Nobody wanted to go. And so, um Helen Evan, an elder here, went with me and we went and set the net together.

JEH: Okay.

JE: You know an elder, because she's like 50, yeah just can't find anybody to go. I'll go with you. And so I took up on that offer because she was here working.

PE: I had to stay on call.

JE: And yeah. And so that you know the runs aren't really long so when you have the opportunity you got to take the opportunity and go out and fish. And did in the week and yeah. She went with me and we got like 26 silvers. I just set it once you know.

JEH: Okay.

JE: One dog. But that was my fishing last year. Thats really all we did.

PE: Yeah we didn't get much but.

JEH: But you took an elder out with you.

JE: Mhmm.

PE: And she had a good time.

JEH: {laughs} I bet.

JE: She was really excited because she went out on a boat ride and didn't have to be the driver. It's usually anywhere they go she's the driver.

JEH: Wow.

PE: Yeah she's tough as nails.

JEH: Yeah. Wow. Anything else you all want to share?

JE: No. I don't think so.

PE: Yeah. {Phone rings}

JEH: Thanks so much for your time today.

INTERVIEW PARTICIPANT: NESTOR AND NAOMI NORBACK

Interview Date: 1/31/20
Interviewer: Janessa Esquible
Home Community: McGrath

Janessa Esquible [JE]: {shuffling and mumbling} So if you all want to go around and just state your name and your home community and cultural affiliation and then the age.
Naomi Norback [NaN]: Okay, I'm Naomi Norback. I'm 63 going on 64 in March. I was born and raised in McGrath and I'm Inupiaq and Caucasian.

Nestor Norback [NN]: And I'm Nestor Norback

NaN: How old are you?

NN: 62 going on 63 and I'm um half Finnish and Eskimo, so Fiskimo

NaN: Inupiaq

NN: Yeah

NaN: We're from Nome area.

JE: Nome area.

NaN: We somehow got around here. Our families.

JE: Oh okay.

NaN: That's our tie though.

JE: Okay, awesome. And, let's see. I'm Janessa. I work for the Bethel tribal council,so Orutsararmiut Native Council. I'm 29, umm and my ties are back in the Great Lakes Region, so I'm part Ojibwe and part Mexican

NN: Mmm.
[did not transcribe review and completion of paperwork]

JE: The check will get sent out next week from the Orutsararmiut Native Council. Thank you. Alright, looks good. And just here, can you just check yes for me and yes there? That's just stating that you said you were okay with it being recorded and having it archived. Okay, great thanks. Alright, you guys have any questions before we begin?

NaN: No.
JE: No, okay. Can you both tell me a bit about your background, how did you group up and what are your connections to salmon?

NaN: I was born and raised here in McGrath and background. I worked as a teacher most of my working life and umm also with forestry, fire, the fire camp as an admin and um connection to salmon. It’s just a part of our diet, you know it's a part of our subsistence diet. Like I said if we don’t have it, we miss it.

NN: Like this year, we sure could...next year were going to have to set a net so we can have salmon this time of year it’s nice to have salmon this time of year...we kind of miss it, you kind of crave for it, were wishing for some holy cross nice juicy salmon strips earlier.

JE: Yum

NaN: You got to give her your background.

NN: I was raised in Red Devil. I used to fish down there with my dad’s friends and used to catch some big fish you know. Probably 60-70 pounds. You don’t see that anymore now a days. I don’t know why but I saw that thing on TV saying that killer whales are eating them but I don’t know. Killer whales have been around a long time too. I think it's the Japanese and foreign...people fishing out all the big fish anymore. They don’t get to be big anymore, so I don’t know. Well anyway, and then we’d commercial fish with my dad and family back to the 64 and 73 over on the Shelikof strait over in Uganak Bay in Kodiak. We ate alot of fresh salmon too then. Now a days here we set nets and stuff and you know, we kind of miss it. We didn’t set any this year because working on the boat, like I said we’re missing it, wanting, you know craving the salmon. This next year were gonna set a net.

NaN: And we usually put up fish. We do it for about a week and we cut it

NN: Jar it

NaN: Can it and smoke it.

NN: smoke it

JE: Canned and smoked? Okay.

NN: And then whole fish too..

NaN: Yeah, freeze it, yeah.

NN: You know, freeze it whole and

JE: You don’t cut into it? You just freeze it like that?

NN: It last longer.

NaN: It doesn’t, it doesn’t freezer burn like that.

NN: Freezer burn as fast. You putting it in food savers.

NaN: Oh yeah, vacuum pack. That works but it's alot of work.

JE: So freezing it whole?

NaN: Yeah.

NN: It tastes better when you thaw it and stuff you know.

JE: And, you kind of touched on this some Nestor. Can you tell us about salmon fishing when you were young and how it's different today?

NaN: What he said, from what I can remember the fish were way bigger.

NN: Back in the old days, yeah growing up as kids huh, compared to what it is now a days. You don't, you catch a fifty pounder anymore that's like holy cow you know.

NaN: Some of the story, like the uh Nikolai stories, there's a story about when they walked to the mountains and there would be families walking and in one of the stories they said that they stopped to eat on a bar and there was, there were dead salmon but they could still eat them. The salmon, and I don't know how many people there were, maybe 20 people. There was a group of them anyway and they ate that one they said, so the fish that they found must have been still in fairly good shape and it was big.

JE: Wow.

NaN: But this was you know years ago, like back in the, I don't know it's a (mistodia) story from Nikolai so it's probably back in the 1900s, you know early.

JE: Early 1900s

NaN: So the fish were way bigger.

NN: Yeah, compared to now a days.

JE: You think they were better like quality too or…

NaN: I don't know about the quality but…

JE: The fish you get here, are they still pretty tasty?

NaN: The early runs are then later in the run they're kinda, not as red and…

NN: Firm, you know oily, not as much oily.

NaN: Yeah they're mushy.

JE: Oh, later on

NaN: Yeah, later in the run they're not as good, but early in the run they're pretty nice. You know, they're red and firm. But the later run…

NN: Mid-run, I guess, I don't know what it is.

NaN: Does it seem like they're not as red? There's more soft, pink meat.

NN: Yeah

NaN: Then it used to be like maybe. It doesn't seem like there's many...cuz up here we expect them not to be as good as down there, but some of them are you know.

NN: Good

NaN: But it seems like anymore, they're more, more of them are not as firm and not as red as they used to be.

JE: Okay.

NN: Global warming crap they talk about too I guess. Huh. Gotta lot to do with it. I don't know.

JE: Global warming

NN: I don't know...I'm still having a hard time believing about that global warming because people talk about..I hear stories about that it used to change weather too back in the old days but I don't know. It could be true from what we seen though. I don't know though.

JE: Not sure…? Yeah.

NaN: It is different. It's definitely different. Cuz we grew up when we would have weeks of fifty below or more. Weeks of fifty below when we were kids.
NN: And then it'd be cold too at the beginning early you know. Like this year it's been warm.

NaN: November

NN: Yeah I guess there is such thing as global warming.
NaN: Well they said, that some people think the earths, the native people think the earth's shifted, the axis or something.

NN: Yeah, could be a little bit of both huh. And then the pollution, so I don’t know.

JE: Pollution too?

NaN: Air pollution, I think.

NN: Ozone layer and all that, were not scientists, but that’s what the scientists seem to think. And they could be probably true too. I don’t know.

JE: Were there any teachings and rules that uh, about harvesting, sharing when you guys were younger and even today, and how those rules were enforced?

NN: Not as much as there is today because there’s more people at fish and game that's managing it more than what it used to be. That’s from what I can remember. I don’t know maybe they did have…

NaN: I don’t remember

NN: No, I don’t remember that either. Even like fishing when we first started out fishing on Kodiak. I remember we used to fish all the way through the season and then mid-way through the ten years we were fishing they started coming up with these regulations and I remember having to pull the net more often so yeah they did start changing because fish and game was following the years. Well they probably did all the time, but I don’t think they did as much as the early years compared to what it is now a days you know?

JE: Yeah.

NN: More people are involved I guess, watching it. I don’t know.

JE: More people monitoring.

NaN: I noticed even now though I don’t remember from a long time ago because my parents were not really...my dad wasn’t really that much subsistence. He liked to fish but he was more like a pike fishermen, you know. Sheefish

JE: Gotcha

NaN: So I don’t remember alot of salmon, but I remember them buying...they bought salmon from over on the Yukon. They would always every year get blackfish and salmon strips from I think it was a Homie over there on the Yukon. My mom would always get that. So I don’t remember from being a kid what they did when they harvested but now...it seems like if a person has a net out, they usually make sure the Elders get fish. Like even right now um, another person, could interview would be Rene Egrass
JE: Okay

NaN: Her husband is Detrich Nikolai, and they always put a net out and they make sure that the Elders got at least a salmon when they put the net out.

NN: Yeah, when the first run comes up.

NaN: Yeah. So I think now a days they're still following that.

JE: Following that? Okay, so sharing with the Elders first.

NaN: Yeah.

JE: Okay. And, that's the next question...are the teachings and rules still being taught and followed today. Sounds like they are.

NaN: I think so, yeah. I think that's engrained in some people pretty well. To carry it on to this generation.

JE: And you think it is getting passed onto this next generation?

NaN: Mmm-hmm

JE: Do you know how your ancestors managed or stewarded or cared for salmon before we had like fish and game or fish and wildlife service?

NaN: From what I know, they didn't waste. They didn't, there was no waste. And what they took, what they took, they took care of. I mean they might of had lots and lots of fish. They probably put up, couple, lots, as much as they possibly could cuz they had to survive off it you know?

NN: And they did some for dogs cuz that's all they used was dog teams back then. Hardly anyone's a musher now a days.

JE: Okay.

NN: I remember, my family getting bundles of fish for dogs, you know dog food back in the old days, back in the 60s, 70s. It's not that way now a days.

JE: Just not as many fish?

NN: No, not as many dogs either so no one makes bundles of fish like they did a long time ago. For dog food and human food.

NaN: For dog food, they freeze it I think anymore.

NN: Mmm-hmm.

JE: Gotcha, okay.

NaN: Nobody really dries fish anymore.

JE: Not as much as they used to?

NN: Yeah.

JE: How come do you think?

NaN: I don't know.

NaN: Why there's not as many people processing fish.

NN: I don't know. It's a thing of the past, the old days, you know, that's kind of like losing, you know like, old people make snow shoes and nobody wants to learn and you know, so they lose that. Life has changed.

JE: Like it's an older tradition. Do you think it's because people don't have time or? Just the traditions not being passed down?

NaN: It could be because there's a set time limit of when you can fish. There's only a certain period of time you can get the fish and if something happens where you're not here, or say..

NN: Times have changed, you know

NaN: Or if the weather is different, or if the water is high, you know but we could get enough fish for us in like 5 days.

NN: Yeah

NaN: So you know it could be people are more busy, you know everyone's got jobs and its alot of work to get and to take care of it.

NN: Mmm-hmm

NaN: When you get a net full of fish, like some people were raised cutting two hundred or hundreds of fish a day.

NN: Back in the old days.

NaN: Yeah

JE: Wow.

NaN: And we get maybe 12 a day

NN: Yeah

NaN: And we think that’s alot {chuckling}

JE: That’s a good number

NN: Yeah

JE: Me and my partner don’t need much either. That’s pretty nice {chuckling} Okay. And do you guys feel, either of you feel that your knowledge, wisdom and values are reflected in fisheries management?

NaN: Well, they’re watching what’s being caught right?

NN: And they’re trying to make sure there’s enough for the next generation you know. And without it, it might get wiped out.

NaN: We must have some kind of management. People just can’t go overboard with it. And it seems to me like they’re watching out for the whole river. Do you think they’re watching out for us up here, upriver compared to downriver?

NN: Yeah, I think so, yeah you know. I hope so.

NaN: I get that feeling that they’re, you know they’re thinking as they’re managing it, they’re thinking of each area as they go up.

JE: Yeah, and when you guys think of managers do you think fish and game, the fish commission, Kuskokwim River Inter-Tribal Fish Commission, Fish and Wildlife Service.

NaN: Fish and game

JE: Okay.

NaN: Fish and Wildlife Service I don’t really have much respect for that organization.

NN: The feds?

NaN: Yeah.

JE: How come?

NaN: Uh, I just get the feeling that they’re too into regulating. We’ve had some bad experiences so that kind of colors our thoughts. But, I think the state people are doing a good job. I don’t really feel like the federal, you know there’s not as maybe they don’t

have the stock in things as much as people that are living here, that are going to stay here, that are part of the state, like you know what I'm saying?

JE: That's an Innoco, I forgot, thats your feds, right?

NaN: Yeah, Innoco. And you know actually did pretty good, you know they tried to train up local people they had young people they mentored, Kevin being one of them. Kevin Whitworth. There's another. Snow boy, Paul

NN: Patrick

NaN: Yeah, they did. Innoco was pretty good, but I don't know federal is just so straight and narrow. To me there's no flexibility there.

NN: There's no common sense it seems like. You know like being out there and actually living off the land.

JE: Yeah. Have you heard anything about the Kuskokwim River Inter-tribal Fish Commission? Do you know of any of their management, or co-management efforts?

NaN: I've heard of it but I don't really know a whole lot. I know Johnny Samuelson is involved in it.

NN: Which is just started how many years ago, since we've found out about. A couple, three years ago or something you know. We found out or heard about it. That's who you work for?

NaN: Yeah…

JE: Hmm-mmm

NN: No, oh okay

JE: No, Bethel council, the Bethel tribe.

NaN: I think it's good because they've been trying to get people from communities it sounds like more community involvement along the river.

JE: Mmm-hmm.

NN: That actually knows what's happening.

NaN: That's what we know of them, you know, but I've never gone to a meeting or anything. Probably should.

JE: Yeah, that's great. Just wondering since...And do either have any worries or concerns about the salmon fishery today?

NaN: I do, because what if they..If they shut down certain time periods where you can't get any fish or there's only a limited amount of time you can fish and you don't get any fish, they wouldn't be, you know that would be hard cuz we really do enjoy eating it. We like having it and we can share it, you know.

JE: So the block openers just aren't really working out?

NaN: It seems short and then we worry at the beginning when are they going to open up?

NN: Yeah, and then you get our weather here, you know you get a lot of rain, water comes up, you know logs start coming down and there's another factor, that part, that you can't fish, and then they have closures. I don't know, like if they had closures after high water and stuff, you know, then you wouldn't miss out. It's just all plays a factor. I don't know.

JE: Yeah, the weather, the closures, if you are limited to that short time period. If anything's like not right in line…

NN: You gotta hit it just right. When the fish are coming and hopefully the waters not high and wipe your net out.

JE: Okay, so that's one of your concerns. Any other concerns that you guys have about salmon fisheries?

NaN: I don't know what the commercial fisheries, if they have any impact. I know that they should really consider the people that need to, for food, before they do alot of…

NN: Alot goes out, you know they sell it too. You know state law, I know Japan gets alot of it too though.

JE: So, they might be having an impact on the subsistence fisheries?

NN: I think it's the high seas fisheries. They come in, they don't care you know, they set a twenty, thirty mile long net and I seen on TV, you know, over, what was it, they've been taking out of the oceans, tons of nets coming out of the ocean. Ghost, they call em' ghost nets you know.

JE: Oh really?

NN: So they cut em' lose when that coast guard comes around and let it go you know so they don't get caught. You cut a net loose that long whose gonna say you were the one that did it, if you cut it lose. They have been catching or getting alot, fishing on the ocean...I just read it on the facebook, just the other day or today. Yesterday, you know ghost nets. Tons of it, miles of it.

NaN: That's a concern, yeah. Our population, all the people in the world, they're trying to catch all that fish and its gonna impact us. It already has.

NN: And that's been going on for a long time, when high seas fishermen, you know, its...I don't know, it's still going on.

NaN: Mmm-hmm

NN: That's why you get all these big glass balls in the ocean. Years ago when we used to fish there were Japanese floats you know, and then you'd see pieces of nets on the beaches and back then it's still going on today.

JE: Wow.

NN: You know. I don't know if there doing anything about it, but like I say you can't really catch em' if they cut their net loose.

JE: Yeap. Are there any concerns that you have about fisheries management in particular other than these you know, the limiting of fishers to these shorter periods of time when they can go out.

NaN: I can't think of anything else because I'm not really that...I don't feel like I'm really that knowledgeable on the management part of things. And uh, not even sure. I just know they keep count.

NN: Yeah.

NaN: They have weirs and they count the fish.

JE: Okay.

NaN: And I trust the uh, I trust them. I trust the process because we have to. We can't be greedy.

JE: Mmm-hmm.

NN: For future generations, anyway for them to have.

NaN: Mmm-hmm, just like right now our son, our grandson, he's really into hunting and fishing. He lives in Anchorage, but he was raised out here, but they moved to Anchorage and he texted me and said grandma, do you have any fish, any salmon filets? I really need it. And he's into his fitness, his body, he goes to the gym, he watches his diet. He was asking us for fish. Poor, we have no fish. {chucklin} So I was like I'll try to ask around to see if anyone has extra fish for you but I haven't really asked. But umm..you know when people have extra like this time of year they share it. Like people from Red Devil. Some people put up fish. I don't think Debby did this year, but if they have extra, they share, you know they share it.

JE: Okay, that's nice.

NaN: But I thought that was funny that its expensive to buy it in Anchorage. You know he doesn't want to buy it, he wanted to know if he had any filets.

JE: Ohh.

NaN: He's 20, going on 21.

JE: Wow, that's good he's health conscious.

NaN: Yeah, he really..he's the one that gets our moose every year. He's been hunting with his grandpa since he's been four years old.

JE: That's great, so he's carrying those subsistence traditions onwards.

NaN: Yeap.

JE: So you guys said what works well about the management is that they're doing the counting, they're monitoring, you already mentioned what doesn't work well. Are there any improvements you both would make?

NN: What's that?

NaN: Improvements in the management. In how they manage fisheries?

NN: I don't know. It's kinda like, live and learn as you go, so they make improvements as they...Like anything else in life, if you don't do something right, you try and make it better. You know, that's if it doesn't work. It's kind of like hit and miss deal too at times for everybody. You know, live and learn as you go through life.

NaN: And then the, whatever they could do about the off the river. Is there anything they can do about the ocean.

JE: The ocean.

NaN: Whatever's going on out there.

NN: They're trying to blame it on the killer whales, but I don't know. Did you see on TV not long ago that killer whales are killing all the fish?

JE: Yeah, huh.

NN: I mean they've been around too for how long and we never had...I think it's high seas. I really think it's the high seas. Yep.

JE: That's neat though that you knew about that cuz I just learned about that week. There was a Kuskokwim River Salmon Science Workshop and they brought someone from University of Washington and he was talking about the decline in the salmon size and other stuff and they said...one hypothesis was that killer whales might be the cause, so.

NN: No, I think they're trying to pass it on. It's the high seas really. They're the ones to be blamed.

JE: Mmm-hmm. Are there killer whales even out in the Bering Sea?

NN: Oh yeah, they go all over the place. Well yeah, they follow fish you know. Maybe it is part of the reason, I don't know. If whales were good to eat, maybe harvest them a little too I don't know. Instead of belugas you know. Harvest a few of them if there's getting to be too many. I don't know.

JE: Yeah, me either.

NN: Yeah, huh.

NaN: You don't eat killer whale?

NN: No, I've never heard of anyone eating killer whales. Have you?

JE: No, I heard the Tlingit people, when I lived in Juneau for a bit, they do not eat them because they're too, they're very intelligent. And I've had stories way back, I don't know if it was the commercial fishers, that they killed like one killer whale and a whole pod of killer whales came and took this boat down.

NN: Yeah, that's what my mom said, don't ever shoot at a killer whale because they will get you. That's what she always told me, but I don't know. When we used to fish she always said don't ever shoot a killer whale cuz the rest of them will get you.

NaN: Whoa.

NN: And I was always scared of that. When we fished out on the ocean, we used to see them out there though. Two, three miles off the shoreline they'd catch sea lions and seals out there and throw them up in the air you know. It used to be pretty neat.

JE: You'd see them get the sea lions and the seals?

NN: Oh yeah, yep. There used to be a rookery between where we fished and across, 10 miles across to the rookery called (Noosiallen) and you'd see em' out there between where we're at and catching and throwing them up in the air and the seagulls are diving down having fun too.

JE: Yeah, I heard the killer whale population is just on the increase. Probably cuz, I don't know. Who would kill them then?

NN: Then I see on the news the other day there's a pod down there in California are dying because there's no food down there. It was just on the news a couple days ago. I don't know, you know. It's pretty interesting. They're worried about them killer whales down states somewhere. They said there's no food for them.

NaN: So if there's no food down there, of course there going to come up and eat our food. So like you said it's the high seas, things are depleting in the oceans I think.

JJ: Hmm, okay. And what do you both want to see for salmon and your people in the next 40 years in this region?

NN: I'd like to see them have fish. You know, like our grandchildren and their kids, to have fish.

NaN: And know how to take care of it, be able to process it, and have smoked salmon, and be able to can and put it up and continue on with how we always have.

NN: For future generations, you know. Hopefully it'll happen you know.

JE: Anything else?

NaN: I can't think of anything.

NN: No.

JE: Quyana, thanks so much for sharing. I really appreciate your time. I'm gonna turn this off okay?

INTERVIEW PARTICIPANT: HELEN EVAN

Interview Date: 1/30/20
Interviewer: Janessa Esquible
Location: McGrath
Home community: McGrath

Shorthand key:
( ) Note about what is happening during the interview, unclear of word, or spelling question
[ ] Words not said but added for clarity
{ } Action
Mmm-hmm, uh-huh are affirmative
Hmm-mmm, uh-uh are negative

JE: Okay, all set with the paperwork. Did you want to keep this for your record?

HE: Okay

JE: Okay. Helen Evan. and Helen is McGrath your home community?

HE: Yes.

JE: What is your cultural affiliation?

HE: What is that?

JE: Athabascan, Yup'ik?

HE: I, I, I'm Athabascan

JE: K. And do you care to share your age?

HE: Yeah, I'm 71.

JE: K. Can you tell me a bit about your background, where you're from, how did you grow up and what connections do you have to salmon?

HE: I don't have that much connections to salmon because we don't usually catch salmon up here. Last couple years is when I caught like 30 salmon in a week. And what did I do with those salmon? Oh, I freeze them in my freezer. I make some half-smoke, and then for the first time I, me and this young boy made some salmon strips.

JE: Yum

HE: And it turned pretty good.

JE: Okay, did you, when you were growing up did you go fishing?

HE: I was born at Deacon's landing which is about 65 miles downriver and we live subsistence all my life that I remember my grandparents and my mom and dad. I think they were catching a lot of big salmons. I was pretty small back then but I think were was lots of big king salmons. Maybe I'm little that's why I look so big but they were big and silver.

JE: Wow.

HE: And he usually start catching down there June fifth at Deacon's landing.

JE: Wow.

HE: I have never tried after he died so I really don't know how to fish with a net {chuckling}

JE: When you were younger you remember seeing them really big?

HE: Yeah, really big. Big and silver. Grandma would cut em' and smoke em' up and make what they call blanket fish. I don't know why they called it blanket. Maybe because it was so big.

JE: Oh really? Ok.

HE: Mmm-hmm. And then after that I think we were catching silvers.

JE: Silvers...too?

HE: Uh-huh, lots of silvers and dog salmons and…

JE: Ok

HE: Until whitefishing in the fall.

JE: Does it..ummm..is it like any different like when you were young thinking about the salmon you got then, is it any different than today?

HE: Yeah, I think they were better back then. Uh, I don't know, seem like last year I tried fishing in second week in June and the fish weren't that good. They were soft, they were old and I fished the same time I fished the year before so..I could tell a big difference on those fish. Like..

JE: Hmm, just even recently?

HE: Yeah, like they got old before they got up here. Like, before they were nice and silver on these dates that I caught em.

JE: Oh

HE: But not this last year, so I didn't fish.

JE: You decided because of the quality was just...

HE: Yeah, I didn't like the taste.

JE: Oh really?

HE: Yeah it was old fish.

JE: How about the other fish? The chum and the silvers? Were they..is it similar?

HE: I didn't get any silvers because these places where I was fishing before are, there creeks that I fished for silver before. Well, they're dying. The water is not clear water like

it used to be when I fished there before. It's dead waters, its brown, ugly water. The fish I got out of there were like sad and weak.

JE: Mmm.

HE: Even though that's where I fish at the, you know, you fish same time every year. And it was really weird and then there's this one lake where I used to go to, to catch pikes. Well that lake is totally dead. It's a big huge lake. Vinasale lake. Me and my girls went moose hunting and we drove in there and we start smelling something bad and the water...usually it's clear water. It was just muddy; you can't see nothing. We got in the lake and it just smelled awful. Everything was dead.

JE: Wow

HE: And my motor hit the bottom and it just hit that mud and that awful bad smell came up from there. We got out of that lake so fast we were so scared. {chuckling}

JE: Oh my goodness.

HE: Yeah

JE: What did it smell like?

HE: Something rotten or awful like toxics or something. I don't know.

JE: Yikes.

HE: Yeah.

JE: So the fishing has changed a lot since you were younger?

HE: Lots, I didn't get any fish.

JE: And even the creek, so not like not only the fish the quality is different but also the bodies of water are different.

HE: Dying or something.

JE: Wow.

HE: Uh-huh. And like oh my woodpeckers here.

JE: So beautiful

HE: It's so huge.

JE: Wow.

HE: Anyways, there's this stuff growing on the edge of the river. It's all along the Takotna river. It's some kind of weed. It's really long when you pull it. I notice it's like ten feet out from the beach growing all along the river. And I never seen that before. Some kind of big weed growing up from the bottom of the river along the edge. It's warming up so fast.

JE: Really, and that's new?

HE: That's new.

JE: It's just not from here, the plant?

HE: I don't know. My friend from the homestead Takotna River the one that told me about it. She said Helen have you seen watched the river along the edge. We looked and sure enough this plant is so long we were trying to pull it but it's really hard.

JE: Really, it's really rooted in there?

HE: Yeah {chuckling}

JE: Wow. Do you think its..you said something about warming? Is it…

HE: I think it has lots to do with climate warming.

JE: Warmer climate and like this plant likes to grow in the warmer weather or something.

HE: And I notice a lot of dead fish along the river when I stop and on the beach. There would be a dead old fish somewhere.

JE: You're seeing more dead fish along the river, along the riverbank there?

HE: Mmm-hmm. Not that many, but I've never seen them before.

JE: Really?

HE: Uh-huh.

JE: And, for when...any teachings and rules that you were taught when you were younger about like caring for salmon? Are those still followed today?

HE: I follow em. Mmm-hmm. I don't know any other way to take care of salmon.

JE: And, the rules of like salmon fishing, all that stuff, pretty similar too?

HE: We used fish wheels when I was young but now I don't think anyone knows how to make real fish wheels.

JE: Really?

HE: Yeah, the one they make around here falls apart all the time. {chuckling}

JE: Oh no. And when you were younger, they were like really good, the fish wheels?

HE: Yeah, my sisters and I used to build them. They taught us how and then we did it wrong they let us take it apart and redo it so we got pretty good at it.

JE: Wow. That's pretty neat.

HE: He'd give us instructions and and you know every time we'd quit following his instructions {chuckling}

JE: So you guys made the best fish wheels? And now they're falling apart? Jeeze.

HE: I think so.

JE: Do you know your ancestors, how they were like salmon stewards...you know how today we have like fish and game, fish and wildlife, these other entities that are like

managing the salmon, how do you think your ancestors did this before there were was like state and federal management.

HE: I don't know. I guess they know how much fish to get and then they all quit. They don't just kill fish for nothing. They just get what they want and that's it and wait for next group of fish. Nothing used to get wasted that I know of. But now I see a lot of waste.

JE: In the community?

HE: Yes.

JE: What do you think that's from?

HE: I don't want to answer that {chuckling}

JE: Okay. Do you feel that your knowledge, values or you know the knowledge and values, Athabascan knowledge and values are incorporated into salmon management today?

HE: I have no idea.

JE: Do you have any concerns about your salmon fisheries today other than what you've mentioned earlier like more dead fish, different plants growing?

HE: I'm not sure if I want to fish next year after what I seen this last summer.

JE: Because the fish were just kind of sick? Sickly?

HE: Yeah, sickly fish and waste of time. But that's not gonna stop me from checking them out of course. {chuckling}

JE: Are there any concerns that you have about the way fish are being managed today? Or like anything about when you can go fishing, where you can go, regulations about gear that you can use?

HE: I don't know if we have any regulations around here. Nobody tells me when to set net. They don't even check on me but they tell me all I have to do is put my name on my floater.

JE: That's it?

HE: That's it?

JE: So you can go whenever?

HE: I guess. I don't know. I have no idea. I know some people get busted up around Nicholai.

JE: Ok. Is there anything you think that works very well, that could work really well with managing a fishery to keep them healthy? Or maybe that doesn't work well?

HE: No idea.

JE: Would you make any improvements to how things are being done now to take care of the fish or manage them?

HE: No.

JE: And, what do you want to see for salmon and people in your region in the next forty years?

HE: I have no idea. Better fishing I guess.

JE: Better fishing?

HE: Yeah.

JE: Anything else that you want to share?

HE: No.

JE: That’s it. Thank you so much for your time, and your knowledge and wisdom.

INTERVIEW PARTICIPANT: ARNOLD ANDREWS, DARA AND KEVIN WHITWORTH
Interview Date: 1/30/20
Interviewer: Janessa Esquible
Location: McGrath
Home community: McGrath

Janessa Esquible [JE]: State your name and home community, your cultural affiliation, and your age.

Arnold Andrews [AA]: Arnold Andrew, and I’m 65 {laughs} um, what else?

JE: Is your home community McGrath?

AA: McGrath yeah.

JE: And what's your cultural affiliation? Are you Athabaskan?

AA: Athabaskan yeah.

JE: And that's it.

Dara Whitworth [DW]: Dara Whitworth. My home community is McGrath. And my cultural affiliation is kinda a mix but it be uhh…

AA: Scandinavian?

DW: Yeah. Scandinavian is probably the way I identify mostly.

JE: Alright.

AA: And what's your age?

DW: Oh and 36. Recent birthday {chuckles}

AA: Me too. D-A-R-A.

Kevin Whitworth [KW]: um, Kevin Whitworth from McGrath. Athabaskan and I am 39. Turning 40 this year.

DW: Cultural affiliation?

JE: And how about these young ones who are present with us?

KW: What’s your name?

DW: Can you say your name?

Rohn Whitworth [RW]: 5.

DW: Your names not 5. {laughs} What's your name?

RW: Rohn.

DW: Yup.

JE: Okay.

KW: Where you from?

RW: Um.

KW: McGrath?

RW: McGrath.

DW: And your (viking) Athabaskan right? {laughs}

RW: Yeah.

KW: Okay your turn Linnea.

DW: Can you say ‘I’m Linnea’ {laughs}

KW: What's your name?

DW: Okay you're Linnea. How old are you Linnea? How many fingers? Say it louder.

Linnea Whitworth [LW]: 3

DW: 3? {laughs}

JE: Wow. And is that L-O-A?

DW: L-I-N-N-E-A

JE: L- for her. And then..

DW: And he's R-O-H-N.

JE: Okay so R-O-H-N. L-I-N-N-E-A.

DW: N-N-E-A.

JE: Okay thanks. And today's January 30th. And I'm Janessa Esquible. Um, I'm Ojibwe and Mexican and I'm 29. That's about me. Let's see. (Continues to go over project forms). So first question, if you could tell me a bit about your background, where you're from, how you grew up, and what are your connections to salmon?

KW: Want me to start? (Mhmm). Uhh, okay I'll start. Should I introduce myself while recording? So this is Kevin, uhhh, grew up here in McGrath.

JE: Oh, uh, where you grew up, or so you're from McGrath, how did you grow up, what connections do you have to salmon. And just a bit about your background.

KW: Okay so, grew up in McGrath. Grew up, in small community, you know tight family, subsistence lifestyle, lived off the land a lot and out in the woods a lot. Being taught by aunts and uncles, grandmas and grandpas, and community members. Spent a lot of time harvesting, hunting natural resources especially salmon in the summer and fished subsistence, ate fish and salmon. I'd say a lot of my base knowledge for salmon came from the early days of fishing.

JE: So all your life?

KW: Well yeah still today, you know, fish uh every summer bring the family out there and trying to help my uncles whenever I can.

JE: Thank you.

DW: This is Dara. I grew up in Homer, Alaska. Born and raised. Part of a multi-generational commercial fishing family. My grandpa was a fisherman for salmon. My mom and dad, my brothers and I, my uncles, all fished for salmon. Um, my dad also fishes other species like crab and cod and halibut. So fishing was our livelihood. {chuckles} Um, fishing is still a big part of our life. We get out of seafood from my parents and as Kevin

mentioned we spent a lot of time on the water in the summer and the fall. Sometimes in the winter. {Chuckles}. Ice fishing. Yeah, we were always around the water growing up. Grew up on boats mainly.

JE: Thank you.

AA: I'm Arnold and was born in Holy Cross on the Yukon. We'd go over to McGrath. Maybe 6 or 7. We always catch salmon. Subsistence.

JE: Thanks. And could you tell us about salmon fishing when you were young and how that might be different to what it's like today?

AA: When we were young, long time ago, (Inaudible). We were always in Holy Cross when I was younger and my dad used to fish (inaudible). There used to be bigger salmon those days. I mean, real big. Nowadays they're smaller.

JE: Mmk, so smaller fish.

AA: Yeah.

DW: Um, what was the question again?

JE: Oh, uh, like how fishing was when you were young. And then if it's any different today.

DW: Uhm. Being still fairly young, there hasn't been a lot of (unknown) since I started fishing till now but quotas for my family has definitely gone down over the years to the point where some species are, um, they're thinking of not even having a fishery for that species like cod, and king crab. Salmon we were a part of a hatchery fishery. So when I was younger the hatchery on (Calgan) Island closed down. So we relied more on wild stocks and that was definitely a change from hatchery fish versus wild stock.

KW: This is Kevin. So uh, when I was younger growing up here in McGrath. It seemed like we traveled more when we were fishing. Going up to Salmon River every year. Going up upriver or downriver. Not really far downriver but fishing up the Takotna. Seems like now we don't travel as much. We just try to stay a little local. When in the past ten years I went to the Salmon River maybe once. Just expensive travel. Business of life so it's hard to get out there but we try to get out there as much as we can. But then also uh, growing up it seemed like there was maybe more fish, but I was young then too so it's hard to realize or think about that. But never, we never really had restrictions like we have now. We've had seasons where they are shut down. And when I was young, we never, you never talk (inaudible) you never had to deal with that. It was just always open. Now we are wondering if there's going to be openers or closures. It's really hard. And it's like that for the whole river but upriver too. And local people upriver always talk about, uh, the fishery down river. How it's going to affect the upriver folks. And affect the fish up here. You know when I was younger that wasn't really something we thought about but when time went on the elders started saying stuff like, you know, there's a lot less fish than

there used to be. So that's always in my mind. There's a lot of things that are different from when I was younger but, mostly that..

JE: So when you were younger there weren't as many concerns about what the folks downriver were catching and if or how that might impact people up here?

KW: Well I wouldn't say, it's just something that I never thought about when I was young.

JE: Gotcha.

KW: But elders talked about it then.

JE: They did?

KW: Yeah. Maybe Arnold could touch on that. But um, talking about like folks like Ray (Collins). Who talks about (unknown). Those guys talk about the early season runs and um, (unknown) but then. Yeah there's all sorts of details you can talk to there but like so when I used to go to the Salmon River. Me and (unknown) used to talk about times a long time ago. When she was a little kid and the Salmon River would just be jammed packed with fish. Bank to bank would be red, solid. And she said it's like those Bristol Bay images where you see the sockeye fill those little creeks. She said that's what Salmon River used to look like. So those images are in my mind. (Unknown) told me about that. And I've asked about just a few years ago I asked the family do you remember (Lena’s) story of the river being jammed with king salmon. And they'd say yeah we remember those stories.

JE: Mmk.

KW: So I mean so much we left. Knowledge as a young kid. You learn.

JE: Anything else on that one? No, okay. And how did you learn to manage, steward, or these are like words can be used interchangeably, managed, steward, or cared for your salmon when you were growing up?

KW: And are you talking about preserve. Take care of the food source. Or are you thinking of individually how does family manage their fish-, um, not overfishing or underfishing?

JE: Yeah kinda all of that. Some responses that we received are you know fishing to take what you need. Taking care of the fish and um, like even like on your boat having everything clean or tidy, or you know different like that. That people say these are the values that we, like that are embedded in us and how we care for salmon.

KW: Well, this is Kevin, I’ll start. There’s um, lets see, where I started.

JE: Did you learn to manage, steward, or care for salmon when you were growing up?

KW: So. Processing fish when we first caught it, I always watch my grandma and grandpa at fish camp. And always there was a process there that uh, grandpa would go check the net, bring the fish up to fish camp. Grandma would clean it. And then there was always a process to get it into the smokehouse. And all under steps. You'd watch and even smoking it. Gathering the wood, making sure the temp and everything is right in the smokehouse and there's no bugs. It was constant. That's preserving but then management of fish growing up, going up Salmon River lot of those things up there even today, even back then, they never went for the females, they always released the females. So it was a local way of trying to manage their fishery. They're not, they are trying to keep those females in the way so they can spawn and try to recoup the population. And uh, even today, Nicholai folks tell ya "let those females go (unknown)" Yeah they'll see it in the river and get that male right there. So there's a local effort to manage their own fishery. There's a lot of detail getting into that but I'll leave it at that.

JE: Okay thank you. Anyone else want to share? Or else we'll move to the next question.

AA: Yeah I always keep my fish clean. Make sure it's fresh and when I smoke it, I use alders and I cut up my fish myself. I do all the processing all by myself. So. And I turned out good. Mmhmm.

KW: You told me stories of uh, grandpa where he used an assembly line of all the kids.

AA: Oh yeah.

KW: At fishcamp.

AA: Yeah

KW: One kid would have a job of cutting the head off.

AA: Yup. Yup.

KW: You move it down the line.

AA: Big family. {chuckles} 13 of us.

JE: Wow.

AA: Yeah.

KW: Next kid would have a job of gutting it and move it down the line and next kid would have a job of you know, fillet or whatever. It was certain jobs. 13 or 14 kids at fish camp. All of them had jobs.

JE: So everybody would help out.

AA: Mhmm.

KW: And Arnold's nickname was (grayjay) camp robber. {chuckles}

JE: Really? {chuckles}

AA: You told me. (inaudible, laughing in background). “Who’s that camp robber up there!” {laughs} And that was me!

JE: Really?

AA: (inaudible) had to go up to get the real dry ones.

JE: Ohh.

AA: Had a two-story fish smokehouse.

JE: Oh okay.

AA: Yeah and I had to go way up there to get the really dry ones {laughs}

JE: Gray Jay.

AA: “Who's the camp robber up there!” {laughs}

JE: Thats funny.

KW: Thats always an image that sticks to my mind because of the size of the smokehouse. Arnolds dad, uh my grandfather on my mom’s side had a smokehouse like I don't even know.

AA: Yeah he, my aunt and my grandpa had three houses.

DW: Wow.

AA: Yeah.

JE: Mmm.

KW: Plus he had his own dog team.

AA: (inaudible) (had to feed his dog team).

KW: Two stories. Smokehouse like as big as this house huh?

AA: Yeah, like this big.

JE: I’ve never seen a smokehouse that big.

AA: And it was full. Three tiers.

JE: That's incredible. Wow. Well, are any um, the teachings or rules that you were taught in the past and like how to care for salmon are those still being followed today?

DW: Mhm.

KW: Yeah especially keeping your fish clean and preserving it and not wasting it. Yeah. That's always number one. Never waste a fish or uh, you know, disrespect the fish or carcasses and stuff. Always trying to use every single piece of it. Even there's rules around here with moose bones, and moose eyes. You don't want to just throw it in the dump. You know, same thing with fish. There's lots of things you wanting to respect for the fish. Give back to the river if you have guts throw it back in the river. You just don't throw it on the road or at the dump. A lot of people here they even put fish hearts and carcasses and stuff in their garden. Produces more down the line.

AA: Blood for my garden.

DW: Did you ever do that growing up? Did you ever put fish in your garden growing up?

AA: No but I heard of it.

DW: Yeah.

AA: That's why. {laughs}

DW: I was curious with that big garden in Holy Cross that they used anything like that.

AA: I don't know.

JE: Will have to try add fish blood.

KW: Yeah it works good. Really good.

JE: You just put it in the dirt?

DW: Even a whole fish works great.

JE: Wow.

KW: Yeah so what we do, you get turnips like this.. (inaudible)

JE: Wow. That’s awesome.

KW: We dig trenches in our garden and bury uh, fish in there.

DW: Whole thing.

KW: Yeah. You know the Cherokee Indians have a system too. When they are planting corn, they add (unknown)

DW: Mhmm.

KW: So they're planting corn. Would you remember?

DW: It was squash, corn, and peas I think or something.

KW: They did the whole and throw fish down and put their seeds in and then start mound it.

JE: Huh. Using fish too

KW: Using fish. Good.

JE: How did your ancestors steward or care for salmon?

AA: Um, they cared for it. They preserve it and smoke it. And jar.

JE: Do you feel like your knowledge or values are reflected in fisheries management? Like your Athabaskan or Scandinavian values?

KW: Can you go back to the other question? The previous one.

JE: Oh yeah. How did your ancestors manage, steward, or care for salmon?

KW: I'd like to comment on that one.

JE: Okay.

KW: Maybe they have some too but. Um, so a long time ago. There wasn't, the fishery was way different than it is today. So our ancestors you know they didn't have the long nets. They didn't drift. There wasn't even fish wheels way back. Fish wheels came from the French I think. French fur traders. That technology came from them. Ray Collins talked about, the folks in Nikolai up in Salmon River they had a weir and fish trap. They would set every year and catch what they needed for the families. Because family groups were smaller. Population was a way bit smaller. They caught what they needed and lot of times they were feeding multiple families. Like Arnold talks about my grandpa feeding three families. And the young guys too were catching with their weirs but it wasn't a terminal fishery. Like salmon (pitka fork). They were fishing the tributaries. And they were

fishing the side channels. And they weren't fishing the main stem because they didn't have the technology to fish the main stem. Maybe with uh, I don't even know if the dip nets were around this country. Maybe in one village (inaudible) dip nets but then it was the Stoney River. So, by fishing the terminal areas, the tributaries, the small creeks, and leaving the (corridor), or the main stem open. It provided a free passage for fish to get up into the tributaries and spawn and keep regenerating and keep the population going. But everybody was doing this (inaudible) Kwethluk. They had traps. They fished the tributaries. They fished off the main stem. Later on, set nets came along. Fish wheels came along. But those were all bank oriented. So a lot of the fish bank oriented were getting caught before they go up in the Kwethluk. They get caught in the wheel; they get caught in the set net. But today, we have drifting. Which is, its uh fishing the main run. Right in the middle of the river. Which is really hard for fish to escape. So its uh, a big change were seeing and Ray Collins, he’s the one who really taught me this knowledge, how its changed and some of the issues were dealing with today. Back then it was a long time ago it was way different. It was easier to manage. I think it was better for the fishery. There's a lot of you know, these questions are really good questions about there and you could almost, each question you could talk about for a long time. Good questions.

JE: Yeah feel free to, if you guys want to share any more you can.

KW: And those, I don't think you could, it would be very hard to overfish a population if you're just fishing the terminal fisheries. Plus, when you're fishing the terminal fisheries, well the tributaries, shoot I call it terminal fisheries, but probably other names but what you're finding is these family groups of fish that terminal the small creeks, they uh, they're closer tied to that creek, they know it really well. They can see uh, looks like there's very few fish coming back, probably not going to be able to catch as many as we did last year or they're really tied to that management of that creek. So, they aren't going to want to overfish it. Cause they know next its going to hurt them so they self-manage those areas. Today, when we are in the bigger communities you know like Bethel. Even McGrath is a big community for their area. The fisheries are concentrated in spots that are hard to uh, get stakeholders to buy into the fisheries as much as they used to be long time ago. That's my point. I think we need to switch back to uh, getting the stakeholders more invested in the management and more intimately (unknown) of fishery because they are the ones that fish, ones that will manage it in the future. So that’s the best scenario for the whole fishery. I mean I could, this is uh, a very neat topic for me. Good topic. Yeah.

DW: Like commercial fisherman. You're tied to so many different things. A lot like farmers. Um. You have not only the specific population you're harvesting. For instance, when your harvesting salmon, the population changes each year as far as who comes in the river system. But you're also dependent on management like the state or feds whoever’s managing that fishery. Um. They're setting the quotas, they're setting the length of time you fish you know; you are very regulated. As commercial fisherman. I mean as you should be. Commercial fishing is one of those things that’s so efficient now that it easily can easily disrupt the system. Especially when you're catching populations out in the ocean where you don't know what river system they're going to. Or the origin of those

species so. Um. As far as how much has changed. Was that what the question was. Oh how did the ancestors manage?

JE: Yeah. We will get to, let's see, the next couple questions if you feel your values are being reflected in fisheries management. Concerns you might have. So on. Yeah. Anything else or?

AA: I think about when they fish on the ocean, then they get those big boats. They have long nets. It seems like they should let them byy before they.. Cause they catch, yeah, tons and tons of fish you know.

DW: For sure.

JE: So you think you're just a little concerned about commercial fishing?

AA: Yeah fishing for up here yeah.

JE: Yeah. Okay.

KW: We've had people come in and intercept fishing, that's what you're referring to?

AA: Yeah

KW: Yeah, so there's people (unknown) false pass and pollock fishery. The false pass is the bottleneck and I guess I don't really know false pass pretty much but uh some local folks they do have that old history yeah that's where this intercept happens. Fish coming up into the rivers.

JE: Do you feel like your knowledge, your values, wisdom, are reflected in fisheries management today?

KW: No. I'd say not completely. (unknown)

JE: Yeah like some of these values like you were talking about how you grew up and how you took care of the salmon. Those types of things are the way that they fished. Maybe like the terminal fisheries. Things like that. If management takes any of that into consideration. In managing the, you know, the system accordingly.

KW: Yeah that's hard, uh I would say that uh, some of my knowledge and values aren't being reflected but it would be very hard to have them all reflected. Times have changed. Just technology of fishing changed. And population size (unknown) changed. Ocean environments changing. So much has changed I don't think a lot of my values and truth and knowledge could all be reflected but there's some things that could happen. I can't think of them right now.

DW: Years of restrictions and stuff.

KW: Yeah and (inaudible)

JE; And there's some, and there's a question later we'll ask you specifically what might work well right now, what does not work well and potential improvements that you might have.

KW: Okay.

JE: Do you feel, I mean would you (unknown) growing up to now are your values and knowledge reflected in the way that fisheries is being managed?

DW: No. I think often fishing, fisheries management is um, higher up in the um, the decision is often political or money driven. Um. I think that's not the most effective way manage a population when those are the two factors determining how I mean, determining everything from commercial fishing openers to subsistence openers to, uh, how many salmon we let past, a lot of that is political..

JE: Okay thanks for your perspective.

KW: Yeah our technology (unknown) political system. Right?

DW: Right.

KW: So that's a very good point. Yeah.

JE: Yeah that's excellent. And the next one is what concerns or worries do you all have about fisheries today? Global warming?

AA: yeah. (inaudible)

KW: Yeah, this summer I mean there was one day Uncle (unknown) and I were out fishing this summer. We pulled our net because it was so too hot, remember that day?

AA: Mhmm.

KW: The fish was like jello.

JE: Wow.

KW: It was not always uh, warm temperatures in the waters are hard on the fish. And it's hard on the people too. Like the resource itself. Uhh, it's hard to want to eat a jello fish. It's not as hard, it's not as good.

AA: Bloating too.

KW: Bloating and..

DW: Warm.

KW: Yeah. You have to check. Very very often. You know McGrath is a set net community we don't drift.

JE: Mhmm.

KW: And even overnight you think it's going to be cold enough through the night but then you go and check your morning net and it wasn't cold enough. Fish start spoiling. That quick.

JE: Wow.

KW: And then you start checking two three hours later and again so fast. They start spoiling. So we just pull our net. Like this is not worth it.

JE: Wow. Does that happen just over the past couple years or no it's been going on for longer?

KW: That's more recent I'd say.

DW: Yeah.

JE: Where you can't leave your nets overnight anymore.

DW: Especially last summer. I mean the heat last summer was um, maybe even unprecedented for hot it was for how long in McGrath.

KW: We've had summers, a few summers ago we had a lot of 90-degree weather. And we had fire years. It was really hot and made lots of fire. There was uh, we didn't fish we were busy fighting fires. But I bet that summer was pretty bad. And it's scary too because these tributaries are warm. And as the water goes downstream it's not going to cool off unless there's a big rain event. So it continues to warm up (unknown). So even if there's, in the spawning grounds warm, all of the migratory path is warm.

AA: That is a big concern of mine too. Change in environment. Overfishing is one of my biggest concerns.

JE: Overfishing?

AA: Overfishing. Not just in river, in the ocean, these fisheries need to be managed. Gravel to gravel. This is something I learned. What I mean by that is when you're fishing more to when it goes out to the ocean and then it comes back and dies in the gravel again. Every step of that fish life cycle needs to be managed. So that it can improve

fishery for the people and for the fish like its (inaudible) off-shore. It’s a different management (unknown) It’s very hard to uh, push and pull you know more specific management transports and (unknown). It’s hard. But that’s a worry of mine. Uh. Anything else.

DW: My family wonders about the longevity of commercial fish as your primary source of income. My older brother works for my dad with the intention of them truly taking over his boat. But I know my mom and dad both wonder how it will look in 10 or 20 years. If they'll have that as a livelihood or not.

KW: Yeah, it's not always hard on commercial fishermen it's hard on subsistence fishermen as well. It's all across the board.

JE: All fisheries.

KW: Yeah. So that's…

JE: Do you have any.. So you kinda touched on this, I think all of you have. Um, about your concerns with fisheries management today then not managing from gravel to gravel. Maybe it not being sustainable and politically or I guess financially driven or driven by money. Do you have any concerns with fisheries management?

DW: Seems like trends when you look at the history of fisheries management from Europe, East coast, Pacific, Alaska. It's not a very good, um, trend. {chuckles}. Most of them are trying to rebuild stocks that have been decimated and that's very thought thinking. Are, is Alaska heading that way? Are we going to be just like Europe, the East Coast and the West Coast, and other places throughout the world or, um, are can we actually manage in a way that is um, is going to maintain those viable populations so.

JE: Mhmm. And (unknown) I have a couple ones if you guys want you can (inaudible) and the next question is.. Well talks about in the past, so yours were hopefully get into what you all think, if any, works well with fisheries management. What doesn’t work well and if you have any suggestions for improvement.

KW: I got one suggestion. But like touch on. So I touched on a little bit. And that is uh, I really think that the fisheries need fisheries management, needs to be in the hands of the people. Stakeholders. Cause the stakeholders uh, if they're not integrating them into the whole system, then you're not going to have buy in. So uh, once you get buy in from stakeholders, and the stakeholders have to be involved in management for it to work but, then you have a better chance of conservation, rebuilding, uh, people being intimately involved. Which is better all around. That means like even to the point where villages and village councils and villages are maybe someday 50 years from now, they are the ones that are going to have advantage in their own little creek that they live on.

JE: Mmm.

KW: So that the management doesn’t come top down. Like Fish and Game, Fish and Wildlife. But comes from the people.

JE: Mhm.

KW: If that makes sense.

JE: Mhm.

KW: I think that's what's best.

JE: Okay.

KW: Cause when it’s top down its, there's a lot of native people been managed for a long time. With schools back then. And uh, colonization and that's sort of stuff. So when you got, they still have a heavy hand in management today and there’s a lot of generational uh, you know, negativity with that. And for that to go away, we got to repress that people. So there’s a lot to it. But getting the people involved is the best.

JE: Thank you.

DW: Um. I also think there's a tendency with managers to kind of of ignore warning signs in favor of saying. For example, I'll use an example. Um, the coast there’s a really healthy pink population, pink salmon. Because it’s been um, hatchery fish that have fed that population for years and years and they pumped more and more pinks into the environment. So, managers well say, ‘look! Look we have this really successful pink salmon population!’ You know. ‘Billions of salmon, look how successful we are’ Yet you have king salmon um, populations taking a nose dive and yeah it’s not being ignored but it’s kind of look ‘let’s just focus on these pink salmon’ which you know, any time you start messing with um, the balance of like you put a whole bunch of pink salmon who are short lived and are great for human consumption but might not be great for the environment any time you start putting that into the system um, and ignoring the way it might affect all of the other salmon species then I think you’re doing something wrong.

JE: Mhm. So do you think that part of the problem with fisheries management is that there’s like a single species management deal instead of like managing for all of the species.

DW: I think there’s a um, a product for focus. Managers want to be able to say ‘hey we have, you know, salable product coming from Alaska's fisheries’ and there not necessarily ignoring the ecosystem but in a way they kind of are because you’re putting the focus on one species like pink salmon and um making that into a billion-dollar industry. While ignoring other species like King salmon which tend to be a more of a subsistence fish that people rely on the rivers. Theres an inequity I think in the way species are managed and the focus that’s put on them. Like pollock, pink salmon. How that in some ways um…

KW: Yeah the other thing too is you bring up a (unknown) we talked about when your managing gravel to gravel like I talked about. You're not only looking at the fish. And lot of fisheries management today is your looking at single species like Dara's talking about. But you need to look at when you're managing gravel to gravel. You're looking at the water, like Arnold talks about climate change. You're looking at the environment and you're looking at the ecosystem. Everything that affects that fish, the people, whatever that is, has to be involved and in place and part of the management. Land. So even uh, Fish and Wildlife and BLM they do this management plans. Land management plans. Fish has to be involved. So it's, you know, you can't just look at the species. You gotta be ecosystem, gotta be broad. And uh, all incorporated so, then you know, we feel like we have history too that shows like agriculture. When you do it monocultures, it fails. Look at the dust bowl. We have history that shows that these sorts of things fail. And when you have a pink salmon it's like a monoculture. At some point it's going to fail. We already see it starting to hurt other species. You know it's not good for the soil. Until we saw the dust bowl. But.

JE: Yeah.

KW: Its uh, we should write a book {chuckles}

JE: Do it! Include the interview too! (inaudible) Do you guys feel like there are any strengths in the salmon management system? In the Kuskokwim that you'd like to highlight? Anything that's working right?

DW: Well, it's interesting that the red salmon seems to be increasing on the Kuskokwim. And I don't know if that's because of management or just environmental change. But it's always nice to see um, a species that's thriving and even increasing. Um. Yeah. I know there's a lot of knowledgeable people managing salmon on the river and um, a lot of times management isn't constrained by will but by the amount of money put towards management. So biologists do a lot of work with a really limited budget. So. I definitely commend them for all the work they do for, with the money they have. But there's room for improvement.

KW: One thing that's working, helping maybe's helping, but one thing that's past few years is buy in river wide conservation and rebuilding king salmon. And that's a really good successful story. I mean your showing, people from the Kuskokwim want to forego harvest. For conservation, rebuild. Cause everybody remembers all the stories of really big salmon. Lots of salmon. And they'd like to get there someday but they know that they have to make sacrifices today for the future. So there's, and you know, you go to Nikolai, Salmon River and these young kids and all the elders they release all of their females. There's a little conservation effort. You see that in (Teslan) so the (Teslan) tribe they up in the Yukon, their tribe actually made a resolution to stop fishing King salmon just to conserve.

JE: Wow.

KW: To try to rebuild. They told all of their members they're not going to fish King salmon. That's success story you know. And uh, anyway.

JE: Thank you.

AA: (inaudible) ain't got no salmon {laughs} They got to uh.

KW: They're on the road so they go to..

DW: They're in Canada right?

KW: Yeah they go to their neighbors, you know towards the coast. And fish but. King salmon they don't.

JE: Wow.

KW: Yeah.

JE: Did you have anything that you wanted to add? No, okay. Well, I think we're on the last question then. What do you want to see for salmon and your people in 40 years?

KW: Well one thing it will be nice to see in 40 years is uh, rebounded king salmon and maybe some of those old 7,8-year-old king salmon that come back. Big salmon.

AA: Big salmon yeah.

KW: Yup big salmon.

AA: Yeah.

KW: Um, you're trying to fill your freezer or smokehouse, those bunch of little salmon just doesn't add up to a few big salmon. It’s important to uh, and also, in the next 40 years um, the less restrictions. If we can rebuild, were not going to have these restrictions. These restrictions are hard when your stop people from fishing because they're not going to fish camp, their culture starting to get you know less and less, fishing with little kids and uh, Bethel region you're seeing fish camps that haven’t fished in a long time you know. Thats hard on that generation of fisherman. And if you don't have that new generation fishing, they're not going to learn. Not going to learn to fish but they're not going to learn the resource and they're not going to be invested as much. As if they were fishing. Learning about the fishery.

JE: So having, wanting the younger generations to still stay.

KW: yup

JE: Involved in fishing. A new interviewee that joins us. {laughs}

DW: Um. Id like to see my kids fishing. Um, family fish, which of right now, two openers a week so.

AA: Two openers. Thats crazy.

DW: I know. Sorry (inaudible) two openers but. Help pay for college at least.

JE: Yeah.

DW: And I hope my brother will be fishing. For my dad if that's what he wants to do.

KW: The other thing I want to see is um, more stakeholder involvement in fish management, and science. Uhh. scientists don't need to come from, uh, Anchorage.

DW: Homer? {laughs}

KW: They can do it here. Yeah. We can do it. And same with the management. We’ve done it for thousand years we just have to teach these folks that reteach these folks. But we can do it. So it’s interesting. But anyway.

JE: Thank you.

DW: I want to see healthy 5 species of salmon too.

JE: Mmk.

DW: And harvest not only kings but if you're in the right spot on the river, reds and silvers and {laughs}

JE: Okay. But at least for the region.

DW: Yeah.

JE: Seeing all the 5 species. Yeah I’m with you there.

DW: Kev didn't we catch like one red up here? Didn't we catch one red this year?

KW: No we caught like 4,5.

DW: Up here.

JE: Were they in good shape?

DW: Uhh, they were decent. For this high up river.

KW: We caught four or five but we ate one.

DW: Yeah we ate one. I think the other ones were too mushy.

KW: They're coming around.

JE: Huh. More reds.

KW: My neighbor said ‘don't tell anybody’ {laughs}

KW: I never see any. Do you catch any in between king salmon and let’s see chum. I can’t remember. But when you’re fishing for, when your fishing for chum, that’s when you’ll catch them And there’s more and more.

DW: Yeah, when was it August? September?

KW: No. It was earlier.

DW: Was it?

KW: But we caught them in September too but those ones were jello.

DW: Oh that’s right.

JE: Wow you got those in September.

KW: Yeah.

JE: We usually get them in July. Sometimes in early August but.

DW: I know on the coast we get them like June. June July.

JE: Okay.

KW: So maybe its July around here. I can’t remember.

JE: But you’re just not starting to, that’s a new thing? Catching reds here or?

DW: Yeah.

KW: For me I don't know, you know people at Nikolai they call they say they’ve been catching reds but I don't know if they're talking about coho.

JE: Huh really.

KW: I think they're talking about coho.

AA: Yeah I think so.

JE: You don't remember catching any reds here.

AA: No.

JE: Maybe the reds are making their way up.

DW: I know.

KW: At the Takotna weir they always counted a red. Not always.

AA: Take over the lakes you know. Lakes to go.

KW: Normally but they're river spawners too. They're finding there’s river spawners. The bigger lakes though they prefer.

JE: Awesome. Yall have anything else you want to share. You want to wrap up?

INTERVIEW PARTICIPANT: NICHOLAS SNOW
Interview Date: 1/30/20
Interviewer: Janessa Esquible
Location: McGrath
Home community: McGrath

Janessa Esquible [JE]: (Goes over project and paperwork). So the first thing is that you are Nick Snow.

Nicholas Snow [NS]: Mmhmm.

JE: And is McGrath your home community?

NS: Yes it is uh-huh.

JE: What is your cultural affiliation?

NS: My culture? Uhh you mean what I'm I breed?

JE: Mmhmm.

NS: Eskimo and Aleut, Russian, German.

JE: Okay.

NS: Eskimo, Aleut, and Russian I guess not German. But somebody said it is German back in there.

JE: Eskimo and Russian?

NS: Yeah well, Russian, Eskimo, and Aleut.

JE: Okay.

NS: Because we're mom and dad both got Russian in them.

JE: Okay.

NS: But in Russians were, remember Eskimos too.

JE: Yeah.

NS: {chuckles} I'm Eskimo.

JE: So Eskimo, Russian, Aleut.

NS: {chuckles}

JE: And how old are you?

NS: Uh 69. Um.

JE: 69. Okay.

NS: Ii-i.

JE: And I'm Janessa Esquible and I work for Bethel.

NS: Uh-huh.

JE: I'm 29 and my cultural affiliation, just if you want to know, I'm, um, Ojibwe and I'm Mexican.

NS: Mexican. What's the first one?

JE: Ojibwe.

NS: What's that?

JE: Um, it's like (Anishnape), have you heard of that?

NS: Uh-uh.

JE: I'm from, so our tribe is from Southeast Ontario.

NS: Okay ummm.

JE: Great Lakes region.

NS: Uhh-huh. Uhh-huh.

JE: Yeah.

NS: I know, um, my sister and my brother they grew up (unsure of what word they said). Were scattered. You know we're just scattered all over the place.

JE: Wow.

NS: Yeah.

JE: Maybe you have ties there too then. {chuckles}

NS: {chuckles} We are, maybe were scattered all the way from here out all over to Asia. And you know in the country over there uh.

JE: Really?

NS: Yeah.

JE: Wow.

NS: Well that's what people say that people came from Asia in the first place you know. Most Alaskans.

JE: Yeah.

NS: Yeah so. I don't know. Uh-huh.

JE: Wow, that's cool. Yeah I haven't done the DNA test.

NS: Yeah that's what, uh, Judy's husband, he 99%, uh, what is it now, god dang it.

JE: {chuckles}.

NS: Umm. {chuckles} I wanna say… Anyway I'm bad with names.

JE: Oh okay.

NS: Really bad at names {chuckles}.

JE: It's okay. Me too, I gotta, I can remember faces and names I have to really try.

NS: I'm good at phone numbers though.

JE: Oh yeah.

NS: Numbers and so, yeah.

JE: Okay so the first question is, tell me a bit about your background, you said you're from McGrath.

NS: Uhh-huh.

JE: How did you grow up and what connections do you have to salmon?

NS: Well I grew up here in McGrath before this side of town. And the first connection with salmon is going across the river with mom. And checking the net with mom. And with boat. And I have horse power. And I remember putting it in coffee cans and water so they

wouldn't get freezer burnt. But it took a lot of room to, you know, uh yeah. Instead of just wrapping them up she put them in coffee cans and filled them full of water and,

JE: Oh really?

NS: Keep them from uh, frostbite, freezer bite, yeah freezer bite. Freezer burn.

JE: Wow.

NS: Uhh-huh. And then I just kept in there when I was gone, of course, I set net all the time and I gave, I um, I'm a giver I guess, giving and receiver. I guess I'm a giver. {chuckles} If there's any I usually give lots of salmon away and that's what (inaudible) that salmon or whatever im catching.

JE: Yeah.

NS: Uhh-huh. But..

JE: So you give a lot of, so you provide for the community?

NS: Yeah but um, uh-huh. Uhh-huh. Community like, uhhh, Mr. Hoffman I don't know if you know him. Stanley Hoffman, Stosh's dad.

JE: I do! Avery Hoffman has worked for us for many years. His grandkids.

NS: Stanley Hoffman must be one of the Hoffman brothers done there.

JE: Yeah yeah.

NS: FAA. Like he, Stanley says if someone needs help, help anybody, I remember uh, thats {chuckles}

JE: So you know Stosh Hoffman Jr?

NS: Oh yeah I know Stosh Hoffman. He's really good too, uhh-huh.

JE: Wow.

NS: And I met his wife up there, and uh, Talkotna.

JE: Ohhhh.

NS: They put a cabin up there. And then they put in a new propane stove and then they want to install a toil stove for them.

JE: Wow.

NS: (inaudible) and a propane stove. Yeah I knew Stosh. When he was going to school here. And his mom Judy. And uh-huh.

JE: Wow.

NS: (Unknown of what was said).

JE: Yeah I was told you provide for a lot of the community here.

NS: Yeah. {chuckles}

JE: That's great.

NS: Uhh-huh.

JE: So that's your connection to salmon?

NS: Okay yeah after, yeah, and then I went to the (unknown) FAA, and I was for awhile and I came back and uh, Sol and I were putting up fish, salmon, dog salmon, and putting away salmon, we had a few dogs. And then that would be in the early 80s. 70s and 80s. (inaudible) I guess 70s that would be. Sol and I put up fish in the backyard. Smoking it. We didn't do a good job on that one {chuckles}

JE: No {chuckles}

NS: With the dogs. For the dogs yeah. You have to smoke it all the time, yeah. And we did that for a while. And I never set net for quite a few years. And I ain't got it no more. Oh! That's because I was DNR and summer time you can't really, uh, the salmon get soft and get ruined and then they, the longer you leave the net the ones that die another one catches. I believe that the live ones are safe and the other ones will be a waste in salmon. So I didn't really lot of salmon during the DNR years. And that was a 12, 15 year period there.

JE: When was that uh,

NS: I go downriver, and you know, and I went down river canned salmon up Swift River. And King salmon, I don't know what year that was. It might be the 90s. Yeah late 90s because uh, I went down river and canned for cases of King salmon. It's not just Swift River, it's another off to Swift River. Downriver. I played with salmon down there before too that's fun.

JE: Oh okay.

NS: And then I went up to (Talkotna) river and we caught a few silver, in the fall time up the (Talkotna) River where they seem to be up there now too.

JE: Oh really?

NS: Uhh-huh.

JE: Wow okay. And..

NS: And last year, well, yeah and then uh, well last year was my best year and not like summer before last. Not last summer but before uhh..

JE: 2018?

NS: We caught 40 kings last summer before last.

JE: Okay.

NS: I found a good eddy down here. While actually my brother found an eddy and uhh, yeah. (Inaudible) The big one got away. {chuckles} Oh boy.

JE: They did?

NS: We don't get big salmon like we used to, you know. The salmon are a lot smaller, they used to be on (unknown). Years ago we used to get these small salmon, they call them Jack salmon and now a lot of them are not much bigger than a jack salmon. You can find upriver now and then. You know, I guess they call jack salmon younger salmon that come up and head back down again or something.

JE: Uhh-huh.

NS: And so uh, yeah the salmon, the king salmon they don't look like the king salmon we used to catch.

JE: Really? Did you use to get jacks too back then but they were even bigger?

NS: Very few ones and while the jacks were small you can tell they were salmon but you didn't know what they were at first. I found out they were jack salmon. We didn't catch a lot back then either.

JE: Okay.

NS: Um, you know don't catch a lot but know where, (inaudible), I guess I'm saying the salmon seem to shrink through the last bunch of years.

JE: Shrink? Yeah.

NS: Uh-huh.

JE: And um, how was the salmon fishing when you were young versus now? The salmon are smaller…

NS: Uhhhh, I don't really know, I mean because I never uhhh, umm.

JE: Like was fishing any different for you?

NS: When I was young-young. I didn't fish when bout 20 years old until 30 almost. Went to the FAA and out of town here. Military. 6 years. 7 years. Anyway, but um, it was just bigger and if you saw me, yeah, they were a lot bigger.

JE: Mmm. And?

NS: And I didnt put (inaudible) with everyone else, yeah. I can yeah. (Inaudible). Didn't have nets when I was a kid. Salmon or something like that when I was little young. Mhhm.

JE: Hmmm. So they were a lot bigger and how did you learn how to care for salmon when you were growing up?

NS: How do I learn what?

JE: How to care or like you know how to manage the salmon?

NS: Mom and yeah. Mom and many freezing. That's what we did a lot.

JE: Really. Mainly froze.

NS: We tried, ah, the salmon eggs are really good, uhh, many salmon strips up here in McGrath because theres not enough (ores) and stuff, I mean yeah, you know make salmon strips and I dry them up and put western oil or (unknown) and seal oil.

JE: Oh I see.

NS: You know you soak and make it more, yeah, cause it's so dry.

JE: Okay.

NS: Cause we went up years ago in 62, 63 when I was 12. We went up to Stewards bend. With mom and Alex Peter and my older brother, Billy went with us, uh. Alec and mom put up salmon one year up there.

JE: Mhmm.

NS: And they came back with several gallon boxes, and came back with (salmon) full. But they, uh, yeah they were Yukon salmon {chuckles} (phone ringing in background)

JE: Yeah yeah.

NS: (answers phone).

JE: Do people still follow the same rules?

NS: Excuse me?

JE: Uh, do people still follow the same rules and like the way they take care of salmon uh today like they did in the past?

NS: I believe so yeah.

JE: Yeah.

NS: Uh-huh. Yeah well like well, more freezers.

JE: More freezers.

NS: I mean so everybody freezes, works through it, does nothing to them but em in a ziploc bag and roll em up and put them in the bottom of the freezer and you know, uh, they don't gut em or anything. More opening to get frostbite.

JE: Oh.

NS: They wrap the whole (language) through the years.

JE: Really.

NS: (Inaudible) back in this, on the Yukon, I went there one time, they went out before the salmon were coming and yeah, ziploc bag.

JE: Wow.

NS: Yeah it's better that way. So it, I do that and I whitefish too you know, nobody really, for (guluk) no one cleans them before they freeze them. They just freeze them and then when they get ready, unless they are frostbiting. But yeah.

JE: Good to know.

NS: Back in the day, the old days you didn't have freezer space. Everything had to be.. Little freezer space anyway. I remember getting our first freezer and our first refrigerator back.

JE: Really.

NS: Yeah.

JE: What did you do?

NS: (inaudible) 29 my sister got locked in. My family was right there when we got it. (unknown) and I jumped in there the little one and we loved to get in on purpose and realize we shouldn't be in there {laughs}. They'd open it and we definitely panicked {laughed}

JE: Oh my gosh. {chuckles}

NS: When we were little people, the freezer was little too. I mean the freezer was you know, the refrigerator opened it up and had a little freezer up there. You just pull down and open the freezer and like, I remember that. And it was a little freezer so we had to be little people.

JE: Wow.

NS: I mean yeah we were young kids uhh,

JE: Yeah.

NS: Uh-huh.

JE: What did you do before you guys got the freezer? Did you smoke them in?

NS: That's what mom had a smoke house right over here in fact. I remember my sister falling off of it. But ummm, I remember collecting.. Mom used to like um, wood. The wood that was kinda uh sprucy, you know. Rotten wood. Then uhh, reddish color or whatever. Momma used to go riding around town here because it was lotta wood here (inaudible). Because yeah. That's right, collecting wood for mom and her smokehouse. Because smoke had a different flavor.

JE: Mmm. Okay.

NS: (unknown) different cottonwood, well it was rotten and it was kinda reddish and you know mostly ummm.

JE: Okay and how did your ancestors manage the salmon before we had Fish and Games, Fish and Wildlife. All those entities. How do you think they took care of the salmon?

NS: Well I well, do you uh,, (unknown) the same as lotta people. You got enough for the dogs and self and that you know that was it. And they uh-huh.

JE: Mmkay.

NS: Cause they. You know, they uh, salmon.

JE: Yeah.

NS: I don't like people that (unknown) they went to salmon river all the time and put up the fish you know.

JE: Okay.

NS: Uh-huh. And that was like a short distance from (unknown) summer time. You have to go by boat. 120 miles by boat.

JE: Wow. Do you…

NS: But yeah smoked it, everyone maybe smoked it. And yeah also when I was younger before the freezers we use to get Holy Cross we used to get a case of salmon bellies in salt.

JE: Ooohh.

NS: Yeah uh-huh that was really good for pickling and they just soaked em and ate them But yeah. They (unknown) wooden kegs, and uhh. And sell out, what's it called (inaudible).

JE: Sounds good.

NS: That's where the blueberries were too. And wooden (kegs) to uh,

JE: Oh uh wooden underground.

NS: Under the house. In the dirt right, kinda a cellor. You know, not a basement. No wood or anything. Hole in the ground.

JE: Oh I see.

NS: Hole in the ground under the house.

JE: That's a good refrigerator though. Freezer. That's a good space.

NS: It's cool, yeah when it freezes. But at what yeah. Uh-huh. It stayed cooler. Well the whole house was cool. I can remember (brick) and ice water in the morning and make coffee. (Inaudible) we were happy campers {laughs}

JE: Yeah

NS: But I can, yeah, I can remember cause the stove would be out and mom would get up, make a fire and stuff. And yeah. (unknown) grabbing a t-shirt and back up to the stove.

JE: Really. {laughs}

NS: But yeah so.

JE: Mmmm

NS: Yeah it was a lot more, we needed a refrigerator like the blue cheese when we were kids and we never had a sink or anything else. But then the cabinet with the blue cheese was under there, well blue cheese is bad all the time people think but I like it. (unknown) liked it. It's kinda like blue cheese is the same as what they used to make yogurt. You know junk for your stomach.

JE: Oh yeah.

NS: And same for blue cheese.

JE: Oh blue cheese.

NS: Yeah fermented. Uh-huh. We always had that under the refrigerator. I mean under the cabinet in there when we were kids.

JE: Mmm.

NS: And I guess, why old timers like that blue cheese around because it was yeah.

JE: Good for the stomach.

NS: Yeah good for the stomach uh-huh.

JE: Mmm. Do you feel like your knowledge or wisdom, values, are they reflected in management today? Fisheries management.

NS: Uhh I don't know. I.. Yeah. I'm pretty happy with what I've taken that was.. I've always been happy with uh, I don't like to agree with them all the time but uh I realize. No I'm always been happy with rules and regulations in the state of Alaska. Because uh. In then (unknown) and then the government backing up the assistance that's another thing, you know, that uh, you know overall, yeah, you can't go back to the old days. But I sure appreciate what's being done today to preserve everything. And the old peoples rights too. Which (unknown) native rights I believe. Republican state.

JE: They're against native rights. Yeah.

NS: {laughs} That's the way I feel anyway.

JE: And do you have any concerns about the salmon fisheries today?

NS: Ummm, don't have any concerns. I aint, I don't know if, I don't think anybody on the river up and down past Aniak, will kill off the salmon. It's downriver, down below in Bristol. In the ocean, we are killing them in the ocean too by polluting the world. We've been killing off this world since we've been here but uhh, (inaudible) And I was in Anchorage for a while and (unknown) because there's no responsibility. All they do is throw their baggage around. Back in 84 I was getting 16 dollars an hour to do that.

JE: Wow.

NS: the union and uhh, I was (unknown) shifts so they gave me an extra 50 cents an hour. But I was, I don't know and they were. I just see Anchorage and everything else and uh, it's like (inaudible) the world I felt like. There's stupid people that handle all the baggage (inaudible) {door creaking} they look at the airplanes, they have their own little tunnels to walk in and other people that tunnels that come in you know {laughs} but I (inaudible) we've been killing off this world for a long time. I don't know if it's yeah. Overfishing. And then I don't agree with Bristol Bay cutting off the gold. Uh, like gold people. Whatever the heck it is. The name of the, not Donlin.

JE: Oh the pebble?

NS: Pebble mine, yeah.

JE: You want the mine?

NS: Yeah I think it's safe because they are large enough. You're not putting a pebble mine in the middle of an area where the population is right next to you. And now with more (inaudible) where they got a two hundred foot (unknown) on top and they go so many feet. Another 200 feet. And another 200 feet. (Unknown) we got enough land. We have to build this dike that's gonna collapse in the first earthquake you know. We're gonna build dikes that are plenty big enough to handle anything they're never going to get filled anyway. You know that's what I believe.

JE: I see. Okay.

NS: You know so I hate that the mines would be a good idea because Alaska like I said, Alaska and the strict rules too. And you know like, once they put a proposal forward. It's gonna be fine. I don't think so. We're losing our fish already. To the ocean pollution. I mean, that's not, the pebble mine. I don't think the pebble mine would have anything like that because of the amount of land we can use to make a dike and pool and everything. We don't have to be (unknown) because of the room they got because of the populated area like. It happened down there. You know they got room to make uh, make it safe. That's what I believe.

JE: Okay. Room to make it safe. Write that down. And um, so you said you think fishers managements are working well. You mention that you think that the regulate, sometimes you get, you don't always agree with regulation or.

NS: Yeah well it's like (unknown) when it closes it down, it aint fish and game (inaudible) you know, and the size net, because you buy a 300 dollar net you find you cant use it because its too big of a mesh. {laughs} and that's well, I mean you know.

JE: Yeah people can't afford…

NS: (inaudible) You know you don't have to like them but you should follow them because it's for your benefit.

JE: Okay.

NS: {laughs} maybe then of the time.

JE: Would there be anything that you do?

NS: Everybody makes mistakes but I mean if yours (inaudible) trying to do right.

JE: Would you improve, if you were to make any improvements for the you know, fish manager. Would you make any?

NS: I don't know, limit the size of uh, my mesh. Because like I said that's what happens, everybody got big mesh so it's all the big salmon off you know. In such a limited size of mesh then maybe I don't know if we'll get them back but I mean you (inaudible) whatever they're doing is good so far you know. I mean, like counting fish and they keep track of things and I mean yeah. What can I say? They spend a lot of money to keep us, to find out what's going on with these fish you know. Uh.

JE: Okay, and is there anything you want to see for the region in the next 40 years?

NS: Uhhh,

JE: Your region? The salmon? Your people?

NS: I know many many people here in the region in this area because of uh, the cost of living and everything. And I uh, maybe just make it happy for up and down the river in the 20 years but like I said {laughs}

JE: Have people…

NS: You know 20 years from now if anybody can afford to live out here in the woods just because of the way, yeah, I don't know. Yeah.

JE: Mmk. So.
NS: Yeah… Yeah.. Mmm. Even if people don't want to (unknown) they cant come up and moose hunt. You can't come out and uh, oh they can't go geese hunting and bird hunting and everything else in the spring time because they're not subsistence hunting anymore because they live in Anchorage and they come out in McGrath even though they were raised here and everything else. They still can't do it because they don't want house and property here.

JE: Wow.

NS: I cant like that too. {laughs}

JE: Yeah you have to live here to hunt.

NS: Uh-huh.

JE: So you want to keep it that way.

NS: (inaudible) and then they got this, yeah I think overall I'm happy with everything.

JE: Okay.

NS: Mmmhmm.

JE: Is there anything that you want to share?

NS: Umm, the ice fishermen too. I give a lot of whitefish away. (Maryann?) caught over 60 um, broad and maybe 5 humpies this year.

JE: Oh yeah.

NS: Under the ice.

JE: How are the whitefish looking?

NS: Well the whitefish they're uh, under the ice, the pike fish because they're different. I mean they're more solid. You know. Yeah. Even Gary was telling his friend, Gary was telling his friend down lower 48 who had net under the ice and were catching fish and he said 'boy I must some be some good fish' you know in cold water and uh-huh. And after a lot of people, yeah, makes better akutaq or something. Cause it's almost like summertime they eat, they get softer and almost like mush almost.

JE: Mmm..

NS: You know when you grab them and you pull them out of the net it seems like you're dealing with a bag of mush or something.

JE: Yeah.

NS: And in wintertime they're nice and solid. I mean their.

JE: I like whitefish. Whitefish is good.

NS: Yeah uh-huh. A lot of people like it. Only got maybe 1 cottage cheese full of akutaq this year. All the whitefish. Usually I get something back you know. Oh no that's right, Nick Gregory and (unknown) they always give something back when I give them something, you know.

JE: That's good.

NS: But the younger ones they don't really get (inaudible, starts laughing) Three nieces.

JE: They're not catching on.

NS: Well yeah I don't know if they bring (unknown) you know like akutaq is uh, (unknown) is small. But you know, my sister still makes the best akutaq.

JE: Really.

NS: You can take it out of the freezer. Freezer nice and frozen and still take it with a spoon and eat it.

JE: Yum.

NS: It gets all the moisture out of it. And enough and everything, I always said if um, somebody give you bread, if they offer you akutaq don't take it because they never put time in their bread (inaudible) akutaq {laughs}

JE: Yeah.

NS: Makes it good and everything. Don't eat their akutaq probably.

JE: Yeah, one of my friends is really yummy akutaq. She put um, raspberries, salmon berries, blue and strawberry. It was so good.

NS: Yeah uh-huh.

JE: Super fluffy and oh.

NS: The fish leftover from last fall. That was good last fall and nice and warm. Nicholai. (Unknown) the last nine or ten whitefish out there. But yeah I always hit him up. There for a while. Well I started getting whitefish under the river with uh, when they were trying to get whitefish from different people bringing in. We got whitefish in out river just some (unknown) {laughs}

JE: Yeah.

NS: I ended up buying, I went all the way down to 3 ½ inch mesh up to uh, 6 inch 5 and something mesh under the ice. You know, only seven feet. Hole above the water weighs heavy enough. And if you got a 7 foot net, you have at least 8 feet of water for your net so won't freeze to the river ice when it freezes. (unknown).

JE: Okay.

NS: and then I um, would come up with, in the old days they used to tell us to get a willow. You know that willow and then put something on it and loop it through to hold, drill a hole and uhh (unknown) and get better. But I came up with an idea when I set up (unknown) years ago, you have to have 8 feet of uh water. 8 foot pole and you get a 4 foot pole and you put it together and then you can just drill a hole here you know like hole you can measure it, drill your first hole. And then you put a fork on the front of your one stick.

JE: Ohh.

NS: And you got a stick here and stick here. And no more willows. That's my idea. And then when you push it in, you can hold it uhh the one of them plastic coca cola bottles on the right over there. (unknown) look at the dust. (inaudible) Works really well on the end of the stick.

JE: Really.

NS: Cause its real plastic and uh, so what you do is you gotta string on it. A fine string and you stick it down and go down and hit the heads and would want to float. So it floats and then they have the little coca, best one so far. One of them. Coke bottle floats and. You put your hole, one hole and you measure this guy 8 feet apart so when it comes up you just turn it back and forth and some… and let go of the string and it pulls up. And you pull it here and then go to the next hole and you do it that way and you do it early in the Fall. It's really easy cause you haven't got that much ice you know.

JE: Yeah. Yeah.

NS: But I did this the first time when we had about three feet of ice and 2 ½ feet of ice and that stick when it you know (inaudible) so that works really good. Gary had a set net in 5 years is always working in the fall. And he didn't want fish this year. And leonard was gone. Put a net in and yeah. Works real fast. Went out there and set a net and less than an hour and a half. We have ice in October. Yeah. {Laughs}. I was only (unknown) in the

early fall. It's really nice and a lot less work. And then when we did it before and it never worked out but again I got led line you know. No led on it. So we tied a led line to the willow leave it in the hole down there where we had one of the net and then we down here we get (unknown) in the spring time we won't, we can just dig up the willows with a string with rope tied on the end with a led line. Between the holes and put no holes and just put two. One on here and one on there set it again. But we never do good in the spring. When it's really good is when the ice is too dangerous. That's when it starts picking up and then you gotta pull out.

JE: You can't, it's too risky.

NS: Uh-huh. But there's a good eddy down here too. A real good eddy for whitefish. Cause I when like put one in last spring caught them whitefish or two,

JE: Nice. I’m glad you guys did well. That's great. Well thank you so much for sharing.

NS: No you're welcome you bet.

INTERVIEW PARTICIPANT: MATILDA EVANS

Interview Date: 5/11/22
Interviewer: Jacqueline Cleveland
Location: Quinhagak
Home community: Quinhagak

Jackie Cleveland [JC]: Can you say your name and spell it?

Matilda Evans [ME]: Matilda Evans

JC: Today's date is May 11, 2022 and we are at noon.We're in the home community at Quinhagak at tribal housing. Can you say your Yup'ik?

ME: I am Yup'ik

JC: And can you say your age?

ME: 58, I just turned 58, April 27.

JC: Oooh day after Janine's Birthday. Happy Belated Birthday. So as a start I am going to ask a question. So you could either if you are finding it hard to understand, ask for it to be translated. Please tell us a bit of your background. Where are you from? How did you grow up? And your connections to salmon, in your upbringing.

ME: How did I grow up?

JC: You're from here

ME: Oh

JC: I did talk about cutting and drying fish, in your upbringing, I talked of…(can't understand)

ME: Can I say something about my mom?

JC: of course...grandpa and grandma

ME: My mom would say that my grandparents would cut and dry fish, and during our breaks, you know we run up from the fish camp, but she would say that her mother would admonish her, "Ok please do not to be cutting fish (as she was inexperienced)" and as they would go up and leave her, she would try to cut fish, and when she finished a fish, she would hide it under the pile of fish, and when her mother found it she would ask her, "did you try cut fish again", and as time went on, her mother started to praise her fish cutting skills, saying that they were better than hers or anyone else's fish cutting work. So it is that way. With me, it's with trouts that she taught me how to prepare and cut fish, down at the gravel pit. Started off with trout first and then with salmon. Yea, but the fondest memories was, as youngsters, at the old KRS campsite, and then that river in that area, maybe it was Mark, or Cingadayuk.

JC: Oh yeah Cingadayuk.

ME: We used to stay at his camp, as a youngster, I don't know how old I was. And mom would cut fish and prepare them there, when there were just the three of us. Me and my

brothers, one time, I don't know where dad was, but one time my brothers found matches and started a fire at the fish camp. So we kids hid, I don't know how we went, but we were scared. They came through the swamp, to help mom to take it out.

JC: It was a good time to be a child back then.

ME: And then when we went home, the fish were strapped, you know, now the strapped fish are put away first, but that was the way back then. ..

JC: Maybe this next question will be the same, or oh no..could you tell us about salmon fishing when you were young. I think you just did, but you can add if you want, and how was it different then it is from today, compared to when you were young, what has changed in procuring salmon?

ME: I don't...I haven't really understood yet, you know with my own eyes, where after someone fishes, I haven't observed, you know, when we catch fish, we put them in the boat, like that, but now they do not ...uhm my mom used to say, but I was not here after I turned 18, my dad told me to sign up for, not sign up, but to apply for work in the canneries as he used too, so I followed his advice, and after that I went to the canneries every year during the fishing season so I missed out on subsistence. My mother would tell me of my late brothers, when one would catch a number of fish, the other would try to out catch the other. My mother said that she didn't know that was what they were doing, but realized that they had been trying to out catch the other all this time. My mother would cut and prepare the fish, and you know, the fish used to be a lot bigger than now.

JC: Are the kings a lot smaller than they used to be, in your reckoning?

ME: Yup! they are a lot smaller, I always feel sorry for them, you know on the north side, that side. They just can't go out and fish, they are regulated. Perhaps some of them come down here and fish for salmon.

JC: Yes, I also feel sorry for them.

ME: You know the other two big rivers (Yukon and Kuskokwim) and in Hooper Bay they did not get any fish either. Perhaps we are a little lucky here. We do catch fish here, and the biologists here have said that we, at Quinhagak, that our ecosystem is pretty resourceful, and right outside that (ecosystem) there are lean runs, but now and then we do have lean runs, but I also feel trepidation about the mines, and that of our waters also, when they do talk of the mines

JC: Yeah that is a concern, and that will relate to this question down here, so we can come back to mine.

ME: Next question?

JC: How did you guys manage or care for salmon when you were growing up?

ME: Do you think that we covered that subject already?

JC: Yea, you did answer that question already. Next question, are these...rules being taught and being followed today? and if not, or if so, then why or why not, or why not i guess.

ME: I think they still follow the rules, but perhaps not even half of them, each generation, they keep changing...(unintelligible) some are taught, some are not.

JC: Ya, uhm, how did our ancestors catch fish back in the day, and how did they care and prepare them? In your assessment and what kind of nets did they have?

ME: That area, on the coast side, used to be very nice, all of it was water, Mr. (unknown) when they were here, I was a little girl, and I came too, their daughter...they used to have a big boat with a huge fan in the back, and we went on a boat ride somewhere. It was very enjoyable and there were fish racks and I used to be with my grandmother. Seems like I was always around here, and one time I was playing with a fish hook, doing this, and the fishhook went in my eye. Then my grandmother took me into the house and just took it off. I just remember that. I probably didn't answer your question.

JC: Oh that's fine. I had a hard time answering that question as well. Maybe your ancestors, how did they catch fish and prepare them, did your mother or grandmother tell you of these ways of catching and caring for them?

ME: My mom used to say that when my grandmother made fish strips, you know they used to tie the ends, my grandmother was the first one to..., my mother mentioned that one time, that my grandmother was the first one to do it, I don't know how she did it but she did.

JC: Was the first one to...

ME: Come up with the third cut style without the string. A whole connected piece we are talking about...I don't know if they will understand. Umm Luck (person's name) used to talk of, at Eek, when the fly's were horrendous, because of the heat. They used to smear the fish with the fish blood.

JC: Yea, I mentioned that, if there isn't any other, but do want to add anything?

ME: I am trying to think, but it's like my mind is blank.

JC: What does indigenizing our ways of knowledge about managing salmon look like for you? So if it were all up to you, as Yup'ik how would we manage the fish?

ME: …Would manage without restrictions and share alike and not waste them. They need not to be wasted anywhere, you know some do not clean the fish well or the processing area clean, and they do not dispose of the parts properly. It is not advisable to improperly dispose of salmon parts anywhere. Although not everyone's the same, some are just messy.

JC:It would be good if we did that, in my opinion. If anything crosses your mine, you can always go back to it. Do you feel like your knowledge values are reflected in fisheries management?

ME: You are aware that we as Yup'ik do not rely on science, because we know when the fish are going to arrive by observing the climate, the weather, by the coincidence of birds arriving...

JC: Our knowledge and our ways regarding gathering food, are those incorporated into fish management or what do you think?

ME: I doubt that. Peter Joshua and I used to fish, and he would tell me, and then one time, we were fishing, and when I observed the weather, it was nice and calm...unintelligible, and the wind picked up and it kept changing, it was at the time that the silvers hadn't hit yet. Peter Joshua would say that when the silvers hit, it is a very enjoyable time. And I used to want to stay when they (silvers) hit, and then one evening about 8:30, the wind change, the clouds, and the weather was changing and then the wind picked up, and started raining and at that point, the net (making splashing sound), when they hit, that was very exciting. And then at that time the fish that hit, they just disappeared, that was just exciting. I haven't experienced that again since. I was observing the weather.

JC: Did he predict the hitting of the fish by observing the weather?

ME: Well he followed what the weather was doing, and I found out that is what they do. And my mom used to say that every fourth of July, you can catch a lot of fish, and I have come to know that is true. They do catch a lot of fish on the fourth of July. But sometimes on the fourth of July, they do not fish. That is also true. ..and he used to be the chairman, when I was small. I used to work in Ekuk cannery, and my mom used to fish with the other ladies, when they used to commercial fish for 24 hour openings, When Teddy used to buy fish, and she would bring home cookies and other things.

JC: Wow, your mom used to fish with other women as her deckhands?

ME: Yeah, (person's name) now deceased, was her favorite (unknown) and those others that are no longer with us, those in the canneries that used to work in the ships, but...I don't know how old I was when they used to do that. I used to stay a lot around my grandmother's greenhouse. It seems like I was the only one that used to stay around my grandmother, I do not know where my two brothers were.

JC: Should I ask a different question?... Thank you. What were or are your concerns about our salmon fisheries today?

ME: Just the disposing of salmon parts improperly is my concern. If they would dispose of the salmon parts properly that would be good. proper way of things...(unknown)...to properly though.

JC: How would the river be good?

ME: You know, some just throw the fish guts and such into the river, if they did not do that, that would be good, and others just dump them onto dry land where we can see them. and where animals could get to them. That is not a good process, but I don't know why they do that. You know, there are more and more people improperly disposing of things like that. Where my parents used to dispose of is where I dispose of fish parts too. The area where the ground sinks. At one time, when I did not have proper working vehicles and no one to help me, I disposed of parts to tengluk, and I felt bad about it, but I couldn't help it. I disposed of what had been in the freezer, when I was cleaning out my mom's freezer, when we did not have any vehicle, just a bravo (250 cc snowmobile).

JC: Are there other concerns you may have? Yes, what you mentioned are very important, what you have said and maybe are our fish, here, the decreasing numbers, is that a worrisome subject for you?

ME: Uhmm..

JC: Or there are a lot of people fishing here, and we fish the whole river, but there are no counting numbers. Is that, such as the kings getting smaller, and the chums dwindling in number, do you notice that? Last year, we were...(unknown)

ME: I only remember what my mom used to say about king salmon or fish, and you know we also fish with rod and reels and some catches are just small, my mother used to predict this by observing the catches as a whole, that she would say that there are going to be plenty of fish, or...she used to say a lot of things, mostly by observing the size and quantity of the fish. And she would predict by the size of the smelts whether the salmon would be big or plenty, she would observe all these to predict what the future holds regarding the fish. So, I am also happy, when I see the smelts, by how big they are.

JC: I do not know how they used to know, and Luck used to know things, and when she predicted, those predictions would come true. She would say, the fish are going to be small, but they would be plenty, and they would be like that. And I forgot how she knew that though.

ME: Yeah, I thought my mother wouldn't predict like that, you know some, even if they are not well educated, they know about subsistence and survival, a lot of things. Yeah I think this is so amazing to me.

JC: Thank you for mentioning that, it is definitely true. I don't worry about those, but I know people here know a lot of knowledge, as Yup'ik, because we grew up in this land, and this land sustains us, provides us sustenance, our land, our water. What you have mentioned is really good. Perhaps you did mention about improvement, but let me read this, next to the last question. What are some concerns you have with the fisheries management today, and in the past, what works, what doesn't work well. How would you improve the fishing out here?

ME: Fishing out here..

JC: Or the management like, you know the state manages the fisheries here, in the bay, and in the river.

ME: Even if they did not create the area, they do, from time unknown, our ancestors, even if they do not regulate it, that would be ok, the state did not create the bay or the river. From starting on regulating the reindeer herders, the state has been regulating these.

JC: The state does regulate this area.

ME: I do think that, you know, we had a small cannery, if they had, you know Ekuk has a dock, and I enjoyed working at Ekuk, and I don't know how many years I worked there, but i used to look forward to working there since I was 18, and it was my last year maybe in 87, from Ekuk to Kenai, and from Kenai to Alaska Princess, we went to through the Aleutians to Kenai again. But if the cannery had been down here, I don't know, but you

know, the mouth of the river is not good for trying to enter. If they had a port as Quinhagak, it would always be dry, but if we had a dock, it would go a long way, but it can't just happen.

JC: There is no parking place and no launching area...we don't have a dock but a launching area. The last question is, what do you want to see for salmon and people in your region or here? How do you want to see salmon and people in the next forty years?

ME: Well, if they want, if they love having salmon, the way a person loves to fish and if he loves our river, if he respects that, we would probably be better. And when they go camping, if they keep an eye on things, and trash and bring them home. The cans in the tundra, those things, I think they may pollute our land.

JC: What about the salmon?

ME: The same thing with the salmon, don't just discard them anywhere. Do not waste them, and share them.

JC: Follow the sayings.

ME: If only the late grandparents would be here, it would be awesome. The grandparents, even if they aren't the same...(unintelligible) like how my grandparents used to treat me. They don't act as a go between anymore, some grandparent do teach. That is really missing now, being taught and talked to.

JC: That was the last, the last but if you have any other thoughts you can add regarding fish, or management or if there is no other...do you have anything to add?

ME: No, I can't really think right now, but if I have something, can I write it down and leave it to you or…?

JC: Me too, when I can't remember I'm not up to par when that happens, but I do say I will bring it up later, yeah if we have anything to say that enters our mind, it can be included.

ME: Yeah, I myself, yesterday when I saw your text I thought that if I just looked at the questions and answered them they probably would have... but I didn't follow that.

JC: No you were good, this way, more natural. Myself; if I haven't looked at them before, ...but you did well, thank you. This marks the end.

INTERVIEW PARTICIPANT: JOSHUA CLEVELAND

Interview Date: 5/11/22
Interviewer: Janessa Esquible and Jacqueline Cleveland
Location: Quinhagak
Home community: Quinhagak

Janessa Esquible [JE]: Ok, we have Janessa Esquible, Joshua Cleveland, Jacki Cleveland and we are in Quinhagak. Where are you from?

Joshua Cleveland [JC]: Originally from Eek but moved on to Quinhagak.

Jacqueline Cleveland [JCl]: Translates question in Yugtun.

JE: And now you live here, this is your home community?

JC: Yeah

JE: You wanted to share a story?

JC: Oh yeah, back around maybe 1969 or so, I was hired by Alaska Department of Fish and Game in Bristol Bay dealing with the salmon. First with the smolts coming into the bay from the lake. One year old smolts coming out of the Kvichak river. I was stationed at King salmon that summer, and I was with them for a couple of months over there. We monitored fish coming from the ocean into the lakes to spawn. That was when all my coworkers were students, all white people {laughing} (Some of the sharing is not understandable).

JC: I was born and raised at Eek and my parents were both alive then and used to camp on the north of Eek river and that's where our fish camp was for the summer. My parents were always working on the salmon then and there was no commercialized fishing then. All of the effort was for subsistence catching all five salmon species coming in from the ocean. They were numerous, very big king salmon compared to what we have today. There were lots of them. Chum salmon was very popular too in those days. In 1975 and in the 80's, it was a small commercial fishery, not a big one. The fish were used to supply restaurants. There were only a few fishermen doing that, probably mainly from Bethel. When I grew up to see my parents take care of the fish, basically the whole family would go to fish camp. Each village would go down to their fish camp in the summertime, practically all of them would go down to fish camp with tents during the summer for the next two months, living out there. Basically all the fish, we don't know how many, each family had two large drying racks for salmon to dry. Maybe for the chums, up to 50-75 chums on one fish rack. Maybe about 100 fish are taken right at the same time and sometimes they load the racks with more salmon after the first group dried. A lot of times, most of the time, this was also a food source for our family and dogs. That's basically what I saw when I grew up.

JCl: Translates question in Yugtun

JC: {Takes out book} It's kind of hard to compare fish to what was then compared to now, but with my personal experience, I see that the fish are not as fat as they used to be. Their oil content is getting low. Chum salmon were fat. Today, they are not fat, and they are getting smaller in size, and I don't know why that's happening. Some think it may be because of the food source in the ocean, it may be getting too warm out there to supply a food source out in the bay. That is possible. Their oil content is getting low and the fish are getting smaller. I wanted to show you a couple of things from my old book here {Shows pages from the text Bethel in the past 100 years}. Two things I want to show you. One is, this is 100 years of information about Bethel, and in 1985 we had our first 100 year celebration of Bethel. The story behind the first commercial fishery was that fish were being delivered to a small company. They share the fish and the people are taking care of the big fish. These are the fish we were getting in these days. That was about 1957, it was 64 years ago when the fish were like this. They were fat and they were big. Looking at another one, this is a picture of a commercial fishing deck with fish that are waiting to be delivered. This is chum salmon. This was taken in 1981. We can tell by this picture just how enormous the chum salmon was in 1981. They were getting them by the boatloads. Today, my friend from Napaskiak told me that there is no more chum salmon coming into the Kuskokwim. That is sad for them because we saw all of that source of fish and for sled dogs isn't there. A lot of them had their dog teams up the Kuskokwim river. So this is salmon being taken from the river and it was numerous and today we see there is no more commercial fishing because all of that fish is gone and a lot of it is the King salmon. Why are fish disappearing? This is another big question that they always have. It's a sad, sad story for the next generation. That there is still a way to supply a food source from the bay, there is little source available. All of the Kuskokwim river is closed to commercial fishery along with subsistence fishery. It makes it hard for all of the people up there utilizing this source. We are still fortunate in Quinhagak right here in our own community because this is looked at by Alaska Department of Fish and Game not as a Kuskokwim tributary. It's a salmon tributary by its own because it has two rivers, Kanetok/Qanirtuuq and Agalik/Agallak, the two rivers where those five salmon species come in the summertime. Kanetok takes all five salmon species while Agalik takes most of the silver salmon and not as much as other sources, like kings, reds and chums. Commercial fishing is open in Quinhagak because they say this is our own source of fish that are coming in is different from the fish coming from the Kuskokwim tributaries, so this is not restricted, but there are some restrictions in King salmon catches. We are limited to how many we can take.

JCl: The only restrictions we have in the village is what the Tribe asks for ourselves and to help to decrease the traffic of all of these other boats coming in to subsist, like instead of saying oh we don't want anybody else here, we are limiting to 6 inch mesh, which are the same measurements as the Kuskokwim, no Sunday's, with 45 inch mesh deep. Those are like restrictions, but we just thought by restricting Sunday with all of the traffic coming in, it wouldn't be two days in a row of massive taking, only just one day of the weekend. So even though it sounds like a religious ask, it is too, it is too because the town doesn't do anything on Sunday's subsistence wise, so that's kind of where that came from, the no fishing on Sundays. So that's our only restrictions, it's going to be the same this year.

JC: So Quinhagak is the only one that is open to both subsistence and commercial fishing out in the bay. Their commercial opening area is maybe about 10 miles long from one end to the other end. That's where there's a commercial fishery available, outside of this 10 mile area, it is closed. This area remains to be open and for the whole Kuskokwim river residents. We have limited it, but all the people can come in even with the declining numbers now. I'm hearing that king salmon is coming this year. I'm looking at it as very low compared to other years.

JCl: Lowest in history.

JC: Under 5000 chum salmon last year.

JCl: The kings met the lower end goal of escapement through aerial surveys, but for commercial, chums last year, chums were 5000 and that's the lowest ever. Goodnews was 500.

JCl: They don't count chum salmon on the river because they change so much, they are like chrome and then spotty, so counters or biologists have a hard time differentiating a chrome chum and a chrome red.

JE: Ohh. But the commercial catches were low last year?

JCl: The lowest on record.

JC: One of the things they complain about is fish counting, there are no weirs, no counting towers, no fish are counted except with the aerial surveys.

JE: And that's concerning to you? You said that's concerning?

JC: It is a big concern because even though the numbers are getting low, we do not have accurate numbers of this resource in Quinhagak. You need that weir so that they can get accurate information.

JE: I see. I have a question about that. Before fish and game was around, how did your ancestors know how many fish were coming back and to not overfish?

JC: They basically can be…they were in Quinhagak, there used to be a hanging net by the two poles, maybe about 25 feet long, between the two poles in the bay. When the water comes in and the fish net is covered up, all the fish passes through the net. When the river goes out, they would take the fish back to fish camp and assume those numbers are numerous and only on high incoming tide is when they could fish. Those are the numbers that they see, and they see they are numerous and are available to catch through the nets. Sometimes in a river like Kanetok, when they swim close together, they are so numerous, the water gets splashed up by their tails swimming together. That tells the number of fish coming in are numerous.

JCl: It’s amazing how they can see fish above the water. I still don’t..I’m still not good at it. But they can just know there’s a bunch and the species even.

JE: So they used, they could only fish at incoming tide, so that was a way to know how many fish but then they also limited and monitored themselves?

JC: Yeah. Very few times some people drifted on the river, but for a very short duration, maybe only 50-100 feet and got a boat load. There were a lot of fish in the river swimming in.

JE: Okay, thanks.

JCl: {Translates question in Yugtun}

JC: Our family had great respect for the salmon coming in. It was basically our only source, an easy source of food for us. You would have to take good care of those. It was so respected that if one fish dropped into a river, they would make an effort to keep that fish from getting lost. This was very important for them, so this is how respected the salmon was by those people. We learned from them that they should take good care of our catch and not let it go to waste. This is the practice we have been doing and we try to protect it by starting early before the rain starts coming in. We tried to dry the fish before the bad season started to come in. Usually late May, early June rains begin to be calm. The water comes down and there’s flowers in the land. So to avoid the drying fish from losing the fish by those wet conditions, they would try to dry them as fast as they can when the sun is up and when they feel rain is coming. They would put them in the smokehouse in good shape. So people have lost the resource because they get wet and spoil. People don’t want the fish to spoil, it’s hard work putting the fish away.

JCl: Quyana. {Asks other question in Yugtun}.

JC: The fish are not being taken care of as good as they used to be. Some people throw what they don’t want to have. We see that in the banks of the river and sometimes along the coast. Some catches are not…mostly chum salmon. They were a popular one that you used to see by the commercial fishery being thrown away because the fish is very cheap to sell compared to reds and silver and king salmon fish. Each time, when the fish is spoiled now, these days, they throw them away and work on another batch of fresh fish after that old spoiled fish has been thrown away. This happens sometimes but it’s not that bad. We see that it’s happening compared to the old times that was avoided when they were taken good care of. That’s what we see. Now we see some of the fish being throw away because there are no dog salmon because of no sled dogs to take the spoiled fish. There’s no more dogs that can use the spoiled fish. I guess there’s no point in keeping that spoiled fish for them.

JE: So that’s partly why…

JCl: And then for commercial, it's because it was the cheapest to sell. It was being wasted on both..

JE: So nobody has dog teams anymore here?

JCl: Translates in Yugtun

JC: No more sled dogs in our villages.

JCl: Remind me to tell you about my sled dog and salmon thing with my grandpa. {Translates in Yugtun}. We had underground storages for the massive chum. I can't believe I didn't think of this yesterday. We would dig a big hole underground and put all the chum in there and it would act as a refrigerator over the years. You open that, crawl in there, get fish and feed your dogs. That's what I used to do with my grandpa. Even to make this underground storage, probably won't work because of the permafrost melt being so extreme, it probably wouldn't even act as a refrigerator anymore.

JE: And people aren't doing that anymore?

JCl: No.

JC: I'm getting a little tired.

JCl: He answered how did your ancestors manage. {She asks the next question in Yugtun}

JC: That's a hard one to answer. Most of the restrictions are imposed by the Alaska Department of Fish and Game, but on the conservative side, I guess the old users were very conservative and the way it was, take what you need and not not take what you don't think you'll need meaning that sometimes a person can overharvest and sometimes nobody wants that overharvested source and it can be thrown away, so they are telling us that you should take only what you need to last for the winter or else the resource will be gone. It's a good idea to conserve and take only what you have to have.

JCl: {Asks next question in Yugtun}

JC: Fisheries management education is going down I think because the old time respectfulness of the resource is declining. The younger generations tend to not pay attention to what they [can't understand] …to the resource.

JE: Do you know what might be a way to try to get the younger generation to better respect the resource?

JC: We can only make an effort to have the parents pass the information down so at least they'll know what was taught and told by the older parents even though the resource is

declining. It should still be reminded by the parents that this resource is important and it is going to last.

JCl: {Translates next question in Yugtun}

JC: We need the weirs to maintain information available so that it can help us decide how to try to manage this resource because now it's open to everyone, it's a concern sometimes that other villages are coming in and taking more than what they should take. Sometimes, I'm hearing that people are using extended lengths of net because there's basically no limitation as to how long this net can be. So other people from other villages might be taking more fish then we normally can take. We need to try and conserve what is available to this community. Again, Alaska Department of Fish and Game doesn't want us to allow us to prioritize our community accessing the resource. They want it to be open to everybody unless it becomes a very serious thing.

JE: Do you think the way, their approach, that management approach, is that working or no?

JC: It's very weak in enforcing. There used to be fish and game person out of Bethel monitoring even the numbers. Looking at how the history…according to the laws. Sometimes, the fishermen may be out of boundary and try to catch other fish even though it's closed. There should be a fine for doing that but I think they don't always know things are being enforced out there maybe because of limited money and resources. I don't know why. They used to go around and check everybody for compliance and check the way we fish. So, I think that needs to be enforced again in Quinhagak because it's open to both commercial, subsistence and sport.

JE: Okay, so better enforcement. Quyana.

JCl: {Asks next question in Yugtun}

JC: At least people know from our own experience, that as we try to conserve fish, they see that. Try to get fish and save fish from spoilage, we try to prevent that from happening. Try to keep the product finished from spoiling. Methods like pulling up…{jegputek],

JCl: Like sheltering the fish, the users how they manage, that's how he is answering the question.

JCl: {Asks the last question in Yugtun}

JC: Well, we know now that the number of fish is declining, and we don't know what is going to happen in the next generation of people. We may no longer be allowed to catch that fish if the number becomes too low. Some of us worry about this and the food source out in the ocean, the 200 mile limit. I guess in the United States, they have control over 200 miles of the sea out there. There are a lot of trawlers out there. There are so many fish they think are not worth taking. I think they should begin to be managed now to keep

things a certain way. We still try, our companies, our people are still trying to make them understand that they are throwing away salmon coming into the Kuskokwim and it is being wasted, and it shouldn’t be. So if there is a way they can try to avoid catching that fish out there, it would help us to keep the fish returning to their spawning grounds in western alaska.

INTERVIEW PARTICIPANT: DOROTHY MARK

Interview Date: 5/12/22
Interviewer: Janessa Esquible
Location: Quinhagak
Home community: Quinhagak

Janessa Esquible [JE]: Quyana Dorothy. I just started the recording and we have Dorothy Mark and Janessa Esquible. It's May 12 and we are in the Tribal Housing in Quinhagak and where are you from, what is your home community?

Dorothy Mark [DM]: Quinhagak.

JE: Are you Yup'ik?

DM: Yes.

JE: K. What is your age Dorothy?

DM: 55

JE: Okay, the first question is can you tell us a bit about your background, you said you're from Quinhagak, how did you grow up and what are your connections to salmon?

DM: I was raised here. It's been my whole, since birth, and growing up I watched my parents doing subsistence, especially subsistence way of life and from them I am able to do now that I have my own family, my kids and my grandkids, I try my best to go with the subsistence traditional, how I was raised, learned from my parents by watching and helping them out.

JE: Quyana. And umm, could you tell us about salmon fishing when you were young and how is it different today?

DM: Seems like back then there were lots of fish, you know. Really big, huge king salmon. Nowadays they are smaller than they used to be. Less chums I think because there used to be lots of chums.

JE: Anything else that is different from when you were younger?

DM: No, but there's not as many fish like there was back then.

JE: Not as many fish?

DM: Yeah.

JE: How was fishing when you were younger? Did you go out with your family?

DM: Yeah, with my dad and mostly after he went fishing I went with him a few times, not all of the time though. I think when they grow up, it helped us prepare for the fish to cut or put away. Not only fish, just you know, everything, meat and all.

JE: So you went out fishing and then you would cut the fish and take care of them and he would help too?

DM: Yesh.

JE: Okay. How did you learn to care for salmon when you were younger? What are some of the values that you have for caring for salmon?

DM: I grew up mostly on traditional, well everyone here I think. Mostly on fish and meat and birds from the land. My parents, mostly from watching my parents and helping. My mom showing me how to take care of them.

JE: Ok, and were there any teachings specifically that you remember about important things about how to care for the salmon or how they managed the fish?

DM: Yeah, see, mostly by verbally, she used to tell me from the beginning how to take care of them as soon as we got them. Not to leave them out, because once you leave out the fish, they get soft and then she said it's more better to work on them right away, while they are fresh. Make strips or suluunaq (salted fish), dry fish.

JE: Quyana. Are the rules that you learned about caring for salmon, do people still follow them today?

DM: Yeah, I do. Most of them.

JE: Most people do and take care of the fish right away?

DM: Yeah.

JE: How did your ancestors manage salmon? You know like before fish and game or before maybe fish and wildlife came around do you know how your ancestors stewarded the salmon?

DM: Stewarded them?

JE: Like took care of them or managed the fishery?

DM: I don't know, but it's still the same, since I remember.

JE: Quyana. Umm, what would it look like if we had Native fisheries management? How would that look to you?

DM: Like what?

JE: Um, you know how, for instance the Alaska Department of Fish and Game, maybe um, there are some people Native working for them but maybe many not Natives, so how would it look, like for you, your ideal Native fisheries management? The question is like what does Indigenizing our ways of knowing about and managing salmon look like for you?

DM: I don't know.

JE: That's okay. We could come back to that…I'm trying to think..

DM: How to manage them? How did they manage them?

JE: Yeah, you know how umm how there are restrictions, regulations, things like that, how would, I'm wondering, how would it look if we Native management of a fishery? If just the people or just…

DM: Oh yeah, that to me would be good?

JE: To have people from here..or? Have people from here managing?

DM: Yeah. Back in the old days, with no restrictions.

JE: No restrictions?

DM: Yeah.

JE: Umm, do you feel like your values or your knowledge, is it reflected in management today? Like you know how you were saying to take care of the fish and put it away immediately, do you think the managers know about that? Or do they consider that?

DM: Yeah, most of them know.
JE: They do?

DM: Yeah, they are making sure to help me with subsistence (referencing her relatives and people of Quinhagak, not western management). I learned from my mom and now I am teaching my kids and grandkids how to manage or take care of them.

JE: Awesome, so they are learning too?

DM: Yeah.

JE: Do you think your knowledge and what you are teaching your kids, do you think that fish and game or other management, do they consider that? Or no…?

DM: I don't think so.

JE: You know what I mean or no? Maybe I'll come back to that one. What concerns or worries do you have about the salmon fisheries today?

DM: I have no worries I think.

JE: No worries? Okay. I know earlier you said that the fish are getting smaller. Is that a concern or no?

DM: It's not a concern to me. What concerns me is seems like last year out of all the fish we caught or my sons caught last year, I think I cut only two chums.

JE: So the chums worry you?

DM: Yeah, the chums are gone.

JE: Do you know what might have happened?

DM: No.

JE: Wow.

DM: But from the beginning my parents used to tell me if we don't take care of fish like we are supposed to and damage them or throwing them away, they'll get lesser and lesser and from the prior years or when they used to commercial fish, there used to be lots and lots of chums, I know. Even upriver when we go upriver, we'd see lots of chums on the sand bars, and I know in some meetings we used to say or told them there is some people that need fish instead of throwing them back we can give them to the people that have no way of getting them. If it's…instead of throwing them back or, because I know a few people have said it, but I haven't seen it..throwing fish, even to the dumpster over there, like trash.

JE: And that's from the commercial fishery, you saw all the chums and they didn't want them so they threw them out?

DM: No, just from commercial and sports.

JE: Oh, sports too?

DM: Yeah, for sure sports too. Even people from here started complaining when they go upriver on the shore there would be chums here and there.

JE: That they threw out?

DM: Yeah. On the sand bars?

JE: Is that a concern for you? Do you worry about that?

DM: {can't understand} I don't know about it nowadays.

JE: Yeah, okay. Are there any concerns the way the fishery is managed, subsistence or commercial?

DM: No, I don't think so.

JE: Is there anything about fisheries management that you think is working good? Like, what do you think is going well about fisheries management right now?

DM: You mean on subsistence?

JE: Yeah, subsistence, commercial, sports…

DM: How they manage them?

JE: Yeah. Like what's working well and what's maybe not working well?

DM: I don't know. I think they are managing them good because they work on fish as soon as they catch them.
JE: The people?

DM: Yeah.

JE: Do you know the Alaska Department of Fish and Game or no?

DM: Not too much.

JE: That's okay. And, what do you want to see for salmon and people in your region in forty years?

DM: I wish there would be fish to manage.

JE: Anything else?

DM: No.

JE: Okay, do you have any questions or anything else you want to share?

DM: I hope managing fish will keep on going (Native management), even to our younger generations, like we are today, feeding their families, preparing for the winter. I hope they don't quit even…cuz' I know there's some families that are hardly doing any…like we do, preparing for winter. I hope it continues for our younger generation.

JE: Okay. Do you know why maybe the families are not putting as much food for the winter?

DM: Mostly, the way I see it is because their older generation is being gone. I know some are not really in it because of that. But me, I try and encourage even show the few families how to cut fish and take care of them and now they are able to do them themselves. To be able to prepare and take care of fish.

JE: Wow, me too. Quyana for sharing.

DM: Yeah, that's it. Today they are able to do it and I'm very happy when they thank me for it and now they are able to cut fish. They'd call me and let me see their fish and then when they are smoking or they are ready to take down, so some…I know there are like three families that are able to do it now.

JE: Wow.

DM: And I hope my kids will be doing that too and to the younger generations too, when it's time for them?

JE: To live the subsistence way?

DM: Yeah.

JE: Quyanaqvaa. I don't have any other questions here. Yeah, I really appreciate your time Dorothy. Thank you sharing. Should I stop recording?
DM: Yeah.

INTERVIEW PARTICIPANT: LUCILLE MARK
Interview Date: 5/12/22
Interviewer: Janessa Esquible
Location: Quinhagak
Home community: Quinhagak

Janessa Esquible [JE]: So this is Janessa Esquible with Lucille Mark and it's May 12. We are in the Tribal Housing in Quinhagak. And where are you from?

Lucille Mark [LM]: Here, Quinhagak.

JE: You're Yup'ik?

LM: Yes.

JE: Do you mind telling me your age?

LM: 57.

JE: Quyana. And we'll begin with the interview. Thanks for joining us. The first question is can you tell me a bit about your background, you said you're from Quinhagak. How did you grow up and what are your connections to salmon?

LM: Umm, from an early age my parents would, I would follow my parents to the fish camp and I would help my dad with that wheelbarrow and bring up really big king salmon for my mom, where she cut and hung and made stinkheads. Well, I grew up that way, going by wooden boat to net in the river for trout or any species of fish, there were a lot and mostly humpies I remember.

JE: Oh a lot of humpies?

LM: Yeah, there used to be millions jumping in the river close by. We would go salmon berry fish camping to Quinhagak and Eek. Is this just about, what was the question?

JE: Oh yeah, just how you grew up. You grew up spending a lot of time on the river fishing, berry picking?

LM: Yeah, we did all that. Yeah, we went all over here and even towards the mountains for black berries, camping and all over, even in the…for king salmon, but there's hardly king salmon anymore.

JE: That actually goes into the next question on how was salmon fishing when you were younger and then how is it today?

LM: In no time at all, when I was in my early twenties, when the tide came in I would go out and I would try to limit 15-20, I would just set out maybe just about, not long…hundred

feet and right away I'd start catching kings. We'd have to pull in before I even finished putting all my net out, I'd have to pull really fast in or I'd over catch. And then today, it takes longer for my husband to go out and try and get fish for us to hang. Like this past summer, it takes a couple hours or so to even fill that fish bin. It takes much longer and less kings, and smaller kings. We hardly see those huge kings anymore and they come…last year they came in really late and we were cutting mostly reds and chums. I put away only one or two kings last year because there was hardly any last year. They're…I don't know every year it's beginning to be like that.

JE: Really? And you said they came in late last year? Have they been coming in at different times?

LM: Usually towards the end of May we start catching or even sometimes middle of May, but last year it was like past toward the middle of June, even this year, the smelts and the trouts were late. We went out ice fishing in November and we used to catch lots of smelts but this year they didn't come around til' maybe February. Even the trouts.

JE: Thank you. Okay. Any other differences from when you were younger to today?

LM: Umm, our fish were pretty big and healthy back in the day. Nowadays some of our catches are really skinny and they have something on their skin and even when you cut them up there's like discoloration in the meat and even when you fully cook it, it's like it's not cooked well, even if you cook it like you normally cook. When I boil fish, I cook them for like 20 minutes and then when we eat them they are still…somehow the meat is not firm and like fresh tasting, but seems like they have less taste like a mushy meat or something like..it's not cooked.

JE: Oh, and when you were younger you didn't really see that?

LM: We never saw that. But now, we wonder if it's some kind of contamination in the ocean if it's something that's causing these fish to be like that.

JE: Oh, thank you. When you were growing up, how did you learn to take care of salmon? Like what kind of lessons or teachings were important for you when you were caring for salmon?

DM: I followed my parents when they went to the net for fish or even with the…we didn't have rods then so we just threw them in the water, like this…and pulled our lines like that. And then I'd watch them how they cut the fish and I start volunteering to do it, like before I was a teenager, so I start helping by heading and gutting while they are making fish to hang. So I watched and followed my parents from a young age.

JE: Are there any rules that you learned when you were growing up on how to take care of salmon? Like did they share anything that was really important for you?

DM: We were taught that if we hang our fish to dry and the guts and heads had to be disposed of in like the flowing water, not just anywhere on the ground. They would tell us that if we just leave any or if we have too much to give them away to give them to people that wanted them, not to waste because they told us that if we waste food too much in the longer…later in the years…there won't hardly be any because they said kelgaq, what's kelgaq in english? The kelgaq is long for…you know if you mishandle or waste fish too much or any kind of food, they know like…they know if people are wasting too much, they won't come around for a long time or hardly be any, like that if you waste. That's why we are always told to properly dispose of things like even animal bones…to dig 'em' in the ground and not just toss them around all over the ground. To respect the animals that we have so they won't lose them because of wasting.

JE: Quyana, umm, are these rules still being followed today?

LM: Hardly anymore. We see people just trash their leftovers or whatever, just anywhere.

JE: How come do you think?

LM: They know but I guess laziness is what I think. They just don't respect what they are told to do with how to treat their catch. I don't know but we see a lot of that today.

JE: Really, and you never saw that when you were younger?

LM: No hardly no, I didn't.

JE: Wow. Okay. And I think this next question you spoke to already, how did your ancestors care for salmon, and you just told me all of those things.

LM: Yeah.

JE: What does…this question is a little hard to phrase…what does Indigenizing our ways of knowing about salmon look to you? So like, if we had Native fisheries management, what would that look like? Umm, like Native-led management or Indigenous fisheries management?

LM: I think the people that actually do subsistence and that know how to hunt and gather and fish should write how to do all of that instead of outsiders doing or telling us you need to do this and that. They need to have people that actually do it to do all that for future generations.

JE: I see. And, would you want people like in Quinhagak, you would want people from here who are subsisting and going hunting and fishing to do that?

LM: Yeah.

JE: Okay, awesome. Umm, do you think, you know you were talking about outsiders who manage the fishery. Do you think that they manage based on your values, like teachings that you know? Do they consider that?

LM: Mmm, I guess in a way, but like they shouldn't limit us to a certain time frame of the year to get what we need, like I said this year, the trouts and smelts and even last year, the kings and fish were late coming. We were hardly catching any at the times we usually do, so I don't like how they time frame us for us to do what we need to gather and stuff like that, so it depends on nature, not on the paper, how they think we should, the times they think we should do it or how we should do it. I don't like that so…

JE: Really ok?

LM: It depends on nature how we can get to whatever we need to store for the winter.

JE: Do you think that they are setting these time frames because they are outsiders and not from here and they don't know what's going on?

LM: Yeah. What we don't like here, try to manage our way of living without knowing our environment.

JE: Wow…okay, quyana. Umm, what concerns do you have about the fish today? I know earlier you talked about there is less, they are smaller, disease. Are there any other concerns that you might have?

LM: Umm, I can't think of any right now.

JE: None right now?

LM: Yeah.

JE: Okay, if you think of any let me know. What are…you mentioned already this next question says…what are some concerns you have with fisheries management? You said earlier that they are not limiting you to this time frame, that's not working. Is there anything else that you feel like is not working about fisheries management?

LM: The limit that we could get. Because some of us have larger families or we share with families that can't go out and get them themselves, so, yeah that's one thing. {speaking in Yugtun with Jacki Cleveland}.

JE: Is there anything you think that is working good? Anything that you like about the fisheries management approach today or no?

LM: I don't like when at the time to they seem to favor sport fishermen and guides over our needs when I remember one year we hardly had any fish coming in and they told us we can't net fish to hang but only fishermen and sports fishermen and guides were

allowed and that’s what we don’t like. If they are going to say we can’t get food or fish to eat they shouldn’t allow outsiders coming in to do it other than the people that actually live here.

JE: Wow. So they are letting people from…they’re prioritizing people not even from here over you and your family?

LM: Yeah, yes. That’s what I don’t like. All of us probably don’t like it here.

JE: Thank you for sharing. This last one is how would you…one more after this. How would you improve these things? I know earlier you said you want people from this region to be managing and basically leading that?

LM: Yeah.

JE: Are there any other ways that you would improve?

LM: I don’t know. I guess I have elder input and you know people…community input on what they write so at this point I don’t think…I don’t know.

JE: Okay. Quyana, I wrote those things down. Appreciate it. This is the last question. What do you want to see for salmon and people in this region in 40 years?

LM: Well, traditionally we shouldn’t be limited to what we need. People should be allowed to gather as much as they need like I said earlier so I hope no more restrictions are put in to try and limit what we can take because that’s our main source of food and many of us aren’t employed. We can’t afford to keep going to the store if prices keep skyrocketing so I think in 40 years they shouldn’t prohibit or make laws for people not to go out and get what they need from the land.

JE: Anything else you want to share before we end?

LM: No, I don’t think so.

JE: Okay, quyanaqvaa for your time today. Really appreciate it. I’m going to press stop on the recording.

INTERVIEW PARTICIPANT: STANLEY BERLIN
Interview Date: 6/16/2023
Interviewer: Janessa Esquible & Morgan Urquia (MU)
Location: Fish Camp
Home community: Kasigluk

Beginning of Recording.

MU: And the light to come on

JE: Okay, should we do a test or you think it's good?

MU: Um the best is to do introductions in the beginning but no a test, it's good.

JE: Perfect. Okay, quyana Stanley Berlin for your time. We're at your fish camp and it's June 16, and we're here with Morgan as well. Um, do you want to tell us a bit about your background, where you're from and how you grew up and what are your connections to salmon?

SB: Well glad to have you two here and glad you could spend, spend some time here at fish camp. But yeah I'm from, I'm Stanley Berlin, I'm from Kasigluk. And for as long as I could remember. Even my mom told me to tell you for as long as she could remember, she's always gone, they've always gone to fish camp.

JE: Mm.

SB: And uh, yeah we'd go as an extended family. Our grandparents would go and our family would go and other in-laws. We'd all camp in the same area.

JE: Mm.

SB: And um, we'd leave like right after smelts go by.

JE: Mm.

SB: Get camp ready and make sure we're ready for the arrival of kings.

JE: Mm.

SB: And there was always an abundance of kings. And we saved everything, even the backbone, smoked them, dried them, smoked them all and put 'em away. And um yeah for as long as I could remember we've always been at, going to fish camp. And my dad passed away eleven years ago. He established this fish camp. 1990. And we been coming every year.

JE: Mm, wow.

SB: But um my mom didn't come for two years after my dad passed away cause she was grieving and [sighs] yeah, just grieving and finally she de-, she asked if I wanted to bring her here so she could clean her house.

JE: Mm.

SB: I said yeah while we're doing that I could tear down the old fish cache and fish rack and build you a new one, so during that time I tore down my dads old fish rack and built her [a] brand new one and from then on, nine years we've been

JE: Wow.

SB: coming to fish camp.

JE: Mm-hm.

SB: And if I don't take her she won't come

JE: Mm.

SB: because she don't like to repeat herself

MU: [laughter]

SB: When she asks for something I stand up and do it.

JE: [laughter] good

SB: So yeah, she's depending on me.

JE: Mm.

SB: And she's getting old right now and she didn't wanna come down early because she's old and it's been cold [sighs] and not too many fish running right now so she's kinda wait-, she wants to come and I hope she does and I hope the fish arrive too.

JE: Mm-hm, yeah.

SB: And she's also afraid of that Donlin, Donlin Gold mine.

[03:27]

JE: Mm.

SB: She said if the dam bursts all that toxic stuff flows into the river, it could flow into both the Yukon and Kuskokwim side where they're located.

JE: Mm.

SB: She's afraid the fish will die off and she depends on salmon. She grew up having salmon.

JE: Mm-hm.

SB: We have some every year and I come from a big family. And they used to store, cut lots, lots of salmon.

JE: Mm. Hm.

SB: My dad had dogs too and they late, late in the uh, chum run toward the end of the chum run, they filled the whole fish rack with chums for dog food.

JE: Wow. Yeah, that actually was my next question is how was salmon fishing when you were younger, you were just describing that some, and how is it different today?

SB: Oh we, we could use any kind of gear, king gear

JE: Wow.

SB: to whitefish net depending on the size and what we're targeting.

JE: Mm-hm.

SB: Yeah, and um, yeah, we'd, we'd wait for a good day not on a rainy day like last opening.

JE: Mm.

SB: Windy, raining, miserable day. Now [breathes in deeply] we go whenever there's an opening. And we have no control over that and if there's no fish on the river, too bad. And uh people from back home it's um, like 25 air miles

JE: Mm-hm.

SB: But by river it's more like uh 35 to here, 35 miles by boat. And I burn like 20 gallons if I decide to go fishing from back home to here and my mom would rather move here and fish.

MU: Mm-hm.

SB: Where's there smoke wood available and all the resources are here.

JE: Mm-hm.

SB: To take care of the salmon.

JE: Hm, and you said you used to get, put away a lot of fish.

SB: A lot of fish

JE: When you were younger. How about today?

SB: Today we're limiting ourselves, since my mom's getting older and uh a shorter window frame to dry them.

JE: Hm.

SB: And smoke them. Everything is seasonal here. When the salmon berries are gonna be ready we hurry up and smoke 'em and if I could I'll smoke them twenty-four hours if we're in a rush to smoke them, cure them, dry them and head back home for the next seasonal.

JE: Mm-hm.

SB: then we go after like salmon berries.

JE: Gotcha. And when you were younger how did you learn to care for salmon?

SB: Oh watch and learn, that's how we learn. Like um I cut salmon yesterday, I learned just from watching my mom and I asked my daughters did your mom ever teach you how to cut whitefish cause I watched her cut fish all my life.

JE: Mm.

SB: And then [plane flying] they asked me "do you know how?" "I think I do. I watched my mom cut fish so let's set a net and see how it goes step by step."

JE: yeah [laughter]

SB: I caught my first salmon, I mean my first whitefish. Ah, shook it and my daughter said so perfect.

MU: Mm, wow.

SB: So, yeah, watch and learn.

MU: Hm.

SB: And yeah my dad took us, my bro-, my brother went out fishing, commercial fishing and subsistence fishing when we could barely pull the net in and barely pull off the kings and fish.

JE: Wow.

SB: So we were introduced to fishing at a young age.

JE: How young?

SB: Mm, for as long as I can remember [chuckles]

MU: Mm-hm [laughter]

JE: Wow.

[07:44]

SB: And the salmon we used to have Sunday two hour commercial fishing, commercial fish openings.

JE: Wow. So were there any rules like about harvesting or sharing when you were younger that you learned from your mom or others?

SB: Harvesting there was you catch what you need, not what you want.

MU: Mm.

SB: So, and depending on your family size too and if you have dogs too back in those days.

JE: What about so, you catch what you need to feed your family and were, did you have to support other families or you know community members, like earlier I remember you said you shared your first fish with an elder and your mom.

SB: Yeah, my mom always tells me that um if, even if I set whitefish net, your first catch give ‘em all away.

MU: Mm.

JE: Mm.

SB: And my first I caught only one king salmon that set net opening they had and I gave it to my mom and

JE: Mm.

SB: my mom told my baby sister Ronda, she does most of the work and my mom asked her “cut what you need, clean them, cut them up and give them away to, give them away to anybody you want.” And my sister said, “no, we’re keeping the rest of them.” [chuckles]

JE: Oh she did?

SB: Yeah

JE: What’d your mom say?

SB: She whispered to me about it.

[group laughter]

JE: That’s what I was going to actually ask you next, how were rules enforced like if you didn’t follow the rules of sharing or harvesting only what you needed?

SB: Yeah we made sure we didn’t waste anything.

JE: Mm.

SB: And I come from a big family. I have six sisters and four, four brothers and my mom she used to, they used to hang and dry lots of fish here and still to this day my mom sends dry fish to my sister in Anchorage and my sisters in Bethel.

JE: Wow.

SB: So

JE: That’s awesome.

SB: It’d be nice to have them come in and help once in a while.

MU: Mm-hm.

SB: [laughter] but uh yeah, they’re, one of my sisters she had someone build a fish rack and a smokehouse for her and my other sister Catherine she um, she wants a fish rack and she wants to come and help when the timings right, so they’re welcome.

JE: Awesome.

SB: We appreciate that.

JE: Yeah.

SB: And I don’t mind sharing

JE: Mm.

SB: Last year my son came down cause of my health issue and um they cut like, cut fish like only a couple days and then they had to go back but um my mom and I we split everything in half.

JE: Mm.

SB: And when I got home I shared my son and his family. Gave ‘em half of their, half of my fish. So I don’t mind sharing.

[11:19]

JE: Wow. Do you feel

SB: And it’s, and it’s better to eat everything, run out of everything so you can fresh everything.

MU: Mm.

JE: Mm.

SB: Upcoming season.

MU: Mm-hm.

SB: Makes you work harder too [laughter]

JE & MU: [laughter]

SB: And look for, look more forward to it too.

MU: It helps yeah.

SB: Yeah. My younger brother Dan junior he’s supposed to be working right now. He just got hired at the local store and um fishing right now, fish camp’s more important to him right now.

JE: Mm-hm.

SB: He’s on probation but he said, “the heck with them, I’m going, we’re going.”

MU & JE: Mm.

JE: Yeah.

SB: They, he, he shares his family too.

JE: That’s awesome. Do you feel like sharing and people taking only what they need and not what they want like that’s still pretty strong in the community?

SB: Oh yeah.

JE: Awesome, and um, how did your ancestors do you think, how do you think they managed or stewarded or cared for salmon before there was any fish and game or fish and wildlife, before any of these other agencies came around?

SB: Go with the season.

MU: Mm-hm.

SB: Just like egg hunting.

JE: Mm.

SB: When the geese, ducks lay eggs, they go out and collect them and once they have the embryo growing, they quit collecting eggs.

JE: Hm.

SB: They manage themselves but yeah, they had less access to the stores in Bethel.

JE: Hm.

MU: Mm.

SB: We had no local stores in the village when the elders were back home, when the elders were alive back then.

JE: Mm.

SB: And they’d make sure they had enough of everything and they’d, if they go to Bethel to shop, they’d buy everything in bulk.

JE: Mm.

SB: Commercial fishing, trapping, fur trapping.

MU: Mm-hm.

SB: Hardly any jobs.

JE: Wow.

SB: So it was a lot tougher and it was more, more important for them to have dried white fish back then.

JE: Mm-hm.

MU: Mm-hm.

SB: If they run out, they had no store to run to.

JE: Wow.

SB: And now with the economy going up and down so bad and if they hadn't raised the debt ceiling.

MU: Mm-Hm.

SB: The government would have probably shut down and like food stamp benefits, I think they're planning on cutting those completely.

JE: Mm, wow.

SB: Not too long from now. I don't depend on that stuff.

JE: Wow. What do you wish that fish managers knew about your community and fishing practices?

SB: We know what we're talking about. The fish aren't here because everybody knows, even our elected officials know and they created a monster when they established the north pacific marine fisheries council [north pacific fishery management council].

JE: Hm.

SB: The state fish and game, the fish and wildlife, they have no control over them, they manage their own fishery.

JE: Hm.

SB: And their council is comprised of there are no, no local, I think they have only one local member

JE: Mm.

SB: on the council as a consultant for information and he has no voting power.

JE: Wow.

[15:22]

SB: I don't know any of them by name, I don't know if any of them are from this region. I think they're all from outside and the governor appointed them. Governor and Ted Stevens and the late Don Young. They established all of that.

JE: You think that's a problem?

SB: Yeah, they created a monster, yes.

JE: Wow.

SB: Like we been trying to have fish and game manage or cut down the intercept fisheries in Area M and the uh pollock fishing industry out in the Bering Sea but we have no power, we have no say in that, so we're pretty much stuck here under regulations and there's a incidental bycatch number for Chinook only.

JE: Mm-hm.

SB: There's no incidental bycatch limit for chums and sockeye

JE: Mm.

SB: and silvers. And they catch halibut too.

JE: Mm-hm.

SB: The coastal villages, the coastal village they had their um, they were buying fish and they set up small fish plants all the way up and down the coast to harvest salmon and halibut.

JE: Mm-hm.

SB: And a lot of coastal village people, they were doing pretty good on halibut fishing.

JE: Hm.

SB: Catch like, maybe make like a hundred thousand dollars at most in the season, but um that, that got pulled out from under the rug.

JE: Hm.

SB: So, and we can't commercial fish anymore.

JE: Mm-hm.

MU: Mm.

SB: There's a problem but it's not originating from here.

JE: Yeah.

SB: It's out in the Bering sea. Because ever since they started trawling out in the Bering sea. It was in the 80's and god knows how, how the, salmon were probably more abundant out there then they are right now.

JE: Mm-hm.

SB: They were dumping them over, still dumping them over.

JE: Wow.

SB: So after a few years they, in the 80's the salmon numbers started declining after 1988 that was the strongest year. Strongest um salmon run

JE: Huh.

SB: on record. Even in Bristol Bay, but um, after that everythings been going down hill, down hill, down hill. [long sigh]

JE: Oh. Do you think, do you feel that your knowledge, your values are reflected in fisheries management?

SB: Absolutely not.

JE: Mm.

SB: They turn a deaf ear. But um, I think the organizations are starting to voice themselves.

MU: Mm-hm.

JE: Like which ones?

SB: Like ONC and City of Bethel and other organizations like what's the man, Arctic, what's that uh?

JE: ICC, or?

SB: Yeah. I'm glad they're pursuing to have more management over the intercept fishery and the pollock industry.

MU: Mm-hm.

JE: Um,

SB: They, they have a strong voice. Them Area M fishermen.

JE: Hm.

SB: Because one fisherman, one salmon season, they make up, up to a quarter million dollars, only one season to over a million and the trawlers they like over millions and millions of dollars.

JE: Do you think they have a stronger voice?

SB: And they have a stronger voice.

JE: Hm.

SB: And they probably have, have our elected officials in their pockets but they, because they have been ignoring the situation.

JE: Mm.

SB: In the YK Delta, salmon crises we're in right now.

JE: Mm-hm.

SB: Money talks and they listen to money. I'm a commercial fisherman and I don't make hundreds of thousands of dollars. I'll be lucky, I was lucky to make like over ten thousand in a season.

JE: Wow.

SB: But I had to travel long distance. So I, I had um, lots of expenses.

MU: Mm-hm.

SB: Just to go fishing in Quinhagak or Goodnews Bay. So the rivers flowing the wrong direction [chuckles].

MU & JE: [laughter]

SB: All the moneys staying out there somewhere.

MU: Yeah.

SB: I think since, since the organizations are trying to sue the government and whoever's managing everything there trying to sue them under subsistence perspective.

MU: Mm-hm.

SB: I think it's time all the commercial fishermen from the Kuskokwim and the Yukon got together and put a dollar value on all the incidental bycatch fish they're dumping over the side.

MU: Hm.

JE: Mm.

SB: Put a dollar value over that, that, and sue them for how much, every year, every season.

JE: Mm.

MU: Mm.

SB: Put a dollar value on that.

JE: Mm-hm.

SB: And let them pay for it.

MU: Mm-hm.

SB: Cause I'm, I can't even commercial fish right and I'm a permit holder.

JE: Wow. [birds singing]

SB: I think it's time, I think that's the next step. I'd like to bring up with whoever's willing to listen.

JE: Yeah.

SB: Bring up a civil lawsuit.

JE: Yeah civil lawsuit. Quyana

MU: Yeah.

SB: For the commercial fishermen, put a dollar value on it, maybe they'll listen to the dollar signs.

MU: Speak their language.

SB: Yeah.

JE: Yeah.

SB: Cause that'll be actual number of fish, actual number of pounds and actual co-, cost.

JE: Mm-hm.

MU: Mm.

SB: What they're dumping out.

JE: Yeah thanks for sharing. That's a good suggestion. Wow. And you mentioned you have concerns about the mine, concerns about the trawlers, Area M, what other concerns do you have about salmon fisheries today? Or worries?

SB: Well, my mom's been saying she, she's a smart old lady. If she's been, she told me how many times ever since she heard about that mine and all the tox, toxins they're gonna leave behind and she, she, she's been telling me um if that holding dam breaks, holding pond breaks, gets dumped into the river we won't be able to even drink it.

MU: Mm-hm.

SB: And the salmon won't even be, they'll be poisoned.

JE: Mm.

SB: Everything will be contaminated from the mine on down.

MU: Mm-hm.

SB: Mine and yours [laughter]

MU: [laughter]

JE: Man.

SB: So that’s the god awful truth.

JE: Yeah.

SB: But then again money talks.

JE: Hm. Is that one of your concerns with fisheries management that you feel like, you mentioned earlier Area M they have a strong voice cause they’re bringing in more money.

SB: Oh yeah.

JE: Is that a concern that fisheries management is that way or?

SB: Oh yeah and if there’s any disaster relief moneys cause if there, if they’re gonna cut their fishing season or even close ‘em down they’re gonna cry for lots of money.

JE: Hm.

SB: Even the trawlers.

MU: Mm-hm.

SB: And where the money comes from the government and the state. They’ll be more than happy to hand them millions of dollars.

JE: Mm.

SB: The last time we had disaster relief money for the uh Kuskokwim fishermen, my brother Dan junior applied and he got a whopping thirty-six dollars.

JE: Wow.

SB: And uh, a big part of our disaster relief money went to the universities for studying.

JE: Wow.

SB: Redirected.

MU: Hm.

JE: Do you know whatever came of any of those studies?

SB: Mm, no. They need more money to study more [laughter].

[group laughter]

MU: That’s very convenient.

SB: They know the answers but uh they want more money too [laughter]

MU: It's true.

JE: Jeeze. Do you feel like anything is working well with fisheries management?

SB: Not right now.

JE: Not right now, how about in the past?

SB: In the past there were enough fish that they weren't being dumped over side and we had strong runs. I think the major impact is from the trawlers out there.

JE: From the trawlers?

SB: Yes and from the past experience um area M opened for coho season because the kings were still running and they had a late, late opener in area M. And that season we had lots of reds not too long ago maybe, twenty, 2019.

JE: Hm.

SB: There were lots of reds in August silver season, I came down to fish for silvers I set my net it was, it smoked, caught, filled the tote one set.

JE: Wow.

SB: I pulled in the net there was, there were nothing but reds. And that year, 2011 too, when my, eleven years ago, when my dad passed away we were catching reds all the way up in the tundra area and them lakes back there.

JE: Mm.

SB: And that's not a salmon tributary.

MU: Hm-mm.

JE: Wow.

SB: So that year there was, there were a lot of reds because they had a late opening and the Area M so.

JE: They passed through?

SB: Yeah.

JE: What, what other improvements would you do to, for like, I guess to improve fisheries management since it's not working today, you said to definitely regulate and focus on the trawlers.

SB: Yes.

JE: Anything else that you would do to make improvements?

[27:17]

SB: [big sigh] well the Kuskokwim is considered a chum fishery.

JE: Mm-hm.

SB: We used to have nothing but chums, lots of chums and silvers and kings, but um, now the reds are more abundant and they're running right now. And in July one of my friends from Bethel, he drove from Bethel all the way up to Aniak when the um red, during our peak reds, peak run of red salmon and he saw jumpers from all the way from Bethel to Aniak. All the way up the river.

JE: Wow.

SB: So I'd co-, I'd like them to consider changing the Kuskokwim from a chum fishery to a sub-, red salmon fishery.

JE: Mm-hm.

MU: Yeah.

JE: Thank you. Um, what do you want to see for salmon and people in your region in the next forty years?

SB: I'd like to see salmon [laughter] period.

JE: Yeah [laughter] salmon.

MU: [laughter]

SB: Yeah and um fish and wildlife, and fishing st-, state department of fish and game they'd still have a job.

MU: Mm-hm. Yeah.

SB: No salmon, no job.

MU: That's true [laughter]

JE: Oh I had another question too, do you feel like the uh, is the fish commission. You mentioned a little bit about them earlier? Are they helping any to have more of a local voice in management or do you think they have any control in management or not really?

SB: Not, I don't, I don't fully understand the um, their management strategy.

JE: Gotcha, gotcha.

SB: But um the state and the US fish and wildlife, they have no control over them right now [ocean fisheries].

MU: Mm-hm.

JE: The, oh um, out in the ocean?

SB: Yes.

MU: Right.

JE: Gotcha. Do you feel like they have a lot of control on the river?

SB: Oh yeah, that's their job, they're here.

JE: [chuckles]

MU: That's their [chuckles]

SB: Planes with pontoons fly back and forth everyday

JE: You see 'em both the feds and states?

SB: Yeah, well feds right now.

JE: Oh ok feds right now?

[side conversation with relative]

[30:18]

JE: Quyanaqvaa for sharing. Is there anything else you want to share?

SB: Oh I'm glad you guys came and enjoyed fish camp life for a night. It was nice having you two in and good to see someone experience what we go through every year.

JE: It's been wonderful being here.

SB: For about a month. We used to camp a lot longer til August.

MU: Hm.

SB: But um now it's cut short and even silver season looks, looks bleak right now.

JE: Mm. You were saying it's cut short because uh the people aren't making, they don't have as much money to stay for the whole summer or are there other reasons?

SB: Well uh regulations and um we usually like to dry our salmon by the end of June.

JE: Gotcha.

SB: Or first, end first week of July.

JE: Mm-hm.

SB: Because traditionally July's a wet month.

JE: Mm-hm.

SB: And the flies come out and it's a lot warmer and so yeah, right now there's we hang the fish flies won't claim them. [laughter]

MU: Mm-hm.

JE: [chuckles]

MU: I wonder what you've noticed about the river, changes to the river, whether that's the water and the land?

SB: Oh sand bars, they change constantly.

JE: Mm.

SB: And the channels changing as, every year too even for, I haven't gone down river in, in some time but when we used to go fish in Quinhagak I could travel even at night.

JE: Mm.

SB: With a little assistance from a GPS [laughter]

MU & JE: [laughter]

SB: But yeah, out channels are changing, I've been, I drove my boat down there and I've, I've hit uh, sand bars or shallow where there's, where the channel used to be.

JE: Hm, wow.

MU: And the water, have you noticed any changes in the water?

SB: Well right it's uh silty.

MU: Mm.

SB: Because, the uh snow, lots of snow in the mountains up tribu-, upper tributaries of the Kuskokwim.

JE: Mm.

SB: We had lots of snow. There's lot, high water too even back home.

MU: Mm-hm.

SB: And usually high water all over.

JE: Wow.

SB: But it's slowly receding right now but seems slower on the Kuskokwim.

MU: Mm-hm.

SB: And the current's strong even right up til high tide in this area. The usually, the current's coming in.

JE: Mm.

SB: When the tides coming in but right now it’s just at a standstill when the tides coming in.

MU: Mm-hm.

MU: And the uh, you were talking about wind direction. Uh have you noticed any changes in the winds?

SB: Oh the storms are definitely getting uh stronger.

JE: Hm.

SB: We, I haven’t heard of atmospheric uh rivers even before when was last June or?

MU: Yeah.

SB: Murbock and I, I see more of that on uh weather maps atmospheric rivers. Um, rainfall that look like, it looks like a river, just a straight line of super heavy wind and rain.

JE: Wow.

SB: Yeah storms are on the increase, and nice to have nice weather once in a while [laughter]

MU: Yeah [laughter] ____ [34:38]

JE: Yeah, it is, it’s nicer out there. [chuckles]

MU: Mm-hm.

JE: Anything else you wanna?

MU: Um, how, how are young people learning to fish now a days?

SB: Well, my nieces and nephews, they come to fish camp.

MU: Mm.

SB: They love it here.

MU: Mm.

SB: They don’t mind helping out doing chores and they, they won’t say no if I ask them to come along when I go fishing.

JE: Wow.

SB: Last year when I was in the hospital, admitted in the hospital for four days, my, I think my oldest nephew was like 14 or 15 and they asked, they asked me if um they could go fishing by themselves. Absolutely, I let them drive and I let them set nets and I let them clean the net. They knew where to fish and they know where to fish and they know how to read the tide and they knew the channel, so they went out, they were on facebook last year. A young fishing crew.

MU: Mm.

JE: That's awesome.

SB: Yep, I was real proud of them [tears up].

JE: That's awesome.

MU: Yeah.

JE: So good to see. Wow.

SB: Yep [crying], yep all that hard work paid off.

MU: Mm-hm.

JE: Wow.

SB: Good to see that.

MU: Mm-hm.

JE: That's amazing.

SB: Yep. I'm happy for whoa. [laughter] lights came on [laughter]

MU: [laughter]

SB: Yeah, we're, they love it here. And uh, my two nieces and nephews, my baby sisters um two kids yesterday, they were excited and they were, they start asking, "are we gonna go to fish camp?" in the middle of winter? [laughter]

MU & JE: [laughter]

JE: Really excited [laughter]

SB: And I'd tell them nine of clock in Ju-, sometime in June [laughter]

JE & MU: [laughter]

MU: They're ready.

SB: Yeah [laughter]

JE: Wow.

SB: Yep. Yeah, now a days we didn't even think of bringing freezers down here.

MU: Mm.

SB: Now we have to pack like birds and meat and dry fish and [sighs]

MU: Oh why is that? Why did that change?

SB: We, you want to go fishing on the Kuskokwim right now? [chuckles] It's closed.

JE: [laughter] you have to bring your own food.

SB: Yeah.

JE: Man.

SB: And when we run low we have to either, either have to drive up back home get some more frozen food from back home and drive back [short sigh].

[37:54]

JE: Hm. Wow, so a lot more work on you?

SB: And the fish are running, yeah.

JE: Wow.

SB: Fish are swimming by.

JE: Mm, yeah.

SB: And that

JE: What’s that a couple hour trip for you?

SB: Yeah, hour and a half one way.

JE: Wow, just so you can come and stay here.

SB: Yeah.

JE: Mm.

SB: But I have a nice refrigerator [chuckles]

MU: [laughter]

JE: [chuckles]

SB: That’s all we used to use. We invested in generators so we could uh charge our cell phones

MU: Mm-hm.

SB: and plug in the freezer, but more work, more expensive today.

MU: Mm-hm.

SB: Could you dig near-by that fridge or just make another too? There’d be ice there?

SB: Yeah, there’s, we have several holes in the ground.

JE: Gotcha, wow.

SB: It won’t melt, melt til July, end of July probably.

JE: That's pretty nice.

SB: Yeah, because last year we went home and I came back to do some other things. I checked in there and there was a little bit of ice still in there, last year.

MU: Mm.

JE: Oh I see.

MU: What is the name, is there a Yup'ik word for those?

SB: Teqaq.

JE: How do you spell that?

SB: T-E-Q-A-Q.

JE: for the Yup'ik refrigerator? Oh I forgot to ask you, is that, is that what you identify as is Yup'ik?

SB: Yeah, Yup'ik.

JE: Awesome.

SB: I am Yup'ik and down in the United, US, there's no such thing as Indians, cause that's not India.

JE & MU: [laughter]

JE: Yeah.

SB: [laughter] India's 9000 miles that way.

[group laughter]

MU: You need to get someone a map.

JE: Yeah.

SB: Well,

MU: Quyana

SB: Aang.

JE: Quyana for your time, I'll stop this.
